# Supplementary material for: Barriers and enablers to the implementation of immediate postpartum and post-abortion family planning service integration in primary health care units of Wolaita Zone, Southern Ethiopia: A baseline study for implementation research
Source: PLoS One. 2024 Jul 25;19(7):e0303809. doi: 10.1371/journal.pone.0303809 (PMC11271869; doi:10.1371/journal.pone.0303809)
Supplement: S1 File — (DOCX) [file pone.0303809.s001.docx]

## Annex IV- IDI and FGDs

**In-Depth Interview: Guidelines**

***Purpose:*** *Discussion* ***Guide for recently delivered mothers***

***Tool Notes:*** *The facilitator should read out the consent form and take informed consent for participation. Instructions to the interviewer are in italics.*

**Consent Form for Adult Participant (IDI)**

**Introduction**

Greetings, my name is *<name>,* I am a member of research team of School of Public Health, Addis Ababa University. We are doing this research in collaboration with Engender Health among PP and PA FP clients, providers, and other stakeholders.

**Purpose**

The purpose of the study is to understand the socio-cultural and health system barriers and enablers to use of PP and PA family planning services in two regions in Ethiopia.

**Procedures:**

If you agree to participate in the study, we will ask you to participate in an in-depth discussion. The discussion would take about an hour. We will record the discussion for future reference and analysis.

**Risks:**

There is no risk in the study. To keep your identity confidential, we would not share your name and other identification details. However, if you feel uncomfortable at any point of time, you may discontinue the discussion.

**Benefits & Compensation:**

You will not receive any payment or compensation to participate in this study. There is no direct benefit to you for participating in this study. However, the information that we receive will help us in improving the programs for PP/PA family planning services.

**Confidentiality:**

We will do everything we can to keep the information you share with us, confidential. We will not share the responses with anyone outside the research team. The results of the study will be used to make the program for PP/PA family planning services better. You will not be identified in any publications or presentations resulting from this study. The computer files will be de-identified, meaning no one, not even the research team, will be able to link responses with participants and stored for three years after the study has ended. The list that will have your name and code number will be stored securely.

**Voluntary Participation:**

Participation in this study is completely voluntary, that means you decide whether you want to participate or not. If you decide not to participate, there will be no consequences. If you decide not to take part or leave it in between, we would respect your decision and not ask you any questions regarding your decision.

**Contact Information:**

In case you have any questions about the study at any time, you may contact Ms. Charu Sheela, at 0120-4206545 or email at [charu.sheela@craftconsulting.in](mailto:charu.sheela@craftconsulting.in). If you any questions about your rights as a research subject you may contact Dr. Priyanka Kochar, EngenderHealth at (011) 26526007 or email at PKochar@engenderhealth.org. If you have questions regarding ethical issues, you may contact Ms. S. Prema Swarupa of CONVERGENT-IRB at 0120-4125163 E-mail: [prema.swarupa@convergentview.com](mailto:prema.swarupa@convergentview.com)

***Consent (to be audio recorded):***

| Do you understand the purpose of this research? | 🞎 Yes | 🞎 No |
| --- | --- | --- |
| Should we explain the process again? | 🞎 Yes | 🞎 No |
| Do you have any questions? | 🞎 Yes | 🞎 No |
| Do you give your consent to participate in the study? | 🞎 Yes | 🞎 No |
| Do you need the copy of consent form? | 🞎 Yes | 🞎 No |
| Do you give your consent for audio recording? | 🞎 Yes | 🞎 No |

**Discussion**

**Rapport Building**

Thank you for proving consent to participate in the discussion. There are no right or wrong answers, please share your frank opinion, as it will help us in understanding the situation better.

*Enquire about her recent delivery experience. An example is given below:*

Please tell us about your recent childbirth experience. Where did you deliver? Who attended you during delivery? If you deliver at health facility how far is it from your home? How do you travel to the health facility?

**Assets and Resources**

**Discussion**

Now we are going to discuss about the Healthcare resources and assets available to recently delivered mothers in your area.

1. Health services (PP family planning services) availability: Can you tell me the types of healthcare facilities that provide PP family planning services in your area?
   1. What types of FP services are available at the healthcare facilities?
   2. Are these services equally available to boys, girls, young, old, married, unmarried, people from different geographic locations, religious or cultural background, language etc.? Please tell me more
2. Accessibility of the PP family planning services
   1. Can you tell me how far the PP family planning services providing facility is located? How long does it take for you to access the facility?
   2. What type of transportation means do you use to reach the facility? How easy or difficult is it to use the transportation services?
   3. Is there any cost associated with accessing the PP family planning services? Please tell me more about this
   4. Is the PP family planning service open at all time and is accessible with short waiting time at the healthcare facility? Are there types of FP methods of users’ choice in the healthcare facility? Do healthcare providers receive and treat users with respect and dignity?
   5. Are these services equally accessible to boys, girls, young, old, married, unmarried, people from different geographic locations, religious or cultural background, language etc.? Please tell me more
   6. How easy could you access the PP family planning service? Do you think your access to the PP family planning services is affected because of your background? How?
3. Means to access the PP family planning services
   1. Please tell me about your household resources and assets. Please tell me the resources and assets belong to you.
   2. How is the use of the household resources and assets decided? Who makes the decision? Do you involve in the decision making? How do you think that affects your access to the healthcare services including PP family planning?
   3. Please tell me about your household income. Please tell me about your own income.
   4. How is the use of the household income decided? Who makes the decision? Do you involve in the decision making? How do you think that affects your access to the healthcare services including PP family planning?
   5. In your community how is use of PP family planning seen? Is it acceptable for women to use PP family planning? Why/why not? Please tell me more
4. In your opinion what should be done to improve access to the PP family planning services for all recently delivered mothers in your community?

**Practices, Roles, and Participation**

Now we are going to discuss about your women’s and your role and responsibilities.

1. Please describe to me the typical women’s roles in your community?
   - How are women and girls occupied over the course of 24 hours? Are there seasonal differences in how women and girls use their time?
2. What kind of work do you do? Do these works change when your age or status changes (young, unmarried and married, older)?
3. Do you have restrictions on your mobility? What restrictions? How do the restrictions influence your access to the PP family planning services and supportive social networks?
   - In your community do the restriction change by social characteristic, such as (dis)ability, economic status, health status, educational level, religion, culture, geographical location, and marital status?
4. Do you participate in any family, community, government, or other social networks organizations? Please tell me more.
   - Please tell me how your participation in these organizations affect your PP family planning services use
5. In your community do women assume leadership roles? What types of roles? Do these roles change by social characteristics, such as (dis)ability, economic status, health status, educational level, religion, culture, geographical location, and marital status?
   - Please tell us about your own experience with assuming any leadership roles?
   - Please tell me how your leadership roles affect your PP family planning services use
6. In your community who decides at what age people marry? What are the reasons for getting married at younger or older ages?
   - Please tell us about your own marriage experience? How old were you when you married? What are the reasons you married at that age?
   - Please tell me how your age at marriage affects your PP family planning services use
7. What are your suggestions to improve women’s roles and participation to enhance the use of PP family planning services?

**Knowledge, Beliefs, and Perceptions**

Now we are going to discuss about the issues faced by adolescents in accessing SRH services in your opinion.

1. In your community what is appropriate behavior for a woman? How do they differ from adolescent girls and young women?
   1. What is the appropriate behavior for a recently delivered mother? How do these believe influence health behavior including the PP family planning use?
2. What are the social beliefs and perceptions that share women’s expectations and aspiration for health care including PP family planning?
3. In your opinion who should make decisions regarding use of PP family planning services? What types of decisions do women make regarding use of healthcare including PP family planning services? What types of decisions regarding use of healthcare including PP family planning services are made jointly?

**Legal Rights and Status**

Now we are going to discuss about the marriage related process prevalent in this community.

1. In your community are women who delivered recently legally allowed to receive PP family planning service?

- Is these same for boys and girls, people from different age groups, education background, ethnic groups, socio economic classes, excluded groups? How are they different? Why/ Why not?

1. How do recently delivered women access PP family planning information and resource from the healthcare facilities?

- Is these same for boys and girls, people from different age groups, education background, ethnic groups, socio economic classes, excluded groups? How are they different? Why/ Why not?
- Do the people in your community support recently delivered women to access PP family planning infatuation and resources? How do they provide support? Who are these people? Why do they provide support?
- Are there certain groups in your community who do not support this? Who are these people? Why don’t they support?

1. Please tell me your experience with accessing healthcare information and resources including PP family planning.

- Do you feel you have a right to get healthcare information and resources including PP family planning services? Why/why not?
- Do you think your experience is different from other people in your community? How? Please tell me more
- Is there legal mechanism you can follow to ensure you get quality healthcare information and resources your need? Please tell me more

With this we have come to an end of the discussion. Do you want to add anything before we end the discussion or if do you have any questions you want to ask?

Thank you for your time and in case you want, you can get in touch with us through the details mentioned in the consent form.

*********

**In-Depth Interview: Guidelines**

***Purpose:*** *Discussion Guide* ***for* Regional health bureau focal person**

***Tool Notes:*** *The facilitator should read out the consent form and take informed consent for participation. Instructions to the interviewer are in italics.*

**Consent Form for Adult Participant (IDI)**

**Introduction**

Greetings, my name is *<name>,* I am a member of research team of School of Public Health, Addis Ababa University. We are doing this research in collaboration with EngenderHealth among PP and PA FP clients, providers, and other stakeholders.

**Purpose**

The purpose of the study is to understand the socio-cultural and health system barriers and enablers to use of PP and PA family planning services in two regions in Ethiopia.

**Procedures:**

If you agree to participate in the study, we will ask you to participate in an in-depth discussion. The discussion would take about an hour. We will record the discussion for future reference and analysis.

**Risks:**

There is no risk in the study. To keep your identity confidential, we would not share your name and other identification details. However, if you feel uncomfortable at any point of time, you may discontinue the discussion.

**Benefits & Compensation:**

You will not receive any payment or compensation to participate in this study. There is no direct benefit to you for participating in this study. However, the information that we receive will help us in improving the programs for PP/PA family planning services.

**Confidentiality:**

We will do everything we can to keep the information you share with us, confidential. We will not share the responses with anyone outside the research team. The results of the study will be used to make the program for PP/PA family planning services better. You will not be identified in any publications or presentations resulting from this study. The computer files will be de-identified, meaning no one, not even the research team, will be able to link responses with participants and stored for three years after the study has ended. The list that will have your name and code number will be stored securely.

**Voluntary Participation:**

Participation in this study is completely voluntary, that means you decide whether you want to participate or not. If you decide not to participate, there will be no consequences. If you decide not to take part or leave it in between, we would respect your decision and not ask you any questions regarding your decision.

**Contact Information:**

In case you have any questions about the study at any time, you may contact Ms. Charu Sheela, at 0120-4206545 or email at [charu.sheela@craftconsulting.in](mailto:charu.sheela@craftconsulting.in). If you any questions about your rights as a research subject you may contact Dr. Priyanka Kochar, EngenderHealth at (011) 26526007 or email at PKochar@engenderhealth.org. If you have questions regarding ethical issues, you may contact Ms. S. Prema Swarupa of CONVERGENT-IRB at 0120-4125163 E-mail: [prema.swarupa@convergentview.com](mailto:prema.swarupa@convergentview.com)

***Consent (to be audio recorded):***

| Do you understand the purpose of this research? | 🞎 Yes | 🞎 No |
| --- | --- | --- |
| Should we explain the process again? | 🞎 Yes | 🞎 No |
| Do you have any questions? | 🞎 Yes | 🞎 No |
| Do you give your consent to participate in the study? | 🞎 Yes | 🞎 No |
| Do you need the copy of consent form? | 🞎 Yes | 🞎 No |
| Do you give your consent for audio recording? | 🞎 Yes | 🞎 No |

**Discussion**

**Rapport Building**

Thank you for proving consent to participate in the discussion. There are no right or wrong answers, please share your frank opinion, as it will help us in understanding the situation better.

*Enquire about her recent delivery experience. An example is given below:*

Please tell us about family planning service in your region. Where did women get family planning services? Who provided the services? How do women travel to the health facility?

**Assets and Resources**

**Discussion**

Now we are going to discuss about the Healthcare resources and assets available for provision of PP and PA family planning services in your region.

1. Health services (PP and PA family planning services) availability: Can you tell me the types of healthcare facilities that provide PP and PA family planning services in your region?
   1. What types of FP services are available at the healthcare facilities?
   2. Are these services equally available to boys, girls, young, old, married, unmarried, people from different geographic locations, religious or cultural background, language etc.? Please tell me more
2. Accessibility of the PP and PA family planning services
   1. Can you tell me how far the PP and PA family planning services providing facilities are located? How long does it take for women to access the facilities?
   2. What type of transportation means do they use to reach the facilities? How easy or difficult is it to use the transportation services?
   3. Is there any cost associated with accessing the PP and PA family planning services? Please tell me more about this
   4. Are these services equally accessible to boys, girls, young, old, married, unmarried, people from different geographic locations, religious or cultural background, language etc.? Please tell me more
3. Means to access the PP and PA family planning services
   1. Please tell me about your region’s resources and assets. Please tell me the resources and assets belong to each of the health facilities in your region.
   2. How is the use of the region resources and assets decided? Who makes the decision? Do women involve in the decision making? How do you think that affects access to the healthcare services including PP and PA family planning by women?
   3. Please tell me about income of the health facilities in this region. Please tell me about your region’s income.
   4. How is the use of the region’s income decided? Who makes the decision? Do women involve in the decision making? How do you think that affects their access to the healthcare services including PP and PA family planning?
4. In your opinion what should be done to improve access to the PP and PA family planning services for all women in your region?

**Practices, Roles, and Participation**

Now we are going to discuss about your role and responsibilities.

1. Please describe to me the typical regional health bureau focal person roles in your region?
   1. How are regional health bureau focal persons occupied over the course of 24 hours?
2. As regional health bureau focal person, what kind of work do you do? Do these works different when facility status changes (Rural, urban and geographical locations)?
3. Do regional health bureau focal person like you have restrictions on the mobility? What restrictions? How do the restrictions influence the access of the PP and PA family planning services and supportive social networks?
   1. In your regiondo the restriction change by social characteristic, such as (dis)ability, economic status, health status, educational level, religion, culture, geographical location, and marital status?
4. Do you participate in any family, community, government, or other social networks organizations? Please tell me more.
   1. Please tell me how your participation in these organizations affect the PP and PA family planning services provision
5. In your region do women assume leadership roles? What types of roles? Do these roles change by social characteristics, such as (dis)ability, economic status, health status, educational level, religion, culture, geographical location, and marital status?
   1. Please tell us about your own experience with assuming any leadership roles?
   2. Please tell me how your leadership roles affect the PP and PA family planning services provision
6. In your region who decides at what age people marry? What are the reasons for getting married at younger or older ages in this region?
   1. Please tell me how their age at marriage affects their PP and PA family planning services use
7. What are your suggestions to improve women’s roles and participation to enhance the use of PP and PA family planning services?

**Knowledge, Beliefs, and Perceptions**

Now we are going to discuss about the issues faced by women in accessing PP and PA family planning services in your opinion.

1. In your region what is appropriate behavior for a woman? How do they differ from adolescent girls and young women?
   1. What is the appropriate behavior for a woman in reproductive age group? How do these believe influence health behavior including the PP and PA family planning use?
2. What are the social beliefs and perceptions that share women’s expectations and aspiration for health care including PP and PA family planning?
3. In your opinion who should make decisions regarding use of PP and PA family planning services? What types of decisions do regional health bureau focal person make regarding use of healthcare including PP and PA family planning services? What types of decisions regarding use of healthcare including PP and PA family planning services are made jointly?

**Legal Rights and Status**

Now we are going to discuss about the marriage related process prevalent in this woreda.

1. In your woreda are women in reproductive age group legally allowed to receive PP and PA family planning service?

- Is these same for boys and girls, people from different age groups, education background, ethnic groups, socio economic classes, excluded groups? How are they different? Why/ Why not?

1. How do women in reproductive age group access PP and PA family planning information and resource from the healthcare facilities?

- Is these same for boys and girls, people from different age groups, education background, ethnic groups, socio economic classes, excluded groups? How are they different? Why/ Why not?
- Do the people in your woreda, support women in reproductive age group to access PP and PA family planning infatuation and resources? How do they provide support? Who are these people? Why do they provide support?
- Are there certain groups in your woreda who do not support this? Who are these people? Why don’t they support?

1. Please tell me your experience with accessing healthcare information and resources including PP and PA family planning.

- Do you feel you or your partner have a right to get healthcare information and resources including PP and PA family planning services? Why/why not?
- Do you think your experience is different from other people in your woreda? How? Please tell me more
- Is there legal mechanism you can follow to ensure women get quality healthcare information and resources they need? Please tell me more

With this we have come to an end of the discussion. Do you want to add anything before we end the discussion or if do you have any questions you want to ask?

Thank you for your time and in case you want, you can get in touch with us through the details mentioned in the consent form.

*********

**In-Depth Interview: Guidelines**

***Purpose:*** *Discussion Guide for* ***Woreda health office expert***

***Tool Notes:*** *The facilitator should read out the consent form and take informed consent for participation. Instructions to the interviewer are in italics.*

**Consent Form for Adult Participant (IDI)**

**Introduction**

Greetings, my name is *<name>,* I am a member of research team of School of Public Health, Addis Ababa University. We are doing this research in collaboration with Engender Health among PP and PA FP clients, providers, and other stakeholders.

**Purpose**

The purpose of the study is to understand the socio-cultural and health system barriers and enablers to use of PP and PA family planning services in two regions in Ethiopia.

**Procedures:**

If you agree to participate in the study, we will ask you to participate in an in-depth discussion. The discussion would take about an hour. We will record the discussion for future reference and analysis.

**Risks:**

There is no risk in the study. To keep your identity confidential, we would not share your name and other identification details. However, if you feel uncomfortable at any point of time, you may discontinue the discussion.

**Benefits & Compensation:**

You will not receive any payment or compensation to participate in this study. There is no direct benefit to you for participating in this study. However, the information that we receive will help us in improving the programs for PP/PA family planning services.

**Confidentiality:**

We will do everything we can to keep the information you share with us, confidential. We will not share the responses with anyone outside the research team. The results of the study will be used to make the program for PP/PA family planning services better. You will not be identified in any publications or presentations resulting from this study. The computer files will be de-identified, meaning no one, not even the research team, will be able to link responses with participants and stored for three years after the study has ended. The list that will have your name and code number will be stored securely.

**Voluntary Participation:**

Participation in this study is completely voluntary, that means you decide whether you want to participate or not. If you decide not to participate, there will be no consequences. If you decide not to take part or leave it in between, we would respect your decision and not ask you any questions regarding your decision.

**Contact Information:**

In case you have any questions about the study at any time, you may contact Ms. Charu Sheela, at 0120-4206545 or email at [charu.sheela@craftconsulting.in](mailto:charu.sheela@craftconsulting.in). If you any questions about your rights as a research subject you may contact Dr. Priyanka Kochar, EngenderHealth at (011) 26526007 or email at PKochar@engenderhealth.org. If you have questions regarding ethical issues, you may contact Ms. S. Prema Swarupa of CONVERGENT-IRB at 0120-4125163 E-mail: [prema.swarupa@convergentview.com](mailto:prema.swarupa@convergentview.com)

***Consent (to be audio recorded):***

| Do you understand the purpose of this research? | 🞎 Yes | 🞎 No |
| --- | --- | --- |
| Should we explain the process again? | 🞎 Yes | 🞎 No |
| Do you have any questions? | 🞎 Yes | 🞎 No |
| Do you give your consent to participate in the study? | 🞎 Yes | 🞎 No |
| Do you need the copy of consent form? | 🞎 Yes | 🞎 No |
| Do you give your consent for audio recording? | 🞎 Yes | 🞎 No |

**Discussion**

**Rapport Building**

Thank you for proving consent to participate in the discussion. There are no right or wrong answers, please share your frank opinion, as it will help us in understanding the situation better.

*Enquire about her recent delivery experience. An example is given below:*

Please tell us about family planning service in your woreda. Where did women get family planning services? Who provided the services? How far is the health facility from women’s home (in different directions? How do women travel to the health facility?

**Assets and Resources**

**Discussion**

Now we are going to discuss about the Healthcare resources and assets available for provision of PP and PA family planning services in your area.

1. Health services (PP and PA family planning services) availability: Can you tell me the types of healthcare facilities that provide PP and PA family planning services in your area?
   1. What types of FP services are available at the healthcare facilities?
   2. Are these services equally available to boys, girls, young, old, married, unmarried, people from different geographic locations, religious or cultural background, language etc.? Please tell me more
2. Accessibility of the PP and PA family planning services
   1. Can you tell me how far the PP and PA family planning services providing facilities are located? How long does it take for women to access the facilities?
   2. What type of transportation means do they use to reach the facilities? How easy or difficult is it to use the transportation services?
   3. Is there any cost associated with accessing the PP and PA family planning services? Please tell me more about this
   4. Is the PP and PA family planning service is accessible at all time at the healthcare facilities? Are there types of FP methods of users’ choice in the healthcare facilities?
   5. Are these services equally accessible to boys, girls, young, old, married, unmarried, people from different geographic locations, religious or cultural background, language etc.? Please tell me more
   6. How easy could they access the PP and PA family planning services? Do you think that their access to the PP and PA family planning services are affected because of their background? How?
3. Means to access the PP and PA family planning services
   1. Please tell me about your facilities’s resources and assets. Please tell me the resources and assets belong to this health facilities.
   2. How is the use of the facility resources and assets decided? Who makes the decision? Do women involve in the decision making? How do you think that affects access to the healthcare services including PP and PA family planning by women?
   3. Please tell me about income of households in this woreda. Please tell me about your woredas income.
   4. How is the use of the woreda’s income decided? Who makes the decision? Do women involve in the decision making? How do you think that affects their access to the healthcare services including PP and PA family planning?
   5. In your woreda how is use of PP and PA family planning seen? Is it acceptable for women to use PP and PA family planning? Why/why not? Please tell me more
4. In your opinion what should be done to improve access to the PP and PA family planning services for all women in your community?

**Practices, Roles, and Participation**

Now we are going to discuss about your women’s and your role and responsibilities.

1. Please describe to me the typical woreda health office officials roles in your community?
   1. How are woreda health office officials occupied over the course of 24 hours? Are there seasonal differences in how women and girls use their time?
2. As woreda health office official, what kind of work do you do? Do these works different when facility status changes (Rural, urban and geographical locations)?
3. Do woreda health office officials like you have restrictions on the mobility? What restrictions? How do the restrictions influence the access of the PP and PA family planning services and supportive social networks?
   1. In your woreda do the restriction change by social characteristic, such as (dis)ability, economic status, health status, educational level, religion, culture, geographical location, and marital status?
4. Do you participate in any family, community, government, or other social networks organizations? Please tell me more.
   1. Please tell me how your participation in these organizations affect the PP and PA family planning services provision
5. In your woreda do women assume leadership roles? What types of roles? Do these roles change by social characteristics, such as (dis)ability, economic status, health status, educational level, religion, culture, geographical location, and marital status?
   1. Please tell us about your own experience with assuming any leadership roles?
   2. Please tell me how your leadership roles affect the PP and PA family planning services provision
6. In your woreda who decides at what age people marry? What are the reasons for getting married at younger or older ages in this woreda?
   1. Please tell me how their age at marriage affects their PP and PA family planning services use
7. What are your suggestions to improve women’s roles and participation to enhance the use of PP and PA family planning services?

**Knowledge, Beliefs, and Perceptions**

Now we are going to discuss about the issues faced by women in accessing SRH services in your opinion.

1. In your woreda what is appropriate behavior for a woman? How do they differ from adolescent girls and young women?
   1. What is the appropriate behavior for a woman in reproductive age group? How do these believe influence health behavior including the PP and PA family planning use?
2. What are the social beliefs and perceptions that share women’s expectations and aspiration for health care including PP and PA family planning?
3. In your opinion who should make decisions regarding use of PP and PA family planning services? What types of decisions do women make regarding use of healthcare including PP and PA family planning services? What types of decisions regarding use of healthcare including PP and PA family planning services are made jointly?

**Legal Rights and Status**

Now we are going to discuss about the marriage related process prevalent in this community.

1. In your community are women who delivered recently legally allowed to receive PP family planning service?

- Is these same for boys and girls, people from different age groups, education background, ethnic groups, socio economic classes, excluded groups? How are they different? Why/ Why not?

1. How do recently delivered women access PP family planning information and resource from the healthcare facilities?

- Is these same for boys and girls, people from different age groups, education background, ethnic groups, socio economic classes, excluded groups? How are they different? Why/ Why not?
- Do the people in your community support recently delivered women to access PP family planning infatuation and resources? How do they provide support? Who are these people? Why do they provide support?
- Are there certain groups in your community who do not support this? Who are these people? Why don’t they support?

1. Please tell me your experience with accessing healthcare information and resources including PP family planning.

- Do you feel you have a right to get healthcare information and resources including PP family planning services? Why/why not?
- Do you think your experience is different from other people in your community? How? Please tell me more
- Is there legal mechanism you can follow to ensure you get quality healthcare information and resources your need? Please tell me more

With this we have come to an end of the discussion. Do you want to add anything before we end the discussion or if do you have any questions you want to ask?

Thank you for your time and in case you want, you can get in touch with us through the details mentioned in the consent form.

*********

**In-Depth Interview: Guidelines**

***Purpose:*** *Discussion Guide* ***for Woreda health office officials***

***Tool Notes:*** *The facilitator should read out the consent form and take informed consent for participation. Instructions to the interviewer are in italics.*

**Consent Form for Adult Participant (IDI)**

**Introduction**

Greetings, my name is *<name>,* I am a member of research team of School of Public Health, Addis Ababa University. We are doing this research in collaboration with EngenderHealth among PP and PA FP clients, providers, and other stakeholders.

**Purpose**

The purpose of the study is to understand the socio-cultural and health system barriers and enablers to use of PP and PA family planning services in two regions in Ethiopia.

**Procedures:**

If you agree to participate in the study, we will ask you to participate in an in-depth discussion. The discussion would take about an hour. We will record the discussion for future reference and analysis.

**Risks:**

There is no risk in the study. To keep your identity confidential, we would not share your name and other identification details. However, if you feel uncomfortable at any point of time, you may discontinue the discussion.

**Benefits & Compensation:**

You will not receive any payment or compensation to participate in this study. There is no direct benefit to you for participating in this study. However, the information that we receive will help us in improving the programs for PP/PA family planning services.

**Confidentiality:**

We will do everything we can to keep the information you share with us, confidential. We will not share the responses with anyone outside the research team. The results of the study will be used to make the program for PP/PA family planning services better. You will not be identified in any publications or presentations resulting from this study. The computer files will be de-identified, meaning no one, not even the research team, will be able to link responses with participants and stored for three years after the study has ended. The list that will have your name and code number will be stored securely.

**Voluntary Participation:**

Participation in this study is completely voluntary, that means you decide whether you want to participate or not. If you decide not to participate, there will be no consequences. If you decide not to take part or leave it in between, we would respect your decision and not ask you any questions regarding your decision.

**Contact Information:**

In case you have any questions about the study at any time, you may contact Ms. Charu Sheela, at 0120-4206545 or email at [charu.sheela@craftconsulting.in](mailto:charu.sheela@craftconsulting.in). If you any questions about your rights as a research subject you may contact Dr. Priyanka Kochar, EngenderHealth at (011) 26526007 or email at PKochar@engenderhealth.org. If you have questions regarding ethical issues, you may contact Ms. S. Prema Swarupa of CONVERGENT-IRB at 0120-4125163 E-mail: [prema.swarupa@convergentview.com](mailto:prema.swarupa@convergentview.com)

***Consent (to be audio recorded):***

| Do you understand the purpose of this research? | 🞎 Yes | 🞎 No |
| --- | --- | --- |
| Should we explain the process again? | 🞎 Yes | 🞎 No |
| Do you have any questions? | 🞎 Yes | 🞎 No |
| Do you give your consent to participate in the study? | 🞎 Yes | 🞎 No |
| Do you need the copy of consent form? | 🞎 Yes | 🞎 No |
| Do you give your consent for audio recording? | 🞎 Yes | 🞎 No |

**Discussion**

**Rapport Building**

Thank you for proving consent to participate in the discussion. There are no right or wrong answers, please share your frank opinion, as it will help us in understanding the situation better.

*Enquire about her recent delivery experience. An example is given below:*

Please tell us about family planning service in your woreda. Where did women get family planning services? Who provided the services? How far is the health facility from women’s home (in different directions? How do women travel to the health facility?

**Assets and Resources**

**Discussion**

Now we are going to discuss about the Healthcare resources and assets available for provision of PP and PA family planning services in your woreda.

1. Health services (PP and PA family planning services) availability: Can you tell me the types of healthcare facilities that provide PP and PA family planning services in your area?
   1. What types of FP services are available at the healthcare facilities?
   2. Are these services equally available to boys, girls, young, old, married, unmarried, people from different geographic locations, religious or cultural background, language etc.? Please tell me more
2. Accessibility of the PP and PA family planning services
   1. Can you tell me how far the PP and PA family planning services providing facilities are located? How long does it take for women to access the facilities?
   2. What type of transportation means do they use to reach the facilities? How easy or difficult is it to use the transportation services?
   3. Is there any cost associated with accessing the PP and PA family planning services? Please tell me more about this
   4. Are these services equally accessible to boys, girls, young, old, married, unmarried, people from different geographic locations, religious or cultural background, language etc.? Please tell me more
3. Means to access the PP and PA family planning services
   1. Please tell me about your woreda’s resources and assets. Please tell me the resources and assets belong to each of the health facilities in your woreda.
   2. How is the use of the woreda resources and assets decided? Who makes the decision? Do women involve in the decision making? How do you think that affects access to the healthcare services including PP and PA family planning by women?
   3. Please tell me about income of households in this woreda. Please tell me about your woredas income.
   4. How is the use of the woreda’s income decided? Who makes the decision? Do women involve in the decision making? How do you think that affects their access to the healthcare services including PP and PA family planning?
4. In your opinion what should be done to improve access to the PP and PA family planning services for all women in your woreda?

**Practices, Roles, and Participation**

Now we are going to discuss about your role and responsibilities.

1. Please describe to me the typical woreda health office official roles in your woreda?
   1. How are woreda health office officials occupied over the course of 24 hours?
2. As woreda health office official, what kind of work do you do? Do these works different when facility status changes (Rural, urban and geographical locations)?
3. Do woreda health office officials like you have restrictions on the mobility? What restrictions? How do the restrictions influence the access of the PP and PA family planning services and supportive social networks?
   1. In your woreda do the restriction change by social characteristic, such as (dis)ability, economic status, health status, educational level, religion, culture, geographical location, and marital status?
4. Do you participate in any family, community, government, or other social networks organizations? Please tell me more.
   1. Please tell me how your participation in these organizations affect the PP and PA family planning services provision
5. In your woreda do women assume leadership roles? What types of roles? Do these roles change by social characteristics, such as (dis)ability, economic status, health status, educational level, religion, culture, geographical location, and marital status?
   1. Please tell us about your own experience with assuming any leadership roles?
   2. Please tell me how your leadership roles affect the PP and PA family planning services provision
6. In your woreda who decides at what age people marry? What are the reasons for getting married at younger or older ages in this woreda?
   1. Please tell me how their age at marriage affects their PP and PA family planning services use
7. What are your suggestions to improve women’s roles and participation to enhance the use of PP and PA family planning services?

**Knowledge, Beliefs, and Perceptions**

Now we are going to discuss about the issues faced by women in accessing PP and PA family planning services in your opinion.

1. In your woreda what is appropriate behavior for a woman? How do they differ from adolescent girls and young women?
   1. What is the appropriate behavior for a woman in reproductive age group? How do these believe influence health behavior including the PP and PA family planning use?
2. What are the social beliefs and perceptions that share women’s expectations and aspiration for health care including PP and PA family planning?
3. In your opinion who should make decisions regarding use of PP and PA family planning services? What types of decisions do Woreda health office officials make regarding use of healthcare including PP and PA family planning services? What types of decisions regarding use of healthcare including PP and PA family planning services are made jointly?

**Legal Rights and Status**

Now we are going to discuss about the marriage related process prevalent in this woreda.

1. In your woreda are women in reproductive age group legally allowed to receive PP and PA family planning service?

- Is these same for boys and girls, people from different age groups, education background, ethnic groups, socio economic classes, excluded groups? How are they different? Why/ Why not?

1. How do women in reproductive age group access PP and PA family planning information and resource from the healthcare facilities?

- Is these same for boys and girls, people from different age groups, education background, ethnic groups, socio economic classes, excluded groups? How are they different? Why/ Why not?
- Do the people in your woreda, support women in reproductive age group to access PP and PA family planning infatuation and resources? How do they provide support? Who are these people? Why do they provide support?
- Are there certain groups in your woreda who do not support this? Who are these people? Why don’t they support?

1. Please tell me your experience with accessing healthcare information and resources including PP and PA family planning.

- Do you feel you or your partner have a right to get healthcare information and resources including PP and PA family planning services? Why/why not?
- Do you think your experience is different from other people in your woreda? How? Please tell me more
- Is there legal mechanism you can follow to ensure women get quality healthcare information and resources they need? Please tell me more

With this we have come to an end of the discussion. Do you want to add anything before we end the discussion or if do you have any questions you want to ask?

Thank you for your time and in case you want, you can get in touch with us through the details mentioned in the consent form.

*********

**የIDI እና FGD መመሪያዎች**

**ዓላማ፦** በቅርቡ ከወለዱ ሴቶች፣በቅርቡ ከወለዱ ሴቶች ባሎች፣ከታዳጊ ወጣት ወንዶችና ልጃገረዶችጋር IDI እና FGDን ለማካሄድ እንዲረዳ ማድረግ።

**የመጠይቁ ማስታወሻ**፦ ቃለ መጠይቅ አድራጊው የስምምነት ቅጹን ማንበብና በእውቀት ላይ የተመሠረተ ስምምነት መውሰድ ይኖርበታል። ለቃለመጠይቅ አድራጊው የተሰጡ መመሪያዎች በኢታሊክ ተቀምጠዋል።

**የስምምነት ቅጽ ለአዋቂ ተሳታፊ (IDI)**

**መግቢያ**

ሰላም፣ስሜ<*ስም*> ነው፣የአዲስ አበባ ዩኒቨርሲቲ የህረተሰብ ጤና ትምህርት ቤት የምርምር ቡድን አባል ነኝ። ይህን ምርምር በድህረ-ወሊድ እና ድህረ-ዉርጃ ደንበኞች, አገልግሎት ሰጪዎች እና ሌሎች ባለድርሻ አካላት ላይ ከEngenderHealth ጋር በመተባበር እየሰራን ነው፡፡

**ዓላማ**

የጥናቱ ዓላማ በኢትዮጵያ በሁለት ክልሎች የድህረ-ወሊድ እና ድህረ-ዉርጃ የቤተሰብ እቅድ አገልግሎትን ለመጠቀም ያሉ ማህበራዊ -ባህላዊና የጤና ስርዓት መሰናክሎችንና አስቻይ ሁኔታዎችን ለመረዳት ነው።

**አካሄዶች**

በጥናቱ ለመሳተፍ ከተስማሙ ጥልቀት ባለው ውይይት እንዲሳተፉ እንጠይቅዎታለን። ውይይቱ አንድ ሰዓት ገደማ ይፈጃል። ውይይቱን ለወደፊት ማጣቀሻነትና ለትንተና እንመዘግባለን።

**አደጋዎች**

በጥናቱ ላይ ምንም ዓይነት አደጋ የለም። ማንነትዎን ሚስጥራዊ ለማድረግ የእርስዎን ስም እና ሌሎች መለያ ዝርዝሮችን አንጠቀምም፡፡ ይሁን እንጂ በማንኛውም ጊዜ ምቾት ካልተሰማዎት ውይይቱን ሊያቋርጡ ይችላሉ።

**ጥቅሞችና እና ካሳ**

በዚህ ጥናት ለመሳተፍ ምንም ዓይነት ክፍያ ወይም ካሳ አያገኙም። በዚህ ጥናት ስለተሳተፉ ምንም ዓይነት ቀጥተኛ ጥቅም አያገኙም። ይሁን እንጂ የምናገኘው መረጃ የድህረ-ወሊድ እና ድህረ-ዉርጃ የቤተሰብ እቅድ አገልግሎት ፕሮግራሞችን ለማሻሻል ይረዳናል።

**ምስጢራዊነት**

የምታካፍሉንን መረጃ በምሥጢር ለመያዝ የቻልነውን ሁሉ እናደርጋለን። ምላሾቹን ከምርምር ቡድኑ አባላት ውጪ ለማንም አናጋራም። የጥናቱ ውጤች የድህረ-ወሊድ እና ድህረ-ዉርጃ የቤተሰብ እቅድ አገልግሎት ፕሮግራም ለማሻሻል ይጠቅማሉ። ከዚህ ጥናት በሚገኙ በማናቸውም የጥናታዊ ጽሁፍ ህትመቶች ወይም ጥናታ ጽሁፎቹ በሚቀርቡበት ወቅት የእርስዎ ማንነት አይገለጽም። የኮምፒውተር ፋይሎች ተለይተው የማይታወቁ ሲሆን ይህም ማንም ሰው ሌላው ቀርቶ የምርምር ቡድኑ እንኳን ሳይቀሩ ምላሾችን ከተሳታፊዎች ጋር ማገናኘትና ጥናቱ ካበቃ ከሦስት ዓመት በኋላ ማስቀመጥ አይችሉም። የእርስዎ ስም እና ኮድ ቁጥር ያለው ዝርዝር በድብቅ ይቀመጣል፡፡

**የበጎ ፈቃድ ተሳትፎ**

በዚህ ጥናት መሳተፍ ሙሉ በሙሉ በፈቃደኝነት የሚደረግ ነው፡፡ ይህም ማለት ተሳትፎ ማድረግ ይፈልጉ እንደሆነ ወይም እንዳልሆነ ይወስናሉ ማለት ነው። ተሳትፎ ላለማድረግ ቢወስኑም ምንም ዓይነት መዘዝ የለውም። በዚህ ጥናት ላለመካፈል ወይም በመሀል ለማቋረጥ ከወሰኑ የእርስዎን ውሳኔ እናከብራለን፤እንዲሁም ውሳኔዎትን በተመለከተ ምንም ዓይነት ጥያቄ አንጠይቅዎትም።

**የአድራሻ መረጃ**

በማንኛውም ጊዜ ስለጥናቱ ጥያቄ ካለዎት መሰለች አሰግድን በ+251 91 190 4390 ሊያገኟት ይችላሉ ወይም [meselech.assegid@aau.edu.et](mailto:meselech.assegid@aau.edu.et) ላይ ኢሜይል ማድረግ ይችላሉ። እንደምርምር ተሳታፊ መብትዎትን በተመለከተ ማንኛውም ጥያቄ ካለዎት አዲስዓለም ቲትዮስን, EngenderHealth (011) 26526007 ማነጋገር ይችላሉ ወይም[akebede@engenderhealth.org](mailto:akebede@engenderhealth.org) ላይ ኢሜይል ማድረግ ይችላሉ። ከሥነምግባር ጋር በተያያዘ ጥያቄዎች ካለዎት በአዲስ አበባ ዩኒቨርሲቲ የጤና ሳይንስ ኮሌጅ የምርመራ ቦርዱን ስልክ 251-118961396 ወይም ኢ-ሜይል[chs.irb@aau.edu.et](mailto:chs.irb@aau.edu.et)ሊያገኙ ይችላሉ።

***የስምምነት ማረጋገጫ (ለድምጽ ቅጂ):***

| የዚህን ጥናት ዓላማ ተረድተዋል? | 🞎 አዎ | 🞎 አይ |
| --- | --- | --- |
| ሂደቱን በድጋሚ እናስረዳዎት? | 🞎 አዎ | 🞎 አይ |
| ጥያቄ አለዎት? | 🞎 አዎ | 🞎 አይ |
| በጥናቱ ለመሳተፍ ፈቃደኛ ነዎት? | 🞎 አዎ | 🞎 አይ |
| የስምምነት ማረጋገጫ ወረቀት ኮፒ መውሰድ ይፈልጋሉ? | 🞎 አዎ | 🞎 አይ |
| ድምጽዎ እንዲቀዳ ፈቃደኛ ነዎት? | 🞎 አዎ | 🞎 አይ |

**ውይይት**

**ተግባቦት መፍጠሪያ**

በውይይቱ ለመሳተፍ ፈቃደኛ ስለሆኑ እናመሰግናለን።ትክክለኛወይምየተሳሳቱምላሾችየሉም።ሁኔታውንበተሻለመንገድለመረዳትስለሚረዳንእባክዎንጹህአስተያየትዎንአካፍሉ።

በቅርቡ ስላጋጠማት ነገር ጠይቅ/ቂ። አንድ ምሳሌ ከዚህ በታች ተሰጥቷል፤

እባክዎ በቅርብ ጊዜ ስላጋጠመዎትየወሊድልምድ ይንገሩን።የትነው የወለዱት? ማን ነው ያዋለደዎት? በጤና ተቋም ወልደው ከሆነ ከቤትዎ ምን ያህል ይርቃል? ወደ ጤና ተቋም የምትጓዙ እንዴት ነው?

**ንብረቶች እና ሀብቶች**

**ውይይት**

አሁን በአካባቢያችሁ በቅርቡ እናቶች ሲወልዱ ስለነበሩ የጤና ጥበቃ ሀብቶችና ንብረቶች እንወያያለን።

1. የጤና አገልግሎቶች (የድህረ-ወሊድ የቤተሰብ እቅድ አገልግሎቶች) መኖር- በአካባቢዎ የድህረ-ወሊድ የቤተሰብ እቅድ አገልግሎት የሚሰጡትን የጤና ተቋማት ዓይነቶች ሊነግሩኝ ይችላሉ?

ሀ. በጤና ተቋማት ውስጥ ምን ዓይነት የቤተሰብ እቅድ አገልግሎቶች ይገኛሉ?

ለ. እነዚህ አገልግሎቶች ለታዳጊ ወንዶች፣ ለልጃገረዶች፣ ለወጣቶች፣ በእድሜ ለገፉ፣ ላገቡ፣ ላላገቡ፣ በተለያዩ መልክዓ ምድራዊ ቦታዎች ላሉ፣የተለያየ ሃይማኖት ወይም ባህል ወይም ቋንቋ ላላቸው ወዘተ እኩል ሊገኙ ይችላሉን? እባክዎ ተጨማሪ ይንገሩኝ…

2. የድህረ-ወሊድ ቤተሰብ እቅድ አገልግሎት ተደራሽነት

ሀ. የድህረ-ወሊድ የቤተሰብ እቅድ አገልግሎት የሚሰጡ ተቋማት በምን ያህል ርቀት ሊገኙ እንደሚችሉ ሊነግሩኝ ይችላሉ? ወደ ተቋሙ ለመድረስ ምን ያህል ጊዜ ይፈጅብዎታል?

ለ. ወደ ተቋሙ ለመድረስ ምን አይነት የትራንስፖርት አይነት ይጠቀማሉ? የትራንስፖርት አገልግሎቱን መጠቀም ምን ያህል ቀላል ወይም አስቸጋሪ ነው?

ሐ. የድህረ-ወሊድ የቤተሰብ እቅድ አገልግሎቶችን ለማግኘት የሚደረግ ክፍያ ይኖር ይሆን? እባክዎ ስለዚህ ጉዳይ ተጨማሪ ንገሩኝ፡፡

መ. የድህረ-ወሊድ የቤተሰብ እቅድ አገልግሎት ሁልጊዜ ክፍት ነው፡፡ በተቋሙ በአጭር የመጠበቅ ጊዜ አገልግሎቱን ማግኘት የሚቻል ነው? በጤና ተቋማት ውስጥ ተጠቃሚዎች የሚመርጧቸው የቤተሰብ ምጣኔ ዘዴዎች አሉ? የጤና አገልግሎት ሰጪዎች ተጠቃሚዎችን በአክብሮት ተቀብለው ያስተናግዷቸዋል?

ሠ. እነዚህ አገልግሎቶች ለታዳጊ ወንዶች፣ ለልጃገረዶች፣ ለወጣቶች፣ በእድሜ ለገፉ፣ ላገቡ፣ ላላገቡ፣ በተለያዩ መልክዓምድራዊ ቦታዎች ላሉ፣ የተለያየ ሃይማኖት ወይም ባህል ወይም ቋንቋ ላላቸው ወዘተ እኩል ተደራሽ ናቸው? እባክዎ ተጨማሪ ይንገሩኝ…

ረ. የድህረ-ወሊድ የቤተሰብ እቅድ አገልግሎትን እንዴት ባለ መንገድ በቀላሉ ማግኘት ይችላሉ? የእርስዎ የድህረ-ወሊድ የቤተሰብ እቅድ አገልግሎት ተደራሽነት በእርስዎ የኋላ ታሪክ ምክንያት ተጎድቷል/ተነክቷል ብለው ያስባሉ? እንዴት?

3. የድህረ-ወሊድ የቤተሰብ እቅድ አገልግሎት ማግኘት የሚቻልባቸው መንገዶች

ሀ. እባክዎ ስለቤተሰብዎ ሀብትና ንብረት ይንገሩኝ። እባክዎ የእናንተ የሆኑትን ሀብትና ንብረቶች ይንገሩኝ።

ለ. የቤት ውስጥ ሀብትና ንብረት አጠቃቀም የሚወሰነው እንዴት ነው? ይህን ውሳኔ የሚያደርገው ማን ነው? እርስዎ በውሳኔ አሰጣጡ ላይ ይሳተፋሉ? ይህ ሁኔታ የድህረ-ወሊድ የቤተሰብ እቅድን ጨምሮ የጤና አገልግሎት ማግኘትዎን የሚነካው/የሚጎዳው እንዴት ይመስልዎታል?

ሐ. እባክዎ ስለቤተሰብዎ ገቢ ይንገሩኝ። እባክዎ ስለራስዎ ገቢ ይንገሩኝ።

መ. የቤተሰቡ ገቢ አጠቃቀም እንዴት ነው የሚወሰነው? ይህን ውሳኔ የሚያደርገው ማን ነው? እርስዎ በውሳኔ አሰጣጡ ላይ ይሳተፋሉ? ይህ ሁኔታ የድህረ-ወሊድ የቤተሰብ እቅድን ጨምሮ የጤና አገልግሎት ማግኘትዎን የሚነካው/የሚጎዳው እንዴት ይመስልዎታል?

ሠ. በአካባቢያችሁ የድህረ-ወሊድ የቤተሰብ እቅድ መጠቀም የሚታየው እንዴትነው? ሴቶች የድህረ-ወሊድ የቤተሰብ እቅድ መጠቀማቸው ተቀባይነት አለው? አዎ ከሆነ ለምን/ አይደለም ከሆነም ለምን? እባክዎ ተጨማሪ ይንገሩኝ

4. በእርስዎ አስተያየት በእርስዎ ማህበረሰብ ውስጥ በቅርቡ ለወለዱ እናቶች የድህረ-ወሊድ የቤተሰብ እቅድ አገልግሎት ተደራሽነትን ለማሻሻል ምን መደረግ አለበት?

**ልምዶች፣ሚናዎች እና ተሳትፎ**

አሁን ስለ ሴቶቻችሁ እና ስለድርሻችሁ እና ሀላፊነታችሁ እንወያያለን።

1. እባክዎ በእርስዎ ማህበረሰብ ውስጥ የተለመዱ የሴቶች ሚናዎችን ይግለጹልኝ?

- ሴቶችና ልጃገረዶች በ24 ሰዓት ውስጥ እንዴት ተጠምደው ይገኛሉ? ሴቶችና ልጃገረዶች ጊዜያቸውን በሚጠቀሙበት መንገድ ላይ በየወቅቱ ልዩነት አለ?

2. ምን አይነት ስራ ነው የሚሰሩት? ዕድሜዎ ወይም ደረጃዎ ሲለወጥ (ወጣት፣ያላገባናያላገባ፣በዕድሜየገፋ) እነዚህ ሥራዎች ይለወጣሉ?

3. በእንቅስቃሴዎ ላይ ገደብ አለዎት? ምን ዓይነት እገዳዎች ተጥለዋል? እገዳዎቹ የድህረ-ወሊድ የቤተሰብ እቅድ አገልግሎት አጠቃቀምዎ እና ድጋፍ በሚሰጡ ማኅበራዊ ግንኙነቶች ተሳትፎዎ ላይ ምን ተጽዕኖ ያሳድራሉ?

- በእርስዎ ማህበረሰብ ውስጥ እገዳዎቹ በመቻል/ያለመቻል፣ የኢኮኖሚ ሁኔታ፣ የጤናደረጃ፣ የትምህርት ደረጃ፣ ሃይማኖት፣ ባህል፣ የሚኖሩነት መልክዓምድራዊ ቦታ እና የጋብቻ ሁኔታ በመሳሰሉ ማህበራዊ ባህሪያት ይለወጣሉ?

1. በማናቸውም የቤተሰብ፣ የማህበረሰብ፣ የመንግስት ወይም ሌሎች ማህበራዊ ግንኙነት ድርጅቶች ላይ ይሳተፋሉ? እባክዎ ተጨማሪ ይንገሩኝ።

- በእነዚህ ድርጅቶች ውስጥ መሳተፍዎ የእርስዎ የድህረ-ወሊድ የቤተሰብ እቅድ አገልግሎቶች አጠቃቀም ላይ ምን ተጽዕኖ እንደሚያሳድር እባክዎንገሩኝ?

5. በማህበረሰብዎ ውስጥ ሴቶች የአመራር ሚናን ይሸከማሉ? ምን ዓይነት የሥራ ዓይነቶችን? እነዚህ ሚናዎች በመቻል/ያለመቻል፣ የኢኮኖሚሁኔታ፣ የጤናደረጃ፣ የትምህርትደረጃ፣ ሃይማኖት፣ ባህል፣ የሚኖሩነት መልክዓምድራዊ ቦታ እና የጋብቻ ሁኔታ በመሳሰሉ ማህበራዊ ባህሪያት ይለወጣሉ?

- ማንኛውንም የአመራር ሚና ከመሸከም ጋር በተያያዘ የእርስዎን ተሞክሮ ይንገሩን?
- የእርስዎ የአመራርነት ሥራ የድህረ-ወሊድ የቤተሰብ እቅድ አገልግሎቶች አጠቃቀምዎ ላይ ምን ዓይነት ተጽዕኖ እንደሚያሳድር እባክዎ ንገሩኝ

6. በእርስዎ ማህበረሰብ ውስጥ ሰዎች በምን እድሜ ላይ ማጋባት እንዳለባቸው የሚወስነው ማነው? ሰዎች በወጣትነት ዕድሜ ወይም በዕድሜ ከገፉ በኋላ ትዳር የሚመሰርቱባቸው ምክንያቶች ምንድን ናቸው?

- እባክዎ ስለእርስዎ ትዳር ተሞክሮ ይንገሩን? ትዳር ሲመሠርቱ እድሜዎት ስንት ነበር? በዚያ ዕድሜ ያገቡባቸው ምክንያቶች ምንድን ናቸው?
- ትዳር ሲመሰርቱ የነበርዎት ዕድሜ የድህረ-ወሊድ የቤተሰብ እቅድ አገልግሎት አጠቃቀምዎ ላይ ምን ተጽዕኖ እንዳሳደረ እባክዎ ይንገሩኝ?

7. የድህረ-ወሊድ ቤተሰብ እቅድ አገልግሎቶችን አጠቃቀም ለማሻሻል የሴቶችን ሚና እና ተሳትፎ ለማሻሻል የእርስዎ አስተያየቶች ምንድን ናቸው?

**ዕውቀት፣እምነትና ግንዛቤ**

አሁን በእርስዎ አስተያየት የSRH አገልግሎቶችን ለማግኘት በጉርምስና ዕድሜ ላይ የሚገኙ ወጣቶች ስለሚያጋጥሟቸው ችግሮች እንወያያለን።

1. በእርስዎ ማህበረሰብ ውስጥ ለሴት ተገቢ ባህሪይ ምንድንነው? እነርሱ ከልጃገረዶችና ከወጣት ሴቶች የሚለዩት እንዴት ነው?

ሀ. በቅርቡ ለወለደች እናት ተገቢ ባህርይ ምንድን ነው? እነዚህ እምነቶች የድህረ-ወሊድ የቤተሰብ እቅድን ጨምሮ በጤና አጠባበቅ ባህርይ ላይ ምን ተጽዕኖ ያሳድራሉ?

2. የድህረ-ወሊድ የቤተሰብ እቅድን ጨምሮ የሴቶችን የጤና እንክብካቤ ተስፋ እና ምኞት የሚጋሩ ማህበራዊ እምነቶች እና አመለካከቶች ምንድን ናቸው?

3. በእርስዎ አስተያየት የድህረ-ወሊድ የቤተሰብ እቅድ አገልግሎቶችን መጠቀምን በተመለከተ ውሳኔ ማድረግ ያለባቸው እነማን ናቸው? ሴቶች የቤተሰብ እቅድን ጨምሮ የጤና አጠባበቅ አጠቃቀምን በተመለከተ ምን ዓይነት ውሳኔዎች ያደርጋሉ? የቤተሰብ እቅድን ጨምሮ የጤና አጠባበቅ አጠቃቀምን በተመለከተ ምን ዓይነት ውሳኔዎች በጋራ ይወሰናሉ?

**ህጋዊ መብቶች እና ሁኔታ**

አሁን በዚህ ማኅበረሰብ ውስጥ ስለተስፋፋው ከጋብቻ ጋር የተያያዘ ሂደት እንወያያለን።

1. በእርስዎ ማህበረሰብ ውስጥ በቅርቡ የወለዱ ሴቶች የድህረ-ወሊድ የቤተሰብ እቅድ አገልግሎት እንዲጠቀሙ በህጋዊ መንገድ ተፈቅዶላቸዋል?

- ይህ ለታዳጊ ወንዶችና ለታዳጊ ሴቶች፣የተለያየ እድሜ ክልል፣ የትምህርትደረጃ፣ ጎሣ፣ ማህበራዊና የኢኮኖሚ መደብ ውስጥ ላሉ ሰዎች፣ የተገለሉ ቡድኖች ተመሳሳይ ነው? የሚለዩት እንዴት ነው? አዎ ከሆነ ለምን/ አይደለም ከሆነ ለምን?

2. በቅርቡ የወለዱ ሴቶች የድህረ-ወሊድ የቤተሰብ እቅድ መረጃ እና ንብረቶችን ከጤና ተቋማት እንዴት ማግኘት ይችላሉ?

- ይህ ለታዳጊ ወንዶችና ለታዳጊ ሴቶች፣ የተለያየ እድሜ ክልል፣ የትምህርት ደረጃ፣ ጎሣ፣ ማህበራዊና የኢኮኖሚ መደብ ውስጥ ላሉ ሰዎች፣ የተገለሉ ቡድኖች ተመሳሳይ ነው? የሚለዩት እንዴት ነው? አዎ ከሆነ ለምን/ አይደለም ከሆነ ለምን?
- በእርስዎ ማህበረሰብ ውስጥ ያሉ ሰዎች በቅርቡ የወለዱ ሴቶች የድህረ-ወሊድ የቤተሰብ እቅድ ከፍተኛ የሆነ ፍላጎት እና ሀብት እንዲያገኙ ድጋፍ ይሰጧቸዋል? ድጋፍ የሚሰጧቸው እንዴት ነው? እነዚህ ሰዎች እነማን ናቸው? ድጋፍ የሚሰጡት ለምንድን ነው?
- በማህበረሰብዎ ውስጥ ይህንን የማይደግፉ አንዳንድ ቡድኖች አሉ? እነዚህ ሰዎች እነማን ናቸው? ለምን አይደግፉም?

3. እባክዎ የድህረ-ወሊድ የቤተሰብ እቅድን ጨምሮ የጤና ጥበቃ መረጃ እና ሀብቶችን የማግኘት ልምድዎን ይንገሩን።

- የድህረ-ወሊድ የቤተሰብ እቅድ አገልግሎቶችን ጨምሮ የጤና ጥበቃ መረጃና ሀብት የማግኘት መብት እንዳለዎት ይሰማዎታል? አዎ ከሆነ ለምን/ አይደለም ከሆነ ለምን?
- ተሞክሮዎ በአካባቢዎ ካሉ ሌሎች ሰዎች የተለየ ይመስልዎታል? እንዴት? እባክዎ ተጨማሪ ይንገሩን
- የሚያስፈልግዎትን ጥራት ያለው የጤና አጠባበቅ መረጃና ሀብት ለማግኘት ሊከተሉት የሚችሉት ሕጋዊ አሠራር አለ? እባክዎተ ጨማሪ ይንገሩን

በዚህ ወደ ውይይቱ ማብቂያ መጥተናል። ውይይቱን ከማጠናቀቃችን በፊት መጨመር የሚፈልጉት ነገር አለ? ጥያቄዎች ካሉምመጠየቅ ይችላሉ፡፡

ስለሰጡን ጊዜ እናመሰግናለን፡፡ አስፈላጊ ከሆነ በስምምነት ቅጽ ላይ በተጠቀሱት ዝርዝር ጉዳዮች አማካኝነት ከእኛ ጋር መገናኘት ይችላሉ።

*********
