# Supplementary material for: Barriers and enablers to the implementation of immediate postpartum and post-abortion family planning service integration in primary health care units of Wolaita Zone, Southern Ethiopia: A baseline study for implementation research
Source: PLoS One. 2024 Jul 25;19(7):e0303809. doi: 10.1371/journal.pone.0303809 (PMC11271869; doi:10.1371/journal.pone.0303809)
Supplement: S2 File — (PDF) [file pone.0303809.s002.pdf]

**Interview no**	**02**
**Interviewee code**	**IDI _TG_HU_26_7_2022**
**Sex**	**Male**
**Age**	**35**
**Educational status**	**Bsc Dgree**
**Number of children**	**3**
**Marital status**	**Married**
**Name of interviewer**	**Befekadu Bekele**
**Date**	**26/07/2022**
**Time started**	**3:00**
**Time finished**	**3:57**
**Venue**	**Tome Gerera**

Now we are going to discuss about the Healthcare resources and assets available to recently delivered mothers in your area.

I: Health services (PP and PA family planning services) availability: Can you tell me the types of healthcare facilities that provide PP family planning services in your area?

a. What types of FP services are available at the healthcare facilities?
b. Are these services equally available to boys, girls, young, old, married, unmarried, people from different geographic locations, religious or cultural background, language etc.? Please tell me more

R: Thank you so much. This a is nice question. This is third baby in our family. We used to get and use family planning service in the past time. My wife used to use family planning given for three years.After she delivered the first child and she gave birth after three years gap and she used the same method ‘‘ the FP method given for three years’’ and we have got the third child after three year gap. It has been two weeks since my wife delivered the third child. Concerning the importance of family planning it is obvious that it is important for us. previously as we know that family planning is given after forty five days after delivery. But now we heard from you that it is given immediately after a mother gives birth which is very important because pregnancy

may occur accidentally Within forty five days after birth. We have seen that mothers used to suffer because of un planned pregnancy and birth.As you have introduced it is very nice if the service is given immediately after birth because if there is spacing or enough time gap we can take care of our children fulfill what they need. However,” boli bolan yelikko’’ if there is no enough birth spacing,it can bring negative impact on the health of mother and her child too.In my opinion starting family planning immediately after birth is very crucial for us and help the country as a whole. It is important for both mother and her baby. Therefore, attention should be given for this service.

I: Thank you for your response. You have tried to mention some of the Family planning services given. Would you say about the service available, given in the health center? Which types of F/p methods are available in the health center?

R: my wife can say better on the types of family planning methods. But what I know is there is a method given for a month however, we discussed together with my wife and decided to use the long acting given for three years. This method has helped us a lot. Sometimes ,people remove implants early because of lack of knowledge and perceived side effects and they face difficulty after they remove it. “hezzu laytaaway’’ or implant has helped us a lot.

I: has your wife used immediate post partum family planning ?

R:No ,she is waiting until forty five days. She did not start immediate post partum family planning after she gave birth.

I: Are these services(post partum family planning and post abortion? equally available to boys, girls, young, old, married, unmarried, people from different geographic locations, religious or cultural background, language etc.? Please tell me more

R: Yes the service is given for all but there are some challenges related to the services from the community side. Commonly the community say ‘‘*children are gifts from God ,why do I need to use family planning’’*. The community associate the use of family planning with Bible and they *believe that it is against the Bible*. As a result they face different challenges and expose their children for different challenges. *People should not associate the use of family planning with bible and community*. our mothers used to suffer a lot in the past. But now most people are now getting service unlike that of the past time. This should not be seen as something bad because

when a mother uses family planning both mother and her baby is benefited and the service should be continued. Health care workers from our health center and ,health extension workers have been working and going door to door to give different service. However there are resistant people. who are not willing to accept what is being said by health care workers. *Baaqeela giddon issito issito paxoy deiyoogadan daruwa tamarsin paxxa kiya asay dees*".There are people who receive message continuously but not ready to change themselves. But now the behavior people is being changed now. So I can say , what you have said is very important for us.

I: Are there any challenges related to availability? Do you want to add?

R: There is no as such problems.

Accessibility of the PP family planning services

I: Can you tell me how far the PP family planning services providing facility is located? How long does it take for you to access the facility?

R: the health center is very near to us. It takes five minute to travel. Why not fifty minute? since the service is very important ,no matter how the distant it may be , nothing hinder us from using the services. "Distance cannot be a barrier because the benefit we get from the service outweighs."

I: What type of transportation means do you use to reach the facility? How easy or difficult is it to use the transportation services?

R: we don't use any transportation means because it is very close to us.

I:Is there any cost associated with accessing the PP family planning services? Please tell me more about this

R: There is no any associated cost with it because the health center is very near, we don't have transportation cost and the service is free of charge.

I: Is the PP family planning service open at all time and is accessible with short waiting time at the healthcare facility? Are there types of FP methods of users' choice in the healthcare facility? Do healthcare providers receive and treat users with respect and dignity?

R:some times it could be closed ,during lunch time, or weekends. So, in order to solve such problems we have got their phone numbers of health workers and we can call any time and we make sure whether they are available.There is no waiting time.

I: Are there types of FP methods of users' choice in the healthcare facility?

R: when mothers want to use the service they think before what kind of method to used and the health care workers counsel them and tell all the options to choose. They tell the benefits of each method and its side effects.. mothers can sometimes can change mind or decision after they get counseling and adhere to the health providers advices. For example they may decide to use pills while they are at their home but after they get counseling they may change their idea and start long acting family planning methods. So, the health care workers are good, they teach us well.

I:Do healthcare providers receive and treat users with respect and dignity

R: I heard that some people are facing some problems when they come to health center because of shortage of drugs, the health care workers let the clients wait for long time. But this is not related to maternal services. Because it open all the time. The service provision is also fine, there is counseling service being given. There is series follow up, there is no problem with maternal services. The health workers respect and welcoming us. During delivery the health care workers helped us a lot, respect all people regardless of their status.If there are some cases beyond their capacity ,they immediately refer to the higher levels.

I: Are these services equally accessible to boys, girls, young, old, married, unmarried, people from different geographic locations, religious or cultural background, language etc.? Please tell me more

R: The service is given for all mothers. There is a moto ''issi ayyiane maareta gaasuan hayqana koshena'' which means 'no mother should die while she is giving birth.' Both health workers and health extensions are working hard.

Means to access the PP family planning services

I: Please tell me about your household resources and assets.Please tell me the resources and assets belong to you.

R: We have different assets and resources. Regarding source income, we participate in different income generating activities.I am merchant.I have shopes and than God I have other source of income.

I: How is the use of the household resources and assets decided? Who makes the decision? Do you involve in the decision making? How do you think that affects your access to the healthcare services including PP family planning?

R: We discuss and decide jointly. I respect her idea and she also respects my idea. So we discuss on different matters, which family planning method to use, how many children we should have and the like. We don't decide independently.

I: How do you think that affects your access to the healthcare services including PP family planning?

R: Discussing about this matter help us very well for our today as well as future life and it is good for us and our children too. Most of the time decisions are made by husbands without involving mothers and this will bring different problems within family.This can affect access and utilization of family planning services. Therefore, it is good to have discussion and joint decision should be made in the family.Making decision jointly have many advantages for the family.

I: Please tell me about your household income. Please tell me about your own income. How is the use of the household income decided? Who makes the decision? Do you involve in the decision making? How do you think that affects your access to the healthcare services including PP and PA family planning?

R:Approximately my monthly income is thirty thousand birr because I have different sources of income, shopes, I have car and other source of income and the decision is made jointly as I have said earlier.

I: In your community how is use of PP family planning and post abortion F/P seen? Is it acceptable for women to use PP family planning? Why/why not? Please tell me more

R: As I have said earlier it is acceptable for women to use Post partum family planning. However, there are some people who are not willing to use. But people could be able to learn from past experience and the life by itself. As we can see living condition of the community it is not good because of economy. specially ,families having many children are suffering a lot. This can have effect on their children. No body is expected to teach the community because the life(current Living condition) itself teaching the community. As a result ,people who are resistant also started to use the service. According to my observation most of mothers are using the service. Therefore, the community members are asking God to bless the children they have and started to use family planning.

I: What about others?it is acceptable and seen as something good? Are there people who don't support?

R: In my opinion there is no negative attitude from the community side regarding the utilization of PPFP. There is something happened in our vicinity. My neighbor has got pregnant with in forty five days after birth and gave birth. This is not due to negative attitude towards family planning but the mother was waiting until forty five days to start family planning. This has affected her, and her family, children. As you have mentioned family planning should be given immediately after birth and mothers should not wait until forty five days. It is expected from us to inform the community about this. Therefore, people can get lessons from neighbors and start to use the service.

I: In your opinion what should be done to improve access to the PP family planning services for all recently delivered mothers in your community?

R: It needs strong follow up in order to enhance the service. The health extension workers should play their role by visiting during home visits. The health center should also strengthen the health information dissemination. In addition to this those who have already received the message have to teach their family members and convince them. Counseling from health care workers and health extension workers is very crucial. On top of this, mothers should get counseling about PPFP as soon as she gives birth.

Practices, Roles, andParticipation

I: Please describe to me the typical women's roles in your community?

- How are women and girls occupied over the course of 24 hours?Are there seasonal differences in how women and girls use their time?

What kind of work do you do? Do these works change when your age or status changes (young, unmarried and married, older)?

R:previously I have worked in government organization and resigned the work and engaged in private business.In the former case,I was in engaged in different activities ,I used to spend the day time in the office and I stay in the shop during night time. Currently I am fully engaged in my personal businesses.

I: would you describe the typical women's roles in your community?

R: Women have many roles in the community. There is women structure at the kebele level.They have different roles at family level. For example my daughter started to attend school ,so my wife fulfills all what my daughter needs, she prepare food and feeds her and others

I: Do you have barriers on your access? What barriers? How do the barriers/restrictions influence your access to the PP family planning services and PAFP and supportive social networks?

- In your community do the restrictions/barriers change by social characteristic, such as (dis)ability, economic status, health status, educational level, religion, culture, geographical location, and marital status?

R: First of all husband is responsible to take care of his wife and keep the health of his children. The husbands should support their wives very well. Sometimes ,I observe some negligent husbands who don't feel any responsibility, careless husbands. They don't care whether a mother

started to use family planning or not. They have no concern about their wives.Therefore ,husbands can be one of the most important barrier if they don’t support their wives.In addition to this there are some husbands who are not willing and don’t allow their wives to use family planning because they believe that “*children are gifts from God…why are you thinking to use family planning?’’*. I have been engaged in church leadership and observed different challenges. There was one family who were quarreling all the time.We the church leaders went to their house to make reconciliation. We asked the main reason for their conflict and they told as the reason. The mother wants to use family planning and her husband is not willing to do so. The husband says: I don’t allow my wife to use family planning because it is killing life. We appreciated the mother and told them the disadvantages of ‘‘boli bolan yelluwa’’ giving birth without planning. We told them that this can affect the health of the mother and children as well. We also told them that using family planning is not against the word of GOD(the Bible).However her husband continued to resist us and he was not willing to accept our advices and the mother also had firm stand to use family planning but finally this led them to divorce because of this reason. Here ,the husband became a barrier for immediate post partum family planning. Therefore, husbands should understand their wives and the challenge they face and the problem they face if they don’t use family planning methods. Husbands should play vital role.

I: That is nice, you have said about the role of husbands. what should be done to enhance engagement of husbands? What should be done to increase their participation?

R: Training should be given for husbands. Health care providers should train husbands. It is not enough to educate mothers rather husbands should be also involved. Health extension workers should also gather husbands and inform about PPFP and PPA FP services. They should teach about the advantages and the side effects of these methods. Awareness creation should be done. There should be information dissemination and health education in one to five (1:5) and 1:20network.In addition to this kebele administrators should have awareness about this issue.

I: During discussion you have mentioned about what you observed with in church.So what do you think ?what should be done in religious organization?what is their role? In order to enhance the service. You have mentioned some of the thoughts/beliefs related to the use of family planning

R: There are some activities done in our church level. You find kids, adolescents, adults, males and females and aged ones in the church. We special meeting/program with married husbands and their wives. We let husbands to bring their wives when they come to church. We give special counseling for them, we may teach about family planning, how to lead their family, how to respect one another, to keep their personal and environmental hygiene. We teach them to support one another.How to live a peaceful life. Therefore, other religious organizations should follow our trend and teach their congregations, members of the church.

I: you have mentioned about husbands and their role ,how they can be barrier for family planning. Is there anything you want to add regarding barriers? Culture,religion,disability?

R: Nowadays the behavior of people have been changed. Sometimes religion could also be barrier for the FP service utilization

I: How?

R:The are religious sects which don't allow women to use family planning. The teach their members that children are gifts from God and they quote from the bible that children are gifts from the God. They encourage their members to have many children. However ,such thoughts are not existing. As I have mentioned the life itself teaches the community more than anybody else. Therefore many churches are not currently teaching their members not to use family planning rather they encourage to use.

I: what about culture?

R:There is no problem regarding culture in my observation.

I: In your community who decides at what age should people marry? What are the reasons for a women getting married at younger or older ages?

- Please tell us about your own marriage experience? How old were you when you marry? What are the reasons you she get married at that age?
- Please tell me how your age at marriage affects your PP family planning services use

R: It differs from place to place. Sometimes, adolescents withdraw from their school and get married at their earlier stage. sometimes they get married when they are 15. But we advice them to get married after twenty after they become matured. Nowadays children get old early,their age and their physical is opposite. Their age may be 15 but their physical seems old age.That is why they get married as early as possible.

I: What are the reasons for a women getting married at younger or older ages?

R:Mis use of modern technology like mobile phone,sharing their photographs through social media.So that is why they rush for marriage in their early age. Miss use of technology affects the adolescents. They may get married early or the practice sexual intercourse before marriage.

I: In your community who decides at what age should people marry?

R: Most of the time adolescents decide by themselves without discussing with their family.As I have said earlier modern technology pushes them to get married. Most family hear about their daughter after they already get married. They don't have any information about their children.The decision is made by the adolescents.

I: Please tell me how age at marriage affects your PP family planning services use

R: Early marriage by itself is not good. Because both the male or female should have source of income before they get marry and they should plan for their future. They may drop from school for the sake of marriage, this is also a challenge. They may fall in love and say I love you but many problem happens after they get married. As a result, they divorce due to lack of preparation and due to economy. Therefore, before they get married they have to discuss together and secure their source of income.We advice young ages to think about before they get married and this has effect on the post partum and post abortion family planning services.

I: What are your suggestions to improve women's roles and participation to enhance the use of PP family planning services?

R: The role of women is very high to improve the PPFP services. They can play role more than their husbands. They are the one who manage everything in the family.

I:What about husbands role?

R: Both husbands and women have their role but husbands are negligent with this regards. Husbands are selfish and give priority for themselves. Most husbands don't care about their family, their wives, children.

Knowledge, Beliefs, and Perceptions

Now we are going to discuss about the issues faced by adolescents and women in accessing PP and PA FP services in your opinion.

I: In your community what is appropriate behavior for recently delivered woman regarding use of PP and PA family planning services? How do they differ from adolescent girls and young women?

a. What is the appropriate behavior for a recently delivered mother? How do these believe influence health behavior including the PP family planning use?

I: Most of the community has no awareness about post partum and post abortion family planning services. For me using family planning immediately after birth is very nice as compared to waiting until forty five days after birth. This has to be expanded within the community. Teaching the community has brought change in the community. If the community gets information most people will become volunteer to use the services. There could also be some people who have negative attitude. Mothers may feel bad or it may not give comfort for recently delivered mother but the benefit outweighs. ''*It is better if a mother weigh and see the future benefit rather than looking at her pains.''....dreaming bright future than worrying about today's pain.* Therefore, I encourage and I am very happy if PPFP starts immediately after birth. We should teach others to use the services. I hope majority will accept the idea. Few negligent people will accept later. This what we have observed during the initiation of family planning services ,there were people who were willing to use and resist but through time they started to use.

I: In your opinion who should make decisions regarding use of PP family planning services? What types of decisions do women make regarding use of healthcare including PP family

planning services? What types of decisions regarding use of healthcare including PP family planning services are made jointly?

R: Both husband and wife should discuss and decide jointly. That should be done before a mother gives birth. And once they decide they should consult health care workers. Unless they couples decide ,health care workers cannot decide and they don't force people to use.

I:What should they decide? Discuss to gether?

R: For example I have three children, they need food to eat, cloths, school fee. We are responsible to nurture the kids. Unless we use family planning and 'if we practice ''boli boli yeluwa'' if there is no enough time gap, we will not be able to help our kids attend better school. Therefore, both couples should discuss together on different matters. They can discuss about the number of children they should have ,the time gap previous and succeeding one. Unless there would be so challenge for recently delivered mother ,for her previous children and family as a whole. Therefore there should be discussion on how to take care of the current children and children yet to come.

Legal Rights and Status

Now we are going to discuss about the marriage related process prevalent in this community.

I: In your community are women who delivered recently legally allowed to receive PP family planning service?

- Is these same for boys and girls, people from different age groups, education background, ethnic groups, socio economic classes, excluded groups? How are they different? Why/ Why not?

R: yes it is legally allowed. Starting from grassroots level to the higher bodies issues related with family planning given due attention and it is legally allowed to receive PPFP and PAFP. The government and the policy support this idea.

I: How do recently delivered women access PP family planning information and resource from the healthcare facilities?

- Is these same for boys and girls, people from different age groups, education background, ethnic groups, socio economic classes, excluded groups? How are they different? Why/ Why not?

R: They get information from health extension workers during home visit. Health care workers have been also teaching the community and legal bodies have been also informing the community by using different meetings. Therefore, the community is aware of this. People know about all family planning methods give every month, and long acting ones given for three years, five years and 12 years. However the current approach immediate post partum family planning should be introduced into the community.

I: Is these same for boys and girls, people from different age groups, education background, ethnic groups, socio economic classes, excluded groups? How are they different? Why/ Why not?

Do the people in your community support recently delivered women to access PP family planning infatuation and resources? How do they provide support? Who are these people? Why do they provide support?

R: it is difficult to say it is same for all groups. some people may reject you even though you think good for him. Let alone this, people don't equally accept what the preacher preaches in the church ,some accept and others interpret it in different way. There are deviant people who are against to what the listen. Therefore, Health care workers should not lose hope if people don't listen to their advises. They should continuously teach the community until people accept. By nature people don't accept/adopt things as early as possible rather the accept it through process.

I: Are there certain groups in your community who do not support this? Who are these people? Why don't they support? why do they reject?

R: They reject because of lack of knowledge. There is no other reason. As I have said there are people who usually interpret negatively.

Please tell me your experience with accessing healthcare information and resources includingPP family planning.

I: Do you feel you have a right to get healthcare information and resources including PP family planning services? Why/why not?

- Do you think your experience is different from other people in your community? How? Please tell me more

R: yes they have right to get information and resources including PPFP services.

I: Is there legal mechanism you can follow to ensure you get quality healthcare information and resources your need? Please tell me more

R: We should get information from health care workers, health extension workers. I don't think that there are community members who don't access these workers in our kebele.

If we don't get information, there are legal mechanisms where we follow to ensure whether quality health information reached or not. For example if the health extension workers fail to work we inform to the higher bodies and they can take measures.

I: Is there any burning issues left while you were discussing?

R:Thank you so much for coming and interviewing and giving us knowledge. As I have said earlier it has been two weeks since my wife gave birth.I have question regarding immediate post partum family planning because it has been two weeks she gave birth, so I want to be sure with whether it works for us? Or are we expected to wait until 45 days?

I:As I have said earlier when a mother waits until forty five days after delivery pregnancy can occur. immediate use of family planning helps and prevents unplanned pregnancies. Therefore it is possible for your wife to start family planning she may not need to wait until forty five days.

R: can we get the method from the health center?

I: yes you can get from the health center.You can discuss and ask options and start based on your choice and discussion with health care workers your wife can use.

R: Thank you so much.The reason I asked is because we are planning to use family planning as soon as possible and teach others to use.

I: thank you so much.

R: thank you.

Summary

This is interview conducted with husband of recently delivered woman in Tome Gerera

- ✓ There is good understanding of family planning and knows the effect of unintended pregnancy, has also knowledge about appropriate birth spacing ,good for baby ,health of wife
- ✓ Previous history of family planning helped them continue using family planning but did not heard about immediate post partum family planning, that is why she is waiting until 45 days
- ✓ Couples discuss together which methods to use, this helped them to continue
- ✓ Has knowledge on effect of early removal of implants due to side effects as he mentioned there are people who remove, due to lack of knowledge
- ✓ He discourage those who don't use family planning due to misunderstandings, religious and cultural influences and encourage others to use.
- ✓ There are differences in the community behavior, there are some people deviants and don't want to use, attention should be given for this groups.
- ✓ Distance cannot be a barrier for me because the benefit we get from the service outweighs."
- ✓ Joint decision is very important (couples should decide together) but there are problems in the community with this regards
- ✓ People are lacking information about b/c they are not being counseled ,unintended pregnancy is happening even within forty five days interval.They should get counseling soon after birth.
- ✓ husbands can be one of the most important barrier if they don't support their wives,there fore attention should be given for husbands

- ✓ lack of discussion about family planning has brought different challenges in the life of couples,this led them to divorce.
- ✓ Religious organizations should play their role and teach their members about family planning
- ✓ Mis use of modern technology like mobile phone,sharing of photographs through social media led adolescents foe early marriage
- ✓ target audiences should be identified before dissemination of any health messages because there are people who are willing and those who resist.

In-Depth Interview conducted with MCH Focal at Tome gerera health center by Deginesh Dawit

Sociodemographic information of interviewee

Code	Keble	age	sex	Educational-status	Job responsibility	Marriage-status	Work experience
IDI_TGHC_MCHF_260722	Tome Gerera HC	25	F	BSC midwife	MCH focal	Married	6 years

<u>Assets and Resources</u>

<u>Discussion</u>

I; Health services (PP and PA family planning services) availability: Can you tell me the types of healthcare facilities that provide PP and PA family planning services in your area?

R; yes currently this service is available here in Tome health center previously it has not been practiced so far mothers receive the service after forty five days of delivery but since may 2022, the orientation given to health professionals especially who works in MCH class the activities are widely has being practiced. Mothers oriented about this during pregnant conference at community level and in addition to that they has been counselled during their every ANC visit and signs agreement to receive the service after delivery. We classify it as two type the first one given immediately within 10 minute of delivery we call it post placental and the second one is given within forty eight hour of delivery we call it postpartum family planning method. When we come to post abortion family planning, the safe abortion service is being given by trained professionals as our health center and after counselling them the service is also is being given.

I; What types of FP services are available at the healthcare facilities?

R; we have all types of of planning methods including short and long, for example like Jadelle, IUCD, postpills, condum, pills, implanon and Dipo.

I; Are these services equally available to boys, girls, young, old, married, unmarried, people from different geographic locations, religious or cultural background, language etc.? Please tell me more

R; as this health center we have all kinds of family planning methods except the permanent for both males and females, even they need it we counsel them and refer to Hospital

1. Accessibility of the PP and PA family planning services

I; Can you tell me how far the PP and PA family planning services providing facilities are located? How long does it take for women to access the facilities?

R; the two catchement area the so called Wachiqa Busha and Ansome Gembela are the furthest catchment to this health center and for Ansome Gembella to come here it takes around one hour and ten or twenty minutes to come through walking, for Wachiqa Busha it takes around 30 minutes though transportation

I; What type of transportation means do they use to reach the facilities? How easy or difficult is it to use the transportation services?

R; for both of them to receive delivery service they use Ambulance but for ANC visit and family planning services they use their own transportation for the case of Ansome Gembela due to transportation difficulties they used to walk aroun one hour and 20 to 30 minutes for the case of wachiqa Busha they use car and motorcycle symultanously for example to come through concrete road until the turning point they use car transport and after turning point to this health center they use motor cycle if they come though walking after turning point it takes around 30 minutes. Getting transport for both catchment seems difficult , even if they use car they have to walk up to concrete road it is long jorney for those located far from the concrete road and in general getting transportation to come here is difficult.

I; Is there any cost associated with accessing the PP and PA family planning services? Please tell me more about this

R; associated costs related with post-partum and post abortion family planning is only the transport cost if they don't come for delivery service , other than this everything here in health center provided to them including syringe or glove is freely there is no associated cost for the service.

I; Is the PP and PA family planning service is accessible at all time at the healthcare facilities? Are there types of FP methods of users' choice in the healthcare facilities?

R; In tis health center not only MCH class including OPD works twenty four hour as recommendation, so if they come at any time they can get the serve because the professionals works at three shifts so there is no time that MCH class has been closed, even if they come after eleven o'clock and the card class refuse to give them card we convince them to give card by considering the distance where they come

I; Are these services equally accessible to boys, girls, young, old, married, unmarried, people from different geographic locations, religious or cultural background, language etc.? Please tell me more

R; yes it equally accessible for all regardless of their background, but most the as this health center males using family method or condom is not familiarized.

I; How easy could they access the PP and PA family planning services? Do you think that their access to the PP and PA family planning services are affected because of their background? How?

R; it is not easy to provide services to mothers because there is another way they familiarized with and this the new beginning for them so they challenges us and they had misconception related the services, providing the service for those previously aware during ANC and pregnancy conference is not that much difficult but we face some difficulties to convince those who have no ANC visit come from other catchment area and not counselled during ANC relates the service with tradition , through this condition why I take the service, why don't I stay at that time we encourage them to receive the service because they may get unwanted and unplanned pregnancy before the first baby grown up without feeding their baby adequately. But not all receive our counselling those who are educated relatively easily accept our counselling but those who are not formally not accept our counselling at one time.

2. Means to access the PP and PA family planning services

I; Please tell me about your facilities' resources and assets. Please tell me the resources and assets belong to these health facilities to provide PP and PA FP service.

R; as health center our health center has several resources and assets it is one of standardized facility in Sodo Zurya so all of service provision rooms are functional like Abortion service provision room delivery room contains all the required equipment, YFS service. We have all required resources and assets, which need to be existed in health center but related with postpartum family planning we have no IUCD set. We have functional jadelle set. We counsel for IUC also and if the agree to receive it we refer them to hospital setup. We are getting adequate glove after this two months we request the when we lack it they provide us as soon as possible. Regarding post abortion service we have two-trained professional for abortion service and post abortion family service provision in addition to that we have the required set to provide the abortion service. Regarding the professional who trained for this service are two of them specifically trained for IUCD insertion and I can say all professionals who work in MCH class were trained to provide other type of family planning methods because it is not new for professional. Only one professional who is public health expert not received training and refuse to provide the service, so we will solve the problem

I; How is the use of the facility/Woreda health office resources and assets decided? Who makes the decision? Do you involve in the decision making? How do you think that affects access to the healthcare services including PP and PA family planning by women?

R; in general such kind of resources decided by the established management committee here in health center and the materials stored in health center storage then each department focal request their needs in every two weeks and provided accordingly. Before completeness of the available material at store we all department focal asked to request what is needed in our setup at that, the committee buy the required materials according to each department request. As MCH focal am also member of committee, provide request at every two weeks, and get accordingly. My involvement there plays great role because I know in which type of family planning methods people more interested to use, which is more important to us, what we have, what we haven't if I

don't inform such kind of issue to management they have no idea about it, therefore my existence in there have great effect.

I; Please tell me about income of in this Woreda. Please tell me about your woredas income.

R; I have no clear idea about income of this health center because I assigned as MCH focal after decision of annual income, priority they get from patients but now most of people use community based insurance

I; How is the use of the health center income decided? Who makes the decision? Do you involve in the decision-making? How do you think that affects their access to the healthcare services including PP and PA family planning?

R; the income decision made after the request of each department known, the decision makers are the management of this health center, the members are the focal of each department, health center head, finance head, human resource directorate. As a member of management committee I have also my own role in informing them what should be bought for us, by deciding how much money to be allocated to us, to sustain this service and address it to the community.

I; In your woreda how is use of PP and PA family planning seen? Is it acceptable for women to use PP and PA family planning? Why/why not? Please tell me more (what about boys and girls)

R; the utilization of postpartum or post abortion family method is low as this health center since it is new for the community and affected by several misconception. We can see the performance from our weekly or monthly report; it is difficult to say it is good performance. Some times after, they agreed to receive the service during ANC and then become refuse after delivery, currently service utilization is very low but I have hope it will be improved through time. It does get that much acceptance from the community because previously they had another trend that they were familiarized with so bringing behavioral change cannot be achieved within short period. As a beginner, it is good and become well if we strengthen them through pregnant mother's conference, telling them exhaustively, counselling them during ANC visit. The up to now is not adequate an through doing such things I hope it will be improved.

I; In your opinion what should be done to improve access to the PP and PA family planning services for all women in your community?

R; To improve the access building relationship with mothers, health professionals should approach them as friends and family, teaching them exhaustively about the importance of receiving post-partum and post abortion family planning and the associated risk of not receiving it immediately, especially during pregnant mothers conference. Providing mothers who received it later as example or role model letting them to hear from them. Telling them the benefit for children have if one mother take family planning method she can stay at least for two years safely and her child can fed on breast adequately and also if possible providing mothers who suffered from bearing child without birth spacing. In addition to teaching them in pregnant mothers conference counselling them during ANC visit is also another appropriate option for them, home-to-home visit is also other one especially for those not present during pregnant mothers conference

Practices, Roles, and Participation

I; Please describe to me the typical MCH focal roles in your Woreda/community?

R*; my role as MCH focal in this health center is setting program for health professionals in the MCH class, checking up the required materials whether we have or not, monitoring whether the activities are done according to the plan set, allocating professionals to participate in pregnant mothers conference, arranging meeting on some issues and making meeting, and overall controlling the activities is being done at MCH class.*

I; How is Woreda health office officials occupied over the course of the day? Are there seasonal differences (campaign/political assignment) in how you use time?

R; my daily activities are controlling the overall activities done in our health center and working per as schedule as professional. I face work burden sometimes like when one or two professional go to training I have to cover their places

I; As health center MCH focal what kind of work do you do? Do these works different when facility status changes (Rural, urban and geographical locations)?

R; *; my role as MCH focal in this health center is setting program for health professionals in the MCH class, checking up the required materials whether we have or not, monitoring whether the*

activities are done according to the plan set, allocating professionals to participate in pregnant mothers conference, arranging meeting on some issues and making meeting, and overall controlling the activities is being done at MCH class. Yes, it is not similar everywhere because most of the time people live in urban area are more of educated and it is not difficult to us to convince them and give the service. However, mothers resided in rural areas are not formally educated most of the time and not can get sufficient information from mass media like urban mothers so convincing them and giving service is difficult for them. The other thing is some of the rural catchments are far apart from the health center and we have to go there and address them. So work burden different in urban and rural area.

I; Do Woreda health office officials like you have restrictions/barriers on the accessibility? What restrictions/barriers? How do the restrictions/barriers influence the access of the PP and PA family planning services and supportive social networks?

R; first challenge for us is leaving the workload for family planning method service providers and MCH focal that means not working in collaboration with other departments and stakeholders, MCH focal by herself can't bring any change it needs networking, collaboration. The other one is the limited number of professional, absence of IUCD set.

I; Do you participate in any meetings, workshops, trainings, pregnant women conference, family, community, government, or other social networks organizations? Please tell me more.

R; yes I participated in pregnant mothers conference were held in Tome Gerera kebele, also participated in workshop held in Halaba city by in gender health, in the pregnant mothers conference we taught the pregnant mothers collected from different Keeble's, after the conference I recognized the change.

I; Please tell me how your participation in these organizations affect the PP and PA family planning services provision

R; it created the opportunity to me to share what I got from the workshop and myself I detected the change on utilization and acceptance among pregnant mothers. I also taught them about the advantage of receiving postpartum family planning and the expected risk of not using it as MCH class. Tried to answer the questions raised from mothers and when they come to health center they can get the same thing that we told them during the conference

I; In your Woreda do women assume leadership roles? What types of roles? Do these roles change by social characteristics, such as (dis)ability, economic status, health status, educational level, religion, culture, geographical location, and marital status?

R; in this health center women assumed in leadership position, MCH focal is women and assuming was based on hardworking work status, client approach

I; Please tell us about your own experience with assuming any leadership roles?

R*; now I MCH focal and my experience is I'm hard worker and approach clients friendly , assuming females especially in MCH class is ideal for clients because most of service utilizers are females so it creates great opportunity to them to share their experience or feeling in detail. The clients are happy by receiving especially delivery service through females. This does not mean males not work appropriately in that place; there are males who counsels and approaches better than females. I think the assumption done for client's satisfaction and comfort. In the professional side I approach them sisterly or brotherly and try to identify skill gap or refusal to activity then arrange time to communicate with service provider health professional to solve the problem.*

I; Please tell me how your leadership roles affect the PP and PA family planning services provision

R; from my behavior I'm easily approachable person so I can share the information for them as friend not as professional and tells them my own experience at that time they are happy to receive postpartum and post abortion service. We can bring the required change if we do all activities in coordination, coordinating them and sharing them goal of the service, identifying gap and giving on job training for those who have skill gap just by approaching them friendly.

I; In your Woreda/community who decides at what age people marry? What are the reasons for getting married at younger or older ages in this Woreda?

R; in this community I think family decides to marry, the reason may be the age, if they educated up to tenth grade and if they don't bring the required grade to pass preparatory and if their parents have no enough income to pay for college or university fee, at that time they say if he is male why don't I get my share from my fathers farm and get marriage, and the girl says I didn't

bring the required grade for college or university why don't I marry and bear children. Those who educated or merchant get marriage when they feel they are able to administer or lead my family by economy

I; Please tell me how their age at marriage affects their PP and PA family planning services use

R; getting marriage early can expose female to related pregnancy complication which is not good for the and also they uses family planning method before they give birth which may cause them to infertility.

I; what are your suggestions to improve women's roles and participation to enhance the use of PP and PA family planning services?

R; assigning females to talk in pregnant mother's conference, bringing females to the members of management, letting mothers who received the service previously to share the idea openly, participating mothers in relate activities

Knowledge, Beliefs, and Perceptions

I; In your Woreda what is appropriate behavior for a woman after delivery and abortion regarding the use of PP and PA family planning? How do they differ from adolescent girls and young women?

R; recently delivered mothers says why don't I take some rest, in this body why I take this burning medication, I do not do sexual intercourse before three or two months so why I use it at this time, people may talks me. They feel that it increases bleeding tendency, causes dizziness, makes me thin

I; How do these believe influence health behavior including the PP and PA family planning use?

R; for that mother they refuse to receive the service.

I; What are the social beliefs and perceptions that share women's expectations and aspiration for health care including PP and PA family planning?

R; if the community member hears that they can say why she receive this is her husband starts to do sexual intercourse before drying of the first blood, prior time we stayed long time without becoming pregnant without using any drug, is she planned to do sex as soon as she go to home?

Is it burns her if she stay without sex? But health professionals make it secrete an no one has access to hear it without her husband

I; In your opinion who should make decisions regarding use of PP and PA family planning services? What types of decisions do women make regarding use of healthcare including PP and PA family planning services?

R; wife and husband both should make decision on the utilization of postpartum or post abortion family planning.sometimes after counselling given they say I will inform you my decision after discussing with my husband, at that time also some of them refuses to receive by saying my husband is not voluntary or others agreed to receive.

I; What types of decisions regarding use of healthcare including PP and PA family planning services are made jointly?

R; most of them agree to receive the service but few of them refused due to their husband concern, as I heard from them they say my husband said please deliver safely first the other thing is coming next, or others says even you don't know the wellbeing of yours and your baby so calm to decide this, safe delivery should be first.

Legal Rights and Status

Now we are going to discuss about the marriage related process prevalent in this community.

I; In your community are women who delivered recently legally allowed to receive PP and PA family planning service?

R; yes it is legally allowed because it doesn't interfere with their delivery status whether they delivered through stich or cesarean section the pain or the discomfort will exist whether she receive the service or not it does not relate with post-delivery discomfort or pain. On the other hand receiving the service is beneficial for them so if it is beneficial it should be allowed legally.

I; Is these same for boys and girls, people from different age groups, education background, ethnic groups, socio economic classes, excluded groups? How are they different? Why/ Why not?

R; yes it is similar for all people

I; How do recently delivered/aborted women access PP and PA family planning information and resource from the healthcare facilities?

R; they can get the resources and information related with post abortion family planning when they come to health center from health professionals trained to provide such services and after getting abortion care they counsel to stay at least six months before becoming pregnant next time so to do so they told to receive family planning methods otherwise another pregnancy can start. Information related with postpartum family planning they can get information during ANC VISIT, pregnant mothers conference and at latent phase of delivery and resources from the health center after delivery

I; Is these same for boys and girls, people from different age groups, education background, ethnic groups, socio economic classes, excluded groups? How are they different? Why/ Why not?

R; I don't think so more of pregnant mothers come to ANC visit and get the information and also only pregnant mothers come to pregnant mothers conference and get the information easily

I; Do the people in your community support recently delivered/aborted women to access PP and PA family planning information and resources? How do they provide support? Who are these people? Why do they provide support?

R; maybe there are community members who received the service first and can share their experience and encourage them to receive, the other one is one to thirty leaders may share the information to recently delivered mothers

I; Are there certain groups in your community who do not support this? Who are these people? Why don't they support?

R; yes there is community members who discourages when women uses post-partum family planning methods, those are who do not use family planning methods and bear children without birth spacing they don't considers the quality rather bothers the number of children, they say our children grown up safely

I; Please tell me women's experience with accessing healthcare information and resources including PP family planning.

R; from health center during ANC, delivery, receiving abortion care and at the community during pregnant mothers conference

I; Do you feel they have a right to get healthcare information and resources including PP family planning services? Why/why not?

R; yes they have the right to get the required information and resources and health professionals have obligation to provide the service appropriately

I; Do you think women's experience is different from other people in your community? How? Please tell me more

R; some women's who do not have ANC follow-up and not participated in pregnant mothers conference can get the information from health center during delivery time, but those who have follow-up can get the information either from conference or ANC visit in addition to during latent phase of delivery

I; Is there legal mechanism you can follow to ensure they get quality healthcare information and resources they need? Please tell me more

R; I can follow first through registration because all things done should be registered so I can check from registration book, at that time if I find any gap I can contact the specific service provider professional and communicate with him to solve it in further.

.

****

Summary

This interview conducted with MCH focal at Tome gerera Health center in 26/27/22. The reaction she had were she was slow responsive. The key finding were the utilization of pp fp as Tome health center is low and affected by misconception, to improve this health professionals approach community members friendly rather than professionally. The decision to receive postpartum family planning should be done between husband and wife. There is awareness creation started from community level to latent stage of delivery. They lack functional IUCD set and adequate professional to sustain the service provision.

**Interview no**	**01**
**Code**	**IDI_ TG _WO_26_7_22**
**sex**	**Female**
**Age**	**18(I'm not sure but it is around 18)**
**Educational stutus**	**10th**
**Occupation**	**House wife**
**No of children**	**1**
**kebele**	**Tome gerera**
**Name of interviewer**	**Befekadu Bekele**

I: Health services (PP and PA family planning services) availability: Can you tell me the types of healthcare facilities that provide PP family planning services in your area?

a. What types of FP services are available at the healthcare facilities?
b. Are these services equally available to boys, girls, young, old, married, unmarried, people from different geographic locations, religious or cultural background, language etc.? Please tell me more

R: you are right. The health care worker told me to start family planning method given via injection after birth and I said I should stay because I suffered a lot during pregnancy period. So I was not happy when he told me to start because I wanted to stay for a month. I know that it is good to use family planning because if mother delivers without adequate time gap "ele ele yelikko", it is difficult to fulfill what the children need. It is good to start family planning immediately after birth.

I: you told me that you wanted to stay for a month when the health care worker advise you to start?right? what other family planning methods do you know?

R: I don't know other methods. I'm new comer and I have no idea on this. I don't know.

I: didn’t you hear about other methods?

R:Depo..this is what I know. Is there any other method?

I:Did the health workers tell you options?type of family planning methods?

R:I heard about the methods when I was accompanying other friends.

I: What did you hear about ?

R:They informed about the method given via injection and they told to my friend not to give birth and they told her to have adequate spacing. If you don’t start to use family planning ,unintended pregnancy may occur. I heard all this while they were counseling my friend.

I: Are these services equally available to boys, girls, young, old, married, unmarried, people from different geographic locations, religious or cultural background, language etc.? Please tell me more?

R: Yes, the health workers counsel and teach the community. There is available for different groups.

I: Can you tell me how far the PP family planning services providing facility is located? How long does it take for you to access the facility?

R: you mean how far the health center is from here?

I:yes

R:it is very close to us. It may take five to 10 minutes.

I: What type of transportation means do you use to reach the facility? How easy or difficult is it to use the transportation services?

R: on foot. Because it is near.

I:Is there any cost associated with accessing the PP family planning services? Please tell me more about this

R:what kind of cost?

I: cost related to family planning

R: There is no any other cost. we pay only for medical card. Everything is free of charge.

I: Is the PP family planning service open at all time and is accessible with short waiting time at the healthcare facility? Are there types of FP methods of users' choice in the healthcare facility?

R: the health center is open at all time. We can get them at any time and they treat us and give what we need as soon as possible

I: Are there types of FP methods of users' choice in the healthcare facility? For example there are different family planning methods ,short acting and long acting ones given For three ,five years. Did the health care works tell you about this?

R:They asked about these things. Gave me options a method given for three years and five years. So I can decide the method I like. Nobody forces us to use rather we can use based on our own choice.

I: Do healthcare providers receive and treat users with respect and dignity?

R: They respect us and give the service as soon as possible. But I haven't started to use any type of family planning so far. There are waiting times sometimes. I have't started to use any of the method.

I: why?is there any barrier no to use?Reasons

R: No, there is no one.It is me who was not willing to start the service ''erayda aggas" I refused it intentionally…loughing. By the time I was not in a good health condition so I wanted to stay some time.

I: was there any other reason?

R:There is no other reason.The reason I refused is because I was not healthy by the time. In addition to this we(husband and wife) did not discuss together on this matter. I don't know what is in his mind by the time.

I: you mean can your husband be obstacle for PPFP?

R: No ,I am not saying like that I am going to use family planning in near future.

I: How easy could you access the PP family planning service? Do you think your access to the PP family planning services is affected because of your background? How?

R: There is no problem with this regards. I can access and get the service any time.

I: Please tell me about your household resources and assets. Please tell me the resources and assets belong to you.

a. How is the use of the household resources and assets decided? Who makes the decision? Do you involve in the decision making? How do you think that affects your access to the healthcare services including PP family planning?
b. Please tell me about your household income. Please tell me about your own income.
c. How is the use of the household income decided? Who makes the decision? Do you involve in the decision making? How do you think that affects your access to the healthcare services including PP and PA family planning?
d. In your community how is use of PP family planning seen? Is it acceptable for women to use PP family planning? Why/why not? Please tell me more
e. In your opinion what should be done to improve access to the PP family planning services for all recently delivered mothers in your community?

R: we don't have any resources, asset except God. We don't have cattles, and other resources. It is only God who provides. we don't have agricultural land for farming. Here We live in a rental house.

I: How do you see family planning and the current living condition?

R: I don't know what to do God only knows. we don't have any source of income we only do the work of God because my husband is serving God.

I: In your community how is use of PP family planning seen? Is it acceptable for women to use PP family planning? Why/why not? Please tell me more

R: I don't know how they see.I don't want to use family planning immediately after birth.I don't want use it, I don't like it. I refused because I didn't want to use it immediately I wanted to stay sometime and come back to use the service. Some people want and like to start to use family planning immediately after birth. Some people who are aged once are not happy and say " *hegee ayba palabe ,kowoy ayba palaba ehide? Yelosaara narppiya ekkidi bo gees* "Ha'ii yelida assaaka

narppyia ekkada ba gees xooso ne ashsha'' which means they are very surprised with immediate post partum family planning.what is the government doing?how a recently delivered women are told to use family planning?''.Other people support using family planning immediately after birth, because of the current living condition, because as the population number increases there would be shortage of foods.So they support PPPF and the government is right they say''.There fore,there are two kinds of people ;the one who support and don't support immediate PPFP services.

I:why do you think people think that way? specially those who don't support?

R:it could be lack of understanding. They could understand better if health care workers teach them.If other lay people tell them ,they don't want to accept.

I:you told us there are people who support.why do they support?

R: because of the living condition., lack of economy. Fear of caring children with such low economy. They encourage immediate PPFP, where as others think that it is not good.

I: In your opinion what should be done to improve access to the PP family planning services for all recently delivered mothers in your community?

R: I want to use the service.I encourage others to use because the current livimg condition is not good. we cannot fulfill what our children need.we cannot afford for their cloths,foods.If there is no spacing, that is not good for the children and children live in a poverty.Therefore it is better to use PPFP.

I: in your opinion what should be done to improve or enhance the services?

R:health care workers should play their role discussing with the woreda officials

I: Please describe to me the typical women's roles in your community?

- How are women and girls occupied over the course of 24 hours?Are there seasonal differences in how women and girls use their time?

What kind of work do you do? Do these works change when your age or status changes (young, unmarried and married, older)?

R: ‘‘ kattays,essays,pittays’’ means.. I prepare food, clean house,buy what we need from market etc.This is what I usually do.

I: Do you havebarriers on your access? What barriers? How do the barriers/restrictions influence your access to the PP family planning services and supportive social networks?

- In your community do the restrictions/barriers change by social characteristic, such as (dis)ability, economic status, health status, educational level, religion, culture, geographical location, and marital status?

R: I refused to take family planning because I suffered a lot during pregnancy. I have passed through painful times. Therefore, I want to use when I become stable. I was sick for long time.

I:what about other barriers?culture,religion?

R: There is no barriers other than my health condition. It was the only reason prevented me from using the service. I am willing to it.

I: Do you participate in any family, community, government, or other social networks organizations? Please tell me more. 1 to 5 or (1 to 30 networks?)

- Please tell me how your participation in these organizations affect your PP family planning services use

R: Yes I used to participate in such meetings. I stopped going because of my bad health condition during pregnancy. There are so many benefits we get by participating because they(health workers),teach us many things. for example they teach us about family planning. They teach us about unity, how to live with others in peaceful way.

I:In your community do women assume leadership roles? What types of roles? Do these roles change by social characteristics, such as (dis)ability, economic status, health status, educational level, religion, culture, geographical location, and marital status?

- Please tell us about your own experience with assuming any leadership roles?
- Please tell me how your leadership roles affect your PP family planning services use

R: I did not play any leadership role because I am new comer. Therefore, my involvement is less.

I: In your community who decides at what age should people marry? What are the reasons for a women getting married at younger or older ages?

- Please tell us about your own marriage experience? How old were you when you marry? What are the reasons you she get married at that age?
- Please tell me how your age at marriage affects your PP family planning services use

R: loughing…people can decide by their own. Females usually decide.

I: What are the reasons for a women getting married at younger or older ages?

R: Xosoo waano…my God…what question is it? loughing..

I: Please tell us about your own marriage experience? How old were you when you marry? What are the reasons you she get married at that age?

R: I decided by my self.

I: How old were you when you marry

R: xooso! My God …I don't know at what age I got married. I got married in 2019.

I: you told me that you are 18.so you mean you got married when you are 16?

R: I'm not sure.

I:what forced you to marry in this age?

R:I decided by myself nothing pushed me to decide.

I: What are your suggestions to improve women's roles and participation to enhance the use of PP family planning services? You told us the disadvantages of unintended pregnancies in our earlier discussion.

R: it is good to start family planning immediately to prevent unintended pregnancy. They should take care of their children. It good that mothers are encouraged to use and is legally allowed Because of these reasons, it is very important thing people should use the service.

I: In your community what is appropriate behavior for recently delivered woman regarding use of PP and PA family planning services? How do they differ from adolescent girls and young women?

What are the social beliefs and perceptions that share women's expectations and aspiration for PP family planning?

R: I have already said it before.There are two group of people. those who want to use the service and others who don't want to use. It differs from person.

I: In your opinion who should make decisions regarding use of PP family planning services? What types of decisions do women make regarding use of healthcare including PP family planning services? What types of decisions regarding use of healthcare including PP family planning services are made jointly?

R: I can decide.

I: Can you decide by yourself?

R: No I can't. We decide together.

I: In your community are women who delivered recently legally allowed to receive PP family planning service?

- Is these same for boys and girls, people from different age groups, education background, ethnic groups, socio economic classes, excluded groups? How are they different? Why/ Why not?

R: I don't think that that it is legally forbidden. That is why health workers ask all recently delivered mothers to use the service. Those who are willing to use get the service and those who resist don't use the service.

I: How do recently delivered women access PP family planning information and resource from the healthcare facilities?

- Is these same for boys and girls, people from different age groups, education background, ethnic groups, socio economic classes, excluded groups? How are they different? Why/ Why not?
- Do the people in your community support recently delivered women to access PP family planning infatuation and resources? How do they provide support? Who are these people? Why do they provide support?
- Are there certain groups in your community who do not support this? Who are these people? Why don't they support?

R: They(women development armies) teach us some times in 1 to 5 network. They counsel us.

I: is there any other source of information?

R:There is no other source of information.

I:Do you feel you have a right to get healthcare information and resources including PP family planning services? Why/why not?

- Do you think your experience is different from other people in your community? How? Please tell me more

R: Yes I have right to get information.

I: Is there legal mechanism you can follow to ensure you get quality healthcare information and resources your need? Please tell me more

R: There is no legal mechanism. I don't know about this.

I: Please tell me more

R:women development armies

I: Thank you for response.

R:Thank you.

Summary

- ✓ She believed that if she uses family planning soon after birth. She believes that it will not be good for her health because she suffered a lot during her pregnancy and if she uses F/P soon after birth it may complicate…fear of perceived side effects
- ✓ Health care workers should address new comers because when they are new for the environment ,they may not know where to go, from where to get information
- ✓ It is better not to miss eligible groups who accompany a recently delivered woman while counseling. This could be ideal time where they can get information b/c this can help them to decide
- ✓ Refusal to use immediate F/P was related with previous history of health condition, perceived side effects
- ✓ It is shocking and very new idea in the community ,when health workers counsel a recently delivered women to use F/P people those who accompany a recently delivered woman will be very surprised.

Interview details

**Interviewee**	Recently deliverd woman
**Age**	25
**Interviewee code**	IDI_WA_WO_240622
**Name of interviewer**	Beimnet Desalegn
**Date**	24/06/22
**Time started**	4:06 lt
**Time finished**	4;43 lt
**Venue**	Wadu Health center

Assets and Resources

Discussion

Now we are going to discuss about the Healthcare resources and assets available to recently delivered mothers in your area.

I: Health services (PP and PA family planning services) availability: Can you tell me the types of healthcare facilities that provide PP family planning services in your area?

a. What types of FP services are available at the healthcare facilities?
b. Are these services equally available to boys, girls, young, old, married, unmarried, people from different geographic locations, religious or cultural background, language etc.? Please tell me more

R: - *yea …eee … our residential area is under this health center most of the time we were not coming here for FP services… I am using from privet health facility near to our residential.*

Most of the time we get depo, and pills … we cannot get contraceptive method which used for 3 or 5 year …. There most of the time we can find depo and pills.

Yeah… still we are using service from the man who is respected one and has God fear … he provides the service for all service user equally… I have not seen discrimination or stigma among the user due to their background … he looks us all equally…

I: - Accessibility of the PP family planning services

c. Can you tell me how far the PP family planning services providing facility is located? How long does it take for you to access the facility?

d. What type of transportation means do you use to reach the facility? How easy or difficult is it to use the transportation services?
e. Is there any cost associated with accessing the PP family planning services? Please tell me more about this
f. Is the PP family planning service open at all time and is accessible with short waiting time at the healthcare facility? Are there types of FP methods of users' choice in the healthcare facility? Do healthcare providers receive and treat users with respect and dignity?
g. Are these services equally accessible to boys, girls, young, old, married, unmarried, people from different geographic locations, religious or cultural background, language etc.? Please tell me more
h. How easy could you access the PP family planning service? Do you think your access to the PP family planning services is affected because of your background? How?

R: - I used because this privet clinic is near to our residential area. Even this health center is not far for us … I had pregnancy follow up here … and I completed my pregnant follow up here … I walk or use Bajaj to come here, its cost is about 10 birrs.

Yeah, often I use Bajaj to come to here … it costs 10 birrs in one trip.

Okay… as I told you before, we pay for family planning when we take from privete clinic… but here at health center family planning is free of charge including delivery service, as I heard from other users. I have not used family planning from this health center, I was using from a privete clinic... because of a privet clinic is near to my house I was using from there. … yeah, I go with my feet.

Yeah, I can get any service at any time including family planning but only depo and pills...

I give birth at Ottona hospital, after wadu health center referred me for better care… there, after I have giving birth doctors' at the hospital told me to use contraceptive … as I was suffered with prolonged labour pain and give birth by CS … they told me to take a break and delay birth for more than two year … and they give Implanon after delivery. Now I am Implanon user.

I: - Means to access the PP family planning services

i. Please tell me about your household resources and assets. Please tell me the resources and assets belong to you.

j. How is the use of the household resources and assets decided? Who makes the decision? Do you involve in the decision making? How do you think that affects your access to the healthcare services including PP family planning?
k. Please tell me about your household income. Please tell me about your own income.
l. How is the use of the household income decided? Who makes the decision? Do you involve in the decision making? How do you think that affects your access to the healthcare services including PP and PA family planning?
m. In your community how is use of PP family planning seen? Is it acceptable for women to use PP family planning? Why/why not? Please tell me more

***R: -** I am merchant … yeah, my husband also works with me. Before we were living in the rental house but now we built our own house and living there. Yeah, we have coffee house … when we get time we do our business by ourselves … when we are busy we have employed workers to help us.*

Yeah, first we have peace in God and in the case when we get a problem or when one of us gets sick we discuss and use money from our deposit in the bank. We discuss to use the money we have.… we have common bank account. We can't withdrawal money lonely … Yeah, sometimes this may have an effect if we have emergency condition, so to manage this we have money at hand to use in such case…

I: - In your opinion what should be done to improve access to the PP family planning services for all recently delivered mothers in your community?

***R: -** as you know giving birth frequently may harm mother … and now as we hear there is shortage of short acting family planning method which is most reparable one by the user… because of this many mother is exposed to unplanned pregnancy. So, this short acting family planning should be available to users … because some of the women has fear of using long acting contraceptives methods. There are many mothers who are unwilling to use long acting contraceptive methods … the government should have to avail, contraceptive methods which is preferable by user at health facility.*

Practices, Roles, and Participation

Now we are going to discuss about your women's and your role and responsibilities.

I: - Please describe to me the typical women's roles in your community?

- How are women and girls occupied over the course of 24 hours? Are there seasonal differences in how women and girls use their time?

***R:** - most of the time women in our community are involved in any activity which helps them to support their family economically … no one is set waiting for their husband … they try their best to support the family financially. you can't get anyone at home if you go to their home. They go to the city and work for …*

Yea, there is a session or existing condition that has an effect on their activity. … if you take me, before I got pregnant and gave birth I was working my business by myself but after giving birth, my concern totally shifted to my baby and now I stay at home giving care to my son.

I: - What kind of work do you do? Do these works change when your age or status changes (young, unmarried and married, older)?

R: - *yeah, I expect that when we get old and give more birth our activity will be limed due to different reason and we prepare for a job or engage in activities which fits our ability at that time.*

I: - Do you have barriers on your access? What barriers? How do the barriers/restrictions influence your access to the PP family planning services and supportive social networks?

- In your community do the restrictions/barriers change by social characteristic, such as (dis)ability, economic status, health status, educational level, religion, culture, geographical location, and marital status?

R: - *I don't have any restrictions… as I am using the contraceptive method now.*

I: - Do you participate in any family, community, government, or other social networks organizations? Please tell me more.

- Please tell me how your participation in these organizations affect your PP family planning services use

R: - *yeah, I was participating in different meetings with health extension workers when they invite me at any time. I don't have more than this participation…*

I have learned many things from my participation … they taught us the consequence of frequent birth, and how we space our birth … It helped me to know more about the contraceptive method. Now if you see our community no one is giving birth over and over again … many mothers are using contraceptive methods and spacing their birth. That is due to what we got from health workers in our community. I have seen many heartbreaks on mothers before when I was a child because of having birth every year without a gap…

I: - In your community do women assume leadership roles? What types of roles? Do these roles change by social characteristics, such as (dis)ability, economic status, health status, educational level, religion, culture, geographical location, and marital status?

- Please tell us about your own experience with assuming any leadership roles?
- Please tell me how your leadership roles affect your PP family planning services use

R: - *yes, many women are takings a headship position now, … eee women are represented or invited in any community activity … here in our community, no one undermines women's role.*

Eee… I myself did not assume any leadership role …

I: - In your community who decides at what age should people marry? What are the reasons for a woman getting married at younger or older ages?

- Please tell us about your own marriage experience? How old were you when you marry? What are the reasons you she gets married at that age?
- Please tell me how your age at marriage affects your PP family planning services use

R: *hahaha… some marry late and some at early age… it's the woman's decision. Usually, they marry by 14 years. … I don't know the exact reason to marry late or early … most of the time it has a personal reason for each, I don't know about the other… there might be stress, and don't want to continue their school, … most of the time they do not obey their family now.*

I was 17 when I got married… haha the reason was I failed the university entrance exam and I was alone when my friends join university … then I get stressed and decided to get married.

I: - What are your suggestions to improve women's roles and participation to enhance the use of PP family planning services?

R: - *first, the concerned body should have to create awareness about the issue of post-partem family planning among the women and have to train them to empower them.*

Knowledge, Beliefs, and Perceptions

Now we are going to discuss about the issues faced by adolescents and women in accessing PP and PA FP services in your opinion.

I: - In your community what is appropriate behavior for recently delivered woman regarding use of PP and PA family planning services? How do they differ from adolescent girls and young women?

a. What is the appropriate behavior for a recently delivered mother? How do these believe influence health behavior including the PP family planning use?

R: - *some of the community members specifically educated husbands look at it as an appropriate decision to use contraceptives… but some husbands see it as inappropriate behavior, they think that it may affect their later pregnancy or cause infertility for women. This perception also is true for women too… they also think that using contraception immediately after birth has harm for them.*

Using contraceptives is important for women … if they understood what happens to them if she didn't space and control their pregnancy, they women use contraceptives. In my understanding

women can get pregnant by stopping or by removing contraceptives when they want to have a pregnancy.

I: - What are the social beliefs and perceptions that share women's expectations and aspiration for PP family planning?

R: - *as I told you before, here in our community, most of the time husbands want to have many children… community sees children as wealth, which may influence women's beliefs and expectations of controlling their pregnancy.*

I: - In your opinion who should make decisions regarding use of PP family planning services? What types of decisions do women make regarding use of healthcare including PP family planning services? What types of decisions regarding use of healthcare including PP family planning services are made jointly?

R: - *in my opinion, it should be decided together with the husband. In every discussion partner involvement is important, I know many families whose relationships were broken due to this contraceptive case.*

<u>Legal Rights and Status</u>

Now we are going to discuss about the marriage related process prevalent in this community.

R: - In your community are women who delivered recently legally allowed to receive PP family planning service?

- Is these same for boys and girls, people from different age groups, education background, ethnic groups, socio economic classes, excluded groups? How are they different? Why/ Why not?

***R: -** legally, yeah it is allowed to use contraception after having birth … I think that is the reason for health facilities provide it for us after having birth. Using contraceptives is important for me to space pregnancy or control fertility… it gives me rest and keeps my health.*

There is no excluded group as I see here … everybody who wants a service can use it without any discrimination due to their background.

I: - How do recently delivered women access PP family planning information and resource from the healthcare facilities?

- Is these same for boys and girls, people from different age groups, education background, ethnic groups, socio economic classes, excluded groups? How are they different? Why/ Why not?
- Do the people in your community support recently delivered women to access PP family planning infatuation and resources? How do they provide support? Who are these people? Why do they provide support?
- Are there certain groups in your community who do not support this? Who are these people? Why don't they support?

***R: -** most of the time I got information from the radio… health extension, health workers. I hear from Fana radio or Wogeta radio…*

Nobody is there against the use of contraception by women … hiddenly, few husbands prevent their wives from using contraception.

Also, there are women's development army and 1 to 5 teams that support women in the health-related issue and to use of family planning by team members. They support women by providing information, and support in case of any problem they face …

I: - Please tell me your experience with accessing healthcare information and resources including PP family planning.

- Do you feel you have a right to get healthcare information and resources including PP family planning services? Why/why not?

- Do you think your experience is different from other people in your community? How? Please tell me more
- Is there legal mechanism you can follow to ensure you get quality healthcare information and resources your need? Please tell me more

R: *- yeah, when we come to health facilities we have full right to get information regarding our health as well as about the drug they give to us. … that is our right to ask and get information. Also, health workers are obligated to give information to their clients.*

In our community, no one has the fear to ask for information about their health conditions and information about the drug given to them by health workers.

My experience in using contraceptives is … before I was used depo for a long time … and when I got pregnant my blood pressure raised as I heard that is due to using depo for a long time. Then after having the birth I decided to use Implanon after consulting health workers. And now I am using Implanon. I am thankful now for health workers giving this to me. From my experience, I advise using Implanon for other women who want to use contraception. Since I started to use Implanon I have not got weight gain … which was while I used depo and I am now fine and well.

Most of the time we got information from Tv or radio but it is not trustable as they are human, they pass lie information… we use as the source of information but we didn't trust them … we didn't take as they said.

With this we have come to an end of the discussion. Do you want to add anything before we end the discussion or if do you have any questions you want to ask?

Thank you for your time and in case you want, you can get in touch with us through the details mentioned in the consent form.

****

Summary:

The interview was conducted on 24/07/22 with a recently delivered mother at Wadu health center. She was come to vaccinate her child. The discussion was interesting but she did not explain things associated with the FP experience very well. She does not have a history of using a contraceptive method. She has had a history of spontaneous abortions previously. But she did not use contraceptive methods before or after the abortion. she got pregnant again after three months after the occurrence of the abortion.

In-Depth Interview conducted with recently delivered Mother Husband at Wondo Genet Health center

Sociodemographic information of interviewee

Code	Keble	age	Sex	Educational-status	Job-status	Number of children
IDI_WGHC_H_290622	Wondo Genet HC	35	M	secondary	Farmer	five

Assets and Resources

Discussion

Now we are going to discuss about the Healthcare resources and assets available to recently delivered mothers in your area.

1. Health services (PP and PA family planning services) availability: Can you tell me the types of healthcare facilities that provide PP family planning services in your area?

   R/ those services given Health post health center

I; What types of FP services are available at the healthcare facilities?

R; what I know they teach family planning , due to the lack of economy they health workers teach not deliver many children, they insert on for women upper arm, injection in three months interval, they teach family planning methods what I know this. I don't know other methods,

I; Are these services equally available to boys, girls, young, old, married, unmarried, people from different geographic locations, religious or cultural background, language etc.? Please tell me more

R; I think they give it, they give services but the community don't practice what they teach due the problem of economy, they teach but people don't practice what they learn.

2. Accessibility of the PP family planning services

I; Can you tell me how far the PP family planning services providing facility is located? How long does it take for you to access the facility?

R; It doesn't not take more than five minutes, people can use motor bycle

I; What type of transportation means do you use to reach the facility? How easy or difficult is it to use the transportation services?

R; Community can walk to the facilities, some can use motor bycle

I; Is there any cost associated with accessing the PP family planning services? Please tell me more about this

I; Services provided freely without payment for services.

I; Is the PP family planning service open at all time and is accessible with short waiting time at the healthcare facility? Are there types of FP methods of users' choice in the healthcare facility? Do healthcare providers receive and treat users with respect and dignity?

I; Regarding this health workers treat and give services and teach but the economic proble with eating , food shortage women didn't use as needed. Women didn't willing to receive the services but health workers give the services. Health workers give the services in respectful ways

I; Are these services equally accessible to boys, girls, young, old, married, unmarried, people from different geographic locations, religious or cultural background, language etc.? Please tell me more

I; they give for all, don't favor for some, they are good in delivery services for all. They went to the community and teach and tell for the community to use drugs. This family planning method not well match with women body and health, it affects the health of women combined with lack of food for eating,. Women perceive injecting for family planning might harm the health and not willing to receive the family planning services. But the health workers are willing to teach and provide the family planning services. Women fell the family planning injection make burning sensation in body and dizziness in heading. Therefore the didn't want to receive the family planning Family planning drugs affect the health of women, there were many women felt sick and visit health facilities for treatment two to three times. It affects many women health.it related the living condition of women and affects their health.

I; How easy could you access the PP family planning service? Do you think your access to the PP family planning services is affected because of your background? How?

R; there is no favor in the women background, health workers treat all equally and they working well

3. Means to access the PP family planning services

I; Please tell me about your household resources and assets. Please tell me the resources and assets belong to you.

R; For me, I have livestock I have cassava farming, house. I have livestock, house, farming, and children. Thus all shared with my wife, after our marriage and became one all my assets and resources shared together with my wife. There is no difference in our assets, it is shared with my wife.

I; how is the use of the household resources and assets decided? Who makes the decision? Do you involve in the decision-making? How do you think that affects your access to the healthcare services including PP family planning?

R: *we discuss together with my wife, my elder son, if we need to sell the assets we discuss together. I didn't only myself to decide, we need to agree each other*.

I; Please tell me about your household income. Please tell me about your own income.

R; we have income from farming land, the cow's milk and butter; we have no other sources income. For men we can generate income from farming the land, for women if they cows give milk and butter, selling butter and milk. No other income sources could have. Other than farming, do not have income sources

I; How is the use of the household income decided? Who makes the decision? Do you involve in the decision-making? How do you think that affects your access to the healthcare services including PP and PA family planning?

R; deciding together is very helpful; we agree to gather makes peace in our house hold.

I; In your community how is use of PP family planning seen? Is it acceptable for women to use PP family planning? Why/why not? Please tell me more

R; We used family planning for birth spacing, my wife used three months injection, others take three months injection, my wife used three years family planning method unit the child became three year, we don't practice sexual intercourse before not taking family planning methods, we discuss with an issue. .

I; In your opinion what should be done to improve access to the PP family planning services for all recently delivered mothers in your community?

R; this injection affects the women health, I could suggest the government arrange injection which is not affecting women health. In the community around us there is food shortage, those women lack of enough food affected by using family planning injection, might be die using the injection, therefore I could suggest government distribute the food like those malinurited take, with balanced food content providing with family planning injection. Receiving family planning injection is good for family planning but affects the women health, therefore government should avail food delivery. In my perception I think family planning good, for those women take family planning like injection, inserted with upper arm should get enough food and distributing for women with food enough nutrient recommended.

There is a practice of using desert honey, which is so testy people use with soil attached with because it is too tasty. As this even family planning injection affects the women health, they could receive it delivered with other foods items.

Practices, Roles, and Participation

1. Please describe to me the typical women's roles in your community?

   R; in home women prepare food, nurture their children, collect grass for cattle, they involve social activities like eddir, burial ceremony, they interact with their society

I; How are women and girls occupied over the course of 24 hours? Are there seasonal differences in how women and girls use their time?

R; I don't have girl child, I have boys children, girl children after their school they help mothers by collecting grass for cattle. There is no seasonal variation of work, no additional burden of work in seasonal variation

I; What kind of work do you do? Do these works change when your age or status changes (young, unmarried and married, older)?

R; I am a grade ten student, before going to class I work on my farm, farming and digging my farm. I have also no work burden.

I; Do you have barriers on your access? What barriers? How do the barriers/restrictions influence your access to the PP family planning services and supportive social networks?

R*; there is no burden in my household, until now we are taking family planning.*

I; Do you participate in any family, community, government, or other social networks organizations? Please tell me more.

R; yes I participate in Edir, Equb, churches and any social networks my wife too.

I; Please tell me how your participation in these organizations affect your PP family planning services use

R; it doesn't affected me and my family from using family planning methods

I; In your community do women assume leadership roles? What types of roles? Do these roles change by social characteristics, such as (dis)ability, economic status, health status, educational level, religion, culture, geographical location, and marital status?

R: yes , some are used to work, they work as typist, those women came leadership due to their education level, those educated people have wide mentality, think widely compared with not educated.

I; Please tell us about your own experience with assuming any leadership roles?

R; I have practiced leadership in my local church, I go to teach other as gospel evangelist.

I; Please tell me how your leadership roles affect your PP family planning services use

R: there was no negative effect due to my leadership, we use family planning for my family, you know those women didn't use family planning give birth many children without spacing. My leadership experience helped me use family planning and birth spacing. When you give birth immediately without child spacing; a community don't respect you. Birth Spacing; helped me to get respect from the community and useful. Health workers came to the community and teach about birth spacing. People hear them.

I; In your community who decides at what age should people marry? What are the reasons for a women getting married at younger or older ages?

R; some marry by themselves, they didn't hear community; people advise those individuals before marriage they have get educated and change their economic status but they didn't hear and marry before the marriage age. Also some want to marry but didn't get married and stay long years without marriage.

Early marriage considered as years of 12 or 15 years age considered as early marriage. Some marry and age of 12 years. Some marry at age of 15 years and others might not even marry in their life that might be the will of God. 15 years age considered as early marriage. Community doesn't determine the age to marry. Grils by their own decide to marry.

For men to marry they Base on economy and also girls consider the income of the man to be married.

I; Please tell us about your own marriage experience? How old were you when you marry? What are the reasons you she get married at that age?

R; My father died when I was child, due to lack of other support, I decided to marry a wife which can support me. I got 26 years age, the reason I has got married due the death of my father.

I; Please tell me how your age at marriage affects your PP family planning services use

R: we have health facility near and we were using family planning helped us.

I; What are your suggestions to improve women's roles and participation to enhance the use of PP family planning services?

R: I would suggest gathering community together and other health workers came and teach us, there is a local saying " Local ladies don't respect a local gay" you know when those near to community teach community might not give full attention for the training and accept fp. It would be better those from other area came teach community could accept the family planning. Teaching both women and men would be suggested, if you teach only women it could not work well. It needs teaching both men and women they could accept the training and it also helps.

Knowledge, Beliefs, and Perceptions.

I; In your community what is appropriate behavior for recently delivered woman regarding use of PP and PA family planning services? How do they differ from adolescent girls and young women?

R; as I said previously, it needs education, as I mentioned before there is mentally poor people that didn't want to hear about the family planning, those educated people accept the family planning and bring change and others could be trained can be changed for the future. If there is training woman can accept the family planning, I would suggest providing training

I; What is the appropriate behavior for a recently delivered mother? How do these believe influence health behavior including the PP family planning use?

R; recently delivered mother is in pain and uncomfortable to to use family planning method immediately she may want to stay for two or three months so they can refuse to use

I; What are the social beliefs and perceptions that share women's expectations and aspiration for PP family planning?

R; *they may say is she think to do sexual intercourse within this period? Not to be said like this they do not disclose to others about what they received the service, they keep it as secrete. Most of our community is mentally poor and not thinking widely*

I; In your opinion, who should make decisions regarding use of PP family planning services? What types of decisions do women make regarding use of healthcare including PP family planning services? What types of decisions regarding use of healthcare including PP family planning services are made jointly?

R; Both woman and man should make a decision to make the use of family planning, in a family woman might want to give birth and at same time man not want to give child, in the other hand

man want to have child and but woman cannot want. Therefore both should agree appropriate time for them to have family planning. They should both agree and decide.

Legal Rights and Status

I; In your community are women who delivered recently legally allowed to receive PP family planning service?

R; I did not hear and practices, I do not want to take family planning for who delivered recently by surgery. I would suggest the provision of immediate family planning after the recovery of woman with surgery. I did not learn it, but think that the person provide this services have more knowledge and practice and understand, therefore, I think it might not be illegal practices.

I; How do recently delivered women access PP family planning information and resource from the healthcare facilities?

R: firstly this health facility teach woman before delivering birth. Health workers teach about family planning during home to home visit. Those get education receive FP injection.

When health workers before providing implanting in upper arm provide information for a woman.

In surrounding community discourage who deliver a child without spacing, they say is she not taking a family planning. Community encourages the use of family planning till the first child became matured. Among community member with mentally matured educate and give the information of family planning. In the community there are different people groups, those educated, mentally matured, uneducated mentally poor and even those didn't attend to formal

education but by nature who thick broadly who advise and counsel the community members about Family planning.

- Is these same for boys and girls, people from different age groups, education background, ethnic groups, socio economic classes, excluded groups? How are they different? Why/ Why not?

I;Do the people in your community support recently delivered women to access PP family planning infatuation and resources? How do they provide support? Who are these people? Why do they provide support?

R; yes women selected as HDA among others supports and encourages women to receive the service

I; Are there certain groups in your community who do not support this? Who are these people? Why don't they support?

R; those women who are mentally poor can discourage users from using of it

1. Please tell me your experience with accessing healthcare information and resources including PP family planning.

R; from my formal education, teaching from health workers , health care information

I; Do you feel you have a right to get healthcare information and resources including PP family planning services? Why/why not?

R, I do feel that I have right, you known government give equal right for all. I think I feel I have right to get health care information. I feel I have right to get health care information and teach my community for further, I am broadly thinking.

- Do you think your experience is different from other people in your community? How? Please tell me more

R;teaching of family planning is not new for the community, community get information from health workers, get from raio, FM raido, those who get training teach others.

I; Is there legal mechanism you can follow to ensure you get quality healthcare information and resources your need? Please tell me more

R; I can get health information, even this facility has problem with attitude, I can get from facilities and can get a service from hospitals. If they health workers didn't give quality services, I can ask those above the facility who supervise the facility.

.

****

Summary

An interview conducted with recently delivered mother husband at Wondogenet health center in 29/06/22. He was farmer and learned up to grade 10. Able to understand and clarify the questions accordingly. He was happy during interview time.

Key findings: the decision should be better made between the father and mother to receive the service. It is better if well nutrient contained food given with Family planning methods to reduce its side effects. Those mentally poor mothers discourage mother who receive family planning, method at delivery spot or immediately after delivery and they think it as negative thing and misconception on it. The interviewee not support taking family planning method immediately after delivery because they are in pain and it should be given after they get relief.

**Interviewee code**	**IDI_WG_MF_28_7_2022**
**Sex**	**Male**
**Age**	**39**
**Service year**	**10**
**Job title**	**MCH focal person**
**Name of interviewer**	**Befekadu Bekele**
**Date**	**28/7/2022**
**Time started**	**5:00**
**Time finished**	**5:59**
**Venue**	**Wondogenet Health center**

Now we are going to discuss about the Healthcare resources and assets available for provision of PP and PA family planning services in your area.

I: Health services (PP and PA family planning services) availability: Can you tell me the types of healthcare facilities that provide PP and PA family planning services in your area?

a. What types of FP services are available at the healthcare facilities?

R: wondogenet health center is one of remotest health center in the woreda. Even though the community has less awareness about immediate post partum family planning and post abortion family planning services, the service is given in our health center. We are doing our best to give better service for future by organizing staff members and arranging field works, by mobilizing women conferences.

I: What types of FP services are available at the healthcare facilities?

R:Most of the time mothers use next implanol,pills and some students use emergency pills.As we know most of them are farmers or lead their life by farming they don't prefer IUCD.They use implanol ,jeddle. Even though we have tried to create awareness on IUCD the number of using this method are very few.I was in sick leave for the last 15 days and when I came back I have communicated with the head of the health center to conduct women conferences in one of kebele called " bosa wanche " which is densely populated kebele. We are also planning to conduct similar conferences in other kebele.

I: Are these services equally available to boys, girls, young, old, married, unmarried, people from different geographic locations, religious or cultural background, language etc.? Please tell me more

R: unmarried ones are afraid to use family planning because it is taboo to get pregnant without formal marriage.They don't come to health center to use family planning after post partum and post abortion rather they go to hospitals. Therefore, the service is not fully addressing adolescents.

Do you mean by the drugs or F/P methods given ?

I:yes it could be related to drugs family planning methods. Are the services equally available for boys,girls, married, unmarried?

R: There are times where we couldn't get the service for all groups. There are very few mothers who use immediate post partum family planning. For example mothers who delivered through CS/caesarian section, those who want to stay until next pregnancy want to use immediate post partum family planning. There are other women who don't want to use immediately after birth due to lack of awareness. We have been educating people but it needs time to change their behavior in order to use the service. For example, we use to teach them about IUCD but there are women who complain and they tell us that they don't feel comfort " *we don't want to see it…every time after urine…we can also be exposed to "mich''*.That is what they report to us as we teach them about IUCD. We have tried to change such misperceptions but still they are challenging us. However, we are working to change their behavior

I: Can you tell me how far the PP and PA family planning services providing facilities are located? How long does it take for women to access the facilities?

-What type of transportation means do they use to reach the facilities? How easy or difficult is it to use the transportation services?

-Is there any cost associated with accessing the PP and PA family planning services? Please tell me more about this

-How easy could they access the PP and PA family planning services? Do you think that their access to the PP and PA family planning services are affected because of their background? How?

R: Actually are nine catchments kebele addressed by our health center. There were bottlenecks/barriers for the service provision. For example, kebele's like Ogodama, Bosa wanche are very far from the health center. The health center is not found the center so it is not accessible. It is very far. Not only about the distance, if you take Bosa wanche it is near to Hobicha district. If we want to take their report we have to move there on foot. Home delivery free services, ANC 4 and other activities. we don't have any transportation facility. Therefore this could be one of the challenges. It is good that you came here on winter season. If you come here on summer season the rivers overflow and no one can pass the roads, it is impossible to use car. Sometimes we refer mothers to hospitals but they cannot go there due to overflow of rivers. Therefore, they are supposed to wait until the flow reduces. Therefore it is very difficult for mothers to access the service because of rivers. There are challenges related to infrastructures, road, water, electricity related problems.

I: How long does it take for women to access the facilities?

R: There are people who travel four to five hours until they reach the health center. The topography is not conducive. They use motor bicycle to come here. There are hard to reach areas. Therefore, there are cost associated with the service. There is transportation cost. They are supposed to get the services from their locality but they come here to get the service. They are suffering a lot due to overflow of rivers road and electricity problems. We used to use solar energy to get electricity but it doesn't work in the summer season. This has to be solved soon in order give a better service.

I: Is the PP and PA family planning service is accessible at all time at the healthcare facilities? Are there types of FP methods of users' choice in the healthcare facilities? For example during weekends,night times,holidays?

-Are these services equally accessible to boys, girls, young, old, married, unmarried, people from different geographic locations, religious or cultural background, language etc.? Please tell me more

R: As I have said earlier the challenge is related to infrastructures. Other than this, the health care workers are always available and standby to give the services. There is no way where mothers go away without getting service due to absence of health workers because that is their main responsibility.

We give the services based on their choice. We give them the options and tell detail about the methods, benefits and side effects. We don't give the methods without their consent. We don't force them to use. We encourage them to use long acting family planning methods.

I: why do you encourage them to use long acting family planning methods?

R:It is national agendum. That is why we encourage mothers to use long acting family planning methods. They use next implanon, Jeddle.Very few mothers use IUCD. There is gap with IUCD services.

I: Do you think that their access to the PP and PA family planning services are affected because of their background? How?

R: yes. Therefore, we are trying our best and communicating with the health extension services to give the service in the health post.

I: Please tell me about your facilities' resources and assets. Please tell me the resources and assets belong to these health facilities to provide PP and PA FP service.

-How is the use of the facility/Woreda health office resources and assets decided? Who makes the decision? Do you involve in the decision making? How do you think that affects access to the healthcare services including PP and PA family planning by women?

-Please tell me about income of in this Woreda. Please tell me about your woredas income.

-How is the use of the woreda's income decided? Who makes the decision? Do you involve in the decision making? How do you think that affects their access to the healthcare services including PP and PA family planning?

-In your woreda how is use of PP and PA family planning seen? Is it acceptable for women to use PP and PA family planning? Why/why not? Please tell me more (what about boys and girls)

R: Almost all resources used for family planning service are available in the health center. The drugs and equipments are available in the health center. Some equipments may be out of use, for example forceps, scissors. :What do you mean by assets found in the health center ?

I:Availability of room setups, other materials exist permanently

R:There is lack of rooms. When I compare with other places. I used to work I other places before I come here. In other places there were dormitory for nurses and all necessary equipments needed are available in former working places. There is family planning store in other places but this is not yet fulfilled in our health center. The place is not conducive to put the drugs. There is also lack of beds. I used to raise this in woreda meetings, management meetings. There are times where three or four mothers come at a time. During this time we face shortages of beds. There is still gap with this regards.

I: Who makes the decision? Do you involve in the decision making? How do you think that affects access to the healthcare services including PP and PA family planning by women?

R: The woreda is decision maker. The health center is governed under woreda health office. The health center is autonomous. So far, the procurements have been done by the woreda health office. We haven't been involved in any decision making process. Regarding the income I have no full information. The finance bodies know more about it. There is community based health insurance scheme and majority of the beneficiaries are people who are beneficiaries enrolled under safety net program. Majority of the community are part of community based health insurance. The income is not adequate in my opinion. I cannot exactly say this is annual income. Although we don't have enough information about total budget and income, we discuss together before procurements of necessary drugs. We plan before procurement and evaluate how much we used from the allocated budget and we discuss on how much budget is needed. Financial bodies and head of the health center know more about this

I: In your woreda how is use of PP and PA family planning seen? Is it acceptable for women to use PP and PA family planning? Why/why not? Please tell me more (what about boys and girls)

R:There are mothers counseled by physicians. Specially those who delivered by caesarean section are not allowed to give birth at health center ,they are referred to hospital because she may be in need of oxygen.

I: In your woreda how is use of PP and PA family planning seen? Is it acceptable for women to use PP and PA family planning? Why/why not? Please tell me more? As you know this is new approach previously it was given for mothers after forty five days.

R: The community had no awareness about the advantage of partum family planning services. Recently their behavior is being changed due to efforts taken so far. Now we live in digital world and the health workers are doing their best. Educated once mostly accept immediate post partum family planning. Uneducated people have also started to use the service. As per male family planning we put condom in condom box for males. Although there is miss use of condom. Previously we put condom box at the gate of health center but now it is in MCH room.

I: what about immediate post abortion family planning services?how is it seen?

R:they are willing to use family planning after post abortion.

I: In your opinion what should be done to improve access to the PP and PA family planning services for all women in your community? What should be done to improve the services for Boys and girls?

R: Intervention should be done at the community or grass root level. This is the key issue.

I:what kind of activities to be done? At community level ?how?

R: it is better to give awareness for the community about unintended pregnancy, teaching them about the benefits of immediate post partum family planning. This could improve accessibility.

I: Please describe to me the typical Woreda health office officials/experts roles in your Woreda/community?

a. How is Woreda health office officials occupied over the course of the day? Are there seasonal differences (campaign/political assignment) in how you use time?

2. As Woreda health office official, what kind of work do you do? Do these works different when facility status changes (Rural, urban and geographical locations)?

R: I am engaged in delivery services, ANC services. I participate in all MCH services. we routinely give the services even though there are extra activities like campaign because mothers will be at risk of getting different problems unless we give these services routinely. The health care workers are stand by at any time.MCH activity like delivery is emergency so health workers are available at any time.

I: Do Woreda health office officials like you have restrictions/barriers on the accessibility? What restrictions/barriers? How do the restrictions/barriers influence the access ofthe PP and PA family planning services and supportive social networks?

a. In your Woreda do the restriction/barriers change by social characteristic, such as (dis)ability, economic status, health status, educational level, religion, culture, geographical location, and maritalstatus?

R: would you stop recording? Let me say something. There are times where we boldly raise and discuss about the gaps in the health center. We used to mention all gaps related to the health center. However, it is given another meaning by the politicians. The politician don't like if you mention the existing problems and barriers for the services because they want you to mention about success only and appreciate even though there are many gaps to be filled. They don't let us speak on the weak sides.

I:As a health care worker would you tell us what is on ground. would you tell be about the barriers on the accessibility. barriers influence the access of the PP and PA family planning services and supportive social networks?you tried to mention wrong political interferences. what else? barriers related to culture, religion?

R: There was cultural influences that hinder the provision of health services but now this has been reduced due to improved health literacy of the community. There were gaps in post abortion family planning services because the community had no awareness about it. But now

,due to different interventions taken so far, the community awareness is being improved and they are free from cultural influences because of the influence of sciences, technology.

I: Do you participate in any meetings, workshops, trainings, pregnant women conference, family, community, government, or other social networks organizations? Please tell me more.

-Please tell me how your participation in these organizations affect the PP and PA family planning services provision

R: Yes I have participated in such meetings before I came here, that is when I was in Somalia. since then I haven't participated in different trainings, meetings. However, I am in engaged in creating community awareness. we help them accept the service by teaching the benefit of using immediate post partum family planning. Therefore, they started to use the service and we are working hard to improve the service. We should do more than this for future in order to increase the uptake of the service.

I:In your Woreda do women assume leadership roles? What types of roles? Do these roles change by social characteristics, such as (dis)ability, economic status, health status, educational level, religion, culture, geographical location, and maritalstatus?

-Please tell us about your own experience with assuming any leadership roles?

-Please tell me how your leadership roles affect the PP and PA family planning services provision

R: We created opportunity for women to play leadership role within the health center. Because males and females are equal. Therefore we used to give opportunity to lead the health center. Still there are women participating in leadership positions, management bodies, and we can do better than this for future.

I:In your Woreda/community who decides at what age people marry? What are the reasons for getting married at younger or older ages in this Woreda?

R:Actually girls face different problems when they marry at their earlier age. For example they are exposed to fistula. It is better if they marry at the age of seventeen and above. Unless they are at risk of getting different health problems. Girls don't listen to our counseling, they decide by themselves when they are about to marry. We have been educating girls to marry at the right age. But early marriages is still practiced in some places. Because boys and girls don't know what is good and what is not since they are in fire age. There are unintended pregnancies. This things happen due to early marriage.

I: Please tell me how their age at marriage affects their PP and PA family planning services use

R:There are emergency contraceptives. If they know about the service ,they could have used emergency contraceptives. However, they hide themselves and don't come to health center. They come after pregnancy occur. It is obvious that if they get marry pregnancy comes next. When they get marry at early age they will face different problems during delivery. Because they don't know real life challenges after they give birth. Their uterus cannot carry fetus when they marry at early stage. Adolescents don't know all these challenges before they marry.

Knowledge, Beliefs, and Perceptions

Now we are going to discuss about the issues faced by women in accessing PP and PA family Planning services in your opinion.

I: In your Woreda what is appropriate behavior for a woman after delivery and abortion regarding the use of PP and PA family planning? How do they differ from adolescent girls and young women?

How do these believe influence health behavior including the PP and PA family planning use? What are the social beliefs and perceptions that share women's expectations and aspiration for health care including PP and PA family planning?

R: Because we teach the community and tell them facts, they are willing to accept our advises. They trust health workers. Sometimes we counsel husbands and recently delivered women together, we tell husbands all the risks that their wives face if they don't use immediate post

partum family planning. Husbands are willing to accept our advices. Mothers, mother in laws also encourage their recently delivered daughter to use family planning immediately after birth.

I: In your opinion who should make decisions regarding use of PP and PA family planning services? What types of decisions do women make regarding use of healthcare including PP and PA family planning services? What types of decisions regarding use of healthcare including PP and PA family planning services are made jointly?

R: The woman should decide by herself. We don't force her to use the family planning method. our role is just educating them to use. As they get counseling they will be in a position to decide. However, if a woman decides by herself her husband may not be willing to accept her decision. Therefore we counsel couples to decide joint decision and they decide together. However, women can decide by themselves.

Now we are going to discuss about the marriage related process prevalent in this community.

I: In your community are women who delivered recently legally allowed to receive PP and PA family planning service?

- Is these same for boys and girls, people from different age groups, education background, ethnic groups, socio economic classes, excluded groups? How are they different? Why/ Why not?

R: Even though I have no idea on this, the government is designing different strategies to improve the services.

I: How do recently delivered/aborted women access PP and PA family planning information and resource from the healthcare facilities?

- Is these same for boys and girls, people from different age groups, education background, ethnic groups, socio economic classes, excluded groups? How are they different? Why/ Why not?

- Do the people in your community support recently delivered/aborted women to access PP and PA family planning information and resources? How do they provide support? Who are these people? Why do they provide support?
- Are there certain groups in your community who do not support this? Who are these people? Why don't they support?

R: university students, educated once ,mothers can get information from internet, radio, TV,

They can get information from "Yebeteseb tena radio program" being conveyed. On top of this they can get information from the health extension workers, health workers. The information is available for all groups and all groups can equally get information because we arrange meetings for all age groups and disseminate information. However there could be individuals who don't use any of the information if they don't listen to mass media.

People accept what we say. There could be very few people who can oppose. Those who didn't understand the purpose may not be willing to accept.

We are using different opportunities to teach the community about unintended pregnancies and its consequences at national level.

I: Please tell me women's experience with accessing healthcare information and resources including PP family planning.

- Do you feel they have a right to get healthcare information and resources including PP family planning services? Why/why not?
- Do you think women's experience is different from other people in your community? How? Please tell me more
- Is there legal mechanism you can follow to ensure they get quality healthcare information and resources they need? Please tell me more

R: yes they have right to get information. We are in enlightenment period ,people can learn and understand what we say and use the services.

Regarding to legal mechanism where we can ensure whether they get quality health information, there is legal mechanism up to the grass root level, kebele, health extension. Because we cannot

address all community members we can ensure whether they get quality information by using different networks ,one to five and one to thirty networks.

I: Thank you

R:Thank you very much.

Summary

- ✓ Most people don't prefer IUCD,due to myths and miss perceptions "*we are exposed to mich'',it doesnot give them comfort*
- ✓ Unmarred ones/adolescent don't use family planning services after abortion, it is good address this groups
- ✓ There are people who live in very far and hard to reach areas, don't access the services,so is one of the challenge,
- ✓ "people travel four to five hours until they reach the health center",there are transportation costs which they don't afford.
- ✓ Some equipments may be out of use, for example forceps, scissors,there is lack of rooms and it is not good to put the drugs
- ✓ There is lack of awareness about the advantage of partum family planning services
- ✓ Rather than waiting people to come to the health center addressing people who are not coming should be a key intervention
- ✓ The health workers who are open and raise different questions during meeting will be tagged and the politician will take measures up on such people.so they are not supposed to mention such gaps during meeting. "The expert asked me to hold while recording"
- ✓ Adolescents hide themselves and don't come to health center. They come after pregnancy occur, they don't know emergency contraceptives.

In-Depth Interview conducted with health center Head at Wadu health center

by Deginesh Dawit

Sociodemographic information of interviewee

Code	Keble	age	sex	Educational-status	Job responsibility	Marriage-status	Work experience
IDI_WHC_HCH_250722	Wadu health center	46	F	BSC Degree in public health	Head of Health centre	Married	30 years

Assets and Resources

Discussion.

I; Health services (PP and PA family planning services) availability: Can you tell me the types of healthcare facilities that provide PP and PA family planning services in your area?

R; This service already started in this health center and previously given by some limited trained midwifes. But currently, since Engeder health has given training for health workers, this service comprehensively provided in this health center.we have started delivering postpartium family planning services. We are trying to expand the services, post-partum services should give at spot immediately mother has delivered; previously it was not practiced. The history of family planning service delivery was providing pills, when shifting from pills to dippo women resisting and there were rummer people say dippo was not good for health. When shifting from short acting to long acting it tooks time convince, clear out people perception. We started the delivery post-partum family planning but still it needs work, creating awareness for the women especially

women from age 15 and above when they has start pregnant, we have to teach them when to start family planning, how to use family planning. We already started delivering services but needs work on creating awareness.

I; What types of FP services are available at the healthcare facilities?

R; now we deliver services, already women receiving dippo decreasing and almost long acting family planning has been given, especially in this health center juddle and IUCD services are provided. It would be better one mother injecting three month about dippo, taking long acting family planning is better. In mothers perceive it is better taking long acting family planning three to more years than injecting dippo for three months bases and fear of missing the appointment. Even there is difference in when to start fp, people perceive it is good to take long acting FP. All type family planning services available and provided in our health center.

I; Are these services equally available to boys, girls, young, old, married, unmarried, people from different geographic locations, religious or cultural background, language etc.? Please tell me more

R; if client came to take the fp services we have available services, I think everyone want to take services we have supply, and we have suppliers. No shortage of family planning and we suppliers. In this year, we are planning and giving family planning in room of youth friendly services, previously we had only one FP room, FP services were delivered. But starting from this year we, are delivering fP at youth friendly services. We had review meeting discussion on YFs services and shifted the YFS room to be convenient to them who want to get fp services. It was integrated with FP services and can address both YFS and FP. We are delivering FP for all members of community who have need for FP methods. I think all who need FP can access the FP methods in our health center.

Regarding to post abortion care; we are delivering post abortion care for our clients. We have trained health professional only in post abortion care. Who get abortion care should take post abortion care. We address post abortion care, and counseling for the clients. We have problem with instrument for post-partum care service. We requested the engender health organization, what need the instruments for PP and PA care services, we lack some instruments and materials. We reported to the engender health needed materials for PP and PA. Now we are using instruments borrowed from other health centers. We are delivering all services who need the services without difference.

For males, firstly we are providing awareness creation, teaching them, the room convent to address the males FP need. We distribute condom for males, we put condoms in accessible place. You can see the outside we put condom. Youth came to our health center to get information, either boys, grill with their friends came here.

Accessibility of the PP and PA family planning services

I; Can you tell me how far the PP and PA family planning services providing facilities are located? How long does it take women to access the facilities? In addition, what type of transportation means do they use to reach the facilities? How easy or difficult is it to use the transportation services?

R; We have two catchment kebeles far away from this health center; it takes more than 30 minute by car.it is difficult to travel by walking. We use motor and car to reach. We teach on spot mothers gather. We have trained health workers and collaboration health extension worker teach mothers in their community. We ask mother where to deliver, how many years stay using FP, what type of FP she chose. We teach them about immediate postpartum FP.

Those near kebeles came to our health center through walking and use baggage to reach health center.

For those who far away from the health center for delivery services mothers use ambulance services, for FP and another heath health activities we travel to the community. We integrate another health activity like TT surgery, screening, we sent health workers to the community. We are providing FP at Health post level, trained health worker give FP.

Some mothers easily accept PP FP immediately after delivery, they are happy receiving here,. Some mothers are not deciding immediately and they say after 45 days I will take FP. trained health extension provide for those mothers long acting family planning. Our aim is women receive FP especially long acting FP. Previously mainly provided FP was pills, it was not such much effective. As we talked previously there is shift from dippo to long acting FP.we are working with health extension worker to provide FP who are not receiving postpartum immediately after delivery.

I; Is there any cost associated with accessing the PP and PA family planning services? Please tell me more about this

R; till this time there is no requirement of payment regarding FP, delivery services. There are suppliers of the FP and they do not require payment for the services. The expense covered of our office even you go to rural areas to provide FP. It is the agenda of government that all reproductive age group should use FP. We are trying to provide FP immediately for those who deliver in health facility and those get abortion care. The reason is that if people should use FP immediately after delivery. That concept might change the community perception about FP.

I; Is the PP and PA family planning service is accessible at all time at the healthcare facilities? Are there types of FP methods of users' choice in the healthcare facilities?

R; perhaps Saturday and Sunday is for emergency services but the family planning room is not locked during these days. You can see always the keys for FP and YFS is there. The good thing is three fourth of midwives are trained postpartum care. There is a high chance for clients want to receive care at weekend days because keys of the room at hand of midwives. Because rooms open and I don't think the client have difficult to access the services. Clients can get access for PP and PA care at Saturday and Sundays because those three forth of midwives trained attending deliveries at those days. We have assigned 6 midwives in one day, morning two, day time two and night time two. Of them 4 trained PP and can provide the services.

Firstly, here mothers have choice to get FP types. Secondly, the FP services use is based on client's choice. Women can chose their preferences, as the health worker we are giving information on the benefit of each type of FP. We counsel here are a lot options and give information on each. Clients have an informed choice. In my observation, among women there is no problem with receiving FP.in previous days we insist women to take FP, but currently women request to get FP from us. There is no problem with receiving FP. Now our main issue is providing FP immediately after delivery. Ender health also trained professional on postpartum FP use. Our main work is creating awareness on PP FP. I have experience in working in FP, previously clients pills and then shifted towards dippo. In my observation now clients use long acting FP. We have to focus on PP FP. Our activities know is when clients used the FP, at spot after delivery.

I; Are these services equally accessible to boys, girls, young, old, married, unmarried, people from different geographic locations, religious or cultural background, language etc.? Please tell me more

R, *EEEE, by the way not only FP, for all health activities there is no difference on clients back ground of language culture, and geographical difference. Regarding youth services the main issues is not whether married or not, the main issue is what services they want to receive. Previously it is called the youth reproductive services, but now changed to youth health services. It includes FP, and given for all need the services either female or male, married or single. There is high chance to get services. Clients can get without any differences. Training and orientation was given for all, there is no difference on clients background, educated, not educated.*

I; How easy could they access the PP and PA family planning services? Do you think that their access to the PP and PA family planning services are affected because of their background? How?

R; as previously I mentioned , there is challenge in shifting from one method to other, you can see the challenge from shifting from pills to dippo, then to long acting FP. I believe there is challenge in providing postpartum FP for delivering mother, because you have to start the teaching mothers from their beginning of pregnancy process. Previously we teach women about to use of FP 45 day after delivery. For PP fP method utilization requires training to teach mothers throughout the pregnancy. It is an issue not only our health center staff, it needs thinking and participation from higher officials. Postpartum FP might be effective for mothers, newly delivered babies and families .if she take PP FP, she don't think about the pregnancy but

if say she has to take 45 days after delivery she fear about the pregnancy. For pp FP it needs work to convince clients, it is not jumping to give the services.

> *There is a difference on their education and place residence on the utilization of PP FP. I have experienced that we counseled to give PPFP immediately after delivery, she came from rural area. It is difficult to assume all can accept PP FP, there is a difference in understanding among not educated and not access from different mass media and those have access to education and access to different mass media. We have to create awareness among community regarding the PP FP, we have train the community. If we strength our activity I belief we can bring change on PPFP that is beneficial for the client. It might have challenge and resistance those do not aware, we have create awareness and can bring change. There is a difference among urban and rural residents. There might be the effect of culture. To change the perception it needs work and I belief it will change.*

Means to access the PP and PA family planning services

I; Please tell me about your facilities' resources and assets. Please tell me the resources and assets belong to these health facilities to provide PP and PA FP service.

R; permanent assets are health center itself, the building, workers. There are other temporary materials and supplies, for example we have digital BP cuff but not functional. We share BP cuff among FP room delivery, OPD. Delivery sets, these assets are temporary and give services for specific period. Some time we get shortage of FP, sometimes suppliers do not provide on time due to supply shortage. We face shortage of supplies; in here, we faced the shortage and borrowed from other woreda.

For PP and PA we use IUCD, we face shortage of instruments and we requested suppliers about instruments and supplies of PP and PA care. We face shortage of PA care supplies, most of the we cannot get some supplies and instruments used for PA care. Even some times we face shortage, currently we have supplies of IUCD and other FP supplies can be used for PP services.

I; How is the use of the facility/Woreda health office resources and assets decided? Who makes the decision? Do you involve in the decision-making? How do you think that affects access to the healthcare services including PP and PA family planning by women?

R; firstly Health center decide, those involved in service provision decide on it, when deciding how much done in this month thus can be used as base for next month planning. They select and identify what supplies needed for the service provision. When you providing training, you are improving the knowledge and that enhance the practice. Now we training health extension and coaching them. Those in pinpoint identify what they need and plan for the supply request. We have logistic coordinator request supply for the health center. When we update here the system aware what supply needed in wadu Health at central supply chain. We provide the PA care it indicated on online system what supply we have used. Pinpoint (MCH) focal are responsible for supply used and request accordingly. MCH focal request the supply need and Health center director approve and sent it to woreda.

Last Friday we were discussing with MCH service providers about how much PP care and PA care activities done. As a leader of health center; I monitor and evaluate the progress of the PP and PA care. Asking questions like, what are the problems there, why not achieved according to the plan. Regularly on Friday, we discuss and record on minute about our discussion. As this health center, we are focused on PP because most of the kebeles are rural area and mothers

might be challenge to get access. I call all health extension and we review our activities performance, discuss, and forward the direction. We discuss with extension refer mothers for PP and PA care. We planned to strengthen PP and PA care in this health facility. It gives pleasure and happiness for health workers when those mothers used FP and led their life. I had many years' experience on FP provision; today mothers acknowledge you helped me to lead my family providing FP. It gives me happiness. Fp activities are activities carried out in creating awareness.

I; Please tell me about income of in this Woreda. Please tell me about your woredas income.

R; health center has their inside generation of income from fee from service users. Health center monitor income generated from Health center. We planned to generate 1 million birr in this year. Sources through fee and CBHI. We have changes for example we provide delivery services free. However, to deliver one mother we utilize 6 gloves each costs 60 birr, gauze, and stich for free. Minimum 300 cost to deliver. However, do it free? We provide fP for free that reduces our income.

I; how is the use of the woreda's income decided? Who makes the decision? Do you involve in the decision-making? How do you think that affects their access to the healthcare services including PP and PA family planning?

R; town administration support some amount for purchasing of drugs, we allocate for administration cost. We generate income and decide on it but there is no sufficient income generated from health center. We have lack of budgets for purchasing supplies. It is difficult to run with income generated from health center. We collect income from different services provision from laboratory services, drugs, CBHI and other sources. I have role in generating income in my Health center. There is a direction from top we have to collect one million birr and

utilize it for running the activities. You have generated the planned birr. This health center administered by health center Board, leaded by the mayor of the town. There is review meeting and audit the generated income and decided by the board to utilize the income. Fee revision also carried out by Board. The health center management facilitates the activities of the health center. Those services FP, maternal health services provided freely and incur the cost and burden on health center finance. We inform such problem for higher officials because it affects our finance. We inform the for the board about compensation method for freely provided services and we also facing problem market fluctuation. Previously one glove cost ten birr but it is about birr that affects the health center.

I; In your woreda how is use of PP and PA family planning seen? Is it acceptable for women to use PP and PA family planning? Why/why not? Please tell me more (what about boys and girls)

R; regarding acceptance of the PP, as I said previously, there are early adaptors, late adaptors and not accepting the new approach. In addition, the acceptance of PP likes this. Some accept easily, some look about others around them. Some are also do not like others taking FP, so there are different groups of people. I have experience that one mother have used dippo for 8 years herself but trying to challenge other women came to me to take FP, There are mothers don't want others to take the family planning and hinders other not to take. I could suggest working activities creating awareness on PP FP. knowledge based work should be carried out. It is difficult to bring change without creating awareness among clients. I belief creating awareness among women, it might take time to bring change.

We started providing PP FP in this health center two months back. But few mothers received PP fp. Not all delivered mothers use linked with PPFP. The problem is we ask those mothers come to delivery.

I; In your opinion what should be done to improve access to the PP and PA family planning services for all women in your community?

R, Ok this is a good question, to improve access to we have work on community, when mother starting pregnancy journey. We have to train and create awareness on PP and convince mother to receive the services. Before coming to delivery mother should be convinced. You have ask mothers her pregnancy was planned or not, you have built rapport with mothers and awareness the availability of this services in the health facility. As I have told you family planning needs time to change mothers use the services. It might not change easily, it takes time. Mother should aware and plan for this services.

Practices, Roles, and Participation

1. Please describe to me the typical Woreda health office officials/experts roles in your Woreda/community?

I; How is Woreda health office officials occupied over the course of the day? Are there seasonal differences (campaign/political assignment) in how you use time?

R, it is the role of different actors can participate. Health system can carry out these activities. In community level health extension worker can do, HDA leaders can help to gather mothers, HAD leaders are aware and can carry this role in community level. in here there are assigned in MCH have role to work this activity. The work should be carried out before coming here to get delivery services. Health center works should visit community HDA leaders and capacitate by training them.

As health center leader, my responsibility is following, activities health carried out in right way and the community linking and referral contributes this work. My responsibility integrated the community work and health center activities. We have pregnant mothers forum in each Keble, using the opportunity we can improve FP. My role is addressing all kebeles and assuring the activities done appropriately, monitoring, providing supplies.

In 24 hour , In health center, I carry out admiration activities following and ensuring activities are carried out in proper way. I also involve in the community workers, providing supplies and supporting the implementation of all health activities can be campaign. Monitoring and follow up activities, whether clients received their services or not, I check it.

I; As Woreda health office official, what kind of work do you do? Do these works different when facility status changes (Rural, urban and geographical locations)?

R; it is different, when you are worker and Leader; leaders involve different seasonal activities with additional to routine activities. Some time you are to busy. During campaign session you are became too busy and stay without eating lunch, you have follow the assigned members and support logistics for them. When you are health professional assigned you need only eight hours but leader stay more than, you can came early morning to till nighttime.

There might be patient and client load in town and rural health center. Town there are many clients to serve compared to rural and needs addition health workers for town. Extension of health facilities also needs.

I; Do Woreda health office officials like you have restrictions/barriers on the accessibility? What restrictions/barriers? How do the restrictions/barriers influence the access of the PP and PA family planning services and supportive social networks?

R; in this health center we have opportunity to start PP, because we have trained health workers on PP Fp. I think the challenges is we have to start work on community regarding PP FP and PA care, should participate males , youths and women to be effective in providing services. Health center has limited budget, it requires financial support for the delivery of services. We need logistic and financial support for strengthen services.

I; Do you participate in any meetings, workshops, trainings, pregnant women conference, family, community, government, or other social networks organizations? Please tell me more.

Please tell me how your participation in these organizations affect the PP and PA family planning services provision

R; I have participated in pregnant mothers forum to see the how health workers train mothers. Pregnant mothers are happy when you discuss with them and hearing them. You make relationship with them and build rapport and it make opportunity to teach PP FP. When I teach Fp for mothers use an example of bread ("Kita") when he is alone this kita belongs to himself only. When he marry he shares with his wife and when they have one child the kitta shared by three and then two child the father get a quarter of kitta. We teach mothers about family planning; I give this example and when you add more child you are minimizing kitaa of your shares and convince them to use fP. Pregnant mothers forum is a good opportunity to teach PP Fp.

I; In your Woreda do women assume leadership roles? What types of roles? Do these roles change by social characteristics, such as (dis)ability, economic status, health status, educational level, religion, culture, geographical location, and marital status?

a. Please tell us about your own experience with assuming any leadership roles?

b. Please tell me how your leadership roles affect the PP and PA family planning services provision

R; currently, management members are nine and four of them are females. Emergency case team leader is female, Human resource team leader is female and acting as deputy following me. The third delivery case team leader is female. The fourth one audit team leader is female. Around 40% of management members are females. In my opinion female came to leadership, it would be better. Post-partum service also given to women and would be better led by female leader. I had experience on family planning and I myself became female. Ahhhh mothers tell us about what they face the side effects of FP they use. If females lead the it could be better to understand the mothers feeling and provide according to their need. You need to discuss sitting a long time with mothers, for this reason it would be better led by females. I have no reservation if females led and I support.

For the position we are assigned by woreda health office leaders. I have opinion to became a leader females should be preferred themselves to shoulder the responsibility. When you came to leadership there is a lot of burdens, you need to work for 24 hours. Sometimes they call at nine hour of night time for ambulance, my wife ask who are calling for me at night. There are many constraints in work places therefore females should be prepared to take the responsibilities. Females can work more than males, if the responsibilities given.

I have many years of experience working as professional. I could wish to work under other leadership. I would prefer providing vaccine, treating child room working as service provider. But the organization assigned me to lead the health center and give the

responsibility. I prefer to work under others leadership, I have experience that I am an old person and recruited one young boy as my child and he became a leader in my organization. I was under his supervision and I like it to work with.

My long experience in working on FP planning and this leadership role helped me to focus on FP. We discuss with our team to expand work regarding family planning focus on post-partum FP and PA care for need group. We have to work with based on knowledge and creating awareness of the community.

2. In your Woreda/community who decides at what age people marry? What are the reasons for getting married at younger or older ages in this Woreda?

I; Please tell me how their age at marriage affects their PP and PA family planning services use

R; kkkkk, this is strong question, as we health workers educate the community not marry in under age. You know one can be physical fit for marriage that means not mature for the marriage. It needs physical mental and psychological maturity for marriage.

There is social pressure for early marriage, their relationship. Previously household decides for their child's marriage but now days families cannot decide for marriage. Currently they themselves decide to marry.

I; What are your suggestions to improve women's roles and participation to enhance the use of PP and PA family planning services?

R, problem of early marriage is risk, after the marriage the next stage is became pregnant. Those early married do not understand the consequences of risks related to pregnancy. Even they marry early should wait before becoming a pregnant, they should develop knowledge regarding family planning. This concept is very important that married people should know. I think when couple think for marriage they should have think about FP. Those lately marry have tendency to

deliver more children without using FP. It has also their own risks. I believe there should understanding of FP.

To make more participation as government directs need on focusing youth health packages. Youth health services should not only limit to health center but focus youth center, school level to access youths to provide youth health services. Training should be strengthened. Training should contain all packages like Fp, early marriage and its risks. We sometimes gather females in kebele level to discuss health issues; we have to use such opportunities to teach FP and PA

Knowledge, Beliefs, and Perceptions

Now we are going to discuss about the issues faced by women in accessing PP and PA family Planning services in your opinion.

R, when you advise them to take PP FP and PA care they might resist. It needs long time creating awareness to convince them. They may perceive why he asks me immediately I deliver and might ask themselves.

It is a new thing for the community, PP FP, if newly delivered mother receive the PP FP community might perceive why she take immediately, what happen if she wait some days. If you insert IUCD after 45 days it will be normal for the community this is a FP method they practice. When you provide immediately community might perceive why she hurry for this. If she received PA care immediately they perceive the same.

I; In your Woreda what is appropriate behavior for a woman after delivery and abortion regarding the use of PP and PA family planning? How do they differ from adolescent girls and young women?

How do these believe influence health behavior including the PP and PA family planning use?

R; in Wolaita culture the recently delivered mother stay at home at least for two months even she don't go to churches, if she is orthodox religion follower and gave birth to female should stay at home until here new baby get Christianity at eighty days of delivery during this time even she don't go to neighbor's home so it is difficult to them to come to health center before the day of Christianity for vaccination also she sends her baby on other person so it is better for her to receive the service before going to her home.

I; What are the social beliefs and perceptions that share women's expectations and aspiration for health care including PP and PA family planning?

R; . *Now a days those live in urban and young mothers easily decide to use FP. Some encourage others to receive FP. Without any pressure from external body partners should have make decision to use FP*

1. In your opinion who should make decisions regarding use of PP and PA family planning services? What types of decisions do women make regarding use of healthcare including PP and PA family planning services? What types of decisions regarding use of healthcare including PP and PA family planning services are made jointly?

   R; who should make decision, mother should make decision to use PP FP. In my belief mother should make a decision to use FP or the family together should make a decision. Primary the decision should be carried by partners but health workers can facilitate a decision. Hearing from the partners; health workers should support for the decision to use FP. Health workers should provide choices. If partners make their own decision, it will be effective. We observe the decision made by others, the FP fail and early removal of FP method.

Legal Rights and Status

I; In your community are women who delivered recently legally allowed to receive PP and PA family planning service?

R; regarding legality the question should be answered is why this FP provided. As we think as health workers it is good that all mothers after delivery receive FP.

I; How do recently delivered/aborted women access PP and PA family planning information and resource from the healthcare facilities?

R; regarding getting information, primary she can get information from health extension workers. They can get from meeting like pregnant mothers forum, when females gather many things including their secrets. In peer group discussion, they can get information. When they the discuss themselves that makes them easy accept the PP FP and PA care. Therefore, we have to facilitate the discussion. In meeting, church, you can make awareness creation among them they can easily accept the PP FP method and PA care services.

I; Do the people in your community support recently delivered/aborted women to access PP and PA family planning information and resources? How do they provide support? Who are these people? Why do they provide support?

R; There are members also positively make a pressure. You can find HAD leaders, religious leaders, those more contact with health extension workers, aged and in their life used FP, encourage others to get services. Those members model in using FP can help others to receive PP FP. In kebele level you might find mothers say I had used FP in my last stage and encourage others to use early stage of their life. There two type mothers, the first one in similar age group eligible for FP and second aged group groups.kkkkkk those think pregnancy make you old, when

young deliver more child you are became more old,kkkkkk saying she similar with my age and using FP, and still she is looks young. I delivered more child and became look old.

I; Are there certain groups in your community who do not support this? Who are these people? Why don’t they support?

R, *Firstly is there community members oppose, yes there are who oppose. Always there are people think negatively. You have to teach them. That discourage who want to use PP FP, saying you are hurrying to use PP FP. There are also good facilitators within community members and bad meditators being obstacles for services. Some of members might tell negative things always*.

I; Please tell me women’s experience with accessing healthcare information and resources including PP family planning.

R, mothers taking fP , after delivery and 45 days when they came to child vaccination they use FP, but PP FP has yet started. Currently we are trying to start at spot mother deliver should take PP FP and PA care. Before we were doing and teaching mothers to use FP and but not PP FP using different women meeting including pregnant mothers forum. Now we have to shift toward PP FP immediately after delivery. We can use the supply and system available, it doesn’t require the new supply and system, we should have bring the concept of PP FP and PA care using existing system and approach.

When you start for FP, you deliver information to women; they can make their own decision to use FP. In here, we are working creating awareness when they deliver immediately use FP. We have to teach mothers, using HEW, HDA, to shift the use of Fp to PP FP use. It might take time as we have seen shift from short acting to long acting took time, we have always teach and create community awareness. We have use community mobilizers.

I; Do you feel they have a right to get healthcare information and resources including PP family planning services? Why/why not?

R; yes they have right to get service and information because all things formed for them and they are also citizen of the country.

I; Is there legal mechanism you can follow to ensure they get quality healthcare information and resources they need? Please tell me more

I; Providing quality services is the main task of health center. Currently the main thing is not coverage, it is about quality. The work for quality is given due attention from government and given direction. It starts from recording of the activities, in this Health center we have quality assurance team follow whether the services provided meet the quality standards or not. What are the barriers in providing quality services? One mother can report complain about their services, we have system getting feedback from clients. We have quality committee bring an issue of quality and we discuss to improve the quality. There system of reporting from pinpoint tracking quality and report to health center for discussion.

We have 14 indicators to monitor service provision quality. Those quality team monitor quality starting from recording of activities, clear history should be clearly written about FP, it needs quality of supplies, quality of place you give care for mothers. We have 14 standards of quality and according we perform our activities.

I; With this we have come to an end of the discussion. Do you want to add anything before we end the discussion or if you have any questions you want to ask?

R, Thank you, this activity might improve the health of mothers and should give time and due attention. Health activities are one of the government's main agenda. For effective FP mothers

should immediately receive after delivery. If we strength and follow this can improve mothers, child and family health, I believe it might improve the fp.

I thank you; behalf of Wadu Health center, coming here to make interview and discussion.

Thank you for your time and in case you want, you can get in touch with us through the details mentioned in the consent form.

****

Summary

An interview conducted with Wadu health center head at Wadu health center in 25/07/22. He has BSC degree in public health and 30 years work experience. He has worked around ten years in family planning service provision center, due to that he has adequate knowledge regarding postpartum family planning method. During an interview time, he was stable and gave sufficient information regarding the service. For me it is the longest interview I ever did and were satisfied with his answers. In general, the information I got from him is rich and sufficient. The key finding were:-the service utilization was low currently and it is not that much easy access all with in short period of time because shifting them from what they familiarized to new one will takes time. Both wife and husband should made decision together. To make the service easily accessible the awareness creation should be started at community level and convinced during ANC visit time. To address the service for young also the awareness creation should be focused on schools, youth center and wfs centers, rather than limiting the service to health center.

Code	IDI _WL_WO_1_7_2022
sex	Female
Age	20
Educational status	8th
Occupation	
No of children	1
Date of delivery	1/6/2022
kebele	Waraza Lasho

Now we are going to discuss about the Healthcare resources and assets available to recently delivered mothers in your area.

I: Health services (PP and PA family planning services) availability: Can you tell me the types of healthcare facilities that provide PP family planning services in your area?

a. What types of FP services are available at the healthcare facilities?
b. Are these services equally available to boys, girls, young, old, married, unmarried, people from different geographic locations, religious or cultural background, language etc.? Please tell me more

R: The services are given in the health center. 'Layta suggi yellanawu koyyiko' if a mother intends to give birth and want to have space between the next delivery, they give family planning methods. There is a method given in the form of injection, There is also a method inserted in the arm in order to prevent un planned pregnancy, there is Depo given , and they counsel us to plan. They teach us about this during meetings or gatherings. Health workers gather people and teach all pregnant mothers about the service . I used to participate in the meeting and get information when I was pregnant.

I: How do they teach you ?what do they teach about ?

R: They taught us how to keep ourselves to prevent unintended pregnancy and to have gap between pregnancies. They told us that ‘‘It is good to give birth but if there is ‘‘boli boli yelioy ’’(if the is no adequate spacing this affects your health’’, they teach us about the effect of unintended pregnancies.

Accessibility of the PP family planning services

I: Can you tell me how far the PP family planning services providing facility is located? How long does it take for you to access the facility?

R: The health center is not far. it is about five minutes.

I: What type of transportation means do you use to reach the facility? How easy or difficult is it to use the transportation services?

R: we don't use any transportation means, we go there on foot.

I: Is there any cost associated with accessing the PP family planning services? Please tell me more about this

R: I don't know whether there is any related cost because I did not started to use it so far ‘‘family planning’’

I: you mean you have no idea whether there is cost or not.

R: Yes, I don't know.

I: did not you hear about the services that the service is given free of charge?

R: The health care workers taught us about the services but they did not teach us whether there is cost associated with it.

I: Is the PP family planning service open at all time and is accessible with short waiting time at the healthcare facility? Are there types of FP methods of users' choice in the healthcare facility? Do healthcare providers receive and treat users with respect and dignity?

R: sometimes we might not get the services ,they let us wait for long time so we suffer a lot some times. ‘ *naagisiyone waaysiyo meray dees'*. However, some good health workers who

treat us well they don’t let us wait. They are not good some times. They way they treat differs from professionals to professionals. Some are good ,where as others not.

I:Do healthcare providers receive and treat users with respect and dignity?

R: during pregnancy ,they treat us well and they respect us.

I:I don’t mean only during pregnancy. What about during post partum and post abortion family planning” yelossara nne boshape simin immetiya halchidi haasidi haggazuwa’’?

R: they are good, good they serve us as soon as possible. There is no problem during this time. They treat us well.

I: How easy could you access the PP family planning service? Do you think your access to the PP family planning services is affected because of your background? How?

R:It is easy and I can access the services when I need it.

Means to access the PP family planning services

I: Please tell me about your household resources and assets.Please tell me the resources and assets belong to you.

R: Do you mean cattles, and sheeps and goats?

I: Yes ,any resources and assets you have? please mention what you possess?

R: we don’t have cattles.. loughing, we don’t have anything. As you can see. This is the way how we live.

I: How about the occupation of your husband?

R: He is working on wood works

I: How is the use of the household resources and assets decided? Who makes the decision? Do you involve in the decision making? How do you think that affects your access to the healthcare services including PP family planning?

R: It is my husband. the decision is made by him and also we discuss to gather and decide jointly.

I: How do you think that affects your access to the healthcare services including PP family planning?

R: ….hhh?what? ‘‘The respondent did not respond relevant answer may be she could be able to understand the question’’

I: In your community how is use of PP family planning seen? Is it acceptable for women to use PP family planning? Why/why not? Please tell me more

R: I think it is good.the community also have positive attitude towards immediate post partum family planning. Loughing…Don't you think it is not good if it 'family planning' is immediately given after birth?

I: What made you lough ?would you tell me please?

R: kkk lughing. I mean it is good to use family planning immediately after birth.

I:How about the community? how is use of PP family planning seen? Is it acceptable for women to use PP family planning? Why/why not? Please tell me more.For instance ,you have neighbors, relateives,father or mother in laws? How do they see?

R: The community support the use of family planning immediately after birth. But there is no problem if we use it " PP FA lately.

I: Did you get my idea? How do they see ? do the community encourage people to use immediately or discourage? if family planning is given immediately after birth?how do they see?do they accept?

R:people don't accept and they have negative perception.

I:Don't they accept?

R:Yes,they don't think see it"PPFP" as a good thing.

I:why do you think people see that way?

R: Because they think and say "*is the recently delivered woman going to have sexual intercourse with her husband immediately after birth?why do the health workers do that*? Therefore the community don't support immediate post partum family planning.

I: In your opinion what should be done to improve access to the PP family planning and post abortion services for all recently delivered mothers in your community?

R:health workers should gather mothers who come for antenatal care follow ups, and they should announce and gather community members , and disseminate information on the post partum family planning and post abortion F/P services, so that people can get knowledge and share their thoughts. Awareness creation should be done for the community to increase the uptake of the services.

Practices, Roles, andParticipation

Now we are going to discuss about your women's and your role and responsibilities.

I: Please describe to me the typical women's roles in your community?

- How are women and girls occupied over the course of 24 hours?Are there seasonal differences in how women and girls use their time?

R: I spend my time by preparing food for my husband,until I go to market I spend my time by ‘‘dantel sira’’ is locally known open works

I: Do you have barriers on your access? What barriers? How do the barriers/restrictions influence your access to the PP family planning services and supportive social networks?

- In your community do the restrictions/barriers change by social characteristic, such as (dis)ability, economic status, health status, educational level, religion, culture, geographical location, and maritalstatus?

R: what do you mean by barriers ‘‘ xube gidiyabati aybe’’?

I: What are the barriers/obstacles? influence your access to the PP family planning services and supportive social networks? xube gidiyabati aybe’’?yelidossara halchidi haasidi yelanawu immetiya hagaazuawa ekenaadan digana dandayes giyobati aybe?

R: There is no any barrier.I can access the service and use it ” PPFA’’.

I: can culture, the community perception be barrier for accessing and using Post partum family planning service?

R:For me these cannot be barrier.

I: Do you participate in any family, community, government, or other social networks organizations? Please tell me more. 1 to 5 or 1 to 30 meetings?

- Please tell me how your participation in these organizations affect your PP family planning services use

R: I haven’t participated in such gatherings. I didn’t participate so far. We rarely participate in community gatherings during pregnancy time.

I: Did you get any thing from the meeting?did they teach you about this matthers” PPFA?

R:It is not as such satisfactory.

I: Do you think   it is important to attend in such gatherings?what benefits do you get from meetings?

R:yes it is important for people who have good mind…I mean those who can understand what is being said by health workers, because they can get information from health workers and use the services.

I: In your community do women assume leadership roles? What types of roles? Do these roles change by social characteristics, such as (dis)ability, economic status, health status, educational level, religion, culture, geographical location, and maritalstatus?

- Please tell us about your own experience with assuming any leadership roles?
- Please tell me how your leadership roles affect your PP family planning services use

R: They don’t participate in any leadership roles.

I: In your community who decides at what age should people marry? What are the reasons for a women getting married atyounger or older ages?

- Please tell us about your own marriage experience? How old were you when you marry? What are the reasons you she get married at that age?
- Please tell me how your age at marriage affects your PP family planning services use

R: Loughing….this day boys and girls are rushing to get married.

I: why?

R: they feel that they reached to maturity level, so they decide by themselves to get marry without their family approval.

I: what about you? Please tell us about your own marriage experience? How old were you when you marry? What are the reasons you she get married at that age?

R: Louging…my experience is also similar to that of others .I mean I got married without my family approval.Loughing..... I was get married when I was in" tammane giyogan gelaas''

I: What do you mean" tammane giyoogan?'' were you get married when you are close to twenty or far below twenty?

R:It is far below (15-16)

I: what are the reasons you she get married at that age?

R:I got married due to peer pressure. My friend knows everything and she intentionally spent time with me and when it get dark my husband and others took me away and it became marriage. It was not my plan, it was done without my willingness by the time to get marry.

<u>Knowledge, Beliefs, and Perceptions</u>

Now we are going to discuss about the issues faced by adolescents and women in accessing PP and PA FP services in your opinion.

I: In your community what is appropriate behavior for recently delivered woman regarding use of PP and PA family planning services? How do they differ from adolescent girls and young women?

a. What is the appropriate behavior for a recently delivered mother? How do these believe influence health behavior including the PP family planning use?

R: The community don't see it as good.

I:why?

R: aybe kiyay ha'I yelossara? Why does a recently delivered woman rush to use immediate post partum family planning? It is a big deal for the community to use PPFA immediately after birth.

I: what about you ?what is your perception?

R: personally I agree with the community. I don't think it is good to start Post partum family planning as soon as a woman get birth.

I:why do you think that way?

R: It is good to start family planning but, why is it given immediately after birth? what is its significance?it is good if there is time gap after delivery and start to use family planning.

I: What are the social beliefs and perceptions that share women's expectations and aspiration for PP family planning?

R: There are negative perceptions from the community side.

I: In your opinion who should make decisions regarding use of PP family planning services? What types of decisions do women make regarding use of healthcare including PP family planning services? What types of decisions regarding use of healthcare including PP family planning services are made jointly?

R: I can decide by my self with out any discussion with my husband..loughs..but usually we discuss and decide things jointly.

Legal Rights and Status

Now we are going to discuss about the marriage related process prevalent in this community.

I: In your community are women who delivered recently legally allowed to receive PP family planning service?

- Is these same for boys and girls, people from different age groups, education background, ethnic groups, socio economic classes, excluded groups? How are they different? Why/ Why not?

R: what do you mean?

I: In your community are women who delivered recently legally allowed to receive PP family planning service?

R: Yes it is allowed.it is allowed for girls, boys for all.

I: How do recently delivered women access PP family planning information and resource from the healthcare facilities?

- Is these same for boys and girls, people from different age groups, education background, ethnic groups, socio economic classes, excluded groups? How are they different? Why/ Why not?
- Do the people in your community support recently delivered women to access PP family planning infatuation and resources? How do they provide support? Who are these people? Why do they provide support?
- Are there certain groups in your community who do not support this? Who are these people? Why don't they support?

R: We get information when there is gathering or meetings. And we get information informally when we discuss about our home matters during coffee ceremony.

I: DO males discuss on such matters or females only discuss during coffee ceremony?

R: Females only discuss.

I: what do you discuss about during coffee ceremony?

R: we discuss about family planning, share our experience for others

I: while you are discussing during coffee ceremony , Do the people in your community support recently delivered women to access PP family planning infatuation and resources? Are there certain groups in your community who do not support this? Who are these people? Why don't they support?

R:Yes there are people who support women to access and use family planning services.

I: Who are these people?

R:Those who got married people support us.

I: Are there certain groups in your community who do not support this? Who are these people? Why don't they support?

R: Are there certain groups in your community who do not support this? Who are these people? Why don't they support?

I: There is no community group who don't support this. all of them support the idea.

R:How do you see these two ideas?because you were telling me that there are certain group who don't support the idea and now you are telling me that there are no certain groups who don't support the idea.

R: Actually there are two groups. Those who support and don't support.

I: who are those people ?who don't support?

R: They are aged ones,very old people don't support the idea or " to access PP family planning infatuation and resource".They say that this not exits in our ages, this is what current or modern people do. " *we did not use family planning but we could be able to nurture many of our children''*.This people discourage others not to access and use the services.

I: are there such perceptions among young age groups?

R: No there isn't. The young groups support the use of PPFP because use of post partum family planning helps a lot and it helps you not to loss societal values because if you give birth without spacing the community gives less value. If I give birth without spacing I will be in danger "boli boli yeliko wooqagays" 'I may decay' ..useless. Therefore, it is better to use family planning

and wait until the current child gets matured and it is good if there is time gap between pregnancies.

I: Do you feel you have a right to get healthcare information and resources including PP family planning services? Why/why not?

- Do you think your experience is different from other people in your community? How? Please tell me more

R:Yes I feel that I have a right to get information.

I: Is there legal mechanism you can follow to ensure you get quality healthcare information and resources your need? Please tell me more

R: yes we have legal mechanisms. We used to raise questions if we don't get information, we ask health care workers if they fail to disseminate information.

Summary

This is interview conducted with recently delivered woman in waraza lasho kebele.

- ✓ She had knowledge about the effect of unintended pregnancy
- ✓ Did not start to use family planning and doesn't know the service is given free of charge
- ✓ There are negative attiude, perception about PPFP
- ✓ Awareness should be given to the community to increase the uptake
- ✓ She was influenced by her friends ,Peer pressure was one of the factor that force them to marry at their early age
- ✓ Old age groups discourage recently delivered mothers ,not to use PPFP
- ✓ "Why does a recently delivered woman rush to use immediate post partum family planning?" she shares the idea of community some how. She wants to use but being immediate after birth does not give her comfort

**Interview no**	**01**
**Interviewee code**	**IDI-WL-HHC 1/07/22**
**Sex**	**Male**
**Service year**	**10 years**
**Job title**	**Head of the health center**
**Name of interviewer**	**Befekadu Bekele**
**Date**	**1/07/22**
**Time started**	**7:00**
**Time finished**	**8:18**
**Venue**	**Waraza lasho health center**

Now we are going to discuss about the Healthcare resources and assets available for provision of PP and PA family planning services in your area.

I: Health services (PP and PA family planning services) availability: Can you tell me the types of healthcare facilities that provide PP and PA family planning services in your area?

a. What types of FP services are available at the healthcare facilities?
b. Are these services equally available to boys, girls, young, old, married, unmarried, people from different geographic locations, religious or cultural background, language etc.? Please tell me more

R:These services are given based on women's preference. We have methods of family planning in the health center. Oral contraceptives, implants, jaddle, IUCD. We provide the service after we counsel them. After we take consent from mothers we give her options. Mostly we counsel them to use long acting family planning methods. After counseling we give the services based on her preferences.

These services are available in the health center. However, we have budget constraints to fulfill some resources like gloves sanitation materials, soaps and other necessary services. Those working in MCH department midwife's, public health workers are not satisfied due to budget

constraints. We get the family planning methods based on our request. We don’t have shortage except from gloves.

Post abortion family planning service is also being given. There are trained midwives on post abortion and counseling services. There is adequate number of trained professionals. We are giving post abortion family planning services. The methods are available for both males and females. We don’t have problems with Family planning methods but there is a lot of things to be done because there is challenge regarding counseling. Not only for community members coming to the health center but also there needs to be much emphasis on the community level, kebele administrative, health extension workers, health development armies(HDAs).Women conferences are being conducted at kebele level but there are some constraints to conduct the conference on regular basis.

Most of community have awareness but there are some religions which don’t allow their followers not to use family planning. Regarding abortion most community’s perception is not good. It seen as sin. Community have negative attitude towards abortion but this kind of negative attitude is being changed after interventions has been made, on job trainings for health workers.

2. Accessibility of the PP and PA family planning services

a. Can you tell me how far the PP and PA family planning services providing facilities are located? How long does it take for women to access the facilities?
b. What type of transportation means do they use to reach the facilities? How easy or difficult is it to use the transportation services?
c. Is there any cost associated with accessing the PP and PA family planning services? Please tell me more about this
d. Is the PP and PA family planning service is accessible at all time at the healthcare facilities? Are there types of FP methods of users’ choice in the healthcare facilities?
e. Are these services equally accessible to boys, girls, young, old, married, unmarried, people from different geographic locations, religious or cultural background, language etc.? Please tell me more

f. How easy could they access the PP and PA family planning services? Do you think that their access to the PP and PA family planning services are affected because of their background? How?

R: There are health posts which found six kilometer away from the health center. There are two kebele which are not easy to access the services. The health extension workers there are not trained. There should be two health extension in the health post but in one of the health post there is only one health extension worker. This can be a challenge regarding accessibility. On job training should be given for health workers including health extension workers and this should be also done for health development armies to increase their awareness. Apart from this there is no problem related with accessibility except the quality of service given, lack of resources needed, skill gap among health providers. Therefore, awareness creation should be done and emphasis should be given for quality.

There are some difficult places where people cannot easily get transportation. Damota, Zulo areas are not easy for transportation because of their topography. It is difficult even for ambulance ,therefore people use locally made stretcher. The health post also lack different resources needed for family planning service provision. The places I have mentioned earlier are far from this so we give immunization services on monthly bases.

I: Is there any cost associated with accessing the PP and PA family planning services? Please tell me more about this

R: There are costs related with accessing the service. There is transportation cost for motor bicycle. They pay around 200 for two trips. If it is rainy season it becomes more complicated.

I: what about the costs related to these services once the came to the health facility?

R: Yes there could be associated costs. previously the price of glove is 5 birr only but now it is 50 birr people cannot easily afford. For a single mother came for post partum family planning three to five glove may be needed, they may also need fluid. However, no mother is forced to pay for these service once they came to the health center. The health center is doing its best. Previously there were local NGOs supporting on this area but there is no one supporting such

resources. On top of this they may pay for their meal as they come here. As far as I know there is no mother I remember who cost for gloves and other services given even though we have glove shortage. We prepare ourselves a head of time because we know the economic capacity of the community, so the cost related to family planning is totally covered by the health center.

I; Is the PP and PA family planning service is accessible at all time at the healthcare facilities? Are there types of FP methods of users' choice in the healthcare facilities? Would you tell me what is on ground?

R:No one denied of getting the service but there could be long waiting time sometimes. During lunch time and during evening times, and sometimes they may come in absence of the trained health workers. These services are not given in weekends,. If there are no trained health workers we immediately refer to other health facilities. Even though mothers wait during lunch times they get the services they need. The services is given regularly from monday to Friday.Most of the time their preference is long acting family planning methods and rarely there are mothers who prefer IUCD after they have got counseling.

I: Are these services equally accessible to boys, girls, young, old, married, unmarried, people from different geographic locations, religious or cultural background, language etc.? Please tell me more

R: In the kebele most of them are adolescent groups, There is Mega project in the kebele(Electricity project),near to the main city of Wolaita zone. However, there is no youth center in our kebele. We have scarcity of rooms in our health center. Even though there is a need from their side there is no setup for them. For example they need counseling services, family planning services, counseling after birth. But we don't have conducive environment which attracts them to come. There is lack rooms, there are also necessary services which are very important for youths. There is no youth friendly services given due to this reason. There is no special attention given for adolescents ,they are treated in the same manner with that of adults.

I:How did you relate availability of Mega project here and accessibility of post partum and post abortion family planning services specially with adolescents?

R: When there are Mega projects in your locality, there are many people coming from different place to seek job opportunity. There are daily laborers and other people. They can abort pregnancy due to different reasons. Mostly they come from such setting. We give PIHCT services and other services. Therefore there are people in need of the service. Mostly youth friends come to gather to get the services. In order to make the service accessible for youth groups we need additional rooms.

I: How easy could they access the PP and PA family planning services? Do you think that their access to the PP and PA family planning services are affected because of their background? How?

R: Their access to PP and PA family planning services can be affected because of their background. Both males and females when their age becomes fifteen and above their age contributes a lot. This is the age at which they disobey their families, get intoxicated after they go home from market places. Unless they are leaned about PP and PA family planning methods, they may not want to use family planning so this can affect utilization of F/P services among this groups.

3. Means to access the PP and PA family planning services

I: Please tell me about your facilities' resources and assets. Please tell me the resources and assets belong to these health facilities to provide PP and PA FP service

a. Please tell me about your facilities' resources and assets. Please tell me the resources and assets belong to these health facilities to provide PP and PA FP service.
b. How is the use of the facility/Woreda health office resources and assets decided? Who makes the decision? Do you involve in the decision making? How do you think that affects access to the healthcare services including PP and PA family planning by women?
c. Please tell me about income of in this Woreda. Please tell me about your woreda income.
d. How is the use of the woreda's income decided? Who makes the decision? Do you involve in the decision making? How do you think that affects their access to the healthcare services including PP and PA family planning?

e. In your woreda how is use of PP and PA family planning seen? Is it acceptable for women to use PP and PA family planning? Why/why not? Please tell me more (what about boys and girls)

R: During start of fiscal year we have woreda baseline plan. We evaluate our performance of last year and discuss on what we need for the next year and we include this during planning. As part of woreda baseline plan we plan on the resources needed for family planning service. Sometimes we have overachievements. We transferred budget codes from other capital budgets to priority services like maternal services. Due to this people are getting the F/P services they need. No mother sent home because of absence of gloves. But still we are struggling on this regards. We have health center board. Woreda administrator is the leader of health center board. We are struggling and trying to narrow the gaps by communicating with concerned bodies, other health centers. If the nearby health centers have over stock we share with together and we share what we have when they face shortage.

I: How is the use of the facility/Woreda health office resources and assets decided? Who makes the decision? Do you involve in the decision making? How do you think that affects access to the healthcare services including PP and PA family planning by women?

R: There is MCH focal person and other focal persons of each case teams and management bodies we discuss to gather and then we discuss with health center board members during board meeting and final decision will be done in board meeting.

I: Please tell me about income of in this Woreda. Please tell me about your woreda income

R:The income of the health center is increased as compared to the previous years because majority of the community members become members community based health insurance.Almost 70% of the community members were enrolled in CBHI scheme.As a result service provision is being improved, we have medical doctor so the number of attendants to seek care is increased.on average the case flow is from 40 to 80 attend on daily basis.We try our best to satisfy the community.The drug cost is also increased. Last year we planned 552 thousand birr but we 700 thousand birr is utilized for purchasing drugs.

I:How do you think that affects their access to the healthcare services including PP and PA family planning?

R: Even though post partum and post abortion service is given free of charges there other costs related with gloves and other resources which is still covered by the health center.Thefore when the income is increased this service will not be affected. Therefore, income has effect on PP and PA family planning services because in order to fulfill the resources needed the income matters a lot.

I: In your opinion what should be done to improve access to the PP and PA family planning services for all women in your community?

R: The good thing here is that we health care workers have awareness. In order to improve access to the PP and PA family planning services for all women in your community awareness creation should be done for health development armies, because they are very close to the community they can disseminate information, awareness creation should also be done for health extension workers, opinion leaders ,significant others, religious leaders, kebele administrative bodies. On top of this concerned bodies have to play their role to fulfill and support with inputs needed for family planning services. If that is the case the community can access the service and this may help both married and unmarried ones to lead their future life.

I: what additional thing should be done in order to improve access of PP and PA family planning services?

R: Awareness creation should be done at community level because once they have awareness they don't challenge us here while counseling. interventions should also be done in YFS, adolescents should get information before they get abortion services and post abortion family planning services. As we do prevention activities ,there should also be awareness creation done ahead of getting the services. There should be counseling services for youth groups or adolescents so that they can voluntarily start to use the services. Since most of them are students

they can teach others. Youth friendly service should also be there at health center in order to ensure accessibility for this group.

Now we are going to discuss about the issues faced by women in accessing PP and PA family Planning services in your opinion.

I: In your Woreda what is appropriate behavior for a woman after delivery and abortion regarding the use of PP and PA family planning? How do they differ from adolescent girls and young women?

a. How do these believe influence health behavior including the PP and PA family planning use?

R: Since we are on initiation phase we convince people after we counsel them to get the services.There are challenges we face. Because the community members raise different issues and they say ‘‘ *why do health care workers give family planning service immediately after birth ,while mothers are in the health center since they are not healed* ?’’because they think about the pain during labour time in addition to this recently mothers by themselves challenge us while counseling and say ‘‘ *I have been using family planning in previous pregnancies ,why is it needed to get the service here?’’ .*

Some educated mothers also ask the health workers ‘‘ *we heard that family planning is not given until 42 weeks after a mother give birth, we also heard that breast feeding can prevent pregnancy or it helps as family planning method but you let us get the service immediately after birth’’*

Uneducated mothers also raise different questions while we counsel them to get IUCD service ‘‘ *How is it possible to start/use the service while bleeding not yet stopped’’.* But there are about 3 women who received IUCD after training was given for health workers by engender health. There is fear from community side because this is new approach. In addition to this primimother says ‘‘ *I will not have sexual intercourse with my husband because I will go to my family and stay about six month ,so I don’t want to get service and I will start when I come back to my*

husband, why are you pushing me to get the service by now?’’ .Therefore, we have such a challenges.

I: What are the social beliefs and perceptions that share women’s expectations and aspiration for health care including PP and PA family planning?

R: The community has not yet get the service. We are conducting women conferences, health development armies are aware of this. There needs to be awareness creation at community level.

I: what about beliefs and perceptions regarding post abortion family planning?

R: There is no problem if it is safe abortion. however, for those who had unsafe abortion ,mothers say I had plan to conceive they say but we counsel them to stay one to two years. After we counsel them they become willing to start post abortion family planning. We don’t have as such challenges among adolescents. When there is unintended pregnancies occur they terminate it and willing to use post abortion family planning services. Whether they are recently married and unmarried ones they are willing to use the services.

I: Please describe to me the typical Woreda health office officials/experts roles in your Woreda/community?

a. How is Woreda health office officials occupied over the course of the day? Are there seasonal differences (campaign/political assignment) in how you use time?

As Woreda health office official, what kind of work do you do? Do these works differentwhen facility status changes (Rural, urban and geographical locations)?

R:As far as my roles and responsibilities concerned, we have different health packages to be done at health facilities, there are 2nd transformational agendas to be implemented here. We focus on four areas. Reform agendas, that is providing quality health service for the community, ensuring the service available and accessible to the community. We mainly focus on disease prevention within the health center and in the community level. Almost 85% of activities are

prevention based activities. We mainly focus maternal health services, communicable disease and non communicable disease, environmental sanitation, and organize to have better income for health facility and manage the budget accordingly. We make sure whether the clients are satisfied. We also make sure and follow whether health care workers are performing as per the standard. I also play my best to ensure and let my staffs give compassionate ,respectful and caring services for the clients.

I:How is Woreda health office officials occupied over the course of the day? Are there seasonal differences (campaign/political assignment) in how you use time?

R: Yes of course there are extra activities cascaded from higher bodies. We don't always perform as per our plan because of such extra activities. There are supportive supervisions being conducted at zonal, regional or from federal level. Even though there are extra activities we give priority for preplanned activities. However, we have lack of human power in the health center. Majority of health workers are females almost 75% of them are females. Some of them might be in maternity leave. Currently we have five midwives and two of them in maternity leave. They may stay 3 to 4 months at home. So we shift health workers from other departments in their albescence. Even though there are seasonal or emergency activities cascaded from higher levels, the routine activities will continue without any challenge. But due to lack of human power ,there are times where the clients wait for long time to get the services.

I: Do Woreda health office officials like you have restrictions/barriers on the accessibility? What restrictions/barriers? How do the restrictions/barriers influence the access of the PP and PA family planning services and supportive social networks?

b. In your Woreda do the restriction/barriers change by social characteristic, such as (dis)ability, economic status, health status, educational level, religion, culture, geographical location, and marital status?

R: One of the barrier in the community could be fear. The community members want to get service secretly. They don't disclose to the health care workers. There are community members who don't even want to come to the health center. They hide themselves but now this is being solved. For example people are not willing to come after post abortion some times they may

prefer traditional practioners. In addition to this they may also go to private clinics in sodo because if they come to health center they will be identified. That is why they are afraid to come to the health center. This is both for abortion and post abortion family planning service. There are community members who don’t even know whether abortion service is given here or not.However ,there are improvements and some people started to visit our health center.

I: Do you participate in any meetings, workshops, trainings, pregnant women conference, family, community, government, or other social networks organizations? Please tell me more.

Please tell me how your participation in these organizations affect the PP and PA family planning services provision

R: I have participated in workshop organized by Wolaita sodo university and ENGENDER health that was held at Halaba. In addition to this there was on job training here. My participation in this workshop is vital because I am the head of the health center, every activity is done under my leadership. Because I have participated in launching phase it easy for me to cascade the activity. I can play my role being part of stakeholder.

I: In your Woreda do women assume leadership roles? What types of roles? Do these roles change by social characteristics, such as (dis)ability, economic status, health status, educational level, religion, culture, geographical location, and marital status?

Please tell us about your own experience with assuming any leadership roles? Please tell me how your leadership roles affect the PP and PA family planning services provision

R: As I have said earlier 75% of our staff are female workers. We assigned them in different positions in different departments. Currently three of them are among management bodies. Though they are good in leadership, they give more emphasis for their home matters because they are overburdened with such matters. They are willing to take responsibilities. When women are assigned in leadership positions there are different advantages because mothers can disclose everything related to PP and PA family planning services as compared to males. They can also

share their experience to mothers while counseling. Females participation is more important than males because mothers are not ashamed of talking before females.

I: In your Woreda/community who decides at what age people marry? What are the reasons for getting married at younger or older ages in this Woreda? Please tell me how their age at marriage affects their PP and PA family planning services use

R: Currently adolescents marry at the age of 15 and above. Those who are grown up in church area obey their parents inform their parents before they get married and respect their family's decision. Most of the time they decide by themselves. Girls marry when their age is 15 and above. If there is trained personnel in the health center, and if they counsel as per the standard, and if there is follow up services after they get the service there will not be a problem. Unless the health workers follow ,and fail to counsel as per the standard the service can be affected.

I: What are your suggestions to improve women's roles and participation to enhance the use of PP and PA family planning services?

R: Female health workers and supportive female staffs can play role to enhance the use of PP and PA family planning services because they have awareness. Health extension workers, health development armies also play their role.

I: In your Woreda what is appropriate behavior for a woman after delivery and abortion regarding the use of PP and PA family planning? How do they differ from adolescent girls and young women? How do these believe influence health behavior including the PP and PA family planning use?

R: The main aim of using PP and PA family planning service is to nurture the current child or children and planning for next delivery. In addition to this it helps a mother to manage her life, economy, it helps her to have healthy child in future, it also helps to keep their health.

In your opinion who should make decisions regarding use of PP and PA family planning services? What types of decisions do women make regarding use of healthcare including PP and PA family planning services? What types of decisions regarding use of healthcare including PP and PA family planning services are made jointly?

R: Concerning the decision making mother should decide for herself. After the health care provider counsel her to use the service it is her right to decide. Our counseling matters a lot in order for her to come to decision. The decision is up to her. We don't have a right to interfere on her decision

While a mother is deciding she decides which family planning to use. By the way we counsel couples together sometimes. In our context males are dominant and females depend on their husbands decision. Therefore, joint decision is needed here. It is must for husband to discuss and come to decide. Both should discuss together with the help of health care provider.

I: In your community are women who delivered recently legally allowed to receive PP and PA family planning service?

- Is these same for boys and girls, people from different age groups, education background, ethnic groups, socio economic classes, excluded groups? How are they different? Why/ Why not?

R: Yes it is legally allowed. There is no forceful law that enforces them to use the services but voluntarily they can use the services. As far as the mother is volunteer to use the service she is legally allowed to use the service.

If they are adolescents come for abortion services her witness is enough to receive the services in my opinion, previously there should be other witnesses. The same is true to use family planning she can decide by her self.

I: How do recently delivered/aborted women access PP and PA family planning information and resource from the healthcare facilities?

- Is these same for boys and girls, people from different age groups, education background, ethnic groups, socio economic classes, excluded groups? How are they different? Why/ Why not?
- Do the people in your community support recently delivered/aborted women to access PP and PA family planning information and resources? How do they provide support? Who are these people? Why do they provide support?
- Are there certain groups in your community who do not support this? Who are these people? Why don't they support?

R: They should access PP and PA family planning information from the health center. They can also get from health development armies, from health extension workers, they can get from any sources.

Accessibility to information related to PP and PA family planning may differ because of difference in educational background, there are people living in remote areas, and near to main town. The awareness can differ from one another.

I: Do you feel they have a right to get healthcare information and resources including PP family planning services? Why/why not?

- Do you think women's experience is different from other people in your community? How? Please tell me more
- Is there legal mechanism you can follow to ensure they get quality healthcare information and resources they need? Please tell me more

R: All of the above mentioned people have the right to get health care information and resources including PP and PA family planning services. It is given for all women in child bearing age.We follow to ensure whether they get information during women conferences but there could be mothers who miss this information.

I: With this we have come to an end of the discussion. Do you want to add anything before we end the discussion or if you have any questions you want to ask?

R: In order to improve the access of PP and PA family planning services, stakeholders should support the service. In our case we have health development armies. It should be our priority to involve HDA's to improve the access, Health extension workers are very close to the community so they can easily get the community. Health extension workers received training on this in soddo. In addition to this we need to involve other stakeholders. In order to improve the quality of this services support especially equipments ,glove should be done from concerned bodies. Training support should also be done on immediate post partum family planning.

I: Thank you for your time and in case you want, you can get in touch with us through the details mentioned in the consent form.

R: thank you.

Summary

- ✓ There is scarcity of materials like gloves sanitation materials, soaps and other necessary services
- ✓ There are some religious groups which don't allow their followers not to use family planning
- ✓ On job training should be given for health workers including health extension workers and this should be also done for health development armies to increase their awareness
- ✓ It is better to work to ensure quality of the services(PPFP,PAFP)
- ✓ It is better to put F/P in the health posts for mothers who can't easily access.There is lack of the f/p methods in the health post
- ✓ There is a need from adolsents to use different services ,however there is no setup for them. For example they need counseling services, family planning services, counseling after birth. But we don't have conducive environment which attracts them to come

- ✓ Attention should be given in places where there are Mega projects b/c people from different places come, adolsents may be exposed to unintended pregnancies and associated problems
- ✓ Adolsent age by it self ,the behavior ,use of alcohol, intoxication can expose them to unwanted pregnancy
- ✓ concerned bodies have to play their role to fulfill and support with inputs needed for family planning services
- ✓ Awareness creation should be done at community level because once they have awareness they don't challenge us here while counseling. interventions should also be done in YFS
- ✓ Conducting women conference can bring change in improving the service.There still people who don't get service b/c of lack of information
- ✓ The re is challenge from the community side b/c the idea of giving PPFP is new they challenge and it doesn't give sense for them b/c they think about their pain, bleeding during labor.
- ✓ Fear is one of the challenge not to come to health center to use the service,b/c they hide them selves(specially adolescents PAFP services)
- ✓ Engaging female health workers and supportive female staffs can play role to enhance the use of PP and PA family planning services because they have awareness
- ✓ Because of male dominant culture women are influenced by their husband's decision

Discussion

Rapport Building

Thank you for proving consent to participate in the discussion. There are no right or wrong answers, please share your frank opinion, as it will help us in understanding the situation better.

Enquire about her recent delivery experience. An example is given below:

Please tell us about your recent childbirth experience. Where did you deliver? Who attended you during delivery? If you deliver at health facility how far is it from your home? How do you travel to the health facility?

Assets and Resources

Discussion

Now we are going to discuss about the Healthcare resources and assets available to recently delivered mothers in your area.

1. Health services (PP and PA family planning services) availability: Can you tell me the types of healthcare facilities that provide PP family planning services in your area?
   a. What types of FP services are available at the healthcare facilities?
   b. Are these services equally available to boys, girls, young, old, married, unmarried, people from different geographic locations, religious or cultural background, language etc.? Please tell me more

It is available at the health post and health center. It is possible to take from the private clinics as well. The types are pills, three months injection and three years “implanon”.

It is not available for young girls who want to use it. They are not eligible to use family planning since they are young. If she is not married, no one will give her at the health facilities. It is also not given for women who come from another place. If a woman come to her family for the first birth, she can use while returning back to her house.

2. Accessibility of the PP family planning services
   a. Can you tell me how far the PP family planning services providing facility is located? How long does it take for you to access the facility?
   b. What type of transportation means do you use to reach the facility? How easy or difficult is it to use the transportation services?
   c. Is there any cost associated with accessing the PP family planning services? Please tell me more about this
   d. Is the PP family planning service open at all time and is accessible with short waiting time at the healthcare facility? Are there types of FP methods of users’ choice in the healthcare facility? Do healthcare providers receive and treat users with respect and dignity?
   e. Are these services equally accessible to boys, girls, young, old, married, unmarried, people from different geographic locations, religious or cultural background, language etc.? Please tell me more
   f. How easy could you access the PP family planning service? Do you think your access to the PP family planning services is affected because of your background? How?

The health facility is very near to me and I have to walk about 2 minutes. It is free of charge and no cost is there. The service is available every day and I get what I wanted when I go there. But, I don’t know

about others. For me I get removed the one for three years one and immediately used depo. I got what I wanted. The health facility staffs care for me and give me the care properly. I don't think those who are not married can access the service. People from different religion can use it since they come from the same kebele.

3. Means to access the PP family planning services
   a. Please tell me about your household resources and assets. Please tell me the resources and assets belong to you.
   b. How is the use of the household resources and assets decided? Who makes the decision? Do you involve in the decision making? How do you think that affects your access to the healthcare services including PP family planning?
   c. Please tell me about your household income. Please tell me about your own income.
   d. How is the use of the household income decided? Who makes the decision? Do you involve in the decision making? How do you think that affects your access to the healthcare services including PP and PA family planning?
   e. In your community how is use of PP family planning seen? Is it acceptable for women to use PP family planning? Why/why not? Please tell me more

We are living as God allowed us to live. We have our own house, we have two children, he has some work at "ELPHA". We have cattle and a sheep. Decision is made jointly. We have to discuss first. We also discuss on using family planning. We have a shop and I help by working here as he work for "ELPHA". People see family planning "PP" as something good and bad. Some see it as good and others as bad thing. Those who say it bad say it sucks blood and causes health problems. They also say it is better to give as much birth as possible early than being late. Some people who say it is good say it helps to have a balanced family and it helps to balance family. Some old women say we have to use family planning because it nowadays the women become pregnant early after the birth. They say in their times women will stay for two or three years before they see mensus. So, they encourage us to use family planning.

4. In your opinion what should be done to improve access to the PP family planning services for all recently delivered mothers in your community?

Women should be taught in one to five and young women who are young and still fertile should attend such sessions. Health care workers should gather women and teach them.

Practices, Roles, and Participation

Now we are going to discuss about your women's and your role and responsibilities.

1. Please describe to me the typical women's roles in your community?
   - How are women and girls occupied over the course of 24 hours? Are there seasonal differences in how women and girls use their time?

The roles are watching over their children, feeding them, taking care of them, feeding breakfast and lunch. In our culture women clean the house, collect the grass, cook, clean, and other household duties. The activities are similar during summer and winter.

2. What kind of work do you do? Do these works change when your age or status changes (young, unmarried and married, older)?
3. Do you have barriers on your access? What barriers? How do the barriers/restrictions influence your access to the PP family planning services and supportive social networks?

- In your community do the restrictions/barriers change by social characteristic, such as (dis)ability, economic status, health status, educational level, religion, culture, geographical location, and marital status?

The barriers can be counseling from some people who discourage taking family planning. If someone advices it is as something bad, then people may be afraid to take it. Indeed, for those want to use it “FP”, there should be something like milk and sorghum in the house so that she will be able to take care of. She should be able to get some fluids to drink because it is an injection “drug” that causes burning sensation. I don’t think there is cultural and religious matters that can prevent women form using it.

4. Do you participate in any family, community, government, or other social networks organizations? Please tell me more.
   - Please tell me how your participation in these organizations affect your PP family planning services use

I participated in 1 to 5 on sanitation related issues. I came to this area recently and I didn’t participate in any conference still. But I still hear information from those who use family planning.

5. In your community do women assume leadership roles? What types of roles? Do these roles change by social characteristics, such as (dis)ability, economic status, health status, educational level, religion, culture, geographical location, and marital status?
   - Please tell us about your own experience with assuming any leadership roles?
   - Please tell me how your leadership roles affect your PP family planning services use

There are women who teach about sanitation and waste management. The mother in law is also a leader of 1 to 30 and they teach us about sanitation. I didn’t assume any leadership role.

6. In your community who decides at what age should people marry? What are the reasons for a women getting married at younger or older ages?
   - Please tell us about your own marriage experience? How old were you when you marry? What are the reasons you she get married at that age?
   - Please tell me how your age at marriage affects your PP family planning services use

The current generation decides by themselves. No one forces them and they chose their age of marriage. There are some who marry at younger age and other at older age. The reasons for early marriage can be beauty and the desire of young age to get in affairs.

I married when I was 18 years old. I married because of my luck. It is God’s gift and I married because God said so. I marriage also because I wanted to do so.

I started using FP after giving birth to the my first child and I took for three years and I gave birth to the next child.

7. What are your suggestions to improve women’s roles and participation to enhance the use of PP family planning services?

People should be educated and counseled about the advantages of using FP. Women should be counseled and get lessons about PP family planning.

<u>Knowledge, Beliefs, and Perceptions</u>

Now we are going to discuss about the issues faced by adolescents and women in accessing PP and PA FP services in your opinion.

1. In your community what is appropriate behavior for recently delivered woman regarding use of PP and PA family planning services? How do they differ from adolescent girls and young women?
    a. What is the appropriate behavior for a recently delivered mother? How do these believe influence health behavior including the PP family planning use?

People think women shouldn't use family planning immediately after birth since the body is weak and not strong yet. I believe women should consider using FP after six months or one year. It is me who decide until then.

2. What are the social beliefs and perceptions that share women's expectations and aspiration for PP family planning?
3. In your opinion who should make decisions regarding use of PP family planning services? What types of decisions do women make regarding use of healthcare including PP family planning services? What types of decisions regarding use of healthcare including PP family planning services are made jointly?

The decision should be made jointly. Everyone should agree within their family to use family planning. the best way is making a joint decision.

Legal Rights and Status

Now we are going to discuss about the marriage related process prevalent in this community.

1. In your community are women who delivered recently legally allowed to receive PP family planning service?
    - Is these same for boys and girls, people from different age groups, education background, ethnic groups, socio economic classes, excluded groups? How are they different? Why/ Why not?

    Yes, they are legally allowed. Young unmarried girls are not legally allowed to use FP.

2. How do recently delivered women access PP family planning information and resource from the healthcare facilities?
    - Is these same for boys and girls, people from different age groups, education background, ethnic groups, socio economic classes, excluded groups? How are they different? Why/ Why not?
    - Do the people in your community support recently delivered women to access PP family planning infatuation and resources? How do they provide support? Who are these people? Why do they provide support?
    - Are there certain groups in your community who do not support this? Who are these people? Why don't they support?

From the health post and from the health center. There are staff members there who provide information and services. There is no one in the community that supports or opposes using family planning.

3. Please tell me your experience with accessing healthcare information and resources including PP family planning.
    - Do you feel you have a right to get healthcare information and resources including PP family planning services? Why/why not?

- Do you think your experience is different from other people in your community? How? Please tell me more
- Is there legal mechanism you can follow to ensure you get quality healthcare information and resources your need? Please tell me more

Yes, I think it is my right to use infoamtion and resources. I took previously for three years “implanon ” and depo then before I give the current birth. If the will God, I would like to give birth after three or four years.

With this we have come to an end of the discussion. Do you want to add anything before we end the discussion or if do you have any questions you want to ask?

Thank you for your time and in case you want, you can get in touch with us through the details mentioned in the consent form.

****

Summary:

The interview was conducted at the house of the informant and it is about one month since she gave birth. She has also a retail shop at her house and in the middle of the interview she has to go and sell to her customers. The informant is somehow shy and I have to encourage her to open up her thoughts freely. Nonetheless, the women is still scared to talk much and answers in short. Finally, I explained to the informant about the possibility of pregnancy before she sees her first menses and to seek service and information form the health facilities.

Discussion

Rapport Building

Thank you for proving consent to participate in the discussion. There are no right or wrong answers, please share your frank opinion, as it will help us in understanding the situation better.

Enquire about her recent delivery experience. An example is given below:

Please tell us about your recent childbirth experience. Where did you deliver? Who attended you during delivery? If you deliver at health facility how far is it from your home? How do you travel to the health facility?

Assets and Resources

Discussion

Now we are going to discuss about the Healthcare resources and assets available to recently delivered mothers in your area.

1. Health services (PP and PA family planning services) availability: Can you tell me the types of healthcare facilities that provide PP family planning services in your area?
   a. What types of FP services are available at the healthcare facilities?
   b. Are these services equally available to boys, girls, young, old, married, unmarried, people from different geographic locations, religious or cultural background, language etc.? Please tell me more

Yes there are health facilities which provide family planning services. For example my wife used to take depo as family planning method which is useful for her health. There is family planning service and she utilizes it. As a family we use family planning methods. There are people who use the services and there are also other people who don't utilize the service. The service is available for all people who want to use it. It is there in the health center. s per my family, we use it for the wellbeing of the mother, the child and as a family as well. We utilize the service made available by the government. We previously used the service as well.

2. Accessibility of the PP family planning services
   a. Can you tell me how far the PP family planning services providing facility is located? How long does it take for you to access the facility?
   b. What type of transportation means do you use to reach the facility? How easy or difficult is it to use the transportation services?
   c. Is there any cost associated with accessing the PP family planning services? Please tell me more about this
   d. Is the PP family planning service open at all time and is accessible with short waiting time at the healthcare facility? Are there types of FP methods of users' choice in the healthcare facility? Do healthcare providers receive and treat users with respect and dignity?
   e. Are these services equally accessible to boys, girls, young, old, married, unmarried, people from different geographic locations, religious or cultural background, language etc.? Please tell me more
   f. How easy could you access the PP family planning service? Do you think your access to the PP family planning services is affected because of your background? How?

The distance from here to the health center is up to 20-25 minutes. If we use Bajaj it is not more than five minutes and it costs five birr. It is not that much far. We have access to vehicles. The cost can be affordable to some people and there are also people who can't afford. It is according to the individual family context. There are people who can afford, and not. If someone is healthy, it is near and anybody can simply walk. If is not more than 25 minutes. It is near. There is no cost associated with getting family planning in the health center. There are always heath care professionals working there to provide FP at the health center. Whenever she goes, she gets someone who gives the service. She gets family planning whenever she visits there. She never comes without receiving the family methods when she visits there. The type of family planning we use is depo and she gets it every three months she visits there. we didn't use another methods. The health care providers treat them respectfully. The give priority to pregnant mothers and those who want family planning services. As far as I know, they provide the service for the and they never send them back without providing the service. Even though boys or girls want to get the service they provide the services for anyone who wants the service. They don't discriminate based on age, rural/urban, or any religion. They provide the service for any Ethiopian who wants to use the service without checking the background. They provide the service there. They treat all who want the service respectfully. They gave postpartum family planning depo service for my wife after asking her willingness immediately after birth.

3. Means to access the PP family planning services
   a. Please tell me about your household resources and assets. Please tell me the resources and assets belong to you.
   b. How is the use of the household resources and assets decided? Who makes the decision? Do you involve in the decision making? How do you think that affects your access to the healthcare services including PP family planning?
   c. Please tell me about your household income. Please tell me about your own income.
   d. How is the use of the household income decided? Who makes the decision? Do you involve in the decision making? How do you think that affects your access to the healthcare services including PP and PA family planning?
   e. In your community how is use of PP family planning seen? Is it acceptable for women to use PP family planning? Why/why not? Please tell me more
4. In your opinion what should be done to improve access to the PP family planning services for all recently delivered mothers in your community?

My job is farming and I am a farmer. I work on my farm using different resources available in the locality. But recently I faced a road car accident that injured my leg. However, I work in shares with others duties beyond my capacity. I am not a merchant. I am a farmer. I help my wife in raising children, I raise small crops and seeds that can be sold to others easily. I produce tomato, cabbage and other crops if the rain condition is good. We also produce maize and teff. I have two cattle. I don't have sheep or goat. I don't have any other resource. I have small land that I grow grasses and trees as well. I am the one who decide on house hold resources and assets as I am the head of this household. Sometimes we discuss and pass the decision together. Regarding postpartum FP, we discuss together to use either the method that uses for three years of three months. We want a space between our children. we are using the PP family planning for that purpose. Since PP is free of charge availed by the government, we want to space between our children. we can't live like our father who want children every year. For example the elder one was born on April 2018 and after four years here is the newborn now. There is a gap between our children.

In our community most people view PP as something good. There are people who view PP family planning as good and also others who view as something bad. It is good for the children and for mothers

as well. when women use FP, they are more encouraged to give birth in the health facilities as well. In order to improve the service uptake in the community, there should be more education and awareness creation. Those who use the service should also increase others to use the service as well.

Practices, Roles, and Participation

Now we are going to discuss about your women's and your role and responsibilities.

1. Please describe to me the typical women's roles in your community?
   - How are women and girls occupied over the course of 24 hours? Are there seasonal differences in how women and girls use their time?

The typical roles of women in our community includes cooking, making coffee, raising children, feeding children, washing and cleaning the household and children, cleaning the cow dung, collecting grass for cattle, helping in the farming, receiving the relatives and in laws and serving them with respect, watching over the environment, and going to the market and coming back on time. Their roles include these. They also wash children, feed them; make their hairs and others related work as well.

2. What kind of work do you do? Do these works change when your age or status changes (young, unmarried and married, older)?

When young women grow in the family, there are still who support the family. It is not like the old times where girls and boys support too much. Now there are few children who support their families. Young ones help their parents by fetching water, by cleaning the house, collecting the grass, and going to the market place other areas their family want them to go. Older women does all the household activities.

3. Do you have barriers on your access? What barriers? How do the barriers/restrictions influence your access to the PP family planning services and supportive social networks?
   - In your community do the restrictions/barriers change by social characteristic, such as (dis)ability, economic status, health status, educational level, religion, culture, geographical location, and marital status?

We don't have any barriers. This is the service availed for the help of our generation by the government. It is free of charge. Even if we pay for the service, we will benefit a lot from it. People from every religion, area, educational status use the service. There are no barriers to use the service whether someone is married or not, from rural or urban areas, has disability or not. The service is available for anyone who want the service.

4. Do you participate in any family, community, government, or other social networks organizations? Please tell me more.
   - Please tell me how your participation in these organizations affect your PP family planning services use

Yes women participate in different gathering when they are called by someone like government. When they participate in such gatherings, they share information about different issues. It also affects their use of PP family planning service.

5. In your community do women assume leadership roles? What types of roles? Do these roles change by social characteristics, such as (dis)ability, economic status, health status, educational level, religion, culture, geographical location, and marital status?
   - Please tell us about your own experience with assuming any leadership roles?
   - Please tell me how your leadership roles affect your PP family planning services use

In our community there are some women chosen by the government to lead others. There are also women who lead in every "ketenas" as women development army leaders. They are the ones who

mobilize others to go and get the service. Women leaders mobilize from each locality and bring others to the health post. It doesn't change by social characteristics. My wife may assume leadership roles in the future if chosen.

6. In your community who decides at what age should people marry? What are the reasons for a women getting married at younger or older ages?
   - Please tell us about your own marriage experience? How old were you when you marry? What are the reasons you she get married at that age?
   - Please tell me how your age at marriage affects your PP family planning services use

There are different ways women get married. Sometimes they arrange and marry by making the decision by them. Sometimes others trick them to marry. Young girls leave their education or business and just get married by their decision. They forget their future plans and get married. When I marry her, she was 19 years old. I was 23 years old when we get married. She wanted to marry me, others wanted to abduct her. That is why I decided to marry her as quickly as possible. We immediately started using PP family planning after the birth of a child.

7. What are your suggestions to improve women's roles and participation to enhance the use of PP family planning services?

We should tell others to take PP family planning. Both men and women should tell others about the benefit of using FP. Even when someone faces abortion, we should tell them jut to consider using the service that is freely given by the government.

Knowledge, Beliefs, and Perceptions

Now we are going to discuss about the issues faced by adolescents and women in accessing PP and PA FP services in your opinion.

1. In your community what is appropriate behavior for recently delivered woman regarding use of PP and PA family planning services? How do they differ from adolescent girls and young women?
   a. What is the appropriate behavior for a recently delivered mother? How do these believe influence health behavior including the PP family planning use?

In our community the behavior is mixed. There are people who chose to go to the health facility and there are also others who don't want to go. Health care providers and health extension workers advice in every locality people should use PP family planning. But, the response is mixed. For my case, I am 100% happy to use the PP service. I am not sure about others. They community perception of appropriate behavior regarding PP family planning is mixed.

2. What are the social beliefs and perceptions that share women's expectations and aspiration for PP family planning?
3. In your opinion who should make decisions regarding use of PP family planning services? What types of decisions do women make regarding use of healthcare including PP family planning services? What types of decisions regarding use of healthcare including PP family planning services are made jointly?

I should be the one who pass the decision in my house. Since I am the father and husband of my wife, I am primarily responsible to make the decisions regarding PP family planning. First I have to know and teach my wife about the health benefits to the mother and child by using PP family planning. I have to teach her that having space between children is helpful to prevent diseases and malnutrition. Then after we agree and make a joint decision. I have to tell her what is right.

Legal Rights and Status

Now we are going to discuss about the marriage related process prevalent in this community.

1. In your community are women who delivered recently legally allowed to receive PP family planning service?
   - Is these same for boys and girls, people from different age groups, education background, ethnic groups, socio economic classes, excluded groups? How are they different? Why/ Why not?

They are legally allowed and encouraged to receive PP family planning service. Is the same for boys and girls, people from any age groups? It is legally allowed to any one irrespective of educational, ethnic or other backgrounds.

2. How do recently delivered women access PP family planning information and resource from the healthcare facilities?
   - Is these same for boys and girls, people from different age groups, education background, ethnic groups, socio economic classes, excluded groups? How are they different? Why/ Why not?
   - Do the people in your community support recently delivered women to access PP family planning infatuation and resources? How do they provide support? Who are these people? Why do they provide support?
   - Are there certain groups in your community who do not support this? Who are these people? Why don't they support?

Recently delivered women get access to PP family planning information and resource from health care providers. Mothers get the first information from them to utilize the service. Yes, they teach all people you mentioned including different age and economic categories. There are some people who encourage us to use the service. Women development army leaders mobilize people to use the service. There are no groups in the community who don't go against this service. I don't know about other areas, but here we don't have certain group who don't support this service.

3. Please tell me your experience with accessing healthcare information and resources including PP family planning.
   - Do you feel you have a right to get healthcare information and resources including PP family planning services? Why/why not?
   - Do you think your experience is different from other people in your community? How? Please tell me more
   - Is there legal mechanism you can follow to ensure you get quality healthcare information and resources your need? Please tell me more

Yes, I have access to the information and use the service. I have been also using PP family planning since the first child. I feel also the right to use the service and get access to the information. There are people who also use the service in a similar way. I have all the right to use the service whenever I want.

With this we have come to an end of the discussion. Do you want to add anything before we end the discussion or if do you have any questions you want to ask?

Thank you for your time and in case you want, you can get in touch with us through the details mentioned in the consent form.

****

Discussion

Rapport Building

Thank you for proving consent to participate in the discussion. There are no right or wrong answers, please share your frank opinion, as it will help us in understanding the situation better.

Enquire about her recent delivery experience. An example is given below:

Please tell us about family planning service in your woreda. Where did women get family planning services? Who provided the services? How far is the health facility from women's home (in different directions? How do women travel to the health facility?

Assets and Resources

Discussion

Now we are going to discuss about the Healthcare resources and assets available for provision of PP and PA family planning services in your area.

1. Health services (PP and PA family planning services) availability: Can you tell me the types of healthcare facilities that provide PP and PA family planning services in your area?
   a. What types of FP services are available at the healthcare facilities?
   b. Are these services equally available to boys, girls, young, old, married, unmarried, people from different geographic locations, religious or cultural background, language etc.? Please tell me more

There are different family planning service we are providing. We give depo as well. some time ago there was stock out of depo, but now we have it. Most of the time now people want to take implanon. And also people us jaddele contraception for five years. Most of the time women feel discomfort with IUCD. They feed ashamed to uncover themselves and be seen to insert it. Even after being of coach they change their mind and avoid taking it. Most of the time IUCD is the most difficult one To convince and provide to women. we teach mothers on women conference and provide them implanon and jaddele by taking sterile materials from here. Previously they prefer depo or pills, but now they want implanon or jaddele. There was a time when we had no depo, then after women started using implannon and jaddele willingly.

After delivery, even though women want to take the service, husbands disagree. People from Burkuto and Kontola give birth to nine or ten babies while they are too weak and poor. In such case, we bring husbands and counsel them to take FP methods. If they agree, we provide FP for three years or fir five years. When they come to receive the service, we provide for everyone. Everyone from urban or rural receive the service. After the delivery, most of the time they are not willing. They want to take the service after six months. But when we see people who are too weak uterus after giving birth, we counsel them and they are wiling the take the PP service.

2. Accessibility of the PP and PA family planning services
   a. Can you tell me how far the PP and PA family planning services providing facilities are located? How long does it take for women to access the facilities?
   b. What type of transportation means do they use to reach the facilities? How easy or difficult is it to use the transportation services?

c. Is there any cost associated with accessing the PP and PA family planning services? Please tell me more about this
d. Is the PP and PA family planning service is accessible at all time at the healthcare facilities? Are there types of FP methods of users' choice in the healthcare facilities?
e. Are these services equally accessible to boys, girls, young, old, married, unmarried, people from different geographic locations, religious or cultural background, language etc.? Please tell me more
f. How easy could they access the PP and PA family planning services? Do you think that their access to the PP and PA family planning services are affected because of their background? How?

Accessible kebeles to the facilities are Buge, Shaki Soshone. Others like Harto Kontola and Damot buge are somehow far to the health center. They sometimes use motorcycle and others who cannot afford come by foot. When they get difficulty to come here due to transportation, they take depo from the health post there. but, those who want jaddele or implanon have to pay for motor and come here to get the service. Health posts have no sterile materials to receive jaddle or implannon. Regarding the cost of services for the FP and other materials live gloves for that, it is free of charge given by the government. Sometimes, when we have no gloves, they have to buy that. Any service regarding FP/PP service is free of charge. We provide the service based on shifting. The challenge in our facility not to provide 24 hours service is that we don't use BPR schedules. For example one provider come today and stays 24 hours handling all the services in the MCH including FP, ANC and delivery. When providing PP and PA services, Sometimes they complain about jaddele and implanon and prefer depo. They say if we use those methods, even after removing them we couldn't conceive for years. And those who use implannon complain of rush and pain around their arm. They complain of difficulty of removing placenta if they use family planning. Besides, anyone can get the service from us.

3. Means to access the PP and PA family planning services
a. Please tell me about your facilities' resources and assets. Please tell me the resources and assets belong to these health facilities to provide PP and PA FP service.
b. How is the use of the facility/Woreda health office resources and assets decided? Who makes the decision? Do you involve in the decision making? How do you think that affects access to the healthcare services including PP and PA family planning by women?
c. Please tell me about income of in this Woreda. Please tell me about your woredas income.
d. How is the use of the woreda's income decided? Who makes the decision? Do you involve in the decision making? How do you think that affects their access to the healthcare services including PP and PA family planning?
e. In your woreda how is use of PP and PA family planning seen? Is it acceptable for women to use PP and PA family planning? Why/why not? Please tell me more (what about boys and girls)
4. In your opinion what should be done to improve access to the PP and PA family planning services for all women in your community?

After PP we have always implannon, jaddle, depo and IUCD. Whenever they want the service, we simply provide them. We don't have scarcity of the service. We have also enough space for that. When they stay here after delivery, we counsel them and they take the service. What matters is our approach. If we gently approach them and counsel them, they take the service immediately. During post natal period, it is easy to give them the service. If we approach them well, they even tell a secrete that the husband needs counseling and should agree to take the service. After that we counsel the husbands and they usually agree. Most of the time "primis" are not willing to take PP family planning because they want more children. Health facilities for mothers are preserved for them only. Even if we take training like cervical cancer, they arrange another room for that service. Health center heads are the ones who

decide on the allocation of resources. People from Buge area are good economically. Those who come from Burqito and Qontola are relatively poor. They have a lot of problems including transportation. The income for the health center is based on the health insurance. All the required inputs come from PFSA for MCH services. They are free. Other supplies are from the Woreda health office. In the health center management, as the case team management leader we participate.

Regarding the community view of PP, many women who come accompanying the delivering mother listen to our advice and agree with what we advise. They say what the health care providers telling about family planning is true. They even say some of their friends are pregnant where the other child is in taking malnutrition treatment milk at the health center. Most women believe taking FP is something good. Some women secretly take the PP family planning without the awareness of their husbands. Others are not that much interested to take FP. But nowadays most women come on market days and take PP if the husbands don't agree. The young boys and girls who come here seeking abortion care are afraid of getting the service. They ask for depo whilst being pregnant. They stand shy and speak slowly through the window. They thought taking depo would help them to have abortion. During such times we provide them meso and give the abortion service after reassuring them and checking the age of the pregnancy. Sometimes they come lately after four or six months. In such times we give them abortion service. Young boys also some when their partner get pregnant. They also get the service for their partner. We give the service and give them counseling as well. young boys also take condoms from the health facility even though they are afraid of being seen. In order to improve the service provision, those in the OPD and laboratory should also get the training. Those who go to the field work should be trained and they will link those who want the service with us. so, the best way is giving the training for all health care providers in the facilities. Engender heath gave the taraining for some of the health care providers, but those who go to the sites should be trained to increase access to PP and PA family planning service. While they are going to the community to build toilets, they can also get those who should get PP and PA services.

Practices, Roles, and Participation

Now we are going to discuss about your role and responsibilities.

1. Please describe to me the typical Woreda health office officials/experts roles in your Woreda/community?
    a. How is Woreda health office officials occupied over the course of the day? Are there seasonal differences (campaign/political assignment) in how you use time?

They typical role of the MCH coordinator is to check and monitor all the recording of maternal and child health services. There are a lot of booklets that needs to be filled in MCH department. So, the role should also include checking that. And also checking everyday activities in the MCH rooms. They check whether the materials are sterile or not. Adjusting the program and follow up of the service is also part of the role. The MCH coordinator also schedules field visits and provision of the service. The coordinator should also check whether supplies are ready for services like PP and PA family planning. They also make accountable those who don't adjust the work and do properly. There are cases where one service is overwhelmed by a lot of activities since a single provider is supposed to provide all the services. It would be better if there are enough number of health care providers and taking care of other women who come seeking the service. There are times women go without getting PP or other family planning services because the provider is occupied by providing the delivery service. We hear such complains and it should be improved. This happens because most of the providers are from the urban areas and they

have to adjust to the shortage of health care providers in the facility. If someone works for 24 hours, they have to be free for 24 hours while the next one is giving the service. Shortage of the care provider is the main challenge in our facility.

2. As Woreda health office official, what kind of work do you do? Do these works different when facility status changes (Rural, urban and geographical locations)?
3. Do Woreda health office officials like you have restrictions/barriers on the accessibility? What restrictions/barriers? How do the restrictions/barriers influence the access of the PP and PA family planning services and supportive social networks?
   a. In your Woreda do the restriction/barriers change by social characteristic, such as (dis)ability, economic status, health status, educational level, religion, culture, geographical location, and marital status?

Sometimes the behavior of the service provider can be a limitation or barrier by itself. Peoples behavior varies according to his personality. Sometimes they are not happy to be treated by some health care providers. For example there was a women who came from Burqito seeking removal of implanon service, but the health care provider gives appointment to another week, then they will not be happy to come and get the service from the facility. This can be one barrier for PP family planning. There are such cases from the health care providers. We don't have restrictions regarding religion. They are happy because they say the service is given freely by the government. There are women who said lack of services previously harmed their uterus and health as well. They encourage others to take PP family planning services. They even bring those women who are not willing take PP family planning after teaching and convincing them to take the service. Regarding religion, young girls those who became pregnant are so scared to get the service and worried so much what to do. But we reassure their privacy and give them the service. Regarding the geography sometimes it becomes as barrier for those who come from far kebeles. They complain of distance for not using the PP family planning. Those with disability like mental health problem and have broken limb also come and use the service. Those who can't hear and speak also bring someone with them and use the service. But still there are men who prevent women from using PP family planning even after providing counseling. Sometimes they change their mind and us the PP family planning.

4. Do you participate in any meetings, workshops, trainings, pregnant women conference, family, community, government, or other social networks organizations? Please tell me more.
   a. Please tell me how your participation in these organizations affect the PP and PA family planning services provision

I took a training on different topics. Previously we didn't take abortion care training and we simply send women to the referral without giving basic care. Now we give them basic care and send to the referral after cleaning and securing IV lines. Now we took training and provide abortion care in our facility. Training helped us also to give counseling and provide PP and PA services as well.

5. In your Woreda do women assume leadership roles? What types of roles? Do these roles change by social characteristics, such as (dis)ability, economic status, health status, educational level, religion, culture, geographical location, and marital status?
   a. Please tell us about your own experience with assuming any leadership roles?
   b. Please tell me how your leadership roles affect the PP and PA family planning services provision

Women assume leadership role in the health center. For example the head of our health center is also a female. There are also women at the Woreda level leadership. Leadership is a challenging task. Regarding PP and PA the leadership role is not that much visible. Rather for PP and PA health extension workers and HDAs roles is too much. Their role is so helpful for us than other leaders in the community.

6. In your Woreda/community who decides at what age people marry? What are the reasons for getting married at younger or older ages in this Woreda?
   a. Please tell me how their age at marriage affects their PP and PA family planning services use

Most of the time young girls from Burqito and Qontola marry at younger age and come seeking delivery care. When we ask a mother who is deciding they say the decision is made by the girls and they are not involved. When we ask them further, they say since they are living in the border areas with Hadiya zone, they believe it is a shame for girls not to marry at early age. So, it is because of that girls marry early there. Most of the time girls decide by themselves and sometimes mothers advise them to marry as early as possible. Some women bring their young girls who gave birth recently to take PP services while bringing them for child vaccination.

7. What are your suggestions to improve women's roles and participation to enhance the use of PP and PA family planning services?

<u>Knowledge, Beliefs, and Perceptions</u>

Now we are going to discuss about the issues faced by women in accessing PP and PA family Planning services in your opinion.

1. In your Woreda what is appropriate behavior for a woman after delivery and abortion regarding the use of PP and PA family planning? How do they differ from adolescent girls and young women?
   a. How do these believe influence health behavior including the PP and PA family planning use?

Sometimes women who faced abortion including young girls take implannon immediately because of the danger of being pregnant again. Young girls are afraid to come to the health facility and go to private facilities seeking FP service. They pay 40 birr to pay depo. They are afraid of being seen by someone on the facility.

2. What are the social beliefs and perceptions that share women's expectations and aspiration for health care including PP and PA family planning?
3. In your opinion who should make decisions regarding use of PP and PA family planning services? What types of decisions do women make regarding use of healthcare including PP and PA family planning services? What types of decisions regarding use of healthcare including PP and PA family planning services are made jointly?

Women should make the decision regarding PP and PA family planning. Women make the decision regarding PP family planning because they think they were the ones who bleed and suffer during and after delivery.

Legal Rights and Status

Now we are going to discuss about the marriage related process prevalent in this community.

1. In your community are women who delivered recently legally allowed to receive PP and PA family planning service?
   - Is these same for boys and girls, people from different age groups, education background, ethnic groups, socio economic classes, excluded groups? How are they different? Why/ Why not?

There are legally allowed to receive PP and PP family planning. There are also no discriminations legally to receive the service for any one you mentioned. But young boys and girls are sometimes afraid of the confidentiality. Because they think we may tell to their family members regarding use of the service, they are ashamed to come freely.

2. How do recently delivered/aborted women access PP and PA family planning information and resource from the healthcare facilities?
   - Is these same for boys and girls, people from different age groups, education background, ethnic groups, socio economic classes, excluded groups? How are they different? Why/ Why not?
   - Do the people in your community support recently delivered/aborted women to access PP and PA family planning information and resources? How do they provide support? Who are these people? Why do they provide support?
   - Are there certain groups in your community who do not support this? Who are these people? Why don't they support?

Yes recently delivered women access and use PP and Pa in our facility. Young boys and girls are afraid and ashamed fearing to stereotyping by the health facility staff members. There are no groups who prevent them from using the service.

3. Please tell me women's experience with accessing healthcare information and resources including PP family planning.
   - Do you feel they have a right to get healthcare information and resources including PP family planning services? Why/why not?
   - Do you think women's experience is different from other people in your community? How? Please tell me more
   - Is there legal mechanism you can follow to ensure they get quality healthcare information and resources they need? Please tell me more

Women freely access healthcare information including PP family planning from our facility. Women also get information from one another. When someone gets one service, the other women come by listening her friends advice. There is frequent meeting with mothers in our department and we evaluate each other when services are not provided properly. There is a means of sharing information and evaluating others incase services are not delivered properly.

With this we have come to an end of the discussion. Do you want to add anything before we end the discussion or if you have any questions you want to ask?

Thank you for your time and in case you want, you can get in touch with us through the details mentioned in the consent form.

****

In-Depth Interview conducted with recently delivered woman at SHC by Degnesh Dawit

Sociodemographic information of interviewee

Code	Keble	age	sex	Educational-status	Job-status	Number of children
IDI_SHC_Wo_230622	Golla	28	F	secondary	housewife	two

Assets and Resources

I; Health services (PP family planning services) availability: Can you tell me the types of healthcare facilities that provide PP family planning services in your area?

R; in my opinion those services have been given in only health centers even they may not given in hospital

I; what types of FP services are available at the healthcare facilities?

R; I know that 5 years implant is given here in health center but there is no Dipo, in addition to that other than Dipo there is all kinds of family planning methods exists here in health center and they give to people as their need.

I; Are these services equally available to boys, girls, young, old, married, unmarried, people from different geographic locations, religious or cultural background, language etc.? Please tell me more

R; I don't know about others but I know only about mine, whether there is service for all regardless of their difference, I have no idea about this, but I know something about it is there is FP service for those who are married and gave birth.

Accessibility of the PP family planning services

I; Can you tell me how far the PP family planning services providing facility is located? How long does it take you to access the facility?

R; For me it's not that much far to come here (SHC), and since our village is around Lewi it takes almost 5-6 minutes by Bajaj transport it is also not far for us even if we come through walking.

I; what type of transportation means do you use to reach the facility? How easy or difficult is it to use the transportation services?

R; to come to this health center we use Bajaj transport, getting Bajaj to come here is not difficult

I; is there any cost associated with accessing the PP family planning services? Please tell me more about this

R; I can get the service freely there is no associated cost as I know.

I; is the PP family planning service open at all time and is accessible with short waiting time at the healthcare facility?

R; I get always open when I come this health center to get the service.

I; Are there types of FP methods of users' choice in the healthcare facility?

R; they give me according to my interest, they respect my need whether I need one year's, three years or five years family planning method, when I need withdrawal they remove it when the removal date reach.

I; Do healthcare providers receive and treat users with respect and dignity?

R; yes they served me with respect when I come here to get the service, so I have no complain regarding these.

I; Are these services equally accessible to boys, girls, young, old, married, unmarried, people from different geographic locations, religious or cultural background, language etc.? Please tell me more

R; I don't know whether there is services accessible for boys or not, I know that the service is accessible to married woman.

I; How easy could you access the PP family planning service? Do you think your access to the PP family planning services is affected because of your background? How?

R; they served me equally with others at that time, not showed different face for me

1. Means to access the PP family planning services

I; Please tell me about your household resources and assets. Please tell me the resources and assets belong to you.

R; in our home thanks to God we have all assets and resources which is important for our life, like Bajaj, house household utensils and cattle, there is no difference between him and me so all things we have is for both and we have no things in separate, his means mine and mine means his.

I; how is the use of the household resources and assets decided? Who makes the decision? Do you involve in the decision-making?

R; the household resource and assets decided after discussing together about the issue and we both decide upon it

R; How do you think that affects your access to the healthcare services including PP family planning?

R; it had great contribution to access any kind of family planning methods which is comfortable for me, He is also volunteer in case of family planning methods

I; Please tell me about your household income. Please tell me about your own income.

R; our source of income is milk from the cattle and from the Bajaj, we can get around 6000-7000 birr per month, I have no my own all of it is for both of us (me and my husband)

I; in your community how is use of PP family planning seen? Is it acceptable for women to use PP family planning? Why/why not? Please tell me more

R; in my community people see and accept it as important thing no one is opposing it, because u can see the time it is very difficult to grow up children in this year, life is too expensive now, that's why government also teaching us to use it.

I; In your opinion what should be done to improve access to the PP family planning services for all recently delivered mothers in your community?

R; for example in these health center there is no three months injection type or Depo they give long term family planning, but many people prefer 3 months injection type so it is better if they avail it and provide it to those who have interest on it, if they do that many people can be benefited from it.

Practices, Roles, and Participation

Now we are going to discuss about your women's and your role and responsibilities.

I; Please describe to me the typical women's roles in your community?

R; some of women engaged in business activity in addition to household activity in my community

I; how are women and girls occupied over the course of 24 hours?

R; preparing food for children and husband, collecting grasses for cattle, Eviction of their dung and caring of their children, but for girls there is not that much workload because they pass most of their time at school after that they may do their homework and read, then they help their parents by preparing food and cleaning the house

I; Are there seasonal differences in how women and girls use their time?

R; yes it may change with season because we are farmer so the farming activity is more of loaded during summer season

I; What kind of work do you do? Do these works change when your age or status changes (young, unmarried and married, older)?

R; my work is more of household activities, but when I become aged I planned to open mini market like shop which goes with my capacity.

I; Do you have barriers on your access? What barriers? How do the barriers/restrictions influence your access to the PP family planning services and supportive social networks?

R; for me there is no barriers to use pp family planning methods

I; Do you participate in any family, community, government, or other social networks organizations? Please tell me more.

R; yes I do participation in many aspects of social networks

I; Please tell me how your participation in these organizations affect your PP family planning services use

R; my participation in social networks used me to get knowledge from different source

I; In your community do women assume leadership roles? What types of roles? Do these roles change by social characteristics, such as (dis)ability, economic status, health status, educational level, religion, culture, geographical location, and marital status?

R; yes I know woman assumed as leader in Idir place, she assumed due to her educational status and based on her leadership ability, ability to read and write

I; Please tell us about your own experience with assuming any leadership roles?

R; Personally I have no experience regarding leadership role

I; In your community who decides at what age should people marry?

R; persons who going to marry will decide their age to marry

I; what are the reasons for a women getting married at younger or older ages?

R; both of their interest and decision matters, most of the time girls looks about does he can support our home through money, by considering this they decide to marry

I; Please tell us about your own marriage experience? How old were you when you marry?

R; I was 25 years old when I got marriage

I; What are the reasons you she get married at that age?

R; I had plan to marry after being independent that was the reason for me to marry at that age

I; Please tell me how your age at marriage affects your PP family planning services use

R; within one year of marriage I gave birth and stayed for three years by using three years FP methods then I removed it and become pregnant again as u are looking this is my second child

I; What are your suggestions to improve women's roles and participation to enhance the use of PP family planning services?

R; health professionals should teach all women about advantage of PPFP methods and encourage them to use it.

Knowledge, Beliefs, and Perceptions

I; In your community what is appropriate behavior for recently delivered woman regarding use of PP and PA family planning services? How do they differ from adolescent girls and young women?

I; the perception of all woman is different some of them accept it as good thing and others seen it negatively, personally I believe on getting family planning method after 45 days of delivery, I think others also think like me , but taking it immediately is difficult through pain, for those not delivered recently it is easy to access it because they are at normal condition.

I; what is the appropriate behavior for a recently delivered mother? How do these believe influence health behavior including the PP family planning use?

R; they think that they should stay at home for at least 45 days without getting out of home, they also not encouraged to go to health center to get family planning methods before that days, so this may affect getting of family planning methods within specific period.

I; what are the social beliefs and perceptions that share women's expectations and aspiration for PP family planning?

R; they think that if they have economy they should give birth without staying too much even within in one year, but those who face difficulties to grow their children should receive it immediately after 45 days of delivery.

I; In your opinion who should make decisions regarding use of PP family planning services?

R; they both husband and wife should discuss on the issue and decide together.

I; what types of decisions do women make regarding use of healthcare including PP family planning services?

R; most of the time they decide to take family planning methods after 45 days of delivery when they take their child to receive first vaccine.

I; What types of decisions regarding use of healthcare including PP family planning services are made jointly?

R; they both decide to receive three years family planning methods when she come to receive first vaccine to her child

Legal Rights and Status

I; In your community, are women who delivered recently legally allowed to receive PP family planning service?

R; yes because if it is not allowed legally they may not give us so yes its legally allowed.

I; Is these same for boys and girls, people from different age groups, education background, ethnic groups, socio economic classes, excluded groups? How are they different? Why/ Why not?

R; yes it is the same for all

I; How do recently delivered women access PP family planning information and resource from the healthcare facilities?

R; she may get the information regarding PP from health center after delivery, they counsel us to receive it after delivery

I; Is these same for boys and girls, people from different age groups, education background, ethnic groups, socio economic classes, excluded groups? How are they different? Why/ Why not?

R; yes it is similar for all

I; Do the people in your community support recently delivered women to access PP family planning infatuation and resources? How do they provide support? Who are these people? Why do they provide support?

R; some of women in our community recommends us to receive it recently, they ask us whether we received it or not, if not they advices us to receive it because they received it before and knows its advantage

I; Are there certain groups in your community who do not support this? Who are these people? Why don’t they support?

R; in my community no one is refusing it

I; Please tell me your experience with accessing healthcare information and resources including PP family planning.

R; I can get the information and resources regarding PP from health center during delivery time, from the other person and from the church

I; Do you feel you have a right to get healthcare information and resources including PP family planning services? Why/why not?

R; yes I feel I have right to get healthcare information and resources including PP family planning services when I need, since I m a human being

I; Do you think your experience is different from other people in your community? How? Please tell me more

R; mine is also similar with others

I; Is there legal mechanism you can follow to ensure you get quality healthcare information and resources your need? Please tell me more

R; yes if they don’t give me quality information and resources I can inform to upper body from them

Summary

The interview conducted with recently delivered woman at Sodo health center at 23/06/22. An educational status of interviewee was secondary, she learned up to grade 10. Her husband is farmer and she is housewife. During interview time, we met her when she came to health center to receive forty-five days vaccination for her child. Despite she learned up to grade 10 she has poor communication skill and even she faces challenge to understand what I am asking her, to solve such issue I am just repeating the question and tried to catch the answer. At that time, she

is also hurry to go her home back, and she has no sufficient knowledge related to postpartum family planning. In general, information I got from her is not rich/ sufficient.

Key findings:- the decision to use fp should be made between wife and husband

Recently delivered human in my community prefer to use family planning method after 45 days of delivery rather than immediately

Discussion

Rapport Building

Thank you for proving consent to participate in the discussion. There are no right or wrong answers, please share your frank opinion, as it will help us in understanding the situation better.

Enquire about her recent delivery experience. An example is given below:

Please tell us about family planning service in your woreda. Where did women get family planning services? Who provided the services? How far is the health facility from women's home (in different directions? How do women travel to the health facility?

Assets and Resources

Discussion

Now we are going to discuss about the Healthcare resources and assets available for provision of PP and PA family planning services in your area.

1. Health services (PP and PA family planning services) availability: Can you tell me the types of healthcare facilities that provide PP and PA family planning services in your area?
   a. What types of FP services are available at the healthcare facilities?
   b. Are these services equally available to boys, girls, young, old, married, unmarried, people from different geographic locations, religious or cultural background, language etc.? Please tell me more

Normally in our health center we provide PP and PA family planning services. at the community level health posts provide the service. The HEW there also provide the service. In our cluster PHC includes health post and health center. The hospital nearby is new and didn't start providing the service. We provide long acting and short acting. Long acting includes jaddle, implanon and the short acting ones are pills and depo. Another one is about IUCD and even though we have a trained staff we have a shortage of equipment. We don't have a complete set for IUCD.

Yes, the service is available for all who want it. The main thing is counseling and when someone comes we tell them the options and the choice they made will be served. If we don't have their choice, we tell them what is available and they will choose from the options.

2. Accessibility of the PP and PA family planning services
   a. Can you tell me how far the PP and PA family planning services providing facilities are located? How long does it take for women to access the facilities?
   b. What type of transportation means do they use to reach the facilities? How easy or difficult is it to use the transportation services?
   c. Is there any cost associated with accessing the PP and PA family planning services? Please tell me more about this
   d. Is the PP and PA family planning service is accessible at all time at the healthcare facilities? Are there types of FP methods of users' choice in the healthcare facilities?
   e. Are these services equally accessible to boys, girls, young, old, married, unmarried, people from different geographic locations, religious or cultural background, language etc.? Please tell me more
   f. How easy could they access the PP and PA family planning services? Do you think that their access to the PP and PA family planning services are affected because of their background? How?

Normally the distance is according to the clusters. The remotest areas include two kebeles. They are addressed by a back-up service from the outreach services in addition to the HEW work. The distance is not too far, but the topography is difficult. The furthest kebele is Anka shashara and Tora sadebo. The main challenge is topography than the distance. If they want to use motorcycle it may take up to 300 birr for one trip. These people have access at the health post, but if they want to come here, it will take that amount. But they have access there at the health post. There is no cost associated with the service and it is fully free.

At the community level there is sometimes shortage of the trained HEWs on long acting FP. In some areas there is shortage of the trained ones. There are such problems in our area.

If the community is not accessing the service at the health post, they will be referred to the health center here and they will get the service from here. And it is accessible for everyone who needs the servce. Anyone who wants to use the service can get it. It is accessible for everyone. For males who want the service such as vasectomy, we inform and can give the such service after counseling.

Normally compared to other FP types, immediate PP family planning is not utilized well. if they don't get counseling during ANC visits, it would be difficult to provide after delivery. So, mothers who didn't follow ANC services are not willing to use PP family planning. so, counseling is needed especially for rural people. Urban women are relatively better to accept PP family planning. usually rural women are less happy to use PP family planning.

3. Means to access the PP and PA family planning services
   a. Please tell me about your facilities' resources and assets. Please tell me the resources and assets belong to these health facilities to provide PP and PA FP service.
   b. How is the use of the facility/Woreda health office resources and assets decided? Who makes the decision? Do you involve in the decision making? How do you think that affects access to the healthcare services including PP and PA family planning by women?
   c. Please tell me about income of in this Woreda. Please tell me about your woredas income.
   d. How is the use of the woreda's income decided? Who makes the decision? Do you involve in the decision making? How do you think that affects their access to the healthcare services including PP and PA family planning?
   e. In your woreda how is use of PP and PA family planning seen? Is it acceptable for women to use PP and PA family planning? Why/why not? Please tell me more (what about boys and girls)

In recent times we have problems related with FP supplies. We have to wait for three or four months to get some methods of FP. There is shortage from the supplying offices. We have shortages of implanon and depo sometimes. And also we have shortage of surgical gauze and surgical blade. These supplies are the main challenges we have. To provide mobile services we have also transportation and equipment set that help to provide the service.

We get the necessary resource from health insurance budget and other programs that support FP services. Regarding FP all supplies are provided from the programs. The rooms are managed by the health center and construction issues are managed by the woreda admiration. The health center management manages the available rooms we have. Regarding income it is as I have told "from health insurance".

We need a good income to avail the necessary resources. After CBHI we don't have any income apart from it. There is no other budget support from the gov't. if someone is not included in CBHI, he will pay for that. Generally the income we get is too small. Because of that we can't afford to buy some basic things like guaze and surgical blade. So, there is a big problem related with income.

PP and PA are seen not as acceptable as other types of family planning. It is something new and there is low level of understanding and perception form the community. Some women who have repeated abortion and those with shortly spaced births are counseled to provide the service. But the perception is new and not yet developed regarding PP and PA. But if counseled well, they can accept it. For boys and girls, if they are not married, it will be challenging to use it. If a girl is using family planning without marrying there is discrimination from the community. If someone is using FP before marriage it is strange in the community, rather commercial sex workers use such services.

4. In your opinion what should be done to improve access to the PP and PA family planning services for all women in your community?

To improve access I think giving a good counseling service during ANC would help. She should be counseled about the next birth by the health care providers. At the community level, strengthening early counseling and referring them to the health facilities would help. I think counseling husbands should be given as well. providing the supplies on time is also important. In addition, providing the service at the community level will also improve the access.

Practices, Roles, and Participation

Now we are going to discuss about your role and responsibilities.

1. Please describe to me the typical Woreda health office officials/experts roles in your Woreda/community?
   a. How is Woreda health office officials occupied over the course of the day? Are there seasonal differences (campaign/political assignment) in how you use time?

The role of a HC head is coordinating the activities at the health center. Giving follow-up, fulfilling the necessary materials, doing a monthly evaluation, supporting those who scored a low performance and improving them. Moniroting, supporting and implementing the directions given from higher higher "governing" body. Checking what should be improved at the health center and outside the health center "health posts" and improving them.

2. As Woreda health office official, what kind of work do you do? Do these works different when facility status changes (Rural, urban and geographical locations)?
3. Do Woreda health office officials like you have restrictions/barriers on the accessibility? What restrictions/barriers? How do the restrictions/barriers influence the access of the PP and PA family planning services and supportive social networks?
   a. In your Woreda do the restriction/barriers change by social characteristic, such as (dis)ability, economic status, health status, educational level, religion, culture, geographical location, and marital status?

What can restrict is related with the attitude of the community. I don't think there will be a much challenge from the religious organizations. As I have to told you, the main restriction that needs a work is related with the attitude of the community. I don't see other restrictions.

4. Do you participate in any meetings, workshops, trainings, pregnant women conference, family, community, government, or other social networks organizations? Please tell me more.
   a. Please tell me how your participation in these organizations affect the PP and PA family planning services provision

As a health center head, I didn't participate on this area since I came to this position recently. However, I took the training when I was professional about community engagement on PP and PA family planning.

The training helped me to improve my understanding and give emphasis on this service. I took the training by Engeder heath at Wolaita Sodo. Besides, I didn't take other trainings on this topic.

5. In your Woreda do women assume leadership roles? What types of roles? Do these roles change by social characteristics, such as (dis)ability, economic status, health status, educational level, religion, culture, geographical location, and marital status?
   a. Please tell us about your own experience with assuming any leadership roles?
   b. Please tell me how your leadership roles affect the PP and PA family planning services provision

At our facility level, women leadership is encouraged. The HC head before me as a women, now the MCH focal is also a women. In our HC management 50% of them are women. from 9 members, 5 are women. Women assuming leadership roles can help them to understand about the problem associated with birth and using family planning since they experience on their own body. Men can't give birth.

6. In your Woreda/community who decides at what age people marry? What are the reasons for getting married at younger or older ages in this Woreda?
   a. Please tell me how their age at marriage affects their PP and PA family planning services use

Previously they marry around the age of 14 years. Now most of them marry after 20 years. The decision is made by them "couples" most of the time. The reason for early marriage is related with economy. When parents have low economic status and when they don't treat psychologically girls well, they get married.

I think the couples would be more advantageous if they can use PP and PA family planning. But, they have less awareness to use the service.

7. What are your suggestions to improve women's roles and participation to enhance the use of PP and PA family planning services?

There is a community perception that women are less competent to assume leadership and exercise it roles. This should be changed through education and creating awareness. At the facility and at the community levels, they should be motivated and encouraged to take responsibility. If we can motivate them, others "women" also see them and follow their footstep.

<u>Knowledge, Beliefs, and Perceptions</u>

Now we are going to discuss about the issues faced by women in accessing PP and PA family Planning services in your opinion.

1. In your Woreda what is appropriate behavior for a woman after delivery and abortion regarding the use of PP and PA family planning? How do they differ from adolescent girls and young women?
   a. How do these believe influence health behavior including the PP and PA family planning use?

What is common in our community is that after giving birth women will stay at home for a long time. Nowadays, the good things is most of them come to get vaccination. This is a good opportunity to provide FP. Previously, the use of vaccination and suing FP was low, but now they come for vaccination.

2. What are the social beliefs and perceptions that share women's expectations and aspiration for health care including PP and PA family planning?
3. In your opinion who should make decisions regarding use of PP and PA family planning services? What types of decisions do women make regarding use of healthcare including PP and PA family

planning services? What types of decisions regarding use of healthcare including PP and PA family planning services are made jointly?

Couples should make a joint decision after discussion regarding the use of family planning in other cases than PP and PA. But if they have a repetitive abortion or birth, it can be a threat to her health. In such cases, I believe she should be able to make decisions. In other cases, I think couples should make a joint decision.

Legal Rights and Status

Now we are going to discuss about the marriage related process prevalent in this community.

1. In your community are women who delivered recently legally allowed to receive PP and PA family planning service?
   - Is these same for boys and girls, people from different age groups, education background, ethnic groups, socio economic classes, excluded groups? How are they different? Why/ Why not?

It is legal for everybody to use family planning in our community.

2. How do recently delivered/aborted women access PP and PA family planning information and resource from the healthcare facilities?
   - Is these same for boys and girls, people from different age groups, education background, ethnic groups, socio economic classes, excluded groups? How are they different? Why/ Why not?
   - Do the people in your community support recently delivered/aborted women to access PP and PA family planning information and resources? How do they provide support? Who are these people? Why do they provide support?
   - Are there certain groups in your community who do not support this? Who are these people? Why don't they support?

They get the information and resources from the health care providers. Nowadays there is an integrated services for youths. Youth friendly service is comfortable for that. So, there is access for youth as well.

There could be some people in the community who have poor awareness and who do not support PP family planning. But I don't know them. They can be there.

There are also others who used it "PP/PA" and benefited from it who support others. "still the informant couldn't mention some"

3. Please tell me women's experience with accessing healthcare information and resources including PP family planning.
   - Do you feel they have a right to get healthcare information and resources including PP family planning services? Why/why not?
   - Do you think women's experience is different from other people in your community? How? Please tell me more
   - Is there legal mechanism you can follow to ensure they get quality healthcare information and resources they need? Please tell me more

We can't understand the level of acceptance from the community level. But we use through the performance report. By checking from the report we can understand the level of utilization in the

community. We don’t have mechanism to ensure quality information level in the community. We use monthly report of utilization.

With this we have come to an end of the discussion. Do you want to add anything before we end the discussion or if you have any questions you want to ask?

Thank you for your time and in case you want, you can get in touch with us through the details mentioned in the consent form.

****

Summary:

The interview was conducted at the office of the health center’s head. The room was temporarily closed to facilitate a smooth interview. He is new and looks cautions in answer the questions. However, I believe he tries to acknowledge the existing reality and looks honest in his replies. The entire interview session was good.

Interview details

Interviewee code	KII_BA_WoEx_270722
Sex	Male
Age	38
Job title	PHO
Name of interviewer	Beimnet Desalegn
Date	27/07/22
Time started	4:46 lt
Time finished	5:50 lt
Venue	Bedesa Health office

Assets and Resources

Discussion

Now we are going to discuss about the Healthcare resources and assets available for provision of PP and PA family planning services in your area.

I: - Health services (PP and PA family planning services) availability: Can you tell me the types of healthcare facilities that provide PP and PA family planning services in your area?

a. What types of FP services are available at the healthcare facilities?
b. Are these services equally available to boys, girls, young, old, married, unmarried, people from different geographic locations, religious or cultural background, language etc.? Please tell me more

R: - postpartum and post-abortion family planning services were provided in our woreda before … but now recently it was gaining high attention and we are implementing it as a program in collaboration with other concerning bodies (engender health).

We have four health centers and of which three health centers were selected as implementation areas and provide post-partum and post-abortion family planning. We have trained health workers in all health centers. … we were providing family planning in the post-partum period but what is new is we now it gains special impasse and attention as a tool to decrease unmet needs. Now delivery service was only provided at the health center level so we trained

community health extension workers to refer pregnant mothers to the health center. So, the delivery mother gets immediate post-partum family planning at the health center.

Badesa health center, Dimtu health center, Seka health center, and Koyo health center were actively engaged in this post-partum and post-abortion family planning service provision. In Badesa health center all midwives have got training …

Most of the time for immediate post-partum and post-abortion family planning long-acting contraceptive is preferable … but short-acting contraceptive is more preferred by users and we face a shortage of them frequently. … I think depo is out of the market now so we have a shortage of it too. We have enough stock of long-acting family planning … we have Implanon, IUCD… but we have no users as we expected to. Shortage of depo is related with … its user is high when compared with another contraceptive method… many women shifting to depo after using some other method. The supply of depo is decreasing from time to time…

Eee… the provision of family planning service … there is a system in which we serve for youth with special care… in some of our health centers. In Badesa health center we have a youth-friendly care service center where we serve youth. And we are serving all ages and women equally without looking at their background. We serve any time when a woman wants the service in a health center or health post. We only consider women's needs.

I: - Accessibility of the PP and PA family planning services

c. Can you tell me how far the PP and PA family planning services providing facilities are located? How long does it take for women to access the facilities?
d. What type of transportation means do they use to reach the facilities? How easy or difficult is it to use the transportation services?
e. Is there any cost associated with accessing the PP and PA family planning services? Please tell me more about this
f. Is the PP and PA family planning service is accessible at all time at the healthcare facilities? Are there types of FP methods of users' choice in the healthcare facilities?

g. Are these services equally accessible to boys, girls, young, old, married, unmarried, people from different geographic locations, religious or cultural background, language etc.? Please tell me more

h. How easy could they access the PP and PA family planning services? Do you think that their access to the PP and PA family planning services are affected because of their background? How?

R: - eee … we have kebeles which are far from the health center and women get immediate post-partum family planning service when they deliver at the health center. Eee... for pregnant mothers, we give ambulance services to come to the health center with an ambulance. other women who need family planning services use the contraceptive method from the health post. But there are kebeles which are far for health post … Anka kebele and Tora Sedebo kebele are far for health post. We have backup service at both kebele due to these two kebeles are far from the health facility. In our back, up service, we provide depo and short-acting contraceptive methods. We can't provide long-acting family planning because we did not have a facility there and we did not bring equipment there. Eee Dagaga Lenda kebele is also far from sake health center… so arranging backup service health extension workers serve the community at the kebele level.

Eee… to get a family planning service woman do not pay for it… it is free of charge. In addition to this eee… at the health center, there is 24-hour service and at the health post, health extension workers serve during day time only (government working time). There might be a gap due to government meetings and campaigns. Regarding drug availability, since it is a programmed drug they (health center) get it from the regional health office and donner directly. Health post refers to women who need to use IUCD … another contraceptive method is given by health extension workers at the health post.

I: - Means to access the PP and PA family planning services

i. Please tell me about your facilities' resources and assets. Please tell me the resources and assets belong to these health facilities to provide PP and PA FP service.

j. How is the use of the facility/Woreda health office resources and assets decided? Who makes the decision? Do you involve in the decision making? How do you think that affects access to the healthcare services including PP and PA family planning by women?
k. Please tell me about income of in this Woreda. Please tell me about your woredas income.
l. How is the use of the woreda's income decided? Who makes the decision? Do you involve in the decision making? How do you think that affects their access to the healthcare services including PP and PA family planning?
m. In your woreda how is use of PP and PA family planning seen? Is it acceptable for women to use PP and PA family planning? Why/why not? Please tell me more (what about boys and girls)

R: - eee… in some of the health centers in our woreda family planning service and ANC service are provided in the same room. We have a shortage of infrastructure or room … in Bedesa health center we are trying to solve this problem after the hospital in Badesa is start functioning. Regarding equipment, we have enough supply from donors so we are using it and also have enough supply of contraceptive methods. But we have fear for the future because many donner and supplier were decreasing their supply from time to time.

Regarding woreda budget allocation … generally, 15 percent of the budget is directly allocated/allowed for the health sector. But it is not clear …

Most of the time health sector is supported by different NGOs … as you know it is so difficult to execute health activity by the government budget alone. If we did not get support from stockholders we can't serve the community by government budget alone. … I think the government budget could not cover fuel expenditure for ambulances.

Regarding our office participation, as a woreda carbine, the member health office head participates in budget allocation. … all woreda office heads are members of the executive body … they are about 20 in number.

I think the government has no budget allocation problem … but we have problems of getting money in cash … so as woreda we use money collected from tax … farmers' land and some from the mining industry as woreda this is finance source but it is not enough.

After the budget is allocated by woreda our health office management reallocates for health centers… management decided on budget breakdown and other money we got from donors.

Yeah, post-partum and post-abortion family planning still now needs more attention … since it is given to mothers immediately, it is seen by women as a new idea … so we have to work on awareness creation more… makes me harry they (recently delivered mother) said. Many women perceive that it has side effects if they use it immediately after delivery … also family push might be one factor, the other thing is lack of awareness. The other issue is maybe we don't have many users to show community as a model…

I: - In your opinion what should be done to improve access to the PP and PA family planning services for all women in your community?

R: - the main thing is awareness creation for women and other community members. The other thing is health workers' commitment and our support in sensitizing the community and other supporting groups in the community and creating awareness among them to influence women. Again, working with elders and religious leaders… are very important.

Practices, Roles, and Participation

Now we are going to discuss about your role and responsibilities.

I: - Please describe to me the typical Woreda health office officials/experts roles in your Woreda/community?

a. How is Woreda health office officials occupied over the course of the day? Are there seasonal differences (campaign/political assignment) in how you use time?

R: - health office head has the responsibility of supporting health centers and health posts to do their activity and evaluating their performance. The Department of mother and child health in the health office work with the health center closely we also support them. We have Weekly supportive supervision at the health center with the health center head… so we evaluate their

performance. We have also monthly supportive supervision at the health center and health post so as office head I involve in this activity.

The effect of meetings/campaigns in our activity is not such big because service-providing health workers did not regularly take part in meetings or campaigns directly but the health center head and different department focal participate in campaign/meetings as representatives. Maybe in case of campaign/meeting at kebele level, there is some effect in their (health workers) activity.

I: - As Woreda health office official, what kind of work do you do? Do these works different when facility status changes (Rural, urban and geographical locations)?

R: - as health office head my responsibility is over watching all the activity under the woreda health facility. Yea we have rural and urban kebeles and the burden of activity differed from place to place.

I: - Do Woreda health office officials like you have restrictions/barriers on the accessibility? What restrictions/barriers? How do the restrictions/barriers influence the access of the PP and PA family planning services and supportive social networks?

b. In your Woreda do the restriction/barriers change by social characteristic, such as (dis)ability, economic status, health status, educational level, religion, culture, geographical location, and marital status?

R: - Big challenge is health workers' motivation decrease … may be budget restraint to review performance and evaluate the activity at health post or center. Also, backup services in remote areas have also budget restrictions. On the other hand, we have also gaps in the health office in regular follow-up at the health center and health post.

I: - Do you participate in any meetings, workshops, trainings, pregnant women conference, family, community, government, or other social networks organizations? Please tell me more.

c. Please tell me how your participation in these organizations affect the PP and PA family planning services provision

R: - Regarding PP and PA family planning I participated in engender health sensitization program … at that time health workers received detailed training on PP and PA family planning. we expect that if we did with all our effort on PP and PA family planning we can achieve the unmet need for contraceptives very well. If this program is implemented very well it will a good improvement in maternal health.

I: - In your Woreda do women assume leadership roles? What types of roles? Do these roles change by social characteristics, such as (dis)ability, economic status, health status, educational level, religion, culture, geographical location, and marital status?

d. Please tell us about your own experience with assuming any leadership roles?
e. Please tell me how your leadership roles affect the PP and PA family planning services provision

R: - at Badesa health center head was female, even though she was now not in position. In Koyo health center also, women lead the health center for a long time. A long time ago deputy head of the health office was a woman …

I have no long-time experience in leadership… but in this one year I was assigned for this position… when we evaluate the performance we have a focus area… for instance when we evaluate PP or PA family planning we take into consideration what we got in training. During our review meeting, we share experiences at the health center.

I: - In your Woreda/community who decides at what age people marry? What are the reasons for getting married at younger or older ages in this Woreda?

f. Please tell me how their age at marriage affects their PP and PA family planning services use

R: - it is different from place to place even within the same woreda … at this time most of the women marry an early age and they delay coming and use contraceptives… they didn't participate in community activity … because of this they have fear of using a contraceptive method. What is good now is women can get contraceptives near health posts or health centers. Yeah, when we look at our report we have contraceptive users starting from 15 and above… sometime we observe privet clinics and most of the youth use contraceptives there.

I: - What are your suggestions to improve women's roles and participation to enhance the use of PP and PA family planning services?

R: - to improve women's participation and contribution to enhancing PA or PP family planning use we are working with schools … that are a potential area to get young women. We have to capacitate them then they go and help their family. So, working with the school is very important to improve this service efficiency. Also, we have to work to incorporate the privet clinic service report in the woreda report because to improve service there we have to track their activity.

Knowledge, Beliefs, and Perceptions

Now we are going to discuss about the issues faced by women in accessing PP and PA family Planning services in your opinion.

I: - In your Woreda what is appropriate behavior for a woman after delivery and abortion regarding the use of PP and PA family planning? How do they differ from adolescent girls and young women?

a. How do these believe influence health behavior including the PP and PA family planning use?

R: - I cannot confidently say the community sees this (PP and PA family planning) as appropriate behavior. If they take it as appropriate the performance may be improved. It (PP and PA family planning users) does not exceed 10 percent in our woreda which is a very small number when we compare with the total delivering mothers at the health center. So, more work is expected from religious leaders and elders …

I: - What are the social beliefs and perceptions that share women's expectations and aspiration for health care including PP and PA family planning?

R: - yeah there are rumors… among the community about some form of contraceptives. For instance, many women think that using IUSD decrease sexual desire. And also complain about discomfort during sex.

I: - In your opinion who should make decisions regarding use of PP and PA family planning services? What types of decisions do women make regarding use of healthcare including PP and PA family planning services? What types of decisions regarding use of healthcare including PP and PA family planning services are made jointly?

R: - it is advisable if they decided together. Because some contraceptive has a side effect that stays for some time so if it was decided together there will be no blaming each other. Other ways male partners may not support a woman. The mutual decision is good for both our family as a whole.

Legal Rights and Status

Now we are going to discuss about the marriage related process prevalent in this community.

I: - In your community are women who delivered recently legally allowed to receive PP and PA family planning service?

- Is these same for boys and girls, people from different age groups, education background, ethnic groups, socio economic classes, excluded groups? How are they different? Why/ Why not?

R: - yeah everybody has the right to use their choice… at all ages, sex, background… other ways I don't know any rule which prevents family planning use.

I: - How do recently delivered/aborted women access PP and PA family planning information and resource from the healthcare facilities?

- Is these same for boys and girls, people from different age groups, education background, ethnic groups, socio economic classes, excluded groups? How are they different? Why/ Why not?
- Do the people in your community support recently delivered/aborted women to access PP and PA family planning information and resources? How do they provide support? Who are these people? Why do they provide support?
- Are there certain groups in your community who do not support this? Who are these people? Why don't they support?

R: - first information flow starts at a higher level at the bureau and health office ... there are stakeholders from the regional bureau to the lower health post level. So, we cascade information to health workers then health extension for 1 to 5 team leaders and health development army leaders then they disseminate information to the community. Most of the time women got information from community health workers.

Eee ... yeah those who support women to use family planning methods are those I mentioned above health development army leaders, 1 to 5 teams, and health extension workers who support women in the community. Again, there are informally not supporting people in the community I can't mention specifically... for instance, one day in a funeral ceremony one preacher teach people not to use contraceptive time this as things happen often. At that time, we trayed to correct him. Yeah, most of the time it is at the individual level not ...

I: - Please tell me women's experience with accessing healthcare information and resources including PP family planning.

- Do you feel they have a right to get healthcare information and resources including PP family planning services? Why/why not?
- Do you think women's experience is different from other people in your community? How? Please tell me more
- Is there legal mechanism you can follow to ensure they get quality healthcare information and resources they need? Please tell me more

R: - coming to the health center and getting any information including service … all clients have full right. Also, women understand that is their right to get full information about services and drugs.

On the health worker side, they respect and support their client respecting their(clients) right. All health workers understand their rights… the only thing is commitment difference by health workers.

To ensure information quality requires research study… again the information was cascaded by health professionals so we believe that it is reliable.

With this we have come to an end of the discussion. Do you want to add anything before we end the discussion or if you have any questions you want to ask?

Thank you for your time and in case you want, you can get in touch with us through the details mentioned in the consent form.

****

Summary

This interview was conducted in Badesa town, on 27/07/22. the informal was MCH department coordinator and he was delegated by the head of health office, the interview was conducted at the office and the place was very quiet. To reduce the flow of staff members or other customers we closed the door until the end of the interview. The informant was confident and well aware of the PP/PA. however, sometimes he explained things from a political point of view, and we tried our best to ket him in our issue of interest by explaining our aim again and again. Generally, I believe it is a detailed and informative session.

Interview details

**Interviewee code**	KII_DI_WHChead_181114
**Sex**	Male
**Age**	29
**Job title**	PHO
**Name of interviewer**	Beimnet Desalegn
**Date**	18/11/2014
**Time started**	4:15 lt
**Time finished**	5:06 lt
**Venue**	Dimtu Health center

Assets and Resources

Discussion

Now we are going to discuss about the Healthcare resources and assets available for provision of PP and PA family planning services in your area.

1. Health services (PP and PA family planning services) availability: Can you tell me the types of healthcare facilities that provide PP and PA family planning services in your area?
   a. What types of FP services are available at the healthcare facilities?
   b. Are these services equally available to boys, girls, young, old, married, unmarried, people from different geographic locations, religious or cultural background, language etc.? Please tell me more

R: - yeah, we are providing immediate PP and PA family planning in our health center. We have trained health worker on this specific service … all MCH team has got training on PP and PA family planning here in Dimitu health center. Regarding contraception method we have all option of contraceptive in our hand. We have IUCD, Implanone, pills eee depo. We got directly from EPSA based on our RRF report. We don't have supply shortage.

We provide service fairly to our community… all health workers including community health extension worker have got training on how to prepare pregnant mothers for postpartum family planning so they have good skill on how to treat mothers … so they serve all equally. Community health extension workers have regular monthly meeting with pregnant mother and during their meeting they discuss about family planning method and their next plan if they agree they also

prefer their contraceptive choice to use after having birth. Again, here in health center health worker counsel and make them use immediate PP contraceptive method.

2. Accessibility of the PP and PA family planning services

a. Can you tell me how far the PP and PA family planning services providing facilities are located? How long does it take for women to access the facilities?
b. What type of transportation means do they use to reach the facilities? How easy or difficult is it to use the transportation services?
c. Is there any cost associated with accessing the PP and PA family planning services? Please tell me more about this
d. Is the PP and PA family planning service is accessible at all time at the healthcare facilities? Are there types of FP methods of users' choice in the healthcare facilities?
e. Are these services equally accessible to boys, girls, young, old, married, unmarried, people from different geographic locations, religious or cultural background, language etc.? Please tell me more
f. How easy could they access the PP and PA family planning services? Do you think that their access to the PP and PA family planning services are affected because of their background? How?

R: - yeah, we have clients who are come here from far area … for instance Blate Charicho is 30 KM far from health center so a client coming from there they suffer to get transportation… they may use motor cycle and they pay 120 birrs for single trip. They do not have health center there so they come here for delivery service. Sometime, marketing day they have transportation access other ways, they use motor cycle. Here in the Dimitu town has no problem they can walk to come here. But those who come from far kebele may expend 250 birr or above for a single visit to health center.

Regarding family planning service … we provide free service. All option of family planning was free of charge.

We get contraceptive method from regional health bureau based on our need … we got it free of charge.

Sometimes clients may not get a method they chose to use due to different reason… but as much as possible we serve 24 hours. Sometime there might be delay in supply and because of this there might be shortage of some type of contraceptive method. Its not only for contraceptive method … we also face shortage of medication for other medical conditions due to inflation or financial problem. We have monthly reporting system by RRF and we are reporting without any delay but sometimes there is short of some drug at regional level and at that time we do not do anything… just we wait for there supply. Sometime to mange it we try to exchange or shift contraceptive from one health center to other … or share among the health center.

In our health center we treat all clients equally… as a health profession. all the client applies for card in their arrival and get the service they want based on their order of arrival. Each client has face to face contact with health workers and have discussion with them …

3. Means to access the PP and PA family planning services

a. Please tell me about your facilities' resources and assets. Please tell me the resources and assets belong to these health facilities to provide PP and PA FP service.
b. How is the use of the facility/Woreda health office resources and assets decided? Who makes the decision? Do you involve in the decision making? How do you think that affects access to the healthcare services including PP and PA family planning by women?
c. Please tell me about income of in this Woreda. Please tell me about your woredas income.
d. How is the use of the woreda's income decided? Who makes the decision? Do you involve in the decision making? How do you think that affects their access to the healthcare services including PP and PA family planning?
e. In your woreda how is use of PP and PA family planning seen? Is it acceptable for women to use PP and PA family planning? Why/why not? Please tell me more (what about boys and girls)

R: - As a health center, we have different problems … this health center is located near to border with two regions … we have 8 kebele to serve as under Wolaita zone health center but we have also clients from nearby kebeles from Oromia and Sidama border kebeles and other wolaita zone woredas like Humbo woreda, and loka Abala we have clients coming there so the resource we

have and people we are serving is incomparable. Our budget is only settled for eight kebele people but we are serving additional 12 kebeles forms different regions. We are totally serving 20 kebeles population. Because of this we have a shortage of drugs and crowding in the facility on marketing day. We have to provide service in separate rooms… we have to have separate delivery, ANC, and family planning room… but now we are merging the family planning room and ANC room due to a shortage of room.

We board … during budget allocation this board decides on the budget. As board members, we participate in meetings. We rise our concern to be considered during budget allocation by our representative, the head of the health center.

Our participation helped us in any way... for instance, it helped us to get money for buying required materials …

Many people living here in Dimtu are farmers… some people living around the town area are engaged in trading activities.

I have seen some changes in PP and PA service use since engender health started the implementation of immediate PP and PA family planning. Before pregnant mothers were not counseled during their ANC follow-up about the contraceptive methods but now it is considered as one dimension of counseling during ANC follow-up. Even after delivery service health workers counsel mothers, this is one change for me in this activity. During the pregnant mother forum also, it is considered as one agenda for dissuasion. It also helped us to improve our performance in unmet need for contraception.

4. In your opinion what should be done to improve access to the PP and PA family planning services for all women in your community?
5.

R: - Yeah, to improve service utilization we have to conduct regular pregnant mother’s forum and discusses about PP/PA family planning with pregnant mothers. We have to serve peoples living in far kebele (Belate Charicho kebele), we have to create means to address those people. Also, all health extension workers and other health professional in addition to midwife have to get training. And all trained health worker has to serve community at kebele level going into the

community. Also elders, religious leaders and popular individuals should have to participate or create the system to involve them. Eee we have to involve husband in this system and if all the people have awareness … we can improve the service access by mother.

We have waiting room here for pregnant mother … those mothers far from health center come and stay here at a health center for one or two weeks …

Practices, Roles, and Participation

Now we are going to discuss about your role and responsibilities.

1. Please describe to me the typical Woreda health office officials/experts roles in your Woreda/community?
   a. How is Woreda health office officials occupied over the course of the day? Are there seasonal differences (campaign/political assignment) in how you use time?

R: - my responsibility is… to follow health center activity. For instance, we have trained health workers in PP/PA family planning … so I observe their activity based on their activity plan. I also observe the registration books and other recording materials … generally I follow for service quality. I also evaluate their performance and put the direction on as required. In addition to health center activity, I also follow activities at the health posts and among the community by community health workers. We have a general meeting monthly and also on Friday each case team (OPD, Laboratory…) has a discussion… then as health center head finally I have a meeting with the case team coordinator on issues raised by workers together.

Yeah, there is a difference in the role or workload in some circumstances, if I have a meeting or campaign I delegate someone who can act and behave for me till my return.

2. As Woreda health office official, what kind of work do you do? Do these works different when facility status changes (Rural, urban and geographical locations)?

R: -

3. Do Woreda health office officials like you have restrictions/barriers on the accessibility? What restrictions/barriers? How do the restrictions/barriers influence the access of the PP and PA family planning services and supportive social networks?
   a. In your Woreda do the restriction/barriers change by social characteristic, such as (dis)ability, economic status, health status, educational level, religion, culture, geographical location, and marital status?

R: - the big challenge here is training related… in MCH-related training the trainee body trained only midwife professionals… but in our health center not only do midwives serve in the MCH department there are other professionals (nurse and Public health officers) but training organizing body did not allow for them to attend the training. This made things difficult for us to manage staff… if we share burden we have share benefit too. We do not have sufficient midwives… we cannot cover the MCH burden only by midwifery so we use other nurses and public health professionals. To gain a better outcome in this program MCH related training organizers should have to consider this issue. We have only two midwives … the standard is six and two midwives in one shift. So, we use nurses and public health professionals instead of midwives. This is always raised by staff members. At the kebele level, health extension workers should also have to train. We have a transportation problem to go and support for far kebele (Bilate charicho) which is 30 Km away from Dimitu health center.

4. Do you participate in any meetings, workshops, trainings, pregnant women conference, family, community, government, or other social networks organizations? Please tell me more.
   a. Please tell me how your participation in these organizations affect the PP and PA family planning services provision

R: - Yeah, I have attended training or meeting at different times, and I learned lessons… it helps me to understand the community and their need, and also to develop my professional skill and leadership skill. I also participated in the community pregnant mother forum at different times. During the community forums, we do awareness creation and it helped us in PP/PA family planning acceptance.

5. In your Woreda do women assume leadership roles? What types of roles? Do these roles change by social characteristics, such as (dis)ability, economic status, health status, educational level, religion, culture, geographical location, and marital status?
    a. Please tell us about your own experience with assuming any leadership roles?
    b. Please tell me how your leadership roles affect the PP and PA family planning services provision

R: - at kebele level… kebele women affaire is a woman and she have high acceptance and influence among the community… they are heard by women and help us in our work while we do on women. They are also involved in awareness creation and education activities with us… we education given by women most of the time mothers are very happy. Again, here at the health center MCH coordinator is a woman.

6. In your Woreda/community who decides at what age people marry? What are the reasons for getting married at younger or older ages in this Woreda?
    a. Please tell me how their age at marriage affects their PP and PA family planning services use

R: - many women marry at an early age in 15 or above… the main reason to marry at an early age is lack of awareness, low family economic status … there also be peer pressure, also environmental effects since the area is hot …

most of the time youths were not using contraceptives… but now recently it was changing since some awareness creation training was given to young married women by NGOs.

7. What are your suggestions to improve women's roles and participation to enhance the use of PP and PA family planning services?

R: - counseling program should be strengthened… the main focus to improve PP/PA acceptance should be working with pregnant mothers in the community and strengthening the pregnant

mother's forums. Health workers here at the health centers should be monitored and evaluated regularly, and have to work with the community.

Knowledge, Beliefs, and Perceptions

Now we are going to discuss about the issues faced by women in accessing PP and PA family Planning services in your opinion.

1. In your Woreda what is appropriate behavior for a woman after delivery and abortion regarding the use of PP and PA family planning? How do they differ from adolescent girls and young women?
   a. How do these believe influence health behavior including the PP and PA family planning use?

R: - it should be decided by users… if it is taken by pushing by health workers they(users) may blame health workers for any consequence including the side effect of a method. So, health workers should have to give full information regarding contraceptive options then the decision should be left to the user. Husband participation in decision-making is also mandatory.

2. What are the social beliefs and perceptions that share women's expectations and aspiration for health care including PP and PA family planning?

R: -

3. In your opinion who should make decisions regarding use of PP and PA family planning services? What types of decisions do women make regarding use of healthcare including PP and PA family planning services? What types of decisions regarding use of healthcare including PP and PA family planning services are made jointly?

R: - it should be decided by users… if it is taken by pushing by health workers they(users) may blame health workers for any consequence including the side effect of a method. So, health

workers should have to give full information regarding contraceptive options then the decision should be left to the user. Husband participation in decision-making is also mandatory.

Legal Rights and Status

Now we are going to discuss about the marriage related process prevalent in this community.

1. In your community are women who delivered recently legally allowed to receive PP and PA family planning service?
    - Is these same for boys and girls, people from different age groups, education background, ethnic groups, socio economic classes, excluded groups? How are they different? Why/ Why not?

R: - yeah, it is legal. Because It is important for community or women and child health in decreasing unmet needs and using delivery time as an opportunity to get mother for discussion and counseling.

2. How do recently delivered/aborted women access PP and PA family planning information and resource from the healthcare facilities?
    - Is these same for boys and girls, people from different age groups, education background, ethnic groups, socio economic classes, excluded groups? How are they different? Why/ Why not?
    - Do the people in your community support recently delivered/aborted women to access PP and PA family planning information and resources? How do they provide support? Who are these people? Why do they provide support?
    - Are there certain groups in your community who do not support this? Who are these people? Why don't they support?

R: - community gets information about PP/PA from community health workers at the kebele or in their village. Again, at the health center after delivery or during ANC follow-up at health post or health center…

There is a health development army 1 to 5 team and health cabin in the community these teams support women to get or use service in the health facility. There is no one against this service.

3. Please tell me women's experience with accessing healthcare information and resources including PP family planning.
   - Do you feel they have a right to get healthcare information and resources including PP family planning services? Why/why not?
   - Do you think women's experience is different from other people in your community? How? Please tell me more
   - Is there legal mechanism you can follow to ensure they get quality healthcare information and resources they need? Please tell me more

R: - there are clients' rights and responsibilities again there is right and responsibility to health workers so we have displayed them on a billboard in front of the health center get so our client can get information from there or also we teach them about their right in another different way. we check for good relations between the client and service provider… we are working for client satisfaction… we have mechanisms of following their satisfaction. We also get feedback from our clients by deferent means. There are questioners and the client answer then we calculate their satisfaction. And take measures accordingly.

With this we have come to an end of the discussion. Do you want to add anything before we end the discussion or if you have any questions you want to ask?

Thank you for your time and in case you want, you can get in touch with us through the details mentioned in the consent form.

****

Summary

This interview was conducted in Dimtu health center with the head of the health center. The interview was conducted at his office and the place was quiet. To reduce the flow of staff members or other customers we closed the door until the end of the interview. The informant

was confident and well aware of the program. In general, the discussion was detailed and informative.

Discussion

Rapport Building

Thank you for proving consent to participate in the discussion. There are no right or wrong answers, please share your frank opinion, as it will help us in understanding the situation better.

Enquire about her recent delivery experience. An example is given below:

Please tell us about family planning service in your woreda. Where did women get family planning services? Who provided the services? How far is the health facility from women's home (in different directions? How do women travel to the health facility?

Assets and Resources

Discussion

Now we are going to discuss about the Healthcare resources and assets available for provision of PP and PA family planning services in your area.

1. Health services (PP and PA family planning services) availability: Can you tell me the types of healthcare facilities that provide PP and PA family planning services in your area?
   a. What types of FP services are available at the healthcare facilities?
   b. Are these services equally available to boys, girls, young, old, married, unmarried, people from different geographic locations, religious or cultural background, language etc.? Please tell me more

There are five functional health centers and one hospital that provide this service. Engender health has selected one health center, Dimtu health center as implementation site. But the service is available in all facilities. After the intervention there is some improvement in Dimtu health center. Previously there is some misconception regarding family planning that it is IUCD only that can be given immediately after PP. But now there is a better understanding that any method can be given within 48 hours after the PP. The methods available in those facilities include IUCD, implanon, Jaddle, depo and other short acting FP. The service is available for everyone who wants to use. For example in Dimtu there is YFS that is friendly for adolescents. For those who decided to use permanent method, the service is also offered. The service is given in collaboration with the hospital. There are already trained IESO who can provide the service. YFS also got attention and the service is also available for youths. Recently every youth below the age of 24 will be referred to youth clinic and every one attends that clinic. So, everyone will be sent to youth clinic if below 24 years of age.

2. Accessibility of the PP and PA family planning services
   a. Can you tell me how far the PP and PA family planning services providing facilities are located? How long does it take for women to access the facilities?
   b. What type of transportation means do they use to reach the facilities? How easy or difficult is it to use the transportation services?
   c. Is there any cost associated with accessing the PP and PA family planning services? Please tell me more about this
   d. Is the PP and PA family planning service is accessible at all time at the healthcare facilities? Are there types of FP methods of users' choice in the healthcare facilities?
   e. Are these services equally accessible to boys, girls, young, old, married, unmarried, people from different geographic locations, religious or cultural background, language etc.? Please tell me more

f. How easy could they access the PP and PA family planning services? Do you think that their access to the PP and PA family planning services are affected because of their background? How?

Before only health centers provide YFS. Now health extension workers also got training and referral the cases towards health center. Indeed the service is available at the health post. For example they insert implanon and other methods like depo and pills. Those who trained remove implanon as well and those who are not refer to higher facilities.

The health facilities are accessible in every areas because we have already five health centers and the sixth one is underconstruction. Since the topography is difficult in this woreda, thy build more health centers than the population number. so, the health facilities are available nearby. They use motorcycle if far and if near they simply walk. Most of the women use the service when they come to the market on thruday around Dimtu. The transportation is accessible.

There is no cost for FP. AMREF brings the necessary supplies and EPSA also provides the service. To prevent artificial shortage we shift materials from one area to another. The is no cost related with it. The service is available 24 hours since there is three shifts who work 8 hours each. The service is accessible to all whether married or not, young or old, boys or girls. Regarding the religious conditions, there was a discussion about mainstreaming and we identified some barriers. People from Hiwot Qal church said we shouldn't use FP. However people from other churches such as Kale-heywet and Orthodox church challenged them saying we were not told to invade the world and we were also told to live a balanced life. The religious people challenged each other and we managed it and clearing misconceptions regarding FP.

There are still some challenges regarding PP family planning. On other times people willingly use the method they choice. But for the case of PP there are some resistances. During ANC visits when we advise them about PP family planning, they are not happy to listen to that. When we tell them about FP immediately about FP, they are not happy and it becomes strange to them. So, it is not easy to provide PP family planning. Now we are trying to address this issue is pregnant mothers conference through teaching.

3. Means to access the PP and PA family planning services

a. Please tell me about your facilities' resources and assets. Please tell me the resources and assets belong to these health facilities to provide PP and PA FP service.
b. How is the use of the facility/Woreda health office resources and assets decided? Who makes the decision? Do you involve in the decision making? How do you think that affects access to the healthcare services including PP and PA family planning by women?
c. Please tell me about income of in this Woreda. Please tell me about your woredas income.
d. How is the use of the woreda's income decided? Who makes the decision? Do you involve in the decision making? How do you think that affects their access to the healthcare services including PP and PA family planning?
e. In your woreda how is use of PP and PA family planning seen? Is it acceptable for women to use PP and PA family planning? Why/why not? Please tell me more (what about boys and girls)

Resources start from human resource. In the project they selected Dimtu "HC"and providing the training for most of the staffs. They provided the training for those who also work at the OPD. In other facilities there those who trained have already left or trained some three/four years ago. Regarding trained staff, the trained ones are in Dimtu only. There are some midwifery in the hospital "Bitana Hospital" who took the training. There is shortage of staffs trained regarding immediate PP family planning. The attention given in other than Dimtu area is less. They need training.

Regarding logistics, there is no shortage of resources. We have resources in th health posts as well. Last time we identified areas where there is excess implanon and shared to other facilities who need them. We are preventing artificial shortage of resources. So, there is no shortage of resources.

There is no problem related with the facility and equipment in this “Dimtu” hospital. Other health facilities have nonfunctional equipment. When the gov’t builds a health center, they provide everything necessary for that including screens. But there is mishandling of equipment that exposes to be not well functional. Rooms are also available and there could be some gaps regarding on how to use the available rooms. There are also screens.

The woreda logistic is a management member here they gather information from every facilities brings for decisions. EPSA delivers every two months the necessary materials for FP. Our logistic brings from Hawassa and we facilitate the transportation for that. Last time when we faced shortage of “contraceptives” we discussed with EPSA and they brought in discussion with AMREF health.

The income condition of the woreda is difficult nowadays. Even the gov’t worker is getting half/quarter of a salary. There is shortage of rain in the woreda and now we have a lot of people who need immediate food supply. By the way the income and food condition is related with the use of family planning. Women were happy to use it when they have something to eat and have milk available for them to drink. There is household food shortage. As woreda this woreda and humbo is one of the hotspot where there is frequent food insecurity. This fact is known by the regional and federal gov’ts. The woreda health head always participates in the decision making and part of the management of member.

PP and PA family planning were previously not part of our main agenda. Now it is becoming part of the pregnant women monthly conferece. In Dimtu area there are women who are participating every month. However, they thing something strange and not quite interest as I have told you previously. There are some women who became pregnant when they are back after postpartum. They say it is not necessary since their body is not yet well matured after birth. They have a view that it is not necessary after PP. There are some health extension workers who have misconceptions about it.

4. In your opinion what should be done to improve access to the PP and PA family planning services for all women in your community?

First, we have to work at lower level until people change their mind as they use other FP methods. “They don’t see PP and PA as other methods of family planning” . The intervention “by Engender health” included only Dimtu health center where 4 out of 9 kebeles were included. We can scale-up on the remaining five kebeles. All health extention workers need to take a training. Health extentios work is becoming week from time to time. all women development army leaders should also be trained. They also lead other 1 to 5 leader s and other 30 women. I believe the work in not done at the grass root level. Another one is training the husbands. There is influence from men and they should be well aware of “PP” family planning. To facilitate this there is a need for some budget support. Previously there were NGO’s like INTRA-HEALTH and IHI who trained some men and trained them about FP. They gave some money to motivate men and after they left the program also ended. So, it has to be budget supported or should be enforced like other activities. We have to use different techniques to put it at grass–root level.

Practices, Roles, and Participation

Now we are going to discuss about your role and responsibilities.

1. Please describe to me the typical Woreda health office officials/experts roles in your Woreda/community?

a. How is Woreda health office officials occupied over the course of the day? Are there seasonal differences (campaign/political assignment) in how you use time?

Our main roles participating in Woreda base plan and DHIS. I have checked whether PP family planning is part of the plan. We plan also other health related indicators. First we plan and follow the implementation and follow-up. We follow who do what and what are the main gaps. We then support those who need supervision. We check the reports months and make a review meeting. Then we give the feedbacks based on the findings. When there is campaigns, there is a big difference "in service provison." For example, this year is full of campaigns. It affects our main activities. There were covid campaigns, measles campaign, and polio campaigns that affected our routine activities. People forget even the routine work during campaigns.

2. As Woreda health office official, what kind of work do you do? Do these works different when facility status changes (Rural, urban and geographical locations)?
3. Do Woreda health office officials like you have restrictions/barriers on the accessibility? What restrictions/barriers? How do the restrictions/barriers influence the access of the PP and PA family planning services and supportive social networks?
   a. In your Woreda do the restriction/barriers change by social characteristic, such as (dis)ability, economic status, health status, educational level, religion, culture, geographical location, and marital status?

The main barrier I would say is shortage of the trained staff. Most of the staffs in the lower levels are not trained and this can be a barrier. So, I think staffs at lower levels need to be trained. Scaling up should be done because I believe the works around Dimtu HC are not being done in other areas.

Besides there are no barriers from "social, religious, geography…" As I have mentioned the previous example about some religious sects, there is no extreme resistance from different groups. There could be a challenge related with the economic condition. There is shortage of food and people think about hunger, not about FP. They even believe FP would worsen their life condition on top of hunger. In addition as I have mentioned shortage of trained staff. There is no shortage of health facilties since the health centers are built close to each other. Even people from neighboring Oromia and Sidama regions are using our facilities.

4. Do you participate in any meetings, workshops, trainings, pregnant women conference, family, community, government, or other social networks organizations? Please tell me more.
   a. Please tell me how your participation in these organizations affect the PP and PA family planning services provision

When this program is being launched, I took the training at Shashemene and it helped to give more emphasis on "PP and PA family planning". It has helped me to give attention related with this issue even though I know it previously.

5. In your Woreda do women assume leadership roles? What types of roles? Do these roles change by social characteristics, such as (dis)ability, economic status, health status, educational level, religion, culture, geographical location, and marital status?
   a. Please tell us about your own experience with assuming any leadership roles?
   b. Please tell me how your leadership roles affect the PP and PA family planning services provision

Recently even health extension workers are being promoted and given leadership roles. Women lead in women and children affairs, woreda house speaker, and public service. But I don't know whether it

affects PP and PA family planning since we never had discussion on such matters. We didn't have meeting together to talk about it.

At the health facilities women prefer women's to get the services. Women are closer to women. the feel free to talk to each other. When they talk to each other they feel free. When I assumed leadership roles I encouraged female staff members to support women more because they easily tell their secret to them. So, I think bringing female focal persons would help to address the problem of women. I helped to bring a lady called "z" to bring to the leadership position at the woreda level.

6. In your Woreda/community who decides at what age people marry? What are the reasons for getting married at younger or older ages in this Woreda?
   a. Please tell me how their age at marriage affects their PP and PA family planning services use

This thing "age people marry" has changed from time to time. Previously in old times parents decide their age of marriage, but now they decide by themselves. Previously girls marry under the age of 16, but now most of them are marrying after 17 years. Most of the girls go to school and marry late. But in lowlands due to economic reasons, some of them marry at lower ages like 15 or 16 years.

When people get married, they usually give birth. But, we account them in to our account in addressing their needs.

7. What are your suggestions to improve women's roles and participation to enhance the use of PP and PA family planning services?

There is a good awareness regarding FP, but there is poor awareness about PP and PA family planning. So, people should be trained at lower levels. Health extension workers should get trained and train others at lower levels. The plan of 10% from the federal gov't is also low. If the plan is low, there is a low commitment to achieve that. When the plan is higher, there is a high commitment. So, the plan should also be improved.

Knowledge, Beliefs, and Perceptions

Now we are going to discuss about the issues faced by women in accessing PP and PA family Planning services in your opinion.

1. In your Woreda what is appropriate behavior for a woman after delivery and abortion regarding the use of PP and PA family planning? How do they differ from adolescent girls and young women?
   a. How do these believe influence health behavior including the PP and PA family planning use?

(From 1:03:24-1:04:25 is not relevant since the informant is talking about what should be done).

The community is not resistance to change. There is a growing acceptance to use family planning. There is an increasing acceptance to family planning.

2. What are the social beliefs and perceptions that share women's expectations and aspiration for health care including PP and PA family planning?
3. In your opinion who should make decisions regarding use of PP and PA family planning services? What types of decisions do women make regarding use of healthcare including PP and PA family planning services? What types of decisions regarding use of healthcare including PP and PA family planning services are made jointly?

They "couples" should make decisions. Some husbands go in fighting with their wives after checking the arm for the existence of implanon. So, some mothers avoid it and use depo instead. So, husbands involvement should be encouraged and they should make a joint decision.

Legal Rights and Status

Now we are going to discuss about the marriage related process prevalent in this community.

1. In your community are women who delivered recently legally allowed to receive PP and PA family planning service?
   - Is these same for boys and girls, people from different age groups, education background, ethnic groups, socio economic classes, excluded groups? How are they different? Why/ Why not?

There is no law that prevents them not to use family planning. But there are some care providers perception who prevent young ones from not using family planning. besides there are no legal barriers.

2. How do recently delivered/aborted women access PP and PA family planning information and resource from the healthcare facilities?
   - Is these same for boys and girls, people from different age groups, education background, ethnic groups, socio economic classes, excluded groups? How are they different? Why/ Why not?
   - Do the people in your community support recently delivered/aborted women to access PP and PA family planning information and resources? How do they provide support? Who are these people? Why do they provide support?
   - Are there certain groups in your community who do not support this? Who are these people? Why don't they support?

Most of women get information from health extension workers. HDAs also provide information. At the health facility level, they get information from the health care providers.

When there are some problem associated with family planning like insertion problem, or when someone comes up after becoming pregnant while using FP, they say it is not useful use family planning. we have seen such cases in Edo site. It could be due to failure or problem from prescreening. When such things happen people could think it is not useful. Besides there is no such an organized group against it.

3. Please tell me women's experience with accessing healthcare information and resources including PP family planning.
   - Do you feel they have a right to get healthcare information and resources including PP family planning services? Why/why not?
   - Do you think women's experience is different from other people in your community? How? Please tell me more
   - Is there legal mechanism you can follow to ensure they get quality healthcare information and resources they need? Please tell me more

Yes we have a legal mechanism we use to follow. We use a monthly report DHIS and we follow it. We have online and off line data and we monitor using this available data. We evaluate monthly and we take actions on the reasons for lower uptake after identifying them.

With this we have come to an end of the discussion. Do you want to add anything before we end the discussion or if you have any questions you want to ask?

Thank you for your time and in case you want, you can get in touch with us through the details mentioned in the consent form.

****

Summary

This interview was conducted in Bitana town, the administrative capital of Duguna Fango Woreda. The interview was conducted at his office and the place was quite. To reduce the flow of staff members or other customers we closed the door until the end of interview. The informant was confident and well aware, however sometimes he bends towards the training effect on PP and PA family planning. the interviewer tried to gear the focus towards before the intervention status. Generally I believe it is a detailed and informative session.

Interview details

**Interviewee code**	KII_GA_HH_270622
**Sex**	Female
**Age**	28
**Job title**	Head of Health center/Delegated
**Name of interviewer**	Beimnet Desalegn
**Date**	27/06/22
**Time started**	3;42lt
**Time finished**	4;23lt
**Venue**	Gacheno Health center

Assets and Resources

Discussion

Now we are going to discuss about the Healthcare resources and assets available for provision of PP and PA family planning services in your area.

I: - Health services (PP and PA family planning services) availability: Can you tell me the types of healthcare facilities that provide PP and PA family planning services in your area?

a. What types of FP services are available at the healthcare facilities?
b. Are these services equally available to boys, girls, young, old, married, unmarried, people from different geographic locations, religious or cultural background, language etc.? Please tell me more

***R: -** here in our health center, we are providing these services for users now …. We have trained health workers …. They took training in Halaba. After training, the trained health workers oriented other health workers on the job … since then we are serving our community. And we will start reporting by this month… yeah, we have all the required equipment. But we often face a shortage of depo … we have enough stock of other options like Implanon/implant, pills, and IUCD. Sometimes depo stock disappears for more than four months…*

We had only one trained person in post-abortion care, for a long time, we were providing service for users here… recently I myself also trained on post-abortion care in Halaba. But I am not actively working on abortion care so I don't know details about the equipment we have

there. now I am requesting the health center's administrating body to... because the previously assigned person is not working actively there ....

Previously engender health staff, directly took report through phone call… but now we are reporting abortion issue with our health center reporting … now the reporting included in health center reporting system.

No, we just provide service to users without looking at any background history of the client … we only consider their health condition and provide for them the appropriate drug considering they chose. We counsel them about each method with its advantages and disadvantage.… then the client chooses a method. We provide their chosen method…

I: - Accessibility of the PP and PA family planning services

c. Can you tell me how far the PP and PA family planning services providing facilities are located? How long does it take for women to access the facilities?
d. What type of transportation means do they use to reach the facilities? How easy or difficult is it to use the transportation services?
e. Is there any cost associated with accessing the PP and PA family planning services? Please tell me more about this
f. Is the PP and PA family planning service is accessible at all time at the healthcare facilities? Are there types of FP methods of users' choice in the healthcare facilities?
g. Are these services equally accessible to boys, girls, young, old, married, unmarried, people from different geographic locations, religious or cultural background, language etc.? Please tell me more
h. How easy could they access the PP and PA family planning services? Do you think that their access to the PP and PA family planning services are affected because of their background? How?

R: - *yeah, the furthest kebele is … do you know Boditi hospital …. Yeah near to the hospital there is a border there, so that is our furthest Kebele and Gacheno town is the nearest Kebele.*

For delivery service they come here by ambulance … when they call for service ambulance. Sometimes for post-abortion care, they come here. Often many safe abortion care sicker come

from neighboring kebele because they fear getting service in their kebele. Even often from Buge we have clients for safe abortion care.

Ade koysh is the furthest kebele. we have many clients come to us for family planning and for other health care services from there. They use transportation to come here… yea means of transportation they often use were a car or public transportation. All the kebele under our catchment are not far from the main road so they can access it easily. Taba kebele is the only kebele out of the main road. clients come from there by their feet or sometimes they use other means of transportation like a horse cart.

Our clients do not pay for service … all MCH services including family planning, we provide without fee … sometimes what we face is a lack of fluid and surgical gloves absence, at that time we prescribe to buy from a privet pharmacy. That is not always, but sometimes we use this means and at that time they may expend some money. ... As I told you we have also a shortage of depo… the good thing is many clients were shifting to long-acting contraceptive methods.

Yeah, they can get service 24 hours, and … we are working an 8-hour shift for 24 hours… but the challenging thing is only one person were assigned for one shift in MCH, and sometimes when they have a delivery case and attending the delivery those who came for family planning may wait for a prolonged time. Usually, our family planning users come during day time…

I: - Means to access the PP and PA family planning services

i. Please tell me about your facilities' resources and assets. Please tell me the resources and assets belong to these health facilities to provide PP and PA FP service.
j. How is the use of the facility/Woreda health office resources and assets decided? Who makes the decision? Do you involve in the decision making? How do you think that affects access to the healthcare services including PP and PA family planning by women?
k. Please tell me about income of in this Woreda. Please tell me about your woredas income.
l. How is the use of the woreda's income decided? Who makes the decision? Do you involve in the decision making? How do you think that affects their access to the healthcare services including PP and PA family planning?

m. In your woreda how is use of PP and PA family planning seen? Is it acceptable for women to use PP and PA family planning? Why/why not? Please tell me more (what about boys and girls)

R: - yeah, a place, where we are providing service for clients, is … in my opinion it's sufficient, well-spaced … but as I told you, we have a shortage of short-acting family planning methods. I have seen clients who are rejected when we ask them to use the long-acting contraceptive method after coming to use the contraception method. Now we don't have depo … as I heard from the user they are using from a private clinic and paying up to 150 birrs for depo …. but Here it was free if fee…

We participate in resource or asset allocation, ... the supply comes based on our request from the government or supporting body. then health workers here collect from our store in our health center. family planning we get from donors so just they bring based on drug store man request. What our drug store man do is report to the health center administering body when our stock run out.… just report to them when our stock run out.

Here most of the residents are farmers… except those who serve the government in service rendering institutions. So their income is not such much here in our community…

As an MCH department, we do not generate income … all the service under MCH is free of fee … most of the service users use MATEMA … as institution we don't have cash …

family planning service is acceptable … I don't know about post abortions cases… I am not involved yet. previously, we do not give family planning immediately after birth,... what we were doing s appoint them to come back after 45 days while coming for vaccination for her baby. But now, we are counseling mothers starting from their ANC visit and we provide family planning after having birth, before leaving the health facility, and going back to their home.

Yeah, sometimes mother asked us come back after discussing with her husband … as some of them do not agree to use family planning. Even some male partners do not allow them to use contraception methods. There are different perceptions in the community …

Yea, young girls are not volunteering to use contraceptives after post-abortion… as I heard from my workers providing services here… they say that "I would never do sex again … this is the last for me".

I: - In your opinion what should be done to improve access to the PP and PA family planning services for all women in your community?

R: - regarding the young girls' issue I raised before, we have to educate them at school or in the community, about reproductive health issues, to change their perception, and to use of contraception methods. We have had youth-friendly service (YFS) here before… at that time they were doing well. After a few years, that service collapsed and supporting NGOs' path out and service stopped.

I said about youths … and to improve mothers' use of PP and PA family planning we do have to strengthen women forums which are held every month and educate them about family planning. Most of the time married women did not hesitate to take contraceptives but their problem is they did not want to use long-acting contraceptive methods. The community's perception of long-acting contraceptives is not good … so we have to educate them during the forum.

Practices, Roles, and Participation

Now we are going to discuss about your role and responsibilities.

1. Please describe to me the typical Woreda health office officials/experts' roles in your Woreda/community?
   a. How is Woreda health office officials occupied over the course of the day? Are there seasonal differences (campaign/political assignment) in how you use time?

R: - okay, as a leader of a service rendering office my first role is assuring the availability of required supplies to provide family planning services for the clients who come to our facility for family planning. also evaluating the program at the health center… and organizing appropriate training for health workers on any available updates of family planning methods.

Yea, our workers have a work burden… as the health center we have only three midwife professionals… we are working in three-shift in 24 hours … I think the standard is six. We are six in number before … some were joined other institutions. Other professionals … nurses and public health workers do not want to join/help this case team.

I: - As Woreda health office official, what kind of work do you do? Do these works different when facility status changes (Rural, urban and geographical locations)?

R: *- as head of health office and health professional follow up of health center and evaluating then putting direction to correct in case of poor achievements.*

I: - Do Woreda health office officials like you have restrictions/barriers on the accessibility? What restrictions/barriers? How do the restrictions/barriers influence the access of the PP and PA family planning services and supportive social networks?

b. In your Woreda do the restriction/barriers change by social characteristic, such as (dis)ability, economic status, health status, educational level, religion, culture, geographical location, and marital status?

R: *- yeah, there are even health professionals who didn't consider post-part family planning as appropriate behavior… this may have an effect on PP family planning coverage and use by clients.*

Some of the clients specifically youths who are not married yet do not want to use family planning services here … that is because we know each other. so, they fear rejection by the community since the community culturally did not allow sexual relations before marriage.

I: - Do you participate in any meetings, workshops, trainings, pregnant women conference, family, community, government, or other social networks organizations? Please tell me more.

c. Please tell me how your participation in these organizations affect the PP and PA family planning services provision

R: - yeah, regarding post-abortion and post-partum family planning … I am participating in women's conferences as a professional. I am educating about PP and PA family planning services. Our participation also changed the women's perception of the use of PP and PA family planning services.

I: - In your Woreda do women assume leadership roles? What types of roles? Do these roles change by social characteristics, such as (dis)ability, economic status, health status, educational level, religion, culture, geographical location, and marital status?

d. Please tell us about your own experience with assuming any leadership roles?
e. Please tell me how your leadership roles affect the PP and PA family planning services provision

R: - *ok, … long time ago I know a woman who was head of this health facility … she was a nice and hard worker at the time… I know her… now Buge health center head is a woman. … eee also I know here in our health center are case team leaders… in the health center.*

I don't know how they appoint for leadership positions… but most of the time they consider the level of education. ….

I: - In your Woreda/community who decides at what age people marry? What are the reasons for getting married at younger or older ages in this Woreda?

f. Please tell me how their age at marriage affects their PP and PA family planning services use

R: - *most of the time by 15 years and above… I haven't seen anyone married under the age of 15. most of the time a woman decides by themselves … now there is no family push.*

Yeah, they have to use contraceptives… most of the time youth feel free to use services even when they come together with their male partner. In my opinion, youths use services better than the older ones who are using family planning. Those who marry late prefer to use contraceptives after having birth.

I: - What are your suggestions to improve women's roles and participation to enhance the use of PP and PA family planning services?

R; - *empowering women to decide for themselves on issue concerning them and creating awareness about reproductive health related issue.*

Knowledge, Beliefs, and Perceptions

Now we are going to discuss about the issues faced by women in accessing PP and PA family Planning services in your opinion.

I: - In your Woreda what is appropriate behavior for a woman after delivery and abortion regarding the use of PP and PA family planning? How do they differ from adolescent girls and young women?

a. How do these believe influence health behavior including the PP and PA family planning use?

R: - *there are different perceptions some think that … their fertility returns after two years… so they do think that they can stay for two years so they do not consider using contraceptives. Most of the time those mothers do not volunteer to use contraceptives postpartum. But they return with pregnancy before a year. At that time, we use those mothers to share their experiences with other women during the forum.*

I: - What are the social beliefs and perceptions that share women's expectations and aspiration for health care including PP and PA family planning?

R: - *Generally, many women perceive that using contraceptive methods immediately after birth may harm them. Some women believe that contraception users should feed good nutritious food … so those women hesitate to use the contraception method mentioning their living conditions. "I don't have anything to eat so taking this drug may harm me," they say. This is a widely distributed perception in our community.*

I: - In your opinion who should make decisions regarding use of PP and PA family planning services? What types of decisions do women make regarding use of healthcare including PP and PA family planning services? What types of decisions regarding use of healthcare including PP and PA family planning services are made jointly?

R: - *first, the women should have to decide by themselves to use contraceptive… because she is the one who faces the consequence of frequent birth-related health problem. But sharing and discussing with male partners is good for a healthy relationship in the family. As I told you…*

some husbands did not allow women to use contraceptives … I know a woman, whose husband brought her here to discontinue a method she is using… also this is the reason for many women to prefer depo… sine depo is invisible and she can take a while she comes to market and out of home for other purposes. Here there is a high influence of husbands on women… its better even if we increase/involve male partner participation in family planning education.

Legal Rights and Status

Now we are going to discuss about the marriage related process prevalent in this community.

I: - In your community are women who delivered recently legally allowed to receive PP and PA family planning service?

- Is these same for boys and girls, people from different age groups, education background, ethnic groups, socio economic classes, excluded groups? How are they different? Why/ Why not?

R: - *yes, … there is an encouraging policy as I know… family planning methods are legally allowed for recently delivered and aborted women.*

I: - How do recently delivered/aborted women access PP and PA family planning information and resource from the healthcare facilities?

- Is these same for boys and girls, people from different age groups, education background, ethnic groups, socio economic classes, excluded groups? How are they different? Why/ Why not?
- Do the people in your community support recently delivered/aborted women to access PP and PA family planning information and resources? How do they provide support? Who are these people? Why do they provide support?
- Are there certain groups in your community who do not support this? Who are these people? Why don't they support?

R; - I think I told you before … they get information about contraceptive methods or PP and PA family planning during women's forums… also from health development army leaders. Health

extension workers also disseminate information about the service. Most women use family planning services from health posts…

There is a woman who did not understand the information easily… and some are active and understand things easily… so we provide information considering their level of understanding.

Yeah, there is. There is 1 to 30 team, again 1 to 5 team and they support each other and share information among them, including the good experience of the model woman. Team leaders communicate with health extension workers and supervise those under their team.

I: - Please tell me women's experience with accessing healthcare information and resources including PP family planning.

- Do you feel they have a right to get healthcare information and resources including PP family planning services? Why/why not?
- Do you think women's experience is different from other people in your community? How? Please tell me more
- Is there legal mechanism you can follow to ensure they get quality healthcare information and resources they need? Please tell me more

R: - *yeah, it is their right to get full information about PP and PA family planning before using any contraception methods. Because she has the right to choose a method … no one has the right to enforce to use of contraception methods without her permission.*

Regarding information quality they have gathered from different sources… our health workers assess and correct when a woman comes for use a contraception method … during a consultation, they dig out what she thinks or what information she has about a contraceptive method and correct them with appropriate information. So, during counseling, health workers can understand whether the information they have got before is correct or rumors …

With this we have come to an end of the discussion. Do you want to add anything before we end the discussion or if you have any questions you want to ask?

Thank you for your time and in case you want, you can get in touch with us through the details mentioned in the consent form.

****

Summary

This interview was conducted in Gacheno health center with the delegated head of the health center. The informant was from MCH case team and delegated by head of health center during our survey. She was confident and well aware of the PP and PA family planning program. In general, the discussion was detailed and informative.

Discussion

Rapport Building

Thank you for proving consent to participate in the discussion. There are no right or wrong answers, please share your frank opinion, as it will help us in understanding the situation better.

Enquire about her recent delivery experience. An example is given below:

Please tell us about family planning service in your woreda. Where did women get family planning services? Who provided the services? How far is the health facility from women's home (in different directions? How do women travel to the health facility?

Assets and Resources

Discussion

Now we are going to discuss about the Healthcare resources and assets available for provision of PP and PA family planning services in your area.

1. Health services (PP and PA family planning services) availability: Can you tell me the types of healthcare facilities that provide PP and PA family planning services in your area?
   a. What types of FP services are available at the healthcare facilities?
   b. Are these services equally available to boys, girls, young, old, married, unmarried, people from different geographic locations, religious or cultural background, language etc.? Please tell me more

In our locality health posts can't give immediate PP family planning. They refer the cases to us "HC" and we try to give to the mothers' immediate PP family planning. Implanon and jaddle can be given at the health post level, but women can come to us to receive them if they don't find HEW at their place. So, both health post and HC provide family planning. At the health post there is COC/POP pills, jaddle, implanon, depo. At the health center there is and IUCD, COC/POP pills, jaddle, implanon, depo.

The service is available for everyone. For males there is no vasectomy service, but there is a condom here so that they can use it.

2. Accessibility of the PP and PA family planning services
   a. Can you tell me how far the PP and PA family planning services providing facilities are located? How long does it take for women to access the facilities?
   b. What type of transportation means do they use to reach the facilities? How easy or difficult is it to use the transportation services?
   c. Is there any cost associated with accessing the PP and PA family planning services? Please tell me more about this
   d. Is the PP and PA family planning service is accessible at all time at the healthcare facilities? Are there types of FP methods of users' choice in the healthcare facilities?
   e. Are these services equally accessible to boys, girls, young, old, married, unmarried, people from different geographic locations, religious or cultural background, language etc.? Please tell me more
   f. How easy could they access the PP and PA family planning services? Do you think that their access to the PP and PA family planning services are affected because of their background? How?

There are 6 health posts in our catchement. Engender health has included for health posts. They are Dagaga Lenda, the neary by one "near the health center" is not included, Mundaja Sake, Daye Sake, and Koyo Sake. The farthest of all is Daye Sake for this health center if they want to come here. If not, they

can use from the nearby six health posts. All health posts have all the options except IUCD. They can use either the health post or the health center.

They will not pay anything for the service. Regarding MCH there is no cost associated with it. If they want to use motorcycle, they may pay for that. Health posts are always open to serve, but during the campaign time they may be close and mothers may come to us. Health posts are closed at night and they can't get at night. At night the FP service is not given except PP "immediate" at the health center as well.

We give mothers the options available at the health center and we give them a chance to chose among them. There is some misconception on some methods like the say implanon something like "thin wood", but we explain them is in not something like wood, rather is a plastic material. We gather women and show they it is not a wood like thing. We explain the ingredients they contain and tell them the options.

We usually have all methods available, except last time we didn't have depo. We have always implanon, jaddle, IUCD. If it runs out, EPSA will bring to us immediately.

We provide the service for everyone who wants to use the service. Young girls usually goes to another place to get such service including CAC. Now we have a trained professional on that "CAC", but they still go to another place to get the service. If they want to use depo, we give them the service.

3. Means to access the PP and PA family planning services

a. Please tell me about your facilities' resources and assets. Please tell me the resources and assets belong to these health facilities to provide PP and PA FP service.
b. How is the use of the facility/Woreda health office resources and assets decided? Who makes the decision? Do you involve in the decision making? How do you think that affects access to the healthcare services including PP and PA family planning by women?
c. Please tell me about income of in this Woreda. Please tell me about your woredas income.
d. How is the use of the woreda's income decided? Who makes the decision? Do you involve in the decision making? How do you think that affects their access to the healthcare services including PP and PA family planning?
e. In your woreda how is use of PP and PA family planning seen? Is it acceptable for women to use PP and PA family planning? Why/why not? Please tell me more (what about boys and girls)

We have most of the resources, but we don't have sponge forceps and a device that we use to open the cervix. We asked the concerned office to bring us those missing materials. The staff member who took the training is also processing transfer to another place. And also for abortion service we need a separate room which is lacking now. We have also shortage of MVA.

We don't usually engage in decision making. There are three staff members including the one who took CAC training. But when we have shortage of resources, we report to the concerned body. We strive to get from other facilities if they have extra materials.

"The informant couldn't understand about the income and resource decisions and she was feeling discomfort and she didn't understand this part. So, I skipped to the next questions"

If there is a post abortion service is available here always, I think girls would utilize that. May be some of the mothers are not willing to use PP or other forms of family planning because there are a lot of rumors in the community who are dissemination information women not to use FP. They say even God will punish them if they use FP. So, some women say they don't want to use FP as result of such things and their husbands complain about using FP. There are also some women who shift the place of implanon from one arm to another to hide from their husbands. Some women even say if they insert implanon it will migrate to the back side and other parts of the body. Other women complain it is

causing much pain arunf their wrist and they don’t want to use it anymore. There are a lot of women with different views. Some say God’s wrath will come up on them if they insist using family planning. There are some women who resist despite provision of a lot of information. They come and cry for removal of the implanon from their arm. But, I think if we consistently give abortion care here, I believe a lot of young girls would come to use the service. There is a private clinic nearby and at Sodo when they use abortion service and then after they will use PA family planning.

Sometimes there are some young unmarried girls who come at late afternoon or Sunday asking for some family planning methods.

4. In your opinion what should be done to improve access to the PP and PA family planning services for all women in your community?
5.

It is shortage of time, but I believe women would you use the service if both husbands and wives are counseled together about FP . Most of the women fear their husbands to use the service. They fear their husbands would beat them if they use it. Husbands say it cause a lot of pain and emaciation if they take contraceptives. They warn their wives not to visit health centers of health care providers. So, couselling to husbands would make a big change in the community. If husbands taught about the benefits of using FP such as having a good and healthy family, they would agree about using FP.

There are some women who use FP despite husbands fear. For some women husbands are willing and for others they are not willing. For women we can counsel them and change their mind, but for husbands it is difficult and I suggest you “implementers of this project” counsel them and I hope they will listen to you and change their mind.

Practices, Roles, and Participation

Now we are going to discuss about your role and responsibilities.

1. Please describe to me the typical Woreda health office officials/experts roles in your Woreda/community?
   a. How is Woreda health office officials occupied over the course of the day? Are there seasonal differences (campaign/political assignment) in how you use time?

MCH focal works on checking the proper registration of documents, whether the necessary equipment and materials are ready to support the coming delivery such as gloves, NS, and other materials are ready. Fulfilling what is missing and monitoring the smooth working environment. Sometimes some staff members miss their duty and I have to replace and give care as well. Sometimes it is difficult during campaigns to provide services, because you have to work in lot of departments such as emergency, OPD, ANC and other areas.

2. As Woreda health office official, what kind of work do you do? Do these works different when facility status changes (Rural, urban and geographical locations)?
3. Do Woreda health office officials like you have restrictions/barriers on the accessibility? What restrictions/barriers? How do the restrictions/barriers influence the access of the PP and PA family planning services and supportive social networks?
   a. In your Woreda do the restriction/barriers change by social characteristic, such as (dis)ability, economic status, health status, educational level, religion, culture, geographical location, and marital status?

There are religious people who prevent people from using family planning. As I have told you husbands also restrict women from using family planning. we usually tell the potential side effects such as excessive bleeding can occur during menses. There are some women who complain of lack of food and milk at the house if they want to use FP. They say they have to eat and drink well to use FP. There are a lot of husbands who warn and threaten their wives if they even come to the health center. Hubands say they will not take to the health facilities in case they develop any health problem as they use health problems.

We heard such things when we visited Dagaga Lenda health post. Most of the mothers educated up to grade 8 are easy to counsel and they use the service. Most of the women prefer implanon than Jadele. They fear IUCD. They are so scared of using IUCD for 12 years. They don’t want even to hear about it.

4. Do you participate in any meetings, workshops, trainings, pregnant women conference, family, community, government, or other social networks organizations? Please tell me more.
    a. Please tell me how your participation in these organizations affect the PP and PA family planning services provision

I participate in women conference and hear a lot of things. We have been working on women conference even before we took the training ”by Engender”. We meet for a brief moment like 30 minutes because if we stay longer they will start complaining. We counsel pregnant mothers to come to the health facilities and give birth there. When we mention the name abortion “Bosha”, they shout on us not to say it in front of a pregnant mother. They say they will come in case they face any health problems, but they don’t want to heat the name abortion.

5. In your Woreda do women assume leadership roles? What types of roles? Do these roles change by social characteristics, such as (dis)ability, economic status, health status, educational level, religion, culture, geographical location, and marital status?
    a. Please tell us about your own experience with assuming any leadership roles?
    b. Please tell me how your leadership roles affect the PP and PA family planning services provision

At the school level, there are students attending Saturday class on adolescent health. Female teachers counsel girls and help them if they have any problems related with unprotected sex and unwanted pregnancy. They bring girls and boys as well.

6. In your Woreda/community who decides at what age people marry? What are the reasons for getting married at younger or older ages in this Woreda?
    a. Please tell me how their age at marriage affects their PP and PA family planning services use

There are early marriages in our community. There is a girl 16 years old who gave birth at that age. There are a lot of girls who marry early and willingly. The reasons are lack of future plan and commitment. They want to get mobile phones, clothes and shoes easily. They are deceived by these things to get married. Some girls marry early because of lack of someone to support their school materials and clothes.

If the husband is educated and decides to delay pregnancy they may use some methods like natural ones. But most of the girls became pregnant immediately after getting married. They will not stay a while.

7. What are your suggestions to improve women’s roles and participation to enhance the use of PP and PA family planning services?

To increase women's participation all health care providers should show a good face and treat them well. even if they don't bring their cards, women should be able to be supported to get the service. If they are not getting a good treatment, they will be not happy to come. If they see a bad face, they will tell to other women in the community.
Another important thing is teaching women again and again. In the kebele I support, almost all women are using FP because I teach them well. I strongly encourage them to use in a friendly way.

Knowledge, Beliefs, and Perceptions

Now we are going to discuss about the issues faced by women in accessing PP and PA family Planning services in your opinion.

1. In your Woreda what is appropriate behavior for a woman after delivery and abortion regarding the use of PP and PA family planning? How do they differ from adolescent girls and young women?
    a. How do these believe influence health behavior including the PP and PA family planning use?

Previously women start to think to about FP after 45 days. Some women don't come to use it and will not come to the health facilities even. Some women return back to use it after delivery.

2. What are the social beliefs and perceptions that share women's expectations and aspiration for health care including PP and PA family planning?
3. In your opinion who should make decisions regarding use of PP and PA family planning services? What types of decisions do women make regarding use of healthcare including PP and PA family planning services? What types of decisions regarding use of healthcare including PP and PA family planning services are made jointly?

Mothers should make decisions because she is the one who suffers a lot due to repeated deliver. But husbands should support her decision. Some husbands discourage women not to use any family planning.

Legal Rights and Status

Now we are going to discuss about the marriage related process prevalent in this community.

1. In your community are women who delivered recently legally allowed to receive PP and PA family planning service?
    - Is these same for boys and girls, people from different age groups, education background, ethnic groups, socio economic classes, excluded groups? How are they different? Why/ Why not?

There is no law that prevents not to use family planning.

2. How do recently delivered/aborted women access PP and PA family planning information and resource from the healthcare facilities?
    - Is these same for boys and girls, people from different age groups, education background, ethnic groups, socio economic classes, excluded groups? How are they different? Why/ Why not?
    - Do the people in your community support recently delivered/aborted women to access PP and PA family planning information and resources? How do they provide support? Who are these people? Why do they provide support?

- Are there certain groups in your community who do not support this? Who are these people? Why don't they support?

There are some kebele leaders and educated families who encourage mothers to use family planning. They advise women to use it. But there are also other people who talk against using FP. Such people are usually drunk people and those who spend of their time in unproductive ways. There are some mothers and fathers who are well educated and who taught their children well to higher levels. Especially there are people who preach in the community not to use FP. Those people are even not known by the church. They are "illegal" preachers here and there.

3. Please tell me women's experience with accessing healthcare information and resources including PP family planning.
   - Do you feel they have a right to get healthcare information and resources including PP family planning services? Why/why not?
   - Do you think women's experience is different from other people in your community? How? Please tell me more
   - Is there legal mechanism you can follow to ensure they get quality healthcare information and resources they need? Please tell me more

There are no cards at the health post and they give them a piece of paper for appointment. We ask mothers if they come to us and verify they type and date of family planning they are using. If they are not sure, we check HCG and give FP for those who are eligible. We have also HMIS professional who shares the information from the health center and health posts.

With this we have come to an end of the discussion. Do you want to add anything before we end the discussion or if you have any questions you want to ask?

Thank you for your time and in case you want, you can get in touch with us through the details mentioned in the consent form.

****

Summary

The interview was conducted at Seke HC, MCH office. First we tried to start at the outside of the office and we changed due to the presence of sound that disturbed our discussion. At the beginning of our interview, the informant thought we came for the evaluation of the project and her performance on the provision of PP family planning since she took the training by Engender health. However, we explained her the purpose of the study again in language that would give her more comfort and freedom by expressing this is simply exploration on the barriers and enables of PP & PA family planning and she wouldn't face any criticism or any negative consequences. We cleared the air before proceeding to the questions. Finally, the conversation went well and she felt at ease throughout the discussion and informed to the questions freely.

Interview details

**Interviewee code**	KII_SO_HC head_161014
**Sex**	Male
**Age**	33
**Job title**	PHO/head of health center
**Name of interviewer**	Beimnet Desalegn
**Date**	16/10/2014
**Time started**	9;09
**Time finished**	10;13
**Venue**	Sodo Health center

Assets and Resources

Discussion

Now we are going to discuss about the Healthcare resources and assets available for provision of PP and PA family planning services in your area.

I; - Health services (PP and PA family planning services) availability: Can you tell me the types of healthcare facilities that provide PP and PA family planning services in your area?

a. What types of FP services are available at the healthcare facilities?
b. Are these services equally available to boys, girls, young, old, married, unmarried, people from different geographic locations, religious or cultural background, language etc.? Please tell me more

R: - *yeah... we are providing PP and PA services in our health center ... after our staff members in MCH were taking training on PP and PA FP they were proving services for the community members.*

Eee ... now here in our health center we are providing for our client .... short acting FP like POP ... eee long acting IUCD, implant, Juedil… but most of the time we face shortage of implant ... we have enough stock for other option…yea… ok that is due to most of our client prefer implant… that is the reason for. We provide based on their interest ... first the provider counsel the client on the available option of FP and live the decision for the client ...

Basically the shortage of implant is due to the supply problem from the heath ... supply and demand is not balanced.

Here in our health center to provide service for client … we don't have any criteria… to get PP and PA FP service having birth/getting delivery service or PA service here in our institution is enough… we did not consider any other things …. Eee … then our health workers counsel the client based on level of understanding … may be their level of understanding may differ based on their level of education, based on their residence, so health workers provide required information about the method and give a method preferred by the client.

I: - Accessibility of the PP and PA family planning services

c. Can you tell me how far the PP and PA family planning services providing facilities are located? How long does it take for women to access the facilities?
d. What type of transportation means do they use to reach the facilities? How easy or difficult is it to use the transportation services?
e. Is there any cost associated with accessing the PP and PA family planning services? Please tell me more about this
f. Is the PP and PA family planning service is accessible at all time at the healthcare facilities? Are there types of FP methods of users' choice in the healthcare facilities?
g. Are these services equally accessible to boys, girls, young, old, married, unmarried, people from different geographic locations, religious or cultural background, language etc.? Please tell me more
h. How easy could they access the PP and PA family planning services? Do you think that their access to the PP and PA family planning services are affected because of their background? How?

R: - *Actually **…** eee as a town … those who are near to the main road can access transportation easily …. Those who is far from the main road has ambulance service that we provide on their request. Those who came for delivery and abortion services in case of incomplete abortion were using motor vehicle or Bajaje and most of the time Ambulance that we provide on their request. We provide ambulance service for all women at any time in anywhere in our catchment area even from the … those from far catchment can use our ambulance service or use the other transportation option.*

Eee now the user to ambulance ratio is not enough to serve or fulfill the need of the user… but we try our best to serve our community with our maximum effort. Sometime we hear complain from users on delay in reaching on time… that is most of the time that is happen when the ambulance is serving other users … to solve this problem we have to work on both side …. First the user should have to inform us as early as possible … and have to be come to us before getting into any emergency situation. That is why here we have many individuals to serve… and on the other hand we as a service provider have to aware the user or community. We have two ambulance serving the community… but the challenging thing here is most of the time they call for ambulance after they getting in emergency condition … after onset of labor or staying long time at their home … in some situation the driver may get difficult reaching with in 30 minute or less. Most of the complaint from the user were due to that….. we provided ambulance service at any time on their request.

No… there is no payment for our service in case of MCH service …. Including PP and PA FP service … FP and delivery services are free of charge…

Yeah … we provide services for all who need our service…. we serve equally… without any discrimination based on their need and chose. Just what health worker did here before giving any FP is providing required information in sufficient and understandable way including advantage as well as the disadvantage of each method for the client.

I: - Means to access the PP and PA family planning services

i. Please tell me about your facilities' resources and assets. Please tell me the resources and assets belong to these health facilities to provide PP and PA FP service.
j. How is the use of the facility/Woreda health office resources and assets decided? Who makes the decision? Do you involve in the decision making? How do you think that affects access to the healthcare services including PP and PA family planning by women?
k. Please tell me about income of in this Woreda. Please tell me about your woredas income.

l. How is the use of the woreda's income decided? Who makes the decision? Do you involve in the decision making? How do you think that affects their access to the healthcare services including PP and PA family planning?
m. In your woreda how is use of PP and PA family planning seen? Is it acceptable for women to use PP and PA family planning? Why/why not? Please tell me more (what about boys and girls)

R: - *when we compare with other health center … PA and PP service here or FP service here is better … as health worker in health center set up this is the first place I see that three components of MCH service were separately provided in different room for the client. Delivery service, service post-natal or ANC service and FP were provided in separated room. We have enough room and working space …*

Regarding required supply … it varies as a season some time we get enough supply … other time we face shortage of some FP options … most of the time as I mentioned before implant … example currently depo shortage is here. We could not say confidently we have enough stoke of FP … also some time we get extra amount .... Sometime we face shortage but it is not for long time … the known shortage is shortage of depo… that is mainly due to high demand of the depo by users.

I: - what was the basic reason behind shortage of short acting contraceptive method?

R: - *the main reason might be the way of requesting for required drugs were not scientific or followed scientific way… the requesting body does not know our minimum stock based on our consuming… there are procedures there to undergo … to know the required need so not sticking to those scientific methods were basic problem here in our health center as well as a zone in my opinion. Most of the time providers were blamed for but I don't believe that they have … to set themselves free of the blame they distribute without evidence more 6han or less than the request. … it's not value less if they request for four hundred but what on the ground is 200 or less… if the requesting is fine and acceptable way the distributer(donner) may also supply in proper way… if the request is illogical which is not considering the existing situation and need … the supplier just give … for example supply remote area health center … like limited number of user in equal amount as health center like sodo town with high amount of FP consumption… then*

shortage of FP may arise. For me what made supplier to distribute in this way were not being ignorant but … the problem is still unsolved and persisted here for long time which is requesting problem of the health institution.

As you know there are national health care financing system declaration … health care facility have the right to use their many they collected after declaration by board. Eee… have to use after declaration. That is for improving service provision for client … that is good, but in my opinion limitation there is … the services here is many type … like there is exempted service, eee… Credit services and services in cash but of it to assure the continuity of service based on the income … we don't know where the balance is…we get the system is not clear… just we collect from user who pay in cash again we lose it in exempted and credit users. May be there is established system but it is not well functioning and not clear for user. Sill now our concern is …This service was considered as very important (exempted service like delivery and FP) … we are also providing them free of charge … as we hear from others … some institution was providing those services with charge due to shortage of budget. As you know even we get FP from donner … we are not getting gloves … we buy it …as you know nowadays it is costly…. Eee… for example to clean the environment… berkina, soap and likes we expend money … but for expend money who should contribute hoe much to assure service consistency should be clearly have to be known. This thing may compromise the service … it is known. Again, we don't have any other source of income … we use what we get from our clients. This is difficult for us as institution but the policy allows us to use our income.

Yeah board … HC board decides how much to use after collecting income and provide for woreda counseling body then they decided.

I don't think that PP and PA services seen as appropriate by our community … but for more detail it requires more investigation and research to talk more … we do not know whether the services were due to counseling effect or by individuals interest we don't know …. People may resist to use or see the service as insignificant if they don't get enough information … they may misunderstand due to lack of information… to change community view towards the service our protentional to convince the client determines … we have to create the condition where …the PP and PA services as issue or set as agenda among the worker as well as among the community members.

I: - In your opinion what should be done to improve access to the PP and PA family planning services for all women in your community?

R: - *For FP supplier…. I advise to take some measure … at least they have to know baseline data from health facility and divide the health institution based on the flow of the user and have to supply based on the case follow … to halt the frequently happening shortage of required FP …*

In general, … eee … in health sector we have to build accountability among the service provider … they have to assess the previous trade … have to reshape the currant service …. They have to plan evidence based eee… purpose based and inclusive effort should be implemented … you are asking for my opinion right …. So, the basic problem is we are not sharing the PP and PA issue as a common goal at different level as well as among the stake holders … all thing what we are doing here is emotion oriented … it is not goal oriented … controlling birth is not the issue or concern of the community … so we are occupied with in day to day emotion-based activity so at the end we fail… to halt this trend, we have to build accountability at different level … also responsibility and creating common goal and sharing with stockholders including community members… also there's leadership responsibility role to convince and involve the community and stockholders. So to attain the goal building system is very crucial … infrastructure, resource like FP and other materials, information system, man power eee … what type of man power required and where based on study and research … as you know recently I expect addition researches on this area soon and … there are study and process of implementing it … it advises or recommends health institution or pregnant mothers to come to health facility for ANC service more than eight times in a month … so ok let us accept it… and in a month we expect more than 40 mothers for first visit … so when we multiply with eight expected visit in a month by single mother … it's about more than 300 visit in a month … as you see we have second, threed and forth visit imagine the number of visit by pregnant mother could we manage it really with this infrastructure and man power… we have known… so our plan should be scientific and logical before implementing any study and new idea… we have to look wisely on issue … we have to wine political view and influence and set us free as a professional… to improve service we have to set us free from political influence …

Again, there is one trained here … which is false reporting …this requires major reform… that trend is killing … it have to be stopped soon … this is very deep rooted trade in health sector. How can we stop it .. it could be by scientific evidence based approach … somebody report then there is man power, required material, resources, money… so when scientifically we audit this and the report exceeds it … the concerning body should have to take correction measure …. rather than appreciating their false achievements. Rather than mare talk we have to take talk to ground and support with scientific evidence.

Practices, Roles, and Participation

I: - Please describe to me the typical Woreda health office officials/experts roles in your Woreda/community?

a. How is Woreda health office officials occupied over the course of the day? Are there seasonal differences (campaign/political assignment) in how you use time?

R: - *yeah … there is one says… eee "if you fail to plane … you planned to fail" so my first role is to planning taking this PP and PA as part of my plan. Eee… and fulfilling required supply for planed activity … the usual way planning is we have target… we plane to achieve our target … and I discus with health workers how to get and reach our clients basically PP and PA … as you know this service were not first line service … it is service provided for the client after they come to us for other service …. Which is delivery and post abortion care … we have created conducive environment for those care service first to get the client. After getting them, we have to council them and provide information … then finally give PF service for them… but I am not looking promising change regarding this service… we are still searching for the reason … why that happen… as I mentioned before we are doing based on emotion not critically thing and planning the activity … because of this we are not observing change at grassroot level. So, my responsibly is planning and as leader of the health center involving in management activities… so I am traying my beat to lead this both activities.*

I: - As Woreda health office official, what kind of work do you do? Do these works different when facility status changes (Rural, urban and geographical locations)?

R: - *yeah from my experience … I was working in rural area as health center head for long time before coming here workload as well as working conditions is different from place to place…*

I: - Do Woreda health office officials like you have restrictions/barriers on the accessibility? What restrictions/barriers? How do the restrictions/barriers influence the access of the PP and PA family planning services and supportive social networks?

b. In your Woreda do the restriction/barriers change by social characteristic, such as (dis)ability, economic status, health status, educational level, religion, culture, geographical location, and marital status?

R: - *for me the big barrier/challenge is workers commitment issue … so how can I improve their commitment … is my big challenge as a head of the institution… it's also time taking to change the existing trade of work. For me every activity accomplishment is based on commitment … also the other issue like supply issues also could be raised as restriction … and this also contribute for not achieving PP and PA FP as planned and our goal.*

Yea the commitment of workers varies with some background characters like educational level … as workers with higher degree and with more experience were more motivated to involve in the works … they were more committed as I compare with those having diploma…

I: - Do you participate in any meetings, workshops, trainings, pregnant women conference, family, community, government, or other social networks organizations? Please tell me more.

c. Please tell me how your participation in these organizations affect the PP and PA family planning services provision

R: - *basically eee…. I got information from … eee… participation in different meeting, workshops… regarding PP and PA … which is professionals but not from local meeting … I got some scientific updates regarding FP. In general as Ethiopian there is one wish… that is when we got position to lead or mange the institution as responsible person we try to contribute our*

part… so we search asses and read different materials to update ourselves … we have different materials as institution we got it from different source and we use it …. as manager I got some important information for my work…

Yeah I was participated at different meeting and discussion with stockholders specifically mothers in the community … that have give me good understanding about the family planning … also I go actual information about the family planning. In addition to information ,… it showed me the direction… in which area should I work or focus to have better achievement or outcome… for instance to improve family planning coverage. As I said before … in two way we might get better outcome or achievement in our work. This participation also helped us to understand PP and PA family planning method and the community perception about it… also helped us to understand community need. We got feedback about our serves to improve service provision.

I: - In your Woreda do women assume leadership roles? What types of roles? Do these roles change by social characteristics, such as (dis)ability, economic status, health status, educational level, religion, culture, geographical location, and marital status?

d. Please tell us about your own experience with assuming any leadership roles?
e. Please tell me how your leadership roles affect the PP and PA family planning services provision

R: - *eee … basically … this health center has been led by woman … and lead department many times … even now … women are led some department and working as focal person … but for more participation I advise them first prefer themselves for such things… for instance if we take MCH department … usually the place by itself occupied by women … so now that department leaders are woman. Even if we did not confidently talk about their participation … some of them were actively contributing their share as our facility worker… but not all of them were active in involving in leadership role … as you know there is big difference taking responsibility by their own interest and by other external body push… so there are some woman contributing well here in our facility. However, they have also limitation … some them were not interested, motivated… and not happy to become to the leading potion. So now here in our facility there is promising movement … and I see it as good side. But when we associate existing change with our target or*

plan still we have to work hard to improve their participation. So we are discussing with concerning body to increase women participation specially in this area.

First of all… there are three thing for man … three basic things to nominate or to propose and assign for a position naturally we weight man by knowledge, attitude and skill… so by considering this we propose for a given position… but even people with good knowledge and skill without good attitude towards the work or clients may not be effective in his work or achieving the goal of the institution. Again in case if having good attitude and lacks knowledge and skill … that alone is also not good or enough to assume position. We did not use others that you mentioned earlier as criteria … we only look competency, should be motivated and with good skill of managing. No one is perfect in his did … I am talking this ideally … on the ground we did not get this all fulfilling person but it should be… and I am using this to assign some one for any potion or duty. I look first for individuals attitude … next does he/she has ability to accomplish assigned work … or does he/she is ready to update himself or herself with updated information and ready to take comments to correct mistakes … if this things are there you can communicate and can bring change.

Eee… actually I have experience in leading health center as well as hospital… I have nine year of work experience and of it I wore eight year in leader potions … starting from remote area. When I was in collage what come to my mind is helping other … so after graduation I started working from remote area to now at this health center… still now my focus is serving in need individuals. … I have one principle haha… that may not work for others …eee 'source for every problem is absence of health' … that is why as public health expert I used WHO definition so whether it is physical or social problem the root cause could be mater of health. So, I share this with others… I believe that we can solve all the problem through working for health. As you know brain development may stop at the age of two from birth so if we did not do something before reaching age of two for baby … what could we do at later age to reverse the effect of food… what if the baby become lazy, none productive and … after completing class and counting grade … he or she use his/her knowledge for useless thing … to hurt community since things were missed from the binging. As you know we under 3rd world … you know what did this … that is all due to our perception towards health and poor effort to improve the health. We can change this through hard working … we cold not change it by good wish or pray. If you ask me for my impact on leadership … I can tell proudly that I foot something in everywhere I was work. I hear

something after I left some institution… I have been health center head for four health facility … I got good feedback… for that the reason was … my commitment to serve community to assure their health.

Surly concerning family planning …. I have concern but still now my focus organizing staff for common goals … to engage them into work … and at the end to get improvement in all aspect of the institution, because there was many good governance issue or problem here… regarding maternal issue my first goal will be making our health center preferable site for delivery and post abortion care with better care and compassion … then the other issue is counselling and providing PP and PA family planning using it as the opportunity… so my first worry is good providing good service and making our facility preferable in delivery and post abortion service. As you asked me … for my impact in PP and PA family planning … as I was new for position here I am searching for supporting staffs to cascade PP and PA family planning issue and organizing them recently … so I expect visible and tangible change in PP and PA FP near soon. I am very happy to serve my community in PP and PA … and ambitious … but it took time even it was not having to took that much time.

I: - In your Woreda/community who decides at what age people marry? What are the reasons for getting married at younger or older ages in this Woreda?

f. Please tell me how their age at marriage affects their PP and PA family planning services use

R: - *from the existing condition …most of the time woman age more than 18 were acceptable age by most of the community member as well as by young girls to get marry. This is my opinion there might be different if the study done… and most of the time youth mothers prefer short acting contraceptive method … very few mothers who are well educated one sometime use long acting contraceptive method. They are few in number … most of the time many mothers prefer short acting contraceptive. As you know long acting requires proper counseling and individual's belief has effect on use … so short acting methods demand is high among our community.*

I: - What are your suggestions to improve women's roles and participation to enhance the use of PP and PA family planning services?

R: - there are good policy as a country … but what lacks is implementation and awareness creation gap as I observe. So, to improve women's role and participation we have to do more in awareness creation, empowering women… women to encourage their participation in economy as a whole in the all existing system we have to involve women … as you know government policy is encouraging and promising. We have or government should have to make them competent to have influence on community as well as within the family… this is from my personal view of point… so it is better if we do in this way… that is why their husband has influence on their contraceptive use … if they have ability to convince their husband … so we have to build their ability.

<u>Knowledge, Beliefs, and Perceptions</u>

Now we are going to discuss about the issues faced by women in accessing PP and PA family Planning services in your opinion.

I: - In your Woreda what is appropriate behavior for a woman after delivery and abortion regarding the use of PP and PA family planning? How do they differ from adolescent girls and young women?

a. How do these believe influence health behavior including the PP and PA family planning use?

R: - yeah, it's difficult to talk about this issue without evidence …. Yea I understand you its qualitative study …. Eee… in my opinion if we provide them with sufficient information they consider it as appropriate … but as usual the community does not allow women to use contraception immediately after giving birth… this is due to this is new for the community. If we provide proper information we can convince them as we are looking know … many women were using it know. There is not such much challenge to convince the community as well as women if we approach them in proper manner. The big issue is do we providing them with appropriate formation or not … that is the case, our community is ready to accept new thing if we provide convincing information.

I: - What are the social beliefs and perceptions that share women's expectations and aspiration for health care including PP and PA family planning?

***R: -** yeah when they hear for the first time about taking contraceptive immediately after having birth… their reaction is not positive … many of them react negatively … as you know previously the same is true for family planning too… through the time things were changing and acceptance were increasing from time to time.*

I: - In your opinion who should make decisions regarding use of PP and PA family planning services? What types of decisions do women make regarding use of healthcare including PP and PA family planning services? What types of decisions regarding use of healthcare including PP and PA family planning services are made jointly?

***R: -** they have decided together for healthy relation in their home …*

Legal Rights and Status

Now we are going to discuss about the marriage related process prevalent in this community.

I: - In your community are women who delivered recently legally allowed to receive PP and PA family planning service?

- Is these same for boys and girls, people from different age groups, education background, ethnic groups, socio economic classes, excluded groups? How are they different? Why/ Why not?

***R: -** we don't have any policy or rule which prevent mothers from using PP and PA family planning … up to my knowledge we have policy which encouraging the user…. There are no excluded groups for services, anyone who need family planning can get services at any time.*

I: - How do recently delivered/aborted women access PP and PA family planning information and resource from the healthcare facilities?

- Is these same for boys and girls, people from different age groups, education background, ethnic groups, socio economic classes, excluded groups? How are they different? Why/ Why not?
- Do the people in your community support recently delivered/aborted women to access PP and PA family planning information and resources? How do they provide support? Who are these people? Why do they provide support?
- Are there certain groups in your community who do not support this? Who are these people? Why don't they support?

R: - *yeah most of the time we expect that most of the community members get information from health workers in the community, from health extension workers. Specifically, those mothers who come for anti-natal care get information about post partem family planning during their visit to health center for ANC care. And youth who need abortion service may get information from their friends… health workers too during their visit. Also, they may get from media … but the most commonly from health extension workers or health care giver during their visit to health institution. ..ee also from health development army who are volunteer in the community and involved information dissemination with health extension workers. Most commonly from health workers … if you take media most the time mothers or women are not consistently following media… they are busy with some other duty.*

Okey… we don't have any formally organized supporting group or promoting group regarding PP and PA family planning service use but everybody here in our institution work towards common goals of the facility so they all support each other… and including this service. All the workers are informed about the services… as team we have MCH case team even this team is working including other services. …eee we do not have specifically established team for PP and PA family planning service … yea here there is no any opposing team in our facility.

I: - Please tell me women's experience with accessing healthcare information and resources including PP family planning.

- Do you feel they have a right to get healthcare information and resources including PP family planning services? Why/why not?

- Do you think women’s experience is different from other people in your community? How? Please tell me more
- Is there legal mechanism you can follow to ensure they get quality healthcare information and resources they need? Please tell me more

R: *- Okay… first of all getting information is the basic right of the client… eee as I told before most of the time they got information from different sources mainly from health workers. This is the decision in their life so that they have full right to get appropriate information before using any method. Its decision in their life as well as health… she has right to space birth… some time there is misunderstanding, husbands force woman to give birth if he economically well… but that is not right because its not having enough money for care but also mother’s health issues … she has right to decide for herself and has legal protection.*

And women’s experience here is almost the same regarding how do they get information as I mentioned above …. The common way of gathering information is health workers and media….

To confirm the information quality … it requires systemic approach in addition to communicating information provider or clients. But we don’t have currently stablished system of controlling or follow up mechanism. We generally check recorded document if the service were provided sticking to standard or not but check documented data is not enough alone … some times we have to check from both sides so we have to aske clients… so we have to set constant survey … we have to supervise regularly … some time I aske mothers who give birth here about the services … we try to check in this way but that is not enough.

With this we have come to an end of the discussion. Do you want to add anything before we end the discussion or if you have any questions you want to ask?

Thank you for your time and in case you want, you can get in touch with us through the details mentioned in the consent form.

****

Summary

This interview was conducted in Sodo twon health center with the head of the health center. It was the first interview of the interviewer in this project. The interview was conducted at his office. When we explained the objective of the research he was very interested. He was also very confident and well aware of the program. In general, the discussion was detailed and informative.

Interview details

**Interviewee code**	KII_WA_WoEx_240622
**Sex**	Male
**Job title**	PHO/head of health coffice /deligated
**Name of interviewer**	Beimnet Desalegn
**Date**	24/06/22
**Time started**	10;22
**Time finished**	11;02
**Venue**	Sodo Zurya Woreda Health Office

Assets and Resources

Discussion

Now we are going to discuss about the Healthcare resources and assets available for provision of PP and PA family planning services in your area.

I: - Health services (PP and PA family planning services) availability: Can you tell me the types of healthcare facilities that provide PP and PA family planning services in your area?

a. What types of FP services are available at the healthcare facilities?
b. Are these services equally available to boys, girls, young, old, married, unmarried, people from different geographic locations, religious or cultural background, language etc.? Please tell me more

R: *- as sodo zurya worda health office, we have four health centers implementing this program... which are PP and PA family planning. If you like to know by name W/Lasho HC, A/Farecho HC, W/Lasho HC and T/Gerera HC… of the six health centers we have, these four-health centers are proving this service, those serving post-partum and post-abortion family planning is … we selected those health center based on a number of populations they serving were large and family planning need is high.*

In all or four health centers, to provide this service, all family planning options are at a health center. We don't have a shortage of contraceptive methods. Specifically, we have enough supply of long-acting contraceptives. We are supplying all options of contraceptives...eee as the need of contraception increasing from time to time … we were providing in enough for health center …

Yeah, the service is available for all equally…. But when we provide this service for the community …. First of all, as we know all service sicker have not an equal level of understanding… yea our health workers consider their client's perception about the contraceptive type and their level of education … during their counseling with their client while providing service for them. They also assess the other existing conditions of the client … up to that level they asses their clients, and give counseling accordingly.

I: - Accessibility of the PP and PA family planning services

c. Can you tell me how far the PP and PA family planning services providing facilities are located? How long does it take for women to access the facilities?
d. What type of transportation means do they use to reach the facilities? How easy or difficult is it to use the transportation services?
e. Is there any cost associated with accessing the PP and PA family planning services? Please tell me more about this
f. Is the PP and PA family planning service is accessible at all time at the healthcare facilities? Are there types of FP methods of users' choice in the healthcare facilities?
g. Are these services equally accessible to boys, girls, young, old, married, unmarried, people from different geographic locations, religious or cultural background, language etc.? Please tell me more
h. How easy could they access the PP and PA family planning services? Do you think that their access to the PP and PA family planning services are affected because of their background? How?

R:- yeah, there is the furthest kebele to the health center are in our woreda. Of those four selected health centers, Tome Gerera health center has kebeles that are far from the health centers. however, the clients from those kebele use a motor vehicle to come to the health center. but they didn't get difficult and they came and used services at the health center. and they are using or getting services still now … as I have information.

Regarding this postpartum or post-abortion family planning service or MCH service in general … as the government put in his policy it is free of fee … as nationally and here in our woreda

too, it is free of charge. Services like delivery care, prenatal and post-natal care, and family planning services are free of fee.

In our woreda in general or in the selected health center selected for this program implementation… in all health centers, health workers are serving the community 24 hours… they are stand by for 24 hours and for 7 days… anyone can get service from health center at any time … and I did not see or hear when they come and fail to get services they want.

I: - Means to access the PP and PA family planning services

i. Please tell me about your facilities' resources and assets. Please tell me the resources and assets belong to these health facilities to provide PP and PA FP service.

j. How is the use of the facility/Woreda health office resources and assets decided? Who makes the decision? Do you involve in the decision making? How do you think that affects access to the healthcare services including PP and PA family planning by women?

k. Please tell me about income of in this Woreda. Please tell me about your woredas income.

l. How is the use of the woreda's income decided? Who makes the decision? Do you involve in the decision making? How do you think that affects their access to the healthcare services including PP and PA family planning?

m. In your woreda how is use of PP and PA family planning seen? Is it acceptable for women to use PP and PA family planning? Why/why not? Please tell me more (what about boys and girls)

***R: -** yeah, regarding the setup or place to provide this service for our client … eee sometimes at health center there are some uncomfortable things …eee even we are providing services for community … sometimes due to the high number of clients or service user … we have … class rooms … we expect to be well spaced and comfortable for client and workers. But they are not connivant for clients and workers … we are thinking about it to set them to the standard. To serve the clients in a good manner we need more investment in building infrastructure… we have to update our health centers and working environment for workers and their clients. However still now we are serving to organize our resources, separating this service from other services … the community is using services. To provide and facilitate services … reasonable … we have*

scarcity of…. eeee equipment and even types of equipment we have are old … so, they need updates.

In the health care financing system… health center uses their budget from the income they generate. The health center finances itself in this way… here at the health office there is organized groups at the health office level here regulating financing and budget issue with a health center. … on budget allocation, we have our part, and also the health center and the kebele representatives have also participated.

I: - In your opinion what should be done to improve access to the PP and PA family planning services for all women in your community?

R: - ok good, this program as a program is very good for our community… eee regarding PP and PA family planning service … when we look at its significance it is very important. Eee… when a woman uses this PP or PA family planning they keep their health too… again can reach her other life goal… so this program facilitates mother and child health. I see it like this it contributes to reducing maternal and child death … also their mental health is kept healthy. So to improve this important service. … as woreda what we are thinking is… using model mothers who are using and benefiting from using this service as an instrument to teach other women in need… we have to work like this. … even though this is an implementation study we have to expand the good experience of those implemented health centers in a better way to other health centers those are not implemented yet. Again, we have to strengthen our relationship with other stack holders and concerned bodies, and NGOs working on family planning to improve PP and PA family planning services in the future.

Practices, Roles, and Participation

Now we are going to discuss about your role and responsibilities.

I: - Please describe to me the typical Woreda health office officials/experts roles in your Woreda/community?

a. How is Woreda health office officials occupied over the course of the day? Are there seasonal differences (campaign/political assignment) in how you use time?

R: - *yeah, for accomplishing this duty as MCH, … eee PHCU directorate or health center heads or even service providing midwives and health extension workers … we all are getting good awareness… we have to hold hand to accomplish the program. … we all have responsibilities, at the health center level head of the health center has its own role and responsibility, and as woreda health office we have our role and responsibility…eee in every health center under our woreda at least three health workers were trained on PP and PA family planning service … including community health extension workers. So, our role is step by step to facilitate the program …*

Yeah, our specific role is monitoring and evaluating health center activity … specifically in PP and PA family planning as woreda health office, we have seen their plan, then monitor and evaluate their activity. And also includes the supply of family planning and other required materials … we asses and support health workers in required skill and capacity building…

I: - As Woreda health office official, what kind of work do you do? Do these works different when facility status changes (Rural, urban and geographical locations)?

R: - yeah, my responsibility is coaching the staff and … evaluating the health center's performance, and communicating with other woreda officials and stockholders.

I: - Do Woreda health office officials like you have restrictions/barriers on the accessibility? What restrictions/barriers? How do the restrictions/barriers influence the access of the PP and PA family planning services and supportive social networks?

b. In your Woreda do the restriction/barriers change by social characteristic, such as (dis)ability, economic status, health status, educational level, religion, culture, geographical location, and marital status?

R: - *yeah, when we engage in the work… there were different challenges at a lower level (health center or community level) … as you know we have to evaluate the activity at the health center level or here at the office, … we have to evaluate the program but sometimes we face logistics scarcity and also budget problems. … also, sometimes we have a shortage of trained professionals in the area like safe abortions care, … that is maybe most of the time the trained*

health workers shift to another facility, like hospitals, and health centers near to the urban. and our health center is in the rural area… and since this site is a program area, we may face a shortage of budget to train again. That is our main concern …

I: - Do you participate in any meetings, workshops, trainings, pregnant women conference, family, community, government, or other social networks organizations? Please tell me more.

c. Please tell me how your participation in these organizations affect the PP and PA family planning services provision

R: - *yeah, we are participating, in different workshops or community meetings. … our participation directly or indirectly has an effect on PP and PA family planning service provision and service use… it also has on clients. During our participation, we pass our messages and promote our PP and PA family planning service. We have weekly command post evaluation at the health center … at that time we evaluate this program plan and achievement then if we get a gap in plan or achievement we put out a direction to improve it. … eee also in public or community meetings… we have our representative participate, either from the health center or health post, so they also pass messages or educate community members about the importance and advantage of this program.*

Yeah, our participation in community meetings and forums helped us a lot in improving the number of family planning users, specifically long-acting family planning methods and postpartum family planning service use...

I: - In your Woreda do women assume leadership roles? What types of roles? Do these roles change by social characteristics, such as (dis)ability, economic status, health status, educational level, religion, culture, geographical location, and marital status?

d. Please tell us about your own experience with assuming any leadership roles?
e. Please tell me how your leadership roles affect the PP and PA family planning services provision

R: - *yeah, women are taking leadership positions at different levels in our woreda… for instance, our woreda deputy administer is a woman, and the women and child affairs office head is also a*

woman at different levels they assume leadership positions. Yeah, they are contributing to facilitating this program and even PP and PA family planning service use by women.

Eee ... ok, regarding my experience .... As we know, before contraceptive users were mainly using short-acting contraceptive methods… but now after observing this at the different health centers and health posts… as a current leading position holder ... discussing and communicating with health workers ... we are working to shift, the users, use a long-acting contraceptive method. ... in this regard, I am contributing my part.

I: - In your Woreda/community who decides at what age people marry? What are the reasons for getting married at younger or older ages in this Woreda?

f. Please tell me how their age at marriage affects their PP and PA family planning services use

R: - ok, ...eee now .... previously there was forced marriage, also because of family economic status they are forced to marry at an early age. eee ... but now most of the women are getting an education and becoming economically strong .... eee women are marrying at the age of 20 or more… so, this is all…

Yeah, ... eee they perceive that if they use contraceptives before having the birth they become infertile and get difficult to become pregnant again ... they perceive that ... if they use contraception when they are young

I: - What are your suggestions to improve women’s roles and participation to enhance the use of PP and PA family planning services?

R: - *Yeah, women are part of society ... but they are vulnerable to different health problems related to maternity. Because of this empowering them is mandatory. Capacitating them economically, building awareness creation centers about reproductive health among the community because about 70% of mothers and children have no access for information… its better if we work hard in this aspect… and this is my view. in this way, we can improve …*

Knowledge, Beliefs, and Perceptions

Now we are going to discuss about the issues faced by women in accessing PP and PA family Planning services in your opinion.

I: - In your Woreda what is appropriate behavior for a woman after delivery and abortion regarding the use of PP and PA family planning? How do they differ from adolescent girls and young women?

a. How do these believe influence health behavior including the PP and PA family planning use?

R: *- in our program area (selected health center for implementation) … it is not practiced very well. It is recently introduced… eee even the time it is given for use is not usual … for instance, IUCD is administered within 10 minutes after removal of placenta … so this is not usual for them… because of this, they see it as inappropriate behavior.*

I: - What are the social beliefs and perceptions that share women's expectations and aspiration for health care including PP and PA family planning?

R: *- Yea, in our culture … Premi mothers, goes to and stay at their family home for more than six months for their first birth, at that time they are hesitant to use contraception mothed… because they do not meet their husband.*

I: - In your opinion who should make decisions regarding use of PP and PA family planning services? What types of decisions do women make regarding use of healthcare including PP and PA family planning services? What types of decisions regarding use of healthcare including PP and PA family planning services are made jointly?

R: *- the decision to use contraception should have to be decided by both… because the husband has influence on here decision … most of the time male partner does not allow a woman to use the contraceptive method … he wants to have many children. Because of this, it's better to involve partners in decision-making.*

Legal Rights and Status

Now we are going to discuss about the marriage related process prevalent in this community.

I: - In your community are women who delivered recently legally allowed to receive PP and PA family planning service?

- Is these same for boys and girls, people from different age groups, education background, ethnic groups, socio economic classes, excluded groups? How are they different? Why/ Why not?

R: - *maybe there is a policy to have an abortion … if a woman has a pregnancy from her close relatives and family member …eee also when they have mental problems… it is legally allowed to discontinue pregnancy… this is a nationally settled policy. So, we serve the community based on this policy …. And we provide post-abortion family planning for those mothers.*

I: - How do recently delivered/aborted women access PP and PA family planning information and resource from the healthcare facilities?

- Is these same for boys and girls, people from different age groups, education background, ethnic groups, socio economic classes, excluded groups? How are they different? Why/ Why not?
- Do the people in your community support recently delivered/aborted women to access PP and PA family planning information and resources? How do they provide support? Who are these people? Why do they provide support?
- Are there certain groups in your community who do not support this? Who are these people? Why don't they support?

R: - *most of the time they get information from health facilities… there are community health extension workers who also disseminate information among the community… yeah, they get information about PA and PP family planning. Also, there are groups like the health development army and 1 to 5 teams … that disseminate information with health extension workers for the community.*

As a community, all the community members do have not an equal level of understanding so there might be no support from a family member, or due to religious beliefs, some people may not support it.

I: - Please tell me women's experience with accessing healthcare information and resources including PP family planning.

- Do you feel they have a right to get healthcare information and resources including PP family planning services? Why/why not?
- Do you think women's experience is different from other people in your community? How? Please tell me more
- Is there legal mechanism you can follow to ensure they get quality healthcare information and resources they need? Please tell me more

R: - women have full right to use this service … and it is their constitutional right to get full information about the service as well as about the contraceptive methods. This is known by all service providers…

we have regular meetings with the health center workers as I told you before and also, we evaluate documents and records ... and also, we do have meetings with community members... we crosscheck the quality in this way.

With this we have come to an end of the discussion. Do you want to add anything before we end the discussion or if you have any questions you want to ask?

Thank you for your time and in case you want, you can get in touch with us through the details mentioned in the consent form.

****

Summery

This interview was conducted in Sodo Zuria health office. We are suffered to get the informant/person very much and after long time of waiting the head of the office send to us the person to give us the information we want. The delegated person was from MCH department in health office and he was trained about PP/PA family planning. The interview was conducted at his office. In general, the discussion was detailed and informative.

Interview details

**Interviewee code**	KII_WO_HH_270722
**Sex**	Male
**Age**	35
**Job title**	PHO/head of health center
**Name of interviewer**	Beimnet Desalegn
**Date**	20/11/2014
**Time started**	8:02 lt
**Time finished**	8:45 lt
**Venue**	Wogera Health Center

Assets and Resources

Discussion

Now we are going to discuss about the Healthcare resources and assets available for provision of PP and PA family planning services in your area.

I: - Health services (PP and PA family planning services) availability: Can you tell me the types of healthcare facilities that provide PP and PA family planning services in your area?

a. What types of FP services are available at the healthcare facilities?
b. Are these services equally available to boys, girls, young, old, married, unmarried, people from different geographic locations, religious or cultural background, language etc.? Please tell me more

***R: -** okay, my name is… and I am head of Wogera health center … eee regarding immediate post-abortion and postpartum family planning service … certainly, before, we conducted forums with mothers and provided family planning when they came to vaccine their baby after 45 days. We provide family planning for those who need to space their pregnancy. But recently after the starting of this program by Engender health, we are offering service in our health center immediately after giving birth or before they leave the health center after delivery. So, we consult the mother during ANC follow-up starting from 3rd visit. we start preparing the mother for this service, and if she agrees we provide their choice of family planning after she has given birth. In this manner, we are working here to serve our community. However, her agreement*

alone has no effect we also tell her to discuss it with her partner or if he comes with her we advise them together.

Regarding the family planning method … we have all options of contraceptives … we get it from engender and also from APSA monthly, directly requesting by RRF for supply. But as Wogera health center we are getting all options of family planning methods but now recently not getting Jadile… we don't have Judile on hand now.

Thanks to God in our health center we have trained health workers by Engender health … so we have knowledgeable trained skill full workers so they provide professional services for users or their clients. They got good skills and they are serving in a good manner … they look at all the clients equally and treat them equally. They are serving all equally without looking at their educational status or religion and cultural background.

I: - Accessibility of the PP and PA family planning services

c. Can you tell me how far the PP and PA family planning services providing facilities are located? How long does it take for women to access the facilities?
d. What type of transportation means do they use to reach the facilities? How easy or difficult is it to use the transportation services?
e. Is there any cost associated with accessing the PP and PA family planning services? Please tell me more about this
f. Is the PP and PA family planning service is accessible at all time at the healthcare facilities? Are there types of FP methods of users' choice in the healthcare facilities?
g. Are these services equally accessible to boys, girls, young, old, married, unmarried, people from different geographic locations, religious or cultural background, language etc.? Please tell me more
h. How easy could they access the PP and PA family planning services? Do you think that their access to the PP and PA family planning services are affected because of their background? How?

R: *- At Wogera health center we have four kebele … the farthest kebele is woshigale, which is away from the main road and difficult to get transportation also its topography is difficult … the*

other kebele Shasha Gale which is also near to Wosha gale and almost similar with washigale in topography and accessing transportation also difficult there too. They come to the health center on foot or sometimes for a pregnant mother who comes for delivery we gave an ambulance service to bring them to the health center. In these two kebele, the topography is difficult as well as getting transportation access also difficult for women living there.

Okay good, regarding service charge… all service related to maternal health is free … also our health center is serving 24 hours … health workers serve the community in 3 shifts… any time you can get service here including holy days and weekend.

Regarding drug supply as I told you before … except for Judiel we have all options of contraceptive methods.

Yeah, we are serving different kinds of people … those who have been educated well and illiterate … so their perception about post-partem or post-abortion family planning is different. Those who have been educated are easily accepting what we advise but others resist believing what we told. Sometimes I have seen those who come from the rural areas for abortion services and after getting service when we advise for contraception they resist using contraceptives. Sometimes many women perceive it as sin…

I: - Means to access the PP and PA family planning services

i. Please tell me about your facilities' resources and assets. Please tell me the resources and assets belong to these health facilities to provide PP and PA FP service.
j. How is the use of the facility/Woreda health office resources and assets decided? Who makes the decision? Do you involve in the decision making? How do you think that affects access to the healthcare services including PP and PA family planning by women?
k. Please tell me about income of in this Woreda. Please tell me about your woredas income.
l. How is the use of the woreda's income decided? Who makes the decision? Do you involve in the decision making? How do you think that affects their access to the healthcare services including PP and PA family planning?

m. In your woreda how is use of PP and PA family planning seen? Is it acceptable for women to use PP and PA family planning? Why/why not? Please tell me more (what about boys and girls)

R; - *regarding health center infrastructure… we have a shortage of room to serve or to provide standard service for our community. We have reported many times concerning body but still, now we did not gotten a response. Now at wogera health center, we don't have room for abortion services we share a delivery room. You can see the room later … it is very small and crowded, with no space.*

Yeah, we are providing family planning and ANC in separate rooms… but as standard, it is better to separate the safe abortion rooms from delivery.

We have a sufficient supply of family planning methods but we don't have a quality delivery coach/bed … we have a delivery coach which served for a long time … maintained many times and old it is not favorable to provide quality service for our clients.

In addition to this as an institution, we are suffering from a lack of water supply and electricity. … after serving clients health workers should have to wash their hands and their gowns, … as well as we have to provide service at night … here we have solar electric but during summer it's not functioning. As I told you we have kebeles which is difficult to reach they don't have road access… also we have a shortage of budget to provide backup service for remote area. We don't have a governmental subsidy, many of our clients are MATEMA service users. Which has its own financing problem or refunding problem which get us into financial difficulties.

Yeah, during budget allocation at the woreda level our health office head participate … and as a health center our budget will be approved by the health center board, we also participate in the board meeting and we have a quarterly meeting with the board members. But we are not getting money in cash, we collect and use collected money based on the budget stated by the board.

Okay, as woreda most of the resident is a farmer and some of them were involved in small businesses.

As this service is new to society, the community has not had enough information about its importance but now we are trying to reach many and promoting it to be used by women among in our community, so some of the community members were using this PP or PA services. All our

staff is introducing this service to the community, and we are getting good responses from the community so things are changing from time to time. In the beginning, women saw us as evil, when we were counseled about contraceptives immediately after having birth… but now it's changing… now they are getting familiar with immediate post-partem family planning services.

I: - In your opinion what should be done to improve access to the PP and PA family planning services for all women in your community?

R: *- to improve access we need your support (engender health), it is better if we have a review meeting to evaluate and monitor our performance and to take appropriate action as early as possible. That is good for us also… if we get a checklist and follow each activity and if we get regular support we can improve the service. Also, if we have sensitization and on-job training to motivate workers … I expect a good outcome if we do things like this… this is my opinion.*

<u>Practices, Roles, and Participation</u>

Now we are going to discuss about your role and responsibilities.

I: - Please describe to me the typical Woreda health office officials/experts roles in your Woreda/community?

a. How is Woreda health office officials occupied over the course of the day? Are there seasonal differences (campaign/political assignment) in how you use time?

R: *- I have the responsibility for overlooking all activities in the health center. I have to check activity recording books and follow the health workers. And as you said … during the different campaigns and meeting the role may be affected.*

And as you know in our country's context workers are not doing their job … they look for and wait for leaders. so, I care about my responsibility daily as much as possible. I evaluate health workers based on assigned responsibility.

I: - As Woreda health office official, what kind of work do you do? Do these works different when facility status changes (Rural, urban and geographical locations)?

I: - Do Woreda health office officials like you have restrictions/barriers on the accessibility? What restrictions/barriers? How do the restrictions/barriers influence the access of the PP and PA family planning services and supportive social networks?

b. In your Woreda do the restriction/barriers change by social characteristic, such as (dis)ability, economic status, health status, educational level, religion, culture, geographical location, and marital status?

***R:** - I haven't seen challenges or restrictions from the community side, but at the health center, we have a shortage of trained professionals in post-partum and post abortion family planning counseling and quality service provision for women. Other ways we don't have any challenges raising from community side or other.*

I: - Do you participate in any meetings, workshops, trainings, pregnant women conference, family, community, government, or other social networks organizations? Please tell me more.

c. Please tell me how your participation in these organizations affect the PP and PA family planning services provision

***R:** - yeah, I have participated in different community meetings or training at a higher level. And I got a good understanding of the community view towards immediate post-partum or post-abortion family planning during community meetings and also, I improved my knowledge and skill in different training that I got at different times regarding family planning or other reproductive health related training.*

This participation helped me in many ways… as I told you it helped me to understand community perception and to engage accordingly in health education programs and planning.

I: - In your Woreda do women assume leadership roles? What types of roles? Do these roles change by social characteristics, such as (dis)ability, economic status, health status, educational level, religion, culture, geographical location, and marital status?

d. Please tell us about your own experience with assuming any leadership roles?

e. Please tell me how your leadership roles affect the PP and PA family planning services provision

R: *women becoming leadership positions is important to facilitate this service implementation. More than men they understand the issue and woman's problems very well. As woreda, I know a woman leading health center in Buge, here also MCH focal person is female.*

Even what we did recently is gathering 1 to 30 leaders and informing them about this service and disseminating information to those under their supervision. And also, to work with health extension workers to make them use this service immediately after delivery. So, working with female leaders is good for assure service utilization by women.

This is not a new activity for us as health professionals but what is new is giving more emphasis than before. So, I will contribute well in the future … I did not do anything special regarding post-partum and post-abortion family planning.

I: - In your Woreda/community who decides at what age people marry? What are the reasons for getting married at younger or older ages in this Woreda?

f. Please tell me how their age at marriage affects their PP and PA family planning services use

R: - *eee… here there are individuals who marry at an early age even before finishing their high school study… when they reach 17 or maximum of at 20 years old. Those who marry at early age perceive that using the contraceptive methods before giving first birth is bad for their reproductive health… so they prefer to have or give birth before starting to use the contraceptive method. We don't know where they got this false information …*

I: - What are your suggestions to improve women's roles and participation to enhance the use of PP and PA family planning services?

R: - *to improve their role to enhance PP and PA service utilization we have to work on awareness creation. Our community is poor and has no access to media so we have to support them by providing information.*

<u>Knowledge, Beliefs, and Perceptions</u>

Now we are going to discuss about the issues faced by women in accessing PP and PA family Planning services in your opinion.

I: - In your Woreda what is appropriate behavior for a woman after delivery and abortion regarding the use of PP and PA family planning? How do they differ from adolescent girls and young women?

a. How do these believe influence health behavior including the PP and PA family planning use?

R: *- certainly, the community did not take it as appropriate … using immediate post-partum family planning. Many women consider that it is bad …*

I: - What are the social beliefs and perceptions that share women's expectations and aspiration for health care including PP and PA family planning?

R: *- yeah, they believe that she should stop bleeding and other vaginal secretion before having sexual relations with her husband, so they believe using the contraceptive method at that time is not mandatory.*

Most of the time presently delivered women agree easily to take contraceptive because she knows how pain full it was during delivery but other family members who are there with her like their mother made them not use immediate postpartum family planning.

Most of the time women come here to premature removal of a method they are using… most of the time the reason is … they complain of some side effects like dizziness and lightened head, loss of weight … that may or may not be due to the method she is using but generally they associate with the contraceptive method. They did not differentiate said effect among the drug they consider all contraceptive methods to cause a similar said effect on all users, which is not correct.

I: - In your opinion who should make decisions regarding use of PP and PA family planning services? What types of decisions do women make regarding use of healthcare including PP and PA family planning services? What types of decisions regarding use of healthcare including PP and PA family planning services are made jointly?

R: - *yeah, I know women who use or take secretly hide from their spouses… because some husbands do not allow them to use a contraceptive method. At that time if her husband knows it may cause distrust between them so it is very important if the contraceptive method is taken by mutual agreement by spouses. For their home peace it is better as I think…, yeah male partner wants to have many children … I don't know the exact reason but they did not know the consequence of having many children … they only consider having many children as wealth..*

Legal Rights and Status

Now we are going to discuss about the marriage related process prevalent in this community.

I: - In your community are women who delivered recently legally allowed to receive PP and PA family planning service?

- Is these same for boys and girls, people from different age groups, education background, ethnic groups, socio economic classes, excluded groups? How are they different? Why/ Why not?

R: - *it is legally allowed for women to get this service after having a birth or abortion. But there are some conditions to getting safe abortion …. But in the case of the contraceptive method, there is no any restricting role.*

I: - How do recently delivered/aborted women access PP and PA family planning information and resource from the healthcare facilities?

- Is these same for boys and girls, people from different age groups, education background, ethnic groups, socio economic classes, excluded groups? How are they different? Why/ Why not?

- Do the people in your community support recently delivered/aborted women to access PP and PA family planning information and resources? How do they provide support? Who are these people? Why do they provide support?
- Are there certain groups in your community who do not support this? Who are these people? Why don't they support?

***R:** - eee… women in our community get information from health extension workers and health workers at the health facilities. Also, they get information from health development army leaders in the community. We have close contact with HDA leaders we inform them then they disseminate information to others under their supervision. Since the area is rural most of the residents get information in this way … no TV or other media access for the community.*

Supporting community members… ee yeah, as I told you previously we have different forms of team among the community like health development army, 1 to 5 team … so this group supports each other. Also, those people who were educated and know well about the importance of spacing birth may encourage others to use contraceptives.

On the other hand, there are individuals who associate it with religious beliefs and teach them not to use contraceptives which are seen as a sin. … yea if you take the shshe area there are Muslim religious followers… and they do not allow women to use family planning.

I: - Please tell me women's experience with accessing healthcare information and resources including PP family planning.

- Do you feel they have a right to get healthcare information and resources including PP family planning services? Why/why not?
- Do you think women's experience is different from other people in your community? How? Please tell me more
- Is there legal mechanism you can follow to ensure they get quality healthcare information and resources they need? Please tell me more

***R:** - yeah, the client has full right… we also post at different areas of the health center compartment to promote their right and responsibility and to get the right service at the health center.*

Yeah, we have a mechanism for assuring information reliability… every fortnight we have regular meeting with health development team leaders, 1 to 5 team leaders we review their activity… they have also reporting mechanism to nearby health post we also evaluate based on that report …

What I want to add is … you have to keep up your regular supportive supervision … if you do that way it will have better outcome … and the program will be effective. Also, I would like to thank you (program implementing body) … if you can it is important to us you support us with required equipment to implement and facilitate the intervention. I was mentioned may problems so we need your support … thank you.

With this we have come to an end of the discussion. Do you want to add anything before we end the discussion or if you have any questions you want to ask?

Thank you for your time and in case you want, you can get in touch with us through the details mentioned in the consent form.

****

Summary

This interview was conducted in Wogera health center and the interview was conducted at his office and the place was quite. To reduce the flow of staff members or other customers we closed the door until the end of interview. The informant was well aware, however sometimes he bends towards the training effect on PP and PA family planning. Generally it was interesting interview, and informative.

FGD_AF__BO_01_28_7_2022

Code	Age	Educational status
R1	17	10
R2	17	12
R3	18	12
R4	16	10
R5	19	11
R6	18	12
R7	18	11
R8	17	11

I: Health services (PP and PA family planning services) availability: Can you tell me the types of healthcare facilities that provide PP and PA family planning services in your area?

a. What types of FP services are available at the healthcare facilities?
b. Are these services equally available to boys, girls, young, old, married, unmarried, people from different geographic locations, religious or cultural background, language etc.? Please tell me more

R2:Regarding to family planning services in Abala, there is lack of family planning methods. There are people who are giving birth without planning. people are not getting the services they need because it is not available in the health center. As the result they go to private clinics to get the services, and the private clinics may give expired drugs /family planning methods for them. There situations where mothers, adolescents conceive without plan. Therefore these are the problems I have observed in Abala abaya health center.

R7: There are different services we get from the health center. We get health information from the health center, they teach adolescents about condom utilization, they teach us how to prevent abortion, teach us about menstruation cycle ,how to count days in the month to prevent un intended pregnancy. In addition to adolescents groups they teach those who are married ones to use ‘loop’,awareness creation is being given, awareness creation is being provided on how to use condom to prevent unplanned pregnancies. They also teach us about abstinence.

R1: Even though there is lack of drugs/family planning methods ,they give us counseling services. For example if a mother/adolescent girl get pregnant without her plan, they get advice from the health center. Mothers ask health providers to get abortion services but the health center don't allow abortion services. The health providers don't counsel mothers to get abortion services because once a mothers/girls terminate pregnancy it affects their health. They encourage mothers to give birth rather than to abort even though it is unplanned. They teach mothers/girls how to prevent unintended pregnancies and also teach them to share their friends not to have unintended pregnancy. However some adolescent girls don't come and don't get the services, they don't plan for their life, once they get pregnant ,they hide themselves and don't come to health center.this is one of the challenges related to adolescents. If they are known to be pregnant without their plan they will bring crises to the community. There is a adolescent girl I remember who died because of unintended pregnancy. She suspected pregnancy and when she knew that she conceived ,she took drugs and lost her life. she did this because of fear of stigma and it was painful for the community and we buried her body at last. Therefore ,other adolescents get such a bad lesson from this girl. Private clinics give drugs to withdraw or terminate pregnancy. I wish this clinics stop giving abortion services because using drugs to terminate pregnancy can affect the health of a mother/girl. As I have said earlier the health center is not giving drugs but the only thing they provide is counseling services. They counsel us to use condom, when to have sexual intercourse, they counsel us to count the days at which pregnancy occur and not to have sexual intercourse on those days to prevent unintended pregnancy. However the private clinics don't give counseling services because they are money oriented. They rush to give abortion services. Once they give drugs to the mother the pregnancy is terminated. They drug they take will kill the fetus. The girl I said before was 8months pregnant and she used traditional medicine and both the fetus and the girl died.

R3:There are different services being given here.Condom is being given as my friends tried to mention. However, it is better if they give family planning methods which are very effective in preventing pregnancy other than condom.

I:which methods ?can you mention the effective methods please?

R3:For example implants are effective. If you take condom it is less effective because there is probability to pass through. Therefore, there could be unintended pregnancy. There is lack of family planning methods specially there is lack of effective ones.

R4:Thank you for giving opportunity. Actually we are getting different essential services. A friend of mine told me that she has got pregnant and her husband left her away and she told me that she has got fincial constraint to get abortion service. She asked me for help and I supported her and gave her what she needs. I did this to save her life. Unless she may commit suicidal acts and lose her life. There are services given for both males and females. For example for males ,there is condom in the condom box. It is better if there is training given for both males and females. Most of adolescent girls and boys are being affected and in a problem due to lack of training services. It is better to gather adolescents (males and females) to use the services they need to and arrange awareness creation session.

Accessibility of the PP and PA family planning services

I: Can you tell me how far the PP and PA family planning services providing facilities are located? How long does it take for women to access the facilities?

-What type of transportation means do they use to reach the facilities? How easy or difficult is it to use the transportation services?

-Is there any cost associated with accessing the PP and PA family planning services? Please tell me more about this

-Is the PP and PA family planning service is accessible at all time at the healthcare facilities? Are there types of FP methods of users' choice in the healthcare facilities?

-Are these services equally accessible to boys, girls, young, old, married, unmarried, people from different geographic locations, religious or cultural background, language etc.? Please tell me more

-How easy could they access the PP and PA family planning services? Do you think that their access to the PP and PA family planning services are affected because of their background? How?

R2: There is gap from adolescents side because they don't come and get the counseling services. it is better to disclose themselves than hiding once they get pregnant accidentally. The main reason they don't disclose is due to fear. It is not good to be afraid of disclosing rather they

should disclose because once the fetus becomes big it is very difficult to terminate the pregnancy. Still there are such problems in the community. This could be due to lack of knowledge and adolescents are not willing to get counseling services they put themselves at risk. Therefore, there should be awareness creation and counseling services for adolescents.

R5: It has been said by the previous discussant, but let me add something. Regarding to abortion service counseling should be done home to home to address females at their home. Males could get information about condom utilization because they spend their time outside they get different information. However, females don't know whether they conceived or not, until their fetus becomes big after they know after 7 or eight months of pregnancy. so this groups face different problems. Therefore it is better to visit and go to their home and share this idea for them because they don't come to the health center.

R7: Sometimes family by itself don't allow girls to disclose and they want to hide from people what happened to their daughter because of the fear that they may lose their value. Therefore they allow their daughters to give birth at home because of fear of stigma. Therefore, it is better to educate family members in addition to adolescent girls.

I: Can you tell me more about how far the PP and PA family planning services providing facilities are located? How long does it take for women to access the facilities?

R5:abortion is against the community value. There is ‘‘Hayttaa haydaa’’ in our culture. If someone does something wrong, they hide themselves from any social system unless the community may outcast that person. It is forbidden by the religion. Therefore, awareness creation should be done by the care workers either to prevent themselves from unintended pregnancy. Abortion is legally not allowed. Most of the community members have faced different challenges due to hiding themselves. Awareness creation is needed. it could be given in school, in places where adolescents gather together.

I: In your woreda how is use of PP and PA family planning seen? Is it acceptable for women to use PP and PA family planning? Why/why not? Please tell me more (what about boys and girls)

R3: girls should be happy and use post abortion family planning service because this is(pregnancy) happened due to failure to use family planning methods. In order to prevent unintended pregnancy and abortion it is better to use family planning after abortion and adolescent girl should be happy. we are also happy if girls use the service. For example if my sister accidentally conceives without her plan and give birth I am happy if she start to use post partum family planning. Females don't use the service because some of them don't know where the service is given, type of service being given, don't use due to fear.

R2: there are people(mothers/girls) who get the family planning services from the health center after delivery when they go back to their home in order to prevent unintended pregnancy. Even though some of them don't get the service, the others are getting the services. Girls sometimes are willing to have sexual intercourse with their boyfriends however males give them false promise for their girl friends and tell them that they are going to marry and live together. Therefore, males should be aware about this.

I: would you elaborate please? how immediate post abortion family planning is seen?

R1: If pregnancy accidentally happens to adolescent girl, we would counsel her so as to her . The community members, elders don't know about such things and they don't understand and don't support her .They can identify those who aborted girl by just looking at her face and she is physically deteriorated. Once people identify the girl they propagate information for others. In order not to lose their social value her family will send her somewhere and she will stay there from five to one year until she becomes stable. If we were in their place we would counsel the girl and counsel her not to repeat such a thing. We would counsel her to use family planning methods after she aborted. We would also counsel her to take care, not to have sexual intercourse during critical period where she could conceive. It is expected from adolescent girls to come and get counseling services. However, adolescent girls don't come to health center ,they go to private clinics to hide themselves from people because if they come to health center people from her neighbor ,family, her mother, brother may be there. But if she go to private clinic during night time it will be confidential and nobody can see. so she can use the service during this time and get counseling services. At this time private clinics may give her expired drugs for the sake of making money.

I: how immediate post abortion family planning is seen? Would explain how community would see?

R6:It is a norm in our community if a girl aborts, people would not disclose. It is very important if such girl uses family planning services, her peer groups can learn from her. we are very happy if this service is given for aborted girl. The community awareness regarding this service is very low. As it was mentioned by previous speaker mothers usually hide their daughter who aborted because of the fear of losing social value, they don't disclose it. Nobody knows whether a girl aborted or not because it is secretly done. Health care providers don't address such groups. A girl may abort frequently more than three or four times. We used to observe girls who gave birth for unknown person who is not her husband. The reason for the problem is that health workers did not reach the community and the community is not getting necessary information. We support the services and we are so worried because our sisters are facing different challenges. In order to prevent this different strategies should be designed ,awareness creation should be done at school.There are health extension workers in each kebele who are supposed to teach the community but if such thing happens(abortion) ,they also hide information. This could prevent a girl from using family planning.

We used to see many adolescent girls who aborted and who also gave without formal marriage for unknown person.In order to mitigate the problem, concerned bodies should give attention and explore the problem.

R7: we don't our sisters to abort but it is obvious that it happens. Once she aborts we encourage her to use family planning from the health center. There are many types of family planning methods. It is good if girls use family planning immediately after abortion. Family should play role by encouraging their daughters to go to health center and use family planning methods rather than hiding them.

R8:If a girl aborts ,first of all we should support her psychologically. Sometimes people in our community stigmatize and outcast if a girl aborts and give birth to unknown person without havening formal marriage. Therefore, we should give her psychological support and give awareness about immediate post abortion family planning services. We support and encourage if the service is given for adolescents. our community usually stigmatize the girl and she will be torched due to the stigma, mistreats. Therefore, there should be awareness creation within the

community. There are hard to reach areas where girls don't get any information, they abort and don't get family planning services after abortion. Health care workers should do home to home visit in order to reach the community because there are many girls who have no idea about family planning methods.

I: In your opinion what should be done to improve access to the PP and PA family planning services for all women in your community?

R1: There are people who are getting services as well as there are also people who are not getting the services. Those who are using the service are very few.

I:who are these? who are using the services and who is failing to use the service?

R1: Those who are living nearby health facilities can use the service. There are housemaids/servants employed. These groups are not getting the services, because the employer may not allow her to go to health facility, so she couldn't access health facility and necessary information. Therefore, information should be disseminated through mass media like radio, Television, printed materials news paper etc and also information should be delivered at school level, so that it is possible to reach unreached groups. we cannot miss if we use all the strategies. In addition to this concerned bodies should also play their role in order to make the service accessible. It could be constructing health facilities, health posts near to the community so as they can easily get the services.

R2: there are many things to be done from government's side. For example, the government has to avail all necessary family planning services in the health facilities, there shouldn't be scarcity.If that is the case the number of people using the service could be increased. On top of this there are community members who are not adhering to advises given by health care workers. For example there are mothers who face trauma, or they may fell down during pregnancy. As a result they may abort. During this time the health care workers advise them to wait for six months even though they have intension to conceive. There are mothers who fail to adhere advises and get pregnant immediately. In this case, their uterus couldn't be able to carry the fetus and it becomes complicated. Therefore, it is expected from the community to adhere advises given by the health workers.

I: Thank you. what additional thing should be done to improve PAFP services?

R3: As you have introduced the research focuses on adolescents. We can get those adolescents in school, specially high schools. I used to observe condom promotion in other regions and help the adolcents to use the service. But I didn’t see condom box in high school here. As it was said earlier the government has to work on accessibility of the services and facility because we have heard a rumor that women are not getting the family planning on her choice because of shortage and they go back to their home without getting the family planning the need.

I: which family planning methods are not available?what did you heard about from mothers?

R3: As I heard, there is shortage of long acting family planning methods. When mothers want to use long acting family planning methods ,they don’t get some times and she may tell her friends about the scarcity so that they will not come to health center. Therefore, the government has to avail all the necessary family planning methods for the community members. In order to ensure accessibility interventions should be done at school level.both male and female Condom promotion should be done to improve the service.

R4:Actually many things have been raised.we could be able to get information about immediate post partum and post abortion family planning services because we came here and we can teach others but there are many adolescents who have no information about this service. Those who can easily access health facility, those live around town area can get information but there are living in remote and hard to reach areas.They may come to town are once a week or a month.If these people don’t get information, if there is no education given ,they will not have awareness because it is not possible to get information about family planning if they don’t come to health facility. The one who live in remote area are at risk of getting different problems because they don’t have access to get information. therefore trainings, all necessary information should be given for those who live in hard to reach area. Health information should be given in every village or “got” so that they will be protected from many health problems.

R5: So far we have been discussing PA family planning in relation to adolescents. However, there are married people are suffering. For example, there are mothers who give birth while they are breast feeding their previous child without having time gap ‘‘boli bola yelluwa’’. Therefore counseling should be given for such mothers. Health care workers should go home to home and create awareness.

R2: adolsents should get information from health post if they can’t access health centers.In order to prevent unintended pregnancies and ‘‘boli boli yelluwa’’ ,all the family planning methods should be available in the health post because the current living condition doesn’t allow women to come to health center, there are transportation and other costs. which can be obstacle for service utilization. People are suffering a lot due to economical crises.

R7:Unintended pregnancy can occur any time. Therefore health care workers should have schedule to go to community and counsel them and do assessment on unintended pregnancy.

I: what are barriers on the accessibility? What restrictions/barriers? How do the restrictions/barriers influence the access ofthe PP and PA family planning services and supportive social networks? In your Woreda do the restriction/barriers change by social characteristic, such as (dis)ability, economic status, health status, educational level, religion, culture, geographical location, and maritalstatus?

R6:one of the possible barrier could be related to religion. Some people believe that using family planning is against God’s commandment. Abortion is also not allowed by religion. If a girl aborts that is curse for the community. Those who have aborted will be out casted from the church and hard measure is taken upon them. This could be a barrier. The other barrier is lack of awareness. As it has been said by my friends earlier those who live in far areas are not getting the services. Health care workers are not willing to go to the community to teach them because of the distance. There are different services given for mothers, children, vaccinations are being given. However, nothing is done for adolescents in our community, health care workers don’t address adolescents. Nobody gathers adolescents therefore, they are neglected groups. There could be information dissemination done once a year but it is also ignored.

Therefore, Strong intervention should be done at each village in each kebele. awareness creation should be done for the community. Awareness creation should be given adolescents to keep themselves from ,unintended pregnancy and abortion and once they aborted there should be awareness creation should be done to use family planning service after abortion.

R2: Adolescent girl may give birth or abort and she may say “my husband is no longer going to have sexual intercourse with me because I live with my family’’ and she may not use family

planning due to this.This could be one of the barrier. In order to solve such problems it is possible to get lesion from “GESHIYARO PROJECT’’ working on hygiene and sanitation and administering mass drug administration for everybody and reach every corners of the village. Likewise, the health workers, health extension workers should also reach hard to reach areas and disseminate health information. In doing so we can solve the barriers.

I: Are there any other barriers? would you add more please?

R3: I heard someone who had sexual intercourse using condom but his friend became pregnant and I’m not sure whether she aborted or not. what I wanted to say here is that the effectiveness of condom is very less. Adolescents should use effective family planning in order to prevent unintended pregnancy. The use of condom can be a barrier. If someone who had sexual intercourse with condom and have conceived, people would not use condom as family planning method because they don’t trust it.

Now we are going to discuss about your role and responsibilities.

I: In your Woreda do women assume leadership roles? What types of roles? Do these roles change by social characteristics, such as (dis)ability, economic status, health status, educational level, religion, culture, geographical location, and maritalstatus?

R1: Females are good and they are effective in leadership areas. However, there are still challenges with this regards because males try to dominate and create conflict.There is one female person who is working and leading one of government sector in our woreda but males will always want and take the lead and they became challenge and she left away the sector although she has potential. Therefore, the government has to support females specially help females on top leadership. the community is happy with their leadership skill. females are mostly trusted. Females are better than males.

R3: Regarding to abortion, and other services since the problem mainly affects females, it is better if counseling is given by females rather than males. This can increase acceptance of the services. Therefore it is good if they are assigned in leadership positions. Most mothers/girls are not using the service due to fear. specially females are not happy to be counseled by males but if the counseling is given by females mothers and girls would come and use the services.

I: In your Woreda/community who decides at what age people marry? What are the reasons for getting married atyounger or older ages in this Woreda?

a. Please tell me how their age at marriage affects their PP and PA family planning services use

R: (R1-R8) laughing.

I: I want to know what makes you laugh.would you share your idea please?

R6: The nature by itself tells girls or boys at what age they should marry.It is legally known and found in constitution of Ethiopian Government. For example,at the age of 18 people can lead their life.early marriage is when they marry before 18 years of age. The family law does not allow them to marry before 18 years of age. Therefore, the first thing is the nature it self, the second is the government. Regarding to factor s that push for early marriage, in our context because our environment is hot girls and boys reach to maturity at their earlier age, there are food types they can easily get. Therefore, nutrition can also one factor ,if they are well nourished their physical body seems matured in their earlier age…laughing. This is not something to be undermined. Because interventions should be done in their context. we need to think about what intervention should be done in such places?.

R7: most of the time decision is made by males. Even though the one they are about to marry are under 18 years of age they decide by themselves and force them to marry. As a result females are forced to marry at their earlier stage.

R8: there are some fathers and mothers who decide. They encourage their boy and daughters to marry.

I: What are your suggestions to improve women's roles and participation to enhance the use of PP and PA family planning services?

R2: it is better to encourage and support females in order to improve women's roles and participation because they can teach about this issue more than males.

I: In your Woreda what is appropriate behavior for a woman/ girl after delivery and abortion regarding the use of PP and PA family planning? How do they differ from adolescent girls and young women?

a. How do these believe influence health behavior including the PP and PA family planning use?

R3: Ethiopia as a country has something called shame ‘‘ pokko’’ means taboo. if it is in abroad it is seen as normal.In Ethiopia most of the things are governed by ‘pokko’ or we call “hyttaa haydaa’’ in wolaita. If a girl accidentally gets pregnant and aborts ,it is seen as something shameful acts. Her family will lose their confidence. Those who aborted girl are ashamed and hide themselves from any social events. Therefore, the community has negative attitude towards it. The relation between aborted girl and her family is also affected. If the community gets information about post abortion family planning service they will accept without any challenge.

R1:Aborted girls is seen as something evil and they will become prostitutes because of the stigma, they can dropout from school and at last they become sexual workers. She says “*Once I lost my social value I don’t have any hope to marry”.*there are also girls who keep themselves after they aborted. Therefore we should support them psychologically.

R2:It is natural that human beings can always do something wrong. It is therefore expected from the family to correct such wrong acts. The family should teach their children so as to behave in right way. Fathers should counsel their daughters and there should be open discussion within the family rather than taking harsh measures up on their children in case their children do things wrong. As a result children can adhere to their parents advises. There are some people who always take harsh measures up on their daughters rather than counseling their children. therefore ,family can play great role.

R7: There are wrong thoughts in the community regarding females.When females are attending their school their family would say “timirtiya ba azinaw tamarawus’’which means “they attend school for the sake of their future husbands, we don’t get any benefit from her’’ …no matter how much they stay with us it is obvious that they will depart their family and get married.’’

I: can this affect family planning services? how

R7: yes ,this can highly affect family planning utilization. If a girl does not attend school ,how can she understand about family planning …she does not know about why family planning is important. Therefore, there are such a thoughts in the community.

R4:It has been already said but let me add something. There are many problems related to family (father and mothers).It is obvious that females are weak lack ,think locally and rashly decide up on their life.if a girl accidentally becomes pregnant her family immediately outcasts because of fear of losing their social value.As a result this girl will go away due to fear of her family.Therefore family(father and mother)should counsel her.

I: In your opinion who should make decisions regarding use of PP and PA family planning services? What types of decisions do women make regarding use of healthcare including PP and PA family planning services? What types of decisions regarding use of healthcare including PP and PA family planning services are made jointly?

R2:If they are married couples ,husband should encourage his wife to use family planning in order to prevent unintended pregnancy. An educated husband who married uneducated wife has to counsel her on this matters.

R8: people at every stage should take decision. If you take school there are school clubs working on family planning like HIV/AIDS club and therefore everyone should decide.

R5: in our community people totally oppose abortion. Let alone aborted girl, people don't like a girl who is standing with her boy friend. She is seen as something evil. They are supposed to treat her and counsel her but they outcast her from family and the community.

R7: it should be the girl and her family should also decide. The family should counsel her not to repeat such a thing. Health workers should also counsel and treat her psychologically. Above all She should decide for her own health

I: In your community are women who delivered recently legally allowed to receive PP and PA family planning service?

- Is these same for boys and girls, people from different age groups, education background, ethnic groups, socio economic classes, excluded groups? How are they different? Why/ Why not?

R3:In my opinion it is not allowed legally because If a girl aborted the chance of delivering will be decreased, and if she uses family planning this can still decrease the probability of giving birth.That is why I said it is legally not allowed.

I:what about others?

R2: we have been learning about family planning. Therefore, it is legally allowed and people should accept the directions from the government. There should be adequate time gap between the current and next child.

R6:In my opinion it is legally allowed. The government is totally against abortion. Those who abort are legally asked because it is crime.

R7:The government doesn't oppose this because it is economically, socially, and good for health of the girl.

I: How do recently delivered/aborted women access PP and PA family planning information and resource from the healthcare facilities?

R1: Married ones can get information from their friends. They get information from those mothers having two or three children, they get their experience. These mothers will counsel the primi mothers. Unmarried ones can also get information from their peers. Those who live in town areas can get information from health center, health workers, mass media, peer groups

R2: it is possible to get information from educated people and teach others.

R7: they can get information from school. unwanted pregnancy is something they can learn from school

I: Do you feel they have a right to get healthcare information and resources including PP family planning services? Why/why not?

R1: Yes they have right to get information. It is their right to access information from anywhere.

R3: they get information from mothers who previously used the services ,peer groups.

I: Is there legal mechanism you can follow to ensure they get quality healthcare information and resources they need? Please tell me more

R7: we don’t have such a legal mechanism to ensure whether they get quality health care information, resources they need. The only place could be health center.

R6: There are different people in the kebele who teach the community about the services ,the problem is distance related problem this can prevent them from going. However there is no legal mechanism to follow whether they get information or not.

I: Thank you so much.you have said a lot about availability of PPFP,PAFP ,accessibility, how the service is seen in the community? the role of women. How to improve the service.Do you have something to add.

R:(R1-R8) thank you very much

Summary

This is Focus group discussion conducted with boys in Abala faracho kebele. The participants were 8 in number. FGD was held within health center YFS room which is free from noise.

- ✓ There is unmet need of family planning services because there is demand from adolescent’s side but there is scarcity of family planning methods
- ✓ There is low Uptake of immediate post abortion services because adolescents hide themselves due to fear of stigma.
- ✓ Adolescents should get effective family planning services because use of condom is under question. It is less effective.
- ✓ Attention should be given for adolescents who live in hard to reach areas and it is better to reach them using different strategies like home visits.
- ✓ It is better to reach adolescents employed like housemaids because their employer may not allow them to use. They can be categorized as one of unreached groups.
- ✓ There is lack of adolescent targeted counseling services
- ✓ Families are identified as one of barrier not to use the services b/c they hide their daughter not to lose their social value or “Haytaa haydaa”.
- ✓ Adolescents should have Open discussion with their family rather than hiding themselves.

- ✓ Counseling should be given for families having adolescents
- ✓ Aborted girl is seen as evil by the community and the community don’t understand her, they point at her this can affect immediate post abortion family planning service.
- ✓ Health care workers ,school should play their role and should create opportunity to disseminate information post abortion family planning services.
- ✓ It is better if counseling service is given by female health workers than males

Profile of the participants

**Participant 1**	**Age**	**Area**	**Remark**
Participant 2	16	Badessa	Single&student
Participant 3	15	Badessa	Single&student
Participant 4	17	Badessa	Single&student
Participant 5	17	Badessa	Single&student
Participant 6	17	Badessa	Single&student
Participant 7	16	Badessa	Single&student
Participant 8	17	Badessa	Single&student
Participant 9	17	Badessa	Single&student
Participant 10	16	Badessa	Single&student
Participant 11	15	Badessa	Single&student

Discussion

Rapport Building

Thank you for proving consent to participate in the discussion. There are no right or wrong answers, please share your frank opinion, as it will help us in understanding the situation better.

Assets and Resources

Health services (Immediate PP family planning services) availability and Accessibility

1. Can you tell us the types of healthcare facilities that provide PP family planning services in your area, and what are the types of FP services are available at the healthcare facilities?

P7: These "PP and PA" are provided at the health center as I know. Another one is it may be given at private clinics. "the participants feels shy to talk and her friends laughs in the middle"

Some people use pills and others use calendar method to prevent pregnancy.

P6: The prevention methods "FP method "include emergency contraceptives or post pill, pills, male and female condoms, injection for three months, the one inserted under arm for three years.

P7: there are also traditional methods provided to prevent pregnancy. Such traditional methods are available at the town. Normally married couples come to the health facilities and others go to the traditional medicine.

2. Are these services equally available and accessible to everyone who is in need and at any time? Please tell me more

P6: There are cases women were forced to give birth despite her willingness being pushed by her husband. In such cases she can't use the service. If not married, women get pregnant and abort the pregnancy such as commercial sex workers.

P1: I saw many people are using FP and it is available in this facility, but there are some women who complain the occurrence of pregnancy despite using FP methods. Why that happening and this is my question. (This question and other questions were addressed at the end of the interview by the facilitators)

3. Can you tell us how long does it take for you to access the facility and what type of transportation means used to reach the facility?

P1: Women come from urban and rural areas. some come from far areas.

P5: Those who are far can use ambulance or motorcycle and the ones who are near walk to the facilities.

4. Are healthcare providers receive and treat users with respect and dignity? If no why and how?

P5: Sometimes it is not comfortable and some women feel bad in service provision. There are also some women who get a good service and are happy.

P6: most of the time rural women who come from rural think they will not be given enough attention from the health providers because they use health insurance and it is not a good service.

5. Who makes the decision for the use of household income? How does this affect to access the healthcare services including PP family planning?

P4: It is the father who makes the decision and that is obvious. Regarding family planning most of the time they make a joint decision.
P7: Regarding FP there could be agreement or not. There could be a disagreement and they could contradict each other like the mother may not want to use the service while the father wants, or the father may disagree when the mother wants to use the service. But it is better if they make a joint decision.
P8: As she said they could agree or not. In one house if the mother wants to delay pregnancy, she will use a long lasting FP for 12 years. As her age goes, she can remove it.

6. In your community how is the use of PP family planning seen? Is it acceptable for women to use PP family planning? Why/why not? Please tell us more about it

P8: The communities have their own view. If a woman gives birth every year, the community may start to make a joke on her saying is she a chicken? So, using FP is seen as something good.

P3: People have different views and it differs from one to another.

P2: There could be a positive attitude from the community and I think the majority supports using family planning because helps have gaps b/n births and to balance with the economy.

P9: There are people who support and not. Some people say it is against the will of God to use it. Especially the wives of religious leaders don't want to use it.

P10: People have their own view. Some husbands enforce their wives to give birth and they even beat them after coming being drunk.

P7: Some people become pregnant after using it "FP" under their arm. Those who become pregnant advise others not to use it saying it doesn't help "to prevent pregnancies".

7. In your opinion what should be done to improve access to the PP family planning services for all recently delivered mothers in your community?

P6: In every house if they get some posters and flyers to read about FP it would help. Especially husbands should be counseled about FP. They should know the advantages of having a balanced family number. There should be community awareness creation at the community level. Those who know about it like YFS members should teach others about using FP.

P10: i disagree with her "the previous speaker". There are different kinds of people. Some can read and some can't read. On other points I agree but flyers can't be used by all since there are people who can't read.

Practices, Roles, and Participation

Now we are going to discuss about your women's role and responsibilities.

1. Is there any restrictions for women's mobility? What restrictions? How do the restrictions influence the access to the PP family planning services and supportive social networks?
   - In your community do the restriction change by social characteristic, such as (dis)ability, economic status, health status, educational level, religion, culture, geographical location, and marital status?

P6: Some women became so sick after they use it "family planning." They become too thin or too fat. They complain of gaining weight and becoming sick. Such things can be prevent people from using it.

P5: I think those who don't use are mainly from the rural areas. They don't have awareness, so they are not willing to use it. The reason may be lack of education and this can be a restriction.

P7: As she "the previous speaker" said most people complain of being sick. Even some people say they immediately develop headache after inserting family planning in to their arm. They also say the feel weakness and they can't do hard work if they use it or lift something if they use it. "implanon".

P9: Some people say they don't need to use it because they say they don't see mensus for up to two years after birth. So, they think they don't have to use it.

2. In your community do women assume leadership roles? What types of roles? Do these roles change by social characteristics, such as (dis)ability, economic status, health status, educational level, religion, culture, geographical location, and marital status?
   - Please tell us about your own experience with assuming any leadership roles?
   - Please tell us how your leadership roles affect your PP family planning services use

P7: Yes, especially after Abiy come to power women assume leadership role. In our area women assumed leadership roles. For example, the leader of this health center, and others in the education sector. There is no relation b/n women leadership role and using family planning.

P6: there are people who come to the leadership in the church and others at national ministerial levels. I don't think there is no effect of bringing women on using PP family planning.

3. In your community who decides and at what age people marry? What are the reasons for getting married at younger or older ages?
   - Please tell us about your own marriage experience? How old were you when you married? What are the reasons you married at that age?
   - Please tell us how your age at marriage affects your PP family planning services use

P2: Most of the time from 20-24 years. Nowadays even people as young as 12 are getting married. Things are surprising nowadays. There are also other who marry at 13, 14, 15 years. "most of the participants mentioned those years simultaneously"

P4: They marry at this age because they want to receive phone, jewelries and other modern stuff from men. They marry because they want to get those things.

P7: Most girls marry after 15 years and the reason for marrying at these early years is to get materials, but they say it is due to love. I don't think it is love. Those who marry at early age say no one will marry those who are already above 20 years.

P5: There is also peer influence that makes people to marry at early age. The influence of peer and when men steal the heart of girls, they can easily fall in to marriage.

P6: There is also parental influence. Parents take care of the boys than girls. Girls have to do a lot of tasks and while boys are enjoying outside. Despite doing a lot of works, still girls will face critisim and insults. In the midst when there is someone who offers something for this girl, she will easily get married. She thinks the care from the boyfriend will continue like this, but it ends soon. Some girls get divorced after that.

P10: Some girls think to have as much children as possible before 45 years and they want to marry early. Muslim girls are forced to marry after 12 years old when her parents push her to get married as early as possible. Some marry because of feelings “sexual desire” and they want to marry as soon as possible. Most girls start to use FP after giving birth to the first child.

P9: Some girls don’t want to use FP because it can cause harm. If women use family planning before giving a birth to the first child, it may cause repeated abortions later. It may even prevent from becoming pregnant.

P7:   most of the time they say the one inserted under arm for three years can cause such “frequent abortions later” problems.

P6: If women use FP before giving the first birth, it causes a lot of problems. I know some people who became unable to give birth after using FP. Even if they are able to give birth, the baby would be usually retarded, sick and weak. So, I don’t think it is a good idea to use FP before giving a birth to the first child. The husbands’ families usually want wives to give birth as soon as possible.

4. What are your suggestions to improve women’s roles and participation to enhance the use of PP family planning services?

P7: To increase girl’s participation, her involvement at the household and locality should be encouraged. She should be supported to come front and be confident. The community should support this. In most places boys are elected for different roles and this decreases her confidence.

P3: Usually girls were ashamed to talk in the public. More than men women discourage and label each other if she stands and speaks in the public.

P5: At the school some teachers encourage and push us to marry as soon as possible. They even say why are you waiting until now? Your maximum achievement is going to Arbaminch college and attends teaching college. So, they say don’t waste your time, marry as soon as possible. Teachers say this ot us last year and this year. There are some girls who believed such speech and followed his advice.

P7: when there was election of representatives in our class, teachers discourage girls not to be class representatives. They say girls will be absent or they will have a date. So, girls shouldn’t be class representatives say some teachers. So, measures should start from the schools.

P9: some female teachcers don’t like girls and they discourage girls from assuming any role.

P6: Most of the people still don’t believe on the equality of women and men from even birth. There is discrimination from the first day of birth. Civics teachers don’t believe on men and women equality even though teach in classes. Families do still not believe on equality of boys and girls.

5. Knowledge, Beliefs, and Perceptions

Now we are going to discuss about the issues faced by adolescents in accessing SRH services in your opinion.

1. In your community what is appropriate behavior for a woman? How do they differ from adolescent girls and young women?
    a. What is the appropriate behavior for a recently delivered mother? How do these believe influence health behavior including the PP family planning use?

"Almost all the participants said it is similar to q6 on the above and said they have already answered this question."

P6: The community believes as we discussed earlier say it is not good before giving birth to the first child and they can use after having a child.
P4: It is not good to immediately use FP after birth since it can cause sickness. They can be sick of weakness and other discomforts.

2. What are the social beliefs and perceptions that share women's expectations and aspiration for health care including PP family planning?
3. In your opinion who should make decisions regarding use of PP family planning services? What types of decisions do women make regarding use of healthcare including PP family planning services? What types of decisions regarding use of healthcare including PP family planning services are made jointly?

P5: They should make a joint decision. In order to live a good life, they have to discuss and make a joint decision. In our community what we people have and number of children is not balanced. It is good to support the family, feed the children and to have a loving family if they make a joint decision.

P7: They should make a joint decision. Making a joint decision is good for both husband and wife. Some husbands think women became less sensitive "for sex" if they use family planning. They don't want women to use FP.

P9: I agree with what they both said. Sometimes husbands become bad after she give birth. So, some women refuse to give birth in order to keep a good shape of their body.

Legal Rights and Status

Now we are going to discuss about the marriage related process prevalent in this community.

1. In your community are women who delivered/**aborted** recently legally allowed to receive PP family planning service?
    - Is these same for boys and girls, people from different age groups, education background, ethnic groups, socio economic classes, excluded groups? How are they different? Why/ Why not?

P8: If someone faces abortion, the law supports her to use either depo or other types of family planning. The law allows them to use it, but they have to be willing to use it. Unless they will be pregnant again.

P10: Families can prevent them from using family planning.

P3: Using FP is not good for young women because it can prevent the future chance of becoming pregnant. It is not advisable to use FP for young girls.

2. How do recently delivered/**aborted** women access PP family planning information and resource from the healthcare facilities?
   - Is these same for boys and girls, people from different age groups, education background, ethnic groups, socio economic classes, excluded groups? How are they different? Why/ Why not?
   - Do the people in your community support recently delivered women to access PP family planning services and resources? How do they provide support? Who are these people? Why do they provide support? if not why? who are these people who don't support?
   - Are there certain groups in your community who do not support this? Who are these people? Why don't they support?

P4: Information and resources can be obtained from the health facilities and those who were trained. And people like you can also give information.

P5: Those who are not yet married can get the information from those who are married and already using it. From health center, from TV, radio and other information sources.

P1: Most of the time women come to the health center and get information from there.

3. Please tell us your experience with accessing healthcare information and resources including PP family planning.
   - Do you feel you have a right to get healthcare information and resources including PP family planning services? Why/why not?
   - Do you think your experience is different from other people in your community? How? Please tell me more
   - Is there legal mechanism you can follow to ensure you get quality healthcare information and resources your need? Please tell me more

P7: We get information from our classmates and from YFS center. Those who get information share with each other. We also help others by sharing the educational materials from here.

P5: There are other girls who engage in unprotected sexual intercourse and we teach them on how to protect themselves.

P6: The YFS coordinator has taught us a lot of things about peer pressure and unprotected sexual intercourse. We also learnt about early marriage, STI, HIV, and other related things. The trainer is educated one the materials are from the ones from educated bodies. So, we know the information is useful for us. we can identify what is beneficial for us. I benefited a lot.

P4: I learned about sexual intercourse and HIV from the "YFS" coordinator here.

P3: If someone advices not to use FP, I think it is a bad person who says so. I learned about HIV/AIDS, STI, unwanted pregnancy. We learned a lot of things.

P8: There is a doctor in Sake who is helping a woman to use family planning very much. I don't know anyone in the community.

P2: We learned about the problems related early sexual debut and other health problems related with unprotected sexual intercourse.

With this we have come to an end of the discussion. Anyone who want to add anything before we end the discussion or have any questions to ask?

Thank you all of you for your time and in case anyone want, he/she can get in touch with us through the details mentioned in the consent form.

Summary:

This FGD was conducted at YFS center of Baddesa health center. At the beginning of the interview 7 gilrs came and after 10 minutes another 4 arrived to attend the session. We decided to include them in to the session hoping to get the rich information. Generally, it is good discussion, but we observed they become shy to express on sometime and we have to make a lot of effort to help them express they thoughts. We tried to get a mix of ideas and few participants were more active than the rest. The level of understanding to some of the questions like the effect of something on PP/PA family planning were difficult for them to understand and express answers in detail. The answers seem honest and they have expressed themselves more and more as the discussion matures, but the answers remained short and precise than exploratory.

FGDs

**Participants**	**Age**	**Area**	**Remark**
P1	**17**	Dimtu	Single&student
P2	18	Dimtu	Single&student
P3	18	Dimtu	Single&student
P4	15	Dimtu	Single&student
P5	20	Dimtu	Single&student
P6	19	Dimtu	Single&student
P7	17	Dimtu	Single&student
P8	15	Dimtu	Single&student

Assets and Resources

Health services (Immediate PP family planning services) availability and Accessibility

I:- Can you tell us the types of healthcare facilities that provide PP family planning services in your area, and what are the types of FP services are available at the healthcare facilities?

P3: - *we can get a family planning service in health post, there are women who were not using contraceptive even though she is living in a union (husband) so the health extension workers educate the community members to use the contraception method to regulate their fertility. If a woman does not want to be pregnant health workers give a drug which put in the arm (Implanon), there is also a drug given in injection form but I do not expect women are using those methods properly except for those who have formal education. As I see women were not using contraceptive methods adequately even the health worker teaches them very well … the reason is they (women) perceive that children as the gift of God and so God cares for them, they said. There is also a rumor that the drug will kill women's fertility so the government gives this drug to control the population number. So, the users here are few in number as I see.*

P2: - *yeah there are health workers here at the health center who provide this service for women, also there are health extension workers at the health post on each kebele who are providing this service for women. I have seen different times coming with users to the health centers or health posts providing contraceptive services for users. They serve their client respectfully and based on their client's needs. I know the drugs like Depo, drugs which are taken per mouth (pills), also which put in arm…*

I:- Are these services equally available and accessible to everyone who is in need and at any time? Please tell me more

P3:- yeah, there are some women who are complaining mistreat by health workers at health center. However there are health workers who serve the community properly by there profession.

I:- Can you tell us how long does it take for you to access the facility and what type of transportation means used to reach the facility?

P6: - *Most of the time people going to health centre by motor cycle or if they can not afford it they go by walk. For pregnant mother health centre provide ambulance service to bring pregnant mother to health centre for delivery. Most of the time women expend for motor cycle about 10 to 50 birrs based on the distance from the health centre.*

P3: - *there are many kebele … for instance if we take charicho, to come here to health centre by motor cycle it costs about 60 birrs… there people only use motor cycle they did not have access the transportation. The road is difficult for car transportations. Also, Monopoly kebele is difficult to car and people there use motor cycle or walk. It (monopoly kebele) costs 40 -50 birrs. There is also far kebele like Abaya… even for them Sodo is nearer then Dimitu health center. Again, in chabicho are is even difficult for motor cycle and people there come here to health centre by their feet. They can not get motor cycle access … I think they have health post there. I have family in chebicha kebele but there is no transportation to Chabicha if you want to bring car there you pay a lot… most of the time they come here to Dimtu by walk.*

I:- Are healthcare providers receive and treat users with respect and dignity? If no why and how?

P5: - *Yeah, the service here is good but sometimes they (health workers) did not come on time … sometimes women do not get the drug they want to use… this is the weak side of the health center.*

P2: - *Regarding maternal service, their service is very nice and they (health workers) care very well. But another service is not good as maternal service. Most of the time they did not have drugs after diagnosis. Their (Health center) maternal service is good when women come for family planning services…*

P1: *- As they said delivery and family planning service is relatively good when compared with the other medical service. The male care provider is more compassionate for women during delivery than the female here at the health center. Most of the time people use privet health facilities for other medical services.*

P3: - *yeah there is a private clinic providing family planning methods for women but they only give depo and drugs which are taken by mouth…*

I:- Who makes the decision for the use of household income? How does this affect to access the healthcare services including PP family planning?

P7: - around the town most of the people were involved in small business and government work so they get money from there. Other rural area residents and some people from the town are farmers and get money from farming activity. Most of the youth males have a motorcycle and they get money from it by serving people. And as the family they may be discussing together to spend money … there are also individuals (male) who is may control and did not allow for women to use money equally. There are different people … and families. Most of the time father or husband is engaged in income-generating activities and women stay at home and focus on homework or activity. Also, in many family father or husband decide on how to use family income.

P3: - in the town area most of the time the income … young mane get money by involving themselves in a different activity, daily labour, and from bus station … they are washing a car and get money…. For rural areas source of income is farming … from their farm they get money selling their product of the farm. Some may involve in small businesses. This (income) decision has an influence on mothers since many women live in a rural areas and need money for transportation and only the husband has income and control of money…

I:- In your community how is the use of PP family planning seen? Is it acceptable for women to use PP family planning? Why/why not? Please tell us more about it

P3: *- Those who live around the town area may use family planning in better way then those who live in rural area.*

P1: - *people see it as dangerous if it (modern contraceptive) used by young women before having birth for first time. They think that if it used by young women it leads women to become infertile and make things difficult when they want to become pregnant again. Most of the time women who want to continue their study use the contraceptive other ways they did not use contraceptive because they perceive it as unimportant. Many women also complain high bleeding during ministration when they use depo. For some user it is covenant … other may loss weight when they use depo and other may get weight. Because of this many woman are hesitating to use modern contraceptive. Most of the time women prefer to use Depo if they have to use modern contraceptive. It (Depo) is easy to use and they can discontinue if they want … if you take implanone they have to go health centre for removal and they have fear of talking for premature discontinuation after once they took it (implanone) …*

P2: - *Here in Dimuntu area… most of the women did not take or use contraceptive and also family member of the youth may not allow them to use contraceptives. They perceive contraceptive may cause infertility if used before giving birth. Many women prefer to use Depo… they fear the side effect of implanon… many women may loss weight or become over weight. And many women perceive gaining weight as good …*

P6:- people in our community advise young married women not to use modern contraceptive method in their young age. They perceive it may cause weight loss and cause infertility. But many women are volunteer to use contraceptive after having birth for the fist time. Most of the time a women who used contraceptive which put on arm complain they can not carry things and dizziness and Depo user also compliant weight gain which is abnormal. Also many people perceive that using contraceptive drug or method needs special meal so many women hesitant if they don't access milk products and milk. Here in Dimitu area people give birth or get pregnant if they are in safe condition.

I:- In your opinion what should be done to improve access to the PP family planning services for all recently delivered mothers in your community?

P5: - if the health worker serves the community appropriately the women may seek service. And also, health workers should have to work on awareness creation. As you know women are busy and they have many responsibilities at home they come to the health centre most of the time leaving many duties so if the service provider gives them quick service without delaying the women may be motivated to gain service.

P3: - It is better if the education and counselling way by health workers are improved and the drug is available always as the women need … because most of the time women get difficulty gaining Depo. The health workers should have to educate on the importance of spacing birth and consequence of having frequent birth on women's health and its effect on other family members. Male health workers are good in-service provision than female health workers. We have previously had youth-friendly service at the health canter and they educate us about reproductive health issues similarly it is better to establish a system to reach mothers who cannot come here among the community.

P1:- previously there is the pregnant mother who come from far kebele, where there is a waiting room so they come during the last month of their pregnancy and stay here till they give birth but know that program was discontinued and the mother come here only during the onset of labor. So the government should have to initiate this program again. Know there are mothers who deliver at home … in far/rural kebele mothers are delivering at home. If they (the health center) fulfill all the required materials in the waiting room and bring mothers and educate them about contraceptives appropriately family planning service may improve.

P4:- male partner support is important to improve contraceptive access by married women. Since the drug has a side effect on the user so the husband has to support the woman. Also, health workers are respected by people in the community so if they educate mothers

appropriately they (mothers) accept and use a contraceptive method. Also, the husband has to get training on how to support his family and wife.

Practices, Roles, and Participation

Now we are going to discuss about your women's role and responsibilities.

I:- Is there any restrictions for women's mobility? What restrictions? How do the restrictions influence the access to the PP family planning services and supportive social networks?

- In your community do the restriction change by social characteristic, such as (dis)ability, economic status, health status, educational level, religion, culture, geographical location, and marital status?

It was addressed under different question above. As the participant is not comfortable we proceed to next

I:- In your community do women assume leadership roles? What types of roles? Do these roles change by social characteristics, such as (dis)ability, economic status, health status, educational level, religion, culture, geographical location, and marital status?

- Please tell us about your own experience with assuming any leadership roles?
- Please tell us how your leadership roles affect your PP family planning services use

P3:- yeah, many women are assuming different roles in our community … there are female peacekeepers and different team leaders in the community.

P2:- there are female leaders but they are few in number as compared to males… for instance, my mother is leading a 1 to 30 network …that I know and there are many similar female leaders… who have the responsibility of disseminating health information among the community.

P5:- to give leading position people look at the individual personal ability to communicant with others, educational status, and motivation, and who can motivate others may have a high chance of getting the leading position. Most of the time females are shy and cannot express their feeling appropriately so that is why they are few in number.

I:- In your community who decides and at what age people marry? What are the reasons for getting married at younger or older ages?

- Please tell us about your own marriage experience? How old were you when you married? What are the reasons you married at that age?
- Please tell us how your age at marriage affects your PP family planning services use

P6;- starting from 15-year women are most likely to get married here in the Dimtu area… but most of them are around 18 and 19years. Most of the time marry at an early age the main reason is family living conditions and absence of comfort in their home and high economical problem.

P3:- most of the time women marry at an early age when they have problems with their family, many women have more burdens when they get mature and due to the stress, they develop as they get mature women decided to marry. Some families may not fulfill the required thing for women even if they want to continue their studies. When they get an exercise book they may have not gotten a pen and … they do have not to get the cloth to wear… they did not get food to eat. As women we need more food even … we need a pad for menstruation… and family cant fulfill this all they got married at early age cutting their studies. Some women may engage in marrying emotionally without knowing about it by looking at others.

P2:- Dimutu area has a very hot climate and very young people physically look very huge. so women get married when they look physically looked old at an early age. If you look at a

12 or 13-year-old young girl she looks 18 or more physically. And as many people think using contraceptives at an early age is bad for women … most of the time they do not use contraceptives at an earlier age or having birth if they are in legal union. They prefer to give first birth before using any FP method. No one has seen whether it is true or false but just it is a rumor.

P1:- what I want to add to my friend's idea is there are women who use or take contraceptives even before having their first birth… as they (previous speaker) many women marry at an early age while they are in high school so there are women who want to continue their study so in such condition women may use contraceptive method and continue their study, I know the such person.

I: - What are your suggestions to improve women's roles and participation to enhance the use of PP family planning services?

P6: - To increase women's participation and involvement they should be encouraged and get exceptional support from community health workers. women should be supported to get information about the services at the health facilities.

2. Knowledge, Beliefs, and Perceptions

Now we are going to discuss about the issues faced by adolescents in accessing SRH services in your opinion.

I:- In your community what is appropriate behavior for a woman? How do they differ from adolescent girls and young women?

a. What is the appropriate behavior for a recently delivered mother? How do these believe influence health behavior including the PP family planning use?

P1: - in our culture women give their first birth at their family home because of this they may not volunteer or see it as inappropriate to use the FP method. But in their second and

other birth, I think it is good for women if she took while she leaving the health center after delivery. I think other people may support this idea.

P6: - I think it is appropriate for women to take the FP method immediately after birth, but not all women who give birth volunteer to use immediate post-delivery FP … it is fifty-fifty. Some may think that their fertility returns stays long or so prefer to use the natural method after delivery.

I: - What are the social beliefs and perceptions that share women's expectations and aspiration for health care including PP family planning?

I: - In your opinion who should make decisions regarding use of PP family planning services? What types of decisions do women make regarding use of healthcare including PP family planning services? What types of decisions regarding use of healthcare including PP family planning services are made jointly?

P5: - as I know most of the time women decide by themselves to use the FP method. There are many women who are using FP without their husbands knowing it. … some spouses may want to have many children and not support using contraception.

P7: - I think it is good if they decided forgather, but as raised or said before some spouses are not allowed to use the FP method at that time in my opinion it is better to use contraceptives by deciding by herself.

P3: - most of the time youth families may discuss together and use the FP method also those who are educated may also decide together. But the problem is in the rural areas I think … deciding together has many benefits for family good relation … so in my opinion, it is good to decide together.

Legal Rights and Status

Now we are going to discuss about the marriage related process prevalent in this community.

I: - In your community are women who delivered/**aborted** recently legally allowed to receive PP family planning service?

- Is these same for boys and girls, people from different age groups, education background, ethnic groups, socio economic classes, excluded groups? How are they different? Why/ Why not?

I:- How do recently delivered/**aborted** women access PP family planning information and resource from the healthcare facilities?

- Is these same for boys and girls, people from different age groups, education background, ethnic groups, socio economic classes, excluded groups? How are they different? Why/ Why not?
- Do the people in your community support recently delivered women to access PP family planning services and resources? How do they provide support? Who are these people? Why do they provide support? if not why? who are these people who don't support?
- Are there certain groups in your community who do not support this? Who are these people? Why don't they support?

P2:- women got information from the health center and there are different networks among the community that is disseminating health-related information among the society who are supported by the government … there are 1 to 5 neatwork or 1 to 30 network. Every woman involved in this team can access information easily. The network leaders work together with community health extension workers. in this way they access information... Also when women come for ANC or delivery service health workers counsel about the FP method. As I mentioned above the team leaders got training at different times. As I told you my

mom is 1 to 30 network leader and she has list of mother under her supervision and she know who is using FP or who is pregnant among the team so she teach them based on their condition. They meet and discuss different health issues. So most of the time community members got information from health workers and different team leaders among the community. I do not know any team who are against FP … but we hear some individuals who are preventing their children or wife from using the FP method.

P5: - women get information about FP from health extension workers… if there is anything that is given at the health post, health extension workers send information to 1 to 30 network leaders, and the team leaders distribute information to women under their supervision…

P3: - there are community health education and sometimes training for mothers by health extension workers. At that time mothers got information.

I:- Please tell us your experience with accessing healthcare information and resources including PP family planning.

- Do you feel you have a right to get healthcare information and resources including PP family planning services? Why/why not?
- Do you think your experience is different from other people in your community? How? Please tell me more
- Is there legal mechanism you can follow to ensure you get quality healthcare information and resources your need? Please tell me more

P2: - *mother got information from health extension workers or community health workers … as we rose/said above people first got training or health information from health professionals so we expect that the information we or the community got from community health workers is reliable. Again, they can check information reliability by asking health workers at different health facilities in the society.*

With this we have come to an end of the discussion. Anyone who want to add anything before we end the discussion or have any questions to ask?

Thank you all of you for your time and in case anyone want, he/she can get in touch with us through the details mentioned in the consent form.

Summary:

This FGD was conducted at Demitu Health center. There were 8 male participants. In general, it was a nice discussion, but we observed they become shy to express ideas since it was culturally sensitive for them to talk and we have to make a lot of effort to help them express their idea. We tried to get a mix of ideas and a few participants were more active and dominated the discussion but we tried our best to balance the idea of the participant.

Their knowledge about immediate PP/PA family planning is very poor and they are confused with normal FP time so we tried to make clarity between immediate PP/PA family planning and normal FP time difference. But for them, it was difficult to understand and express answers in detail.

FGDs_GA_B_300622

FGD: Guidelines

Purpose: *Discussion* ***Guide for FGD target participants***
Tool Notes: *The facilitator should read out the consent form and take informed consent for participation. Instructions to the interviewer are in italics.*

Consent Form for Adult Participant (IDI)

Introduction

Greetings, my name is *<name>,* I am a member of research team of Jijiga University and Wolayita Soddo Univeristy, Research and Community service. We are doing this research in collaboration with Engender Health, Addis Ababa University and RHB among PP and PA FP clients, providers, and other stakeholders.

Purpose

The purpose of the study is to understand the socio-cultural and health system barriers and enablers to use of PP and PA family planning services in two regions in Ethiopia.

Procedures:
If you agree to participate in the study, we will ask you to participate in an in-depth discussion. The discussion would take about an hour. We will record the discussion for future reference and analysis.

Risks:
There is no risk in the study. To keep your identity confidential, we would not share your name and other identification details. However, if you feel uncomfortable at any point of time, you may discontinue the discussion.

Benefits & Compensation:
You will not receive any payment or compensation to participate in this study. There is no direct benefit to you for participating in this study. However, the information that we receive will help us in improving the programs for PP/PA family planning services.

Confidentiality:
We will do everything we can to keep the information you share with us, confidential. We will not share the responses with anyone outside the research team. The results of the study will be used to make the program for PP/PA family planning services better. You will not be identified in any publications or presentations resulting from this study. The computer files will be de-identified, meaning no one, not even the research team, will be able to link responses with participants and stored for three years after the study has ended. The list that will have your name and code number will be stored securely.

Voluntary Participation:
Participation in this study is completely voluntary, that means you decide whether you want to participate or not. If you decide not to participate, there will be no consequences. If you decide not to take part or leave it in between, we would respect your decision and not ask you any questions regarding your decision.

Contact Information:

In case you have any questions about the study at any time, you may contact Dr. Rashid Abdi, at 0915737863 or email: rashidabdi114@gmail.com or Dr. Elyas Abdulahi, at 0941070300 or Email: elyasabdulahi@gmail.com or Mowlid Akil, at 0915752811 or email: mowlidakil@gmail.com

Consent (to be audio recorded):

Do you understand the purpose of this research? ☐ Yes ☐ No

Should we explain the process again? ☐ Yes ☐ No

Do you have any questions? ☐ Yes ☐ No

Do you give your consent to participate in the study? ☐ Yes ☐ No

Do you need the copy of consent form? ☐ Yes ☐ No

Do you give your consent for audio recording? ☐ Yes ☐ No

Code	Kebele	Age	Educational status	Job	Marital status
1	Xaba	18	11	Student	Not married
2	Damot Ofa	18	9	Student	Not married
3	Damot Ofa	19	9	Student	Not married
4	Shakiso Shone	20	9	Student	Not married
5	Xaba	18	10	Student	Not married
6	Gacheno	20	12	Student	Not married
7	Xaba	19	10	Student	Not married
8	Ade Koyisha	21	12	Student	Not married

Discussion

Rapport Building

Thank you for proving consent to participate in the discussion. There are no right or wrong answers, please share your frank opinion, as it will help us in understanding the situation better.

Assets and Resources

Health services (Immediate PP family planning services) availability and Accessibility

1. Can you tell us the types of healthcare facilities that provide PP family planning services in your area, and what are the types of FP services are available at the healthcare facilities?

P8: Thank you for giving this chance. I want to speak about family planning. I saw my mother was using family planning method. And also my sister has been also using it. First of all the benefits of family planning is many. If there is no gap between births is affects the health of children. In addition, frequent birth without spacing results in malnutrition and other growth problems. The facilities providing it in our area is health center. My mother has been using the FP method serving for 3 years. Due to discomfort now she is using the one for three months. Now she is using depo. In addition to the health center, FP services exist in some clinics in our area. There are different family planning service in the health centers such as depo for three months, for the long term there are other methods. There is also short term family planning method that is used by young girls within 72 hours. Such pills are also available in the private clinics as well.

P4: Thank you for giving me this chance. Regarding family planning, even my mother uses it. For example I am 20 years old and there is at least three years gap between the recent births. I know she uses depo. There are also natural family planning methods by using calendar. When someone needs family planning methods such as condoms, it is available all over here. So, anyone can use if for family planning. When there were short birth gaps between births, I told my mother to use depo and now she is using it. The service is available at the health centers in our area.

P6: One of the main challenges in our area is not using family planning methods. I saw some people are having frequent births one on top of the other. I even told to some community members to use family planning to space between births. I saw some of them are becoming pregnant while the other is still breastfeeding. There are family planning services in the health facilities and if someone wants to use them, they can easily get. There are different types of FP. There are methods for 3 months, for three years, and for five years.

P5: There are different people in the community. Some people use the service and have spaces between births. Others don't want to use the service and there is no gap between the births. The services are available in the health facilities, if not they can be accessed from the private clinics.

2. Are these services equally available and accessible to everyone who is in need and at any time? Please tell me more

P8: if there is no family planning methods in the health centers they can use from the private clinics. They are available who even needs the service. I saw different family planning methods for boys and girls available in the private clinics, in the schools, and at the health centers. For example at school there is condom available for young boys. There are such services young people are using. I have also used some times.

P4: when someone wants to use they can get information from the health care providers. When the health care providers go for the outreach, they teach community to come and use the service from the facilities.

P6: yes, the services are available for everyone who wants to use them. The service is availbale, but some people don't want to use the service mentioning religion as one factors that prohibits usingn the service.

P5: If someone wants to use the service, I believe it is found everywhere. So, someone can get the service from different places.

3. Can you tell us how long does it take for you to access the facility and what type of transportation means used to reach the facility?

P6: Regarding the accessibility, the services are available in short distance. It is not far to get the services.

P3: The services are accessible in two ways. For some people it is far and for others it is not far. For example for our case, the health post is located near our home. The services are not available sometimes. Our community has to go from Damot Ofa to Buge health center to get the service. The services are available at the health centers. If the service in not available at the health centers, we get from the private drug shops in the town. From our kebele to the health center, it is about one hour walk. If you use motorcycle it is about 30 birr from one trip. If someone has no money, it is difficult to walk because it has risks like getting darker, raining in the middle of the journey. So, it is not easy if someone cannot afford for the transportation. The service is free of charge. There are cases where the method they want is available and not available. In such cases, they use what is available. For example a recently married young lady from our locality wanted to take the methods for three months, but she couldn't get the method she wanted.

P8: sometimes health centers are far from the community. In such case, people us e the health post. When people visit the health post, if they get the method they want, they can use from that. If not, they have to walk up to 30 minutes to the health center. They road is not accessible by motorcycle as well. when they come to the health center, sometime, they don't get the methods they want. In such case, they use the already available methods of family planning. The services are free of charge either in the health centers or at the health posts. The services are accessible for all married, unmarried, different groups as well. It is according to their wish they use. Sometimes, they change the methods because of some discomfort. They have access to the services at the health center, health post, or at the private clinics. The services are free of charge supported by the government and anyone can use the eservice.

4. Are healthcare providers receive and treat users with respect and dignity? If no why and how?
5. Who makes the decision for the use of household income? How does this affect to access the healthcare services including PP family planning?

P2: the living condition in our community is not good generally. There is shortage of food and problems relate with the drinking water. They get the water from the river. The agricultural production is also not good as well. The decision on using the household income is primarily made y the husbands. Sometimes they make a joint decision, but most of the time, they decision is made by the husbands. Regarding the use of family planning, the decision is made by the husbands.

P1: The living condition is getting bad. There was no rain this year. Most of the corn crops are destroyed due to this reason. Most of the crops are destroyed due to lack of rain. Due to such reasons, even some people are starting to limit the number of children and they are chosing to use family planning methods. Regarding the household income decision, it is made by the husband, and sometimes jointly.

P6: Since most of our community members are farers, the living condition is bad due to lack of rain. Regarding the decision making, most of the decision to use household income is made by the father. Rarely they pass a joint decision.

6. In your community how is the use of PP family planning seen? Is it acceptable for women to use PP family planning? Why/why not? Please tell us more about it

P2: Most of the community sees the use of family planning as something good. People view family planning use as something positive.

P1: The community sees family planning use something positive and good because it is too difficult ,for example raise six children, they have to cover the cost of clothes, books, and other materials required for raising children. That is why they see using family planning as a good thing.

7. In your opinion what should be done to improve access to the PP family planning services for all recently delivered mothers in your community?

P2: In order to improve access to the family planning services for all recently delivered women, those who are already using the service should tell to others who are not using yet. They should tell them the advantages of using the family planning methods.

P1: We students have to teach the community members about the use of family planning. We have to teach our community. Those who were using family planning should tell to the recently delivered women when they visit them as part of cultural ceremony.

P6: there are people who are educating the community about family planning. In addition to teaching, I suggest they could bring the methods to the households and give them in each house that needs the service.

Practices, Roles, and Participation

Now we are going to discuss about your women's role and responsibilities.

1. Is there any restrictions for women's mobility? What restrictions? How do the restrictions influence the access to the PP family planning services and supportive social networks?
    - In your community do the restriction change by social characteristic, such as (dis)ability, economic status, health status, educational level, religion, culture, geographical location, and marital status?

P7: There is lack of knowledge regarding the benefits of family planning. This can be one restriction for not using family planning. There are also other people who don't accept counseling. There are also some areas where people mention religion as barrier. There are no restrictions by educational level or marital status. Young people also use the service.

P3: Husbands can be one restriction. In some cases women want to use the service, but their husbands prevent them. Not being educated can be also another restriction. There are some women who are not listening to use the service. There is no religious, cultural or other barriers who want to use the service. Since it is a free service, economic factors can not hinder women to use women and girls to use the service.

2. In your community do women assume leadership roles? What types of roles? Do these roles change by social characteristics, such as (dis)ability, economic status, health status, educational level, religion, culture, geographical location, and marital status?
    - Please tell us about your own experience with assuming any leadership roles?
    - Please tell us how your leadership roles affect your PP family planning services use

P7: No, there are cases where women assumed leadership roles from our locality.

P3: There are women who serve as local militia to keep the security of the community. In some cases those militia women even enforce women to use family planning service. They work with health extension workers and give family planning to women by forcing them.

3. In your community who decides and at what age people marry? What are the reasons for getting married at younger or older ages?
   - Please tell us about your own marriage experience? How old were you when you married? What are the reasons you married at that age?
   - Please tell us how your age at marriage affects your PP family planning services use

P7: There are cases where the decision is made by the family, and also there are some cases where girls decide by themselves. There are also forced marriage conditions such as abduction. Most of the time girls marry from 15-20 years of age. Young recently married couples don't want to use the service, but they can use after getting education from older people who have used family planning methods.

P3: Most of the time they decide by themselves without the knowledge of the family. There are also few cases who decide jointly with the family. There are also forced marriage when the get abducted. When they get abducted, they can use family planning after they give birth.

4. What are your suggestions to improve women's roles and participation to enhance the use of PP family planning services?

P3: To enhance the use of FP, women should be educated. They should get education. Those who used FP should teach others to use family planning services.

P4: For example my mother is not educated. She doesn't understand well the advantages of using FP. They have to get education from the women conference where all the women in the kebele gather together. Indeed they are doing such things in our kebele.

P8: In order to enhance FP use women development army should be strengthened. For example in our Kebele there is a WDA leader and 1 to 5 leaders. They support HEW. After they get education from the HEW, they can reach others as well. So, educating women by using the available chain is crucial to enhance the use of FP. training

P6: In order to enhance the use of FP, I agree with my friends ideas. If they get education very well, they can use FP methods more. Sometimes people perceive those giving education as someone who have no mission. So, while teaching women, educators should demonstrate the benefits of using FP and the disadvantages of not using FP. They should also bring real case scenarios. Education should be across each locality.

5. Knowledge, Beliefs, and Perceptions

Now we are going to discuss about the issues faced by adolescents in accessing SRH services in your opinion.

1. In your community what is appropriate behavior for a woman? How do they differ from adolescent girls and young women?
   a. What is the appropriate behavior for a recently delivered mother? How do these believe influence health behavior including the PP family planning use?

P7: The typical roles of women in the community include working in the kitchen, cooking, cleaning the house, taking care of the children, and cleaning the environment. Young girls have no much burden as mothers. As the age increase, the burden also increases.

P3: Women's role differ based on the educational lever. Those who were educated work in the offices such as teaching and other roles in different sectors. Or mothers who didn't get education work primarily household chores including cleaning and taking care of the household. Rural mothers are involved in all activities. Young girls sometimes support mothers such as cleaning household, cooking and collecting grass.
P4: In some case women while being pregnant, carry big things and that can cause abortion. There are such cases in our community. We have to help such mothers in order to support their wellbeing.

2. What are the social beliefs and perceptions that share women's expectations and aspiration for health care including PP family planning?

P8: There are some people in the community who mock on those who advise them to use FP. They say why are you telling us this since we were told to be reproduced as we wish. In such case health care providers shouldn't give up. They should give education continuously. But, there are also women who come in search of the services. There are perceptions on both sides. There are people who support and there are also people not supportive.

P5: There are people who perceive it has bad health consequences and also others community members who encourage using FP. There are community members who say it is useless and there are also others who say God said be reproduced.

P3: most of the community members perceive it as something good. I expect up to 95% perceive it as something good and the rest 5% perceive it as something bad. Those who already used the method perceive it as something good. If they have discomfort with one method, they try another method. There are also people who perceive it something unnecessary.

3. In your opinion who should make decisions regarding use of PP family planning services? What types of decisions do women make regarding use of healthcare including PP family planning services? What types of decisions regarding use of healthcare including PP family planning services are made jointly?

P4: The decision should be made jointly with in family. They have to make the decision before health care provider enforce her.

P8: The decision should be made by the husband and wife. Most of the time women seek the advice of their husbands before they use the FP service. After that, HEW give education for them and provide the service.

P3: The decision should be made jointly. Husbands can expel their wives if they don't consult them before using FP methods.

P5: They should agree together before making the decision. Unless, there could be a problem in the household due to even side effects of FP.

Legal Rights and Status

Now we are going to discuss about the marriage related process prevalent in this community.

1. In your community are women who delivered/**aborted** recently legally allowed to receive PP family planning service?

- Is these same for boys and girls, people from different age groups, education background, ethnic groups, socio economic classes, excluded groups? How are they different? Why/ Why not?

P7: Regarding the use of PP family planning service, the government gave the right for young people and for mothers as well. mostly it is required for mothers and fathers, but the service is legally allowed for everyone who wants to use the service.

P3: Since the government is the one who is bringing the service, there is no law prohibiting the use of FP service.

2. How do recently delivered/**aborted** women access PP family planning information and resource from the healthcare facilities?
    - Is these same for boys and girls, people from different age groups, education background, ethnic groups, socio economic classes, excluded groups? How are they different? Why/ Why not?
    - Do the people in your community support recently delivered women to access PP family planning services and resources? How do they provide support? Who are these people? Why do they provide support? if not why? who are these people who don't support?
    - Are there certain groups in your community who do not support this? Who are these people? Why don't they support?

P5: Most of the time, they get information and resources from the health extension workers.

P8: All people have access to the information and resources. They tell the information to WDA leaders and she shares with 1 to 5 leaders. Those who heard it come and use the service. These leaders support women to get information and use the service. The information is accessible for everyone. If they have side effects, they consult the HEW and they change the method. There are women who use the service and sharing with others who are not yet using.

P6: There are also people from the health center and kebele who support PP family planning use by explaining its advantages.

P4: When women develop discomfort after using FP method for five years, they come and remove FP method and tell to other people not to use that. There are such cases in the community.

3. Please tell us your experience with accessing healthcare information and resources including PP family planning.
    - Do you feel you have a right to get healthcare information and resources including PP family planning services? Why/why not?
    - Do you think your experience is different from other people in your community? How? Please tell me more
    - Is there legal mechanism you can follow to ensure you get quality healthcare information and resources your need? Please tell me more

P7: I got information from HIV clubs in the schools about FP methods. I believe I have rights to get the information.

P8: There are people from the health center and health post who are telling us about different FP methods, its advantages and disadvantages. People also get lessons from other members in the community as well such as those who have a good balanced healthy family.

P3: I got information from family members who used the service. HEW and health center workers also provide us the information.

With this we have come to an end of the discussion. Anyone who want to add anything before we end the discussion or have any questions to ask?

Thank you all of you for your time and in case anyone want, he/she can get in touch with us through the details mentioned in the consent form.

FGD_SHC_G_280722

Focal group discussion conducted with girls at Sodo health center by Deginesh Dawit

SOCIDEMOGRAPHIC INFORMATION OF INTERVIEWEES

Code of respondents	Name of Keble	Age of respondents	Sex of respondents	Educational status (grade)<br><br>Of respondents
R1	Merkato	13	Female	Primary (grade 5)
R2	Dil Betigile	15	Female	Primary (Gr. 8)
R3	merkato	16	Female	Secondary (Gr.11)
R4	Merkato	14	Female	Primary (Gr.7)
R5	merkato	12	Female	Primary (Gr.6)
R6	Dil Betigile	15	Female	Primary (Gr.8)
R7	Dil Betigil	16	Female	Secondary (Gr.10)
R8	Dil Betigil	15	Female	Secondary (Gr.9)

Assets and Resources

Health services (Immediate PP family planning services) availability and Accessibility

I; Can you tell us the types of healthcare facilities that provide PP family planning services in your area, and what are the types of FP services are available at the healthcare facilities?

R6; this kind of services given at Health center, Otona hospital, meristope, types of family planning services available are three month injection and three year drug which is inserted at upper arm

R2; family planning services given after delivery or abortion is provided at any health facility that can provide first aid up to final treatment, can be given at health centers and hospitals,

pharmacy, meristope, in addition to that it can be given at private clinics, the types I know are three month injection, three year and five year drug inserted at upper arm.

R4; it can be given at here in health center and Otona hospital, from those the drug I know is three year drug

R3; it can be given at health center, Otona hospital, Christian hospital; I do not know thee types.

I; Are these services equally available and accessible to everyone who is in need and at any time? Please tell me more

R2; it depends on the area that means if you go rural health facility, even it is difficult to mothers to get the services, but in the urban area they may ask some questions like why you use this service, why is this important for you, it is not good for you, sometimes after discussing such issues they may provide it or sometimes they may refuse to provide it by saying this not allowed for you, this is especially when they saw you and early aged person.

R7; it is not provided equally for all they say you are so kid why you are interested on it, it is not good for you.

R3; they say why you need it you are kid do not use this kind of thing.

R1; why you need this thing because you are at earlier age, you can use it when you become large.

I; Can you tell us how long does it take you to access the facility and what type of transportation means used to reach the facility?

R8; to me to come health facility it takes around 20-25mins through walking and 10-15 minutes if I use motorcycle. I can use motorcycle and come through walking

R6; if we walk it takes around 15 minutes and if I use motor cycle it costs around fifteen birr, I can come by Bajaj, motorcycle, or through walking.

R4; for me it takes around 25 minutes through walking, most of the time I used to walk

R3; for me to come to this health center it takes around 30 minutes and we use either motor, Bajaj or come through walking.

I; Are healthcare providers receive and treat users with respect and dignity? If no why and how?

R4; some of professionals don't treat with respect they say you are substance abuser, smoker so we don't give you service go away, it is better if they advise rather than insult us
R2; when we go to health post or health center, some of them respect regarding of clothing but others treat irrespective of everything. But this not at all places there is institution which treat people regardless of educational status, economic status, language status, the opposite done in other facilities
With this, we have come to an end of the discussion. Anyone who want to add anything before we end the discussion or have any questions to ask? They make even people totally hates to go there due to that people are challenged to use such services.

R6; there are some people who treats equally but also there are some people who treat unequally, but they should treat all equally

I; who makes the decision for the use of household income? How does this affect to access the healthcare services including PP family planning?

R4; since my mother is housewife my father gives her for any required expenses, for example he gives her to fulfill monthly required expenses, brings oil and my mother saves and uses the given money wisely, when she finish it he asks about it gives her again, buys clothes for table and saves the remaining money. When she needs to use family planning, she discusses with father and receives it.

R3; in our homer my father and my mother discusses on how to use the income they get, they allocate as this for electricity, this for social life, this for monthly expenses, and the remaining money at Bank because there is good and bad we face. They both discuss on the

use health care services and family planning services. In our home, nothing is done without deciding together.

R2; this kind of things done according to home, for example in our home I'm living together with my mother only so she decides and allocates the money she get for everything for monthly expenses as well as to buy equipment's. She saves for unexpected health problem related costs

I; In your community how is the use of PP family planning seen? Is it acceptable for women to use PP family planning? Why/why not? Please tell us more about it

R2; it is not similar in every home, some family think even using family planning method can cause infertility others accept it as good thing, but young especially works at hotels should use it because they may face unwanted pregnancy. Currently the utilization and acceptance among mothers and young girl's very low people fear to use it immediately after delivery.

R4; most of girls who resided or works on street gave birth many times, so they get lesson from it and started to use family planning methods those are whom I know

R1; yes it has been get acceptance and used by street girls, because they are suffered from giving birth many times so started to use it currently.

I; In your opinion what should be done to improve access to the PP family planning services for all recently delivered mothers in your community?

R2; health professionals should respect and treat all the people equally, awareness creation should be made at school and media level as advertisement

R6; health extension workers and health professionals should go and teach young people at youth center

R4; health professionals should go and find street children and create association for them

<u>Practices, Roles, and Participation</u>

I; Is there any restrictions for women's mobility? What restrictions? How do the restrictions influence the access to the PP family planning services and supportive social networks?

R3; as I know nothing can restrict them from getting it

R2; she herself can also refuses to use it, her husband can prevent her from using it, her love/boyfriend also can oppose her, health professionals can also refuse giving it by saying you should stay at least you become strong

R8; nothing can be barrier for her if she wants to use it, it is her right to use or not to use it, she can use it at any time as her interest, it is very important if she receives it immediately, but her husband can prevent her from using it due to fearing from related infertility due to using of it.

I; In your community, do women assume leadership roles? What types of roles? Do these roles change by social characteristics, such as (dis)ability, economic status, health status, educational level, religion, culture, geographical location, and marital status?

R2; as I know they assumed as Keble leaders, those who assigned in such kind of places know the community problem in detail

R5; In my community there is one woman who is leader in Idir and calls the name of members and collects money, she selected there due to she is able to read and write

R4; I know one female who is leader for street children for all of us we selected her because she can fight with everyone in our group

R7; in our neighbor one woman selected in Equb as leader

I; Please tell us about your own experience with assuming any leadership roles?

R4; before three years I were class representative and had been selected by home room teacher, because at that time I were clever student and ranked third from whole class. I had also good behavior among students well and I do not hit students

R2; at school I used to be class representative, group leader, at church level I am kids teacher. At school I were clever student among my class mates due to that I selected to be group leader, I were selected to be representative because I am able to manage students well, at church level I were assigned as kids teacher because I like to stay with children.

I; Please tell us how your leadership roles affect your PP family planning services use

R4; for me I were kid at that time and it doesn't affected me, I were no intention to use it

R2; I have no contact with males and even I don't have boyfriend so I don't think to use it

I; In your community who decides and at what age people marry? What are the reasons for getting married at younger or older ages?

R4; in my community father decides at what age his children should marry. When their children graduated from college and university and starts job

R2; children themselves decides to marry without involving their family and they decides to marry without predetermined criteria, only by love and thinking that this guy can support me can lead our marriage financially, so I have to marry him they says.

R3; when children get their own job family says your age has been gone so you should marry at this time.

R; in ou community family decides at what age their children should marry, they say like your age has been reached to marriage and also you got job so it is better if you marry

I; What are your suggestions to improve women's roles and participation to enhance the use of PP family planning services?

R6; women who are aged should collect and teach and show direction for those who are younger than they are. They should advise them to do good things and not to practice bad things. They should advice young girls to use these services if they abort

R2; awareness creation should be made at every facility, at youth centers; if this is done they can understand and use the service well.

R5; currently it is good but advising youth should be encouraged

1. Knowledge, Beliefs, and Perceptions

I; In your community what is appropriate behavior for recently delivered a woman? How do they differ from adolescent girls and young women?

R8; they can be voluntary or not voluntary to use service it depends on their interest, but if a girl works at hotel she should use it, if young girl faces abortion resulted from rape the girl refuses to use it because she thinks that it cannot be happen again and she says I can take care of myself. For recently delivered mothers the health professionals force them to receive it they give them without their consent, before two weeks daughter of my aunt gave birth at Otona hospital and they gave her without taking her consent. Girls most of the time fear to use the service they are not use it officially

R2; mothers use the service if they want it but young girls not disclose using of it because they think that people can say something.

I; What are the social beliefs and perceptions that share women's expectations and aspiration for health care including PP family planning?

R2; there are different looking among the community, some of they can say is she not wants to give birth again, is she not wants to continue generation, this kind of people wants to give birth without birth spacing, others can say she did good thing she can take rest and can feed her baby adequately up to two or three years.

R5; some of them may say you did better, others may say why you used it immediately why don't you stayed if it is born it is the will of God can grow them

I; In your opinion who should make decisions regarding use of PP family planning services? What types of decisions do women make regarding use of healthcare including PP family planning services? What types of decisions regarding use of healthcare including PP family planning services are made jointly?

R6; she and her husband should discuss on it and have reach on decision because they both knows their capacity to grow born children, if they both decide together they decide to receive it, but in some mothers receive it without telling to their husband.

R8; husband and wife should decide together but sometimes mother do not discloses everything to her husband, decides lonely to receive the service, and hides about using of it for her husband. If they both decide together, they decide to stay for two or three years

R1; if one young girl attacked her mother and father should

R2; for married women her husband and herself should decide and if she is not married it is decided by her mother father and the girl herself, women I know are most of the time decides together with their husband they discuss on using of family planning methods after delivery, few of them do it by their self by thinking that I am the receiver of the burden and what burden he has. If they decide together they decides very good decision, they considers their economy status, ability to grow their children decides to use the service

Legal Rights and Status

I; In your community are women who delivered/**aborted** recently legally allowed to receive PP family planning service?

R6; without legal allowance this thing cannot reach tis label and the thing told too us to do is not bad thing which is helpful for the community, so it allowed legally

R4; it is legally allowed because when a mother give birth she can be done operation, she can bleed massively so she should use contraceptive to be prevented from getting pregnant as soon as possible

R3; if she is married woman it is legally allowed to receive the service, but if the girl is not married it is not allowed for her.

I; Is these same for boys and girls, people from different age groups, education background, ethnic groups, socio economic classes, excluded groups? How are they different? Why/ Why not?

R2; yes it is similar for all

R5; I think it similar for all

I; How do recently delivered/**aborted** women access PP family planning information and resource from the healthcare facilities?

R2; young can get the information from school, recently delivered mothers can get the serviced and information from health facility when they come to receive family planning services, to get delivery services when they bring their children to seek vaccination or treatment, they can get leaflets which contain such information from health center.

R7; mothers can get the information from health center and young can get from school through science learning.

R8; from Keble meeting

I; Is these same for boys and girls, people from different age groups, education background, ethnic groups, socio economic classes, excluded groups? How are they different? Why/ Why not?

R5; I think it is not the same for boys, most of the they don't want to receive the service

R2; most of boys even do not know services available and allowed to them to use the service.

I; Do the people in your community support recently delivered women to access PP family planning services and resources? How do they provide support? Who are these people? Why do they provide support? if not why? Who are these people who don't support?

R2; many of mothers says you did better now you can feed your baby sufficiently such kind of women are users of family planning methods, educated

R3; some mothers who received the service before can support hey by saying please take I also received it, I don't feel anything bad

I; Are there certain groups in your community who do not support this? Who are these people? Why don't they support?

R2; some women demoralizes those who received the service, such kind of women are normally born many children without using family planning methods previously at the time of family planning methods has not been started so far

R6; for example if one young girl who works at hotels or street or by rape abort and use the service her friends can say her is she need to repeat it, but she knows the risk can do as she chose.

R3; there are people who can say why she did this fast where will this go , this kind of women are not educated.

I; Please tell us your experience with accessing healthcare information and resources including PP family planning.

R6; I can get the information from my mother, my elder sister, from the school through science learning,

R5; I can get the information from the school, from my elders from my family, from the sister of my friend,

R7; from my pears, from the school science learning, my mother teaches us about it

R4; I can get from my friend, from street children association and from health center

R3; I get the information from my mother, father and from the school

I; Do you feel you have a right to get healthcare information and resources including PP family planning services? Why/why not?

R6; yes I have right because it is legally allowed and my age reached to get such information and services.

R4; I do not think so because I faced challenges when I want to use the service, they refused to give me.

R7; yes I feel I have right to get the service as well as information, all pole have right to use the service.

I; you think your experience is different from other people in your community? How? Please tell me more

R3; all students an get the information from school so it is similar with us

R1; others not included in street children association cannot get the information where we get.

I; Is there legal mechanism you can follow to ensure you get quality healthcare information and resources your need? Please tell me more

R7; I have no legal mechanism I follow to get quality service if they refuse to give me, but those who have money can get the service through their money if they refused provision through corruption or even can go to private clinics or pharmacy to get it

R8; I don't have legal mechanism to follow if they refuse to give service to me I willow to another clinic/facility

R6; if they do not give me the service I will leave it and go to pharm

Summary

This focal group discussion conducted between girl aged between eleven and sixteen at Sodo health center in 28/07/22.

Key findings were:- they know three month, three years and five years family methods. The decision for recently delivered mother should be made with her husband and for young girl it should be made between with her family and herself. Health care providers not provide the

service equally regardless of age, marital status. Not treat and respect all people equally. Not feel they the right to get the service there are people who demoralizes the utilizers of the service. To increase the service utilization awareness creation should be started at school and youth centers.

FGD_WL-B-010722

Focal group discussion conducted with boys at Waraza Lasho by Deginesh Dawit

SOCIDEMOGRAPHIC INFORMATION OF INTERVIEWEES

Code of respondents	Name of keble	Age of respondents	Sex of respondents	Educational_status (grade)<br><br>Of respondents
R1	Gurumo Woyde	19	Male	Secondary (grade 9)
R2	Waraza lasho	19	Male	Secondary (Gr. 11)
R3	Gurumo Woyde	21	Male	Secondary ((Gr.11)
R4	Gurumo Woyde	20	Male	Secondary (Gr.10)
R5	Waraza lasho	24	Male	Degree graduate
R6	Waja kerp	19	Male	Primary (Gr.7)
R7	Waja kero	20	Male	Primary (Gr.8)

Assets and Resources

I; Can you tell us the types of healthcare facilities that provide PP family planning services in your area, and what are the types of FP services are available at the healthcare facilities?

R3; as I know young female get unwanted pregnant and she was student at that time and she went to health center by wanting to terminate pregnancy and she received drugs and aborted the pregnancy. I heard this from when the community members talk about her, after that she has gone to universities. , if she need it such kind of services can be given at lasho health center, hospitals. The types are three-month injection, three years drug inserted at upper arm, condom for males

R1; this kind of services given at lasho health center when mothers come to receive delivery service and when they go to home after safe delivery and also it and be given at any time. I heard about three months injection.

R5; this service can be given at shola kodo health center, pregnant mothers come to health center and they diagnose them and give delivery service if they can and if it is beyond their capacity they refer to hospital

I; Are these services equally available and accessible to everyone who is in need and at any time? Please tell me more

R5; yes it available for including males but in the rural area it is not familiarized that males use this services, may be people live in urban area can use this service, even in urban area also males use when they use pension.

R3; yes all types are available for all who need it, but when client come to health center they are unable to get the service to get the service one person must be come at 2 o'clock to 5 o'clock or come after 8:30 o'clock because they exit for lunch at 5:00 and come again at 8:30.

R1; if one girl get unwanted pregnant due to that she may be stressed, and goes to traditional healers not disclose the event to any one at that time. she can be harmed or get die from it. if they want family planning method after this she can get it but most of the time it is unexpected occurrence for them so they restricted from repeated occurrence rather than using family planning method. In addition, they do not know about the family planning method and

abortion care can be given to them legally that is why they go to traditional healers and do not receive the drug.

I; Can you tell us how long does it take you to access the facility and what type of transportation means used to reach the facility?

R3; for me to come health center it takes around one hour and thirty minutes through walking. the road is not that much comfortable for any kind of people to walk. if we use even use motorcycle, I have to walk half road it is difficult to use motorcycle for whole road, during rainy season the bus can't go to our village at all. When they come to health center they used to walk, even people resided in away from us don't use health center at all due to far and difficult road to come here, most of the time they go to traditional healers, even they don't know availability of this service at health center level.

R4; our village is far from this health center it takes around 1;30 mins through walking, to come here we use motorcycle in sunny season or used to walk and at rainy season motor cycle can't go to our area. We face challenge when we come here.

R6; for me to come here it takes around 10-15 minutes and because of it is nearby to us we used to walk.

I; Are healthcare providers receive and treat users with respect and dignity? If no why and how?

R3; there are nurses and doctors at health center who care and respect clients if they come at normal time. for example to come this health center from our area takes 1:30, if they start journey at 3; 00 they reach at 5:00

at that they can exit to lunch so they should wait until 8:30, due to that patient become stressed. That means not they do not give respect for clients but they treat clients with respect those who come in above-mentioned time. Those who has need of family planning methods can their need and services providers provide the service for them with respect if they come on appropriate time

R5; shola kodo health center professionals receive the users with respect and dignity when couples come to use three years family planning method, they put sensation for mates also in different place of health center

I; Who makes the decision for the use of household income? How does this affect to access the healthcare services including PP family planning?

R3; in our home father decides and allocates the household income this for Idir this for Equb, this for food expenses and this for unexpected emergences, my mother also accepts and agrees on my father's decision, children also hears what they says. Because father is head of the home and he has right to buy or sell things because all things are in his control, if someone become sick in our home we have CBHI card and we can bring it can receive treatment, for family planning case I don't know whether they discuss each other or not.

R5; my father is farmer and all incomes they get from farm decides together, from that they allocate for this for Idir, this for Equb, this for treatment, this for to eat summer this for to eat winter, this for emergences they pay for community based health insurance on time and can receive treatment by using CBHI. In our both father and mother discusses on using family planning methods.

R1; my father is farmer and all income he get from farm make decision with my mother and can get health care services by using CBHI, but health professionals don't respect us and gives adequate treatment because we don't give them money in cash send us to buy drug from outside even if the case is emergency they don't treat us on time, Regarding family planning they both decide to use or not to use together currently they stopped bearing children by using family planning method.

I; In your community how is the use of PP family planning seen? Is it acceptable for women to use PP family planning? Why/why not? Please tell us more about it

R7; in our community I am not sure that whether the people started to use it immediately after delivery or abortion or not, I think people use it after three or four month of delivery but they don't tells whether they used it or not, we also don't ask them whether they received or not

R5; I can't tell you surely whether it is highly utilized or not among communities because since we are males they don't discloses whether they use or not use the service, even I don't

know young females use it after abortion or not. I do not know whether it got acceptances among women, I know they use it when they need it.

R8; for me it is first time to hear this because is that possible to use family planning method immediately after giving birth after abortion. Because we are males they do not even talk in front of us about it in detail, even when they are talking about it with their friends they become stop when we approach them.

I; In your opinion what should be done to improve access to the PP family planning services for all recently delivered mothers in your community?

R5; health professionals and health extension workers should go to and share awareness on utilization of it at youth centers and mothers meeting

Practices, Roles, and Participation

I; is there any restrictions for women's mobility? What restrictions? How do the restrictions influence the access to the PP family planning services and supportive social networks?

R2; in our community a recently delivered woman stay at home for at least one month or two month at that time she can't go to neighbor, church and market, even when her baby become sick her neighbor or relatives bring it to health facility and also she come to health facility to receive family planning methods after one or two months at that time she may get pregnant again, I know a woman who get pregnant after two months of delivery.

R5; in some family husband prevents a woman from using it if he wants to bear many children at that time a woman get pregnant again immediately after being strong from the first. However, if she decides together with her husband they can agree on issue, other than this there is no other challenge or restriction.

R3; may be a woman fears to what people can say me if I take it immediately after delivery without staying, such kind of woman can be risked from next pregnancy and child growth. Especially young females fear and hide themselves even from accessing abortion service from health care and uses traditional healers to receive abortion care and they never come to

health center to receive family planning method there is also no awareness for young girls about provision of abortion care legally at health facility.

I; In your community do women assume leadership roles? What types of roles? Do these roles change by social characteristics, such as (dis)ability, economic status, health status, educational level, religion, culture, geographical location, and marital status?

R6; Yes I know a woman who is leader at women's equb and she was selected due to her ability to read and write

R2; for example there are women who knows and understands everything well than males assumed as leader at Equb, leaders at different social organization, leaders at churches, there are woman who get front in everything, who thinks better than males. They were assumed due to their thinking status, ability to write and write communication skills
R5; women's take leadership responsibility in many social association like equb, edir, churches at schools, they assumed in those places due to their educational status, ability to read and write, ability to convince others,

I; Please tell us about your own experience with assuming any leadership roles?

R5; when I were at university I were served as leader in volunteer action, used to clean university and the town by coordinating students

R1; when I were at primary school I were selected as representative or leader of class

I; Please tell us how your leadership roles affect your PP family planning services use

R5; it doesn't affected me from using it, but at that time I had no girlfriend and no intention to use family planning method

I; In your community who decides and at what age people marry. What are the reasons for getting married at younger or older ages?

R4; in my communities couples going to marry decides at what age they should marry, when they are in love with each other, in previous time family decides the age of marriage

R6; In our community family decides the age of marriage when their son or daughter capable and being independent in economy

R3; a boy or girl going to marry decide their age of marriage when they fall in love with each other at that time they don't even hear any one, for example some people not get marriage even they have car, building or their own home and others get marriage even they have nothing even no adequate clothes they only thinks about loving of each other. Most of the time youths not hear others in case of marriage; they can get marriage even faced opposition from brothers or friends, and can refuse those who forces them to marriage.

I; What are your suggestions to improve women's roles and participation to enhance the use of PP family planning services?

R2; health extension workers are females and should teach the mothers

R5; females who received those services should share their experiences to those don't used the service by going meeting place and by collecting mothers

R3; because females are the first risk taker and males are also takes responsibility on child growth, so they should discuss openly and health professionals create awareness for mothers

1. **Knowledge, Beliefs, and Perceptions**

I; In your community what is appropriate behavior for a woman? How do they differ from adolescent girls and young women?

R1; in my opinion she may accept health professionals advice and receive the service because it is bad thing they do not recommend her to receive it.

R5; there is two types of looking regarding it if she is educated woman she can accept it easily if not she may refuse to receive it thinks it as bad things so health professionals should create awareness and convince them.

I; What are the social beliefs and perceptions that share women's expectations and aspiration for health care including PP family planning?

R5; people may have misconception regarding the use family planning methods immediately after delivery, but the mother should hear only herself and the health professionals. They can think the mother or young girl as having intention to do sex; because of people have low awareness about using family planning methods immediately after abortion.

R4; I don't know what can community say, but as my understanding if health professionals give them adequate awareness regarding it and provide the service it is good for her to control her from next time occurrence. Some people can admires the action and others can demoralize the action. As me 60% people can admires her because she and her God knows her risk. From her action, many those who do not know about it can get knowledge. People can say everything about it but the decision is on the hands of her, she knows her benefit and advantages it has for her.

R1; people can't say why she received the service because the risk taker is she herself, for example in our community one woman get pregnant again immediately after first delivery at that time people talked her negatively because she is educated person she is teacher so people negatively saw her action and they said why don't she stayed for at least one year, why she got pregnant before growing of first baby, why she don't used family planning methods

R6; In our community people admires a woman who gave birth to children after staying three or two years and negatively looks those who gave birth without birth spacing, they says why don't she uses family planning methods it has been given freely.

I; In your opinion who should make decisions regarding use of PP family planning services? What types of decisions do women make regarding use of healthcare including PP family planning services? What types of decisions regarding use of healthcare including PP family planning services are made jointly?

R3; regarding family planning both mother and husband should make decision together if father don't know about it she herself took decision and receive it. In some family mother by herself decides to receive family planning methods without informing to her husband by thinking that she more burden receiver than father in child growth. If they decide jointly, their decision should be better for both of them as well as their children

R1; both father and mother should decide regarding use of family planning methods. For any other health care services as our family if one-person get sick our father give money to get treatment and regarding family planning methods they both discuss on it. If they both decide on it they should stay for two to three years before getting another pregnancy and lets her to receive family planning methods as her needs

.

R5. Both father and father decide on using family planning methods otherwise there will conflict occur between based on their interest different. Some mothers even hide their utilization of the services from their husband and it is better if they decide together

Legal Rights and Status

I; In your community are women who delivered/**aborted** recently legally allowed to receive PP family planning service?

R6; legally it is not allowed legally because she is in pain and they should stay up to her health returned, they can be asked from it

R4; in my opinion it is not allowed legally those makes that should be asked

R1; I have two looking it can be legally allowed or not, when I say legally allowed may be her husband has over feeling to do sex within few weeks of delivery and she can get pregnancy again so it should be allowed legally, in other side if she receives it within three weeks and get the service it may harm her health status so it cannot be legally allowed. In general, for me it is not allowed legally

R5; in my opinion it is not legally allowed after four or five months she become strong she should use the service after being strong

I; how do recently delivered/**aborted** women access PP family planning information and resource from the healthcare facilities?

R5; from health facility when she come to get delivery service from health professionals who give delivery service. Either for young girls aborted se may get abortion service from

elsewhere from health facility or traditional healer can come and get the service from health center not to repeat it.

R6; they can get from school or health facility

R3; they can get from one to thirty leaders, health facility or banners or leaflets

R1; those girls who faced abortion can get the service from health center

I; Is these same for boys and girls, people from different age groups, education background, ethnic groups, socio economic classes, excluded groups? How are they different? Why/ Why not?

R5; it is not the same for both males and females most of the time to get delivery service and get both resources and information from health center, but boys not come to receive such services

R1; girls come to get abortion service but boys not so it not the same for all

R4; most of the time planning methods used by females not males, males not use condom for family planning purpose rather use it for to be prevented from HIV rarely

I; Do the people in your community support recently delivered women to access PP family planning services and resources? How do they provide support? Who are these people? Why do they provide support? if not why? who are these people who don't support?

R5; for example one to thirty leader woman encourages females to receive the service and also those who received the service previously can support and encourage them

R4; health extension workers visit home to home and teach the community to receive it

I; Are there certain groups in your community who do not support this? Who are these people? Why don't they support?

R2*; there are people who discourages the service by saying why don't stay, is the girl wanted to do sexual intercourse soon*

I; Please tell us your experience with accessing healthcare information and resources including PP family planning.

R5; I can get health information from health center, social media, banner, Facebook

R3; whe I bring people to health center, from school through biology learning, banners, from pears

R1; I get information from pears, from biology, when I bring people to health center, regarding postpartum family planning methods from nowhere.

R7; I get such information from FM radio

R4; from the school, television

R2; Biology teacher, pears

I; Do you feel you have a right to get healthcare information and resources including PP family planning services? Why/why not?

R5; yes I feel I have right to get the service because I'm also human being

R4; yes I have right to receive the service because I'm also citizen of the country

I; Do you think your experience is different from other people in your community? How? Please tell me more

R2; I don't think so

R7; all people experience is similar

I; Is there legal mechanism you can follow to ensure you get quality healthcare information and resources your need? Please tell me more

R6; I don't have legal mechanism I should follow

R5; yes I have I will report to health center head and concerned body

R3; yes I have report to upper body

Summary

This focus group discussion is conducted with seven young boys aged between eighteen up to twenty-four at Waraza lasho in 01/07/22. They had no sufficient understanding on postpartum and post abortion family planning method.

Key findings were:- young girls are refuse to use the service because they are not want to repeat the action. We are not sure whether the service got acceptance among mothers or not.

We do not have awareness regarding the service. To improve the access they should teach at youth center. People may say something if young girl or recently delivered woman get the service. They feel that it is not legally allowed to give the service for recently delivered or aborted girl.

**Interview no**	**01**
**Interviewee code**	**IDI_AF_ MF _29/06/22**
**Sex**	**Female**
**Age**	**24**
**Service year**	**Four**
**Job title**	**MCH focal person**
**Name of interviewer**	**Befekadu Bekele**
**Date**	**29/06/22**
**Time started**	**9:30**
**Time finished**	**10:54**
**Venue**	**Abela Faracho health center**

I: Health services (PP and PA family planning services) availability: Can you tell me the types of healthcare facilities that provide PP and PA family planning services in your area?

a. What types of FP services are available at the healthcare facilities?
b. Are these services equally available to boys, girls, young, old, married, unmarried, people from different geographic locations, religious or cultural background, language etc.? Please tell me more

R: Concerning availability of PPFP and PAFP is good in our health center. We provide counseling for mothers before postpartum family planning, we assess the current condition of recently delivered mothers, we provide them options of family planning methods and assess how they perceive post partum family planning utilization immediately after birth and we give them adequate time to think and decide. Most of the time they are willing to use post partum family planning after we counsel them to use. Educated mothers are mostly willing to use. However,

there are some barriers not to use PP and PA even after we provide them counseling, after they become willing to use after counseling. We health care providers have awareness on how to counsel mothers and we are trying our best to provide the services after we had training and we recently delivered mothers follow mothers and tell them that their uterus close to its pre-pregnancy size thirty eight days after birth. We build rapport with mothers to ensure confidentially. During the counseling times they disclose everything they have. We invite her husband after she becomes willing to use family planning and counsel them. During this time they are willing to take our advices and they become willing to use family planning immediately after birth. However, there are some barriers after they become willing to use family planning after birth and I will tell you later on about these barriers. Concerning abortion, mostly mothers come after post abortion. They don’t come for safe abortion. There are individuals who have got training about safe abortion but most of the time they are afraid and prefer to go to private clinics. But we counsel our mothers and sisters very well according to the training we received. We could be able to know more how to approach mothers during the training and we try our best but we sense that that there could be mothers who don’t disclose themselves. Aborted mothers come to our facility. Mother who fell down and aborted due to such reasons come. So, we counsel and discuss with them in detail and we tell them to use post abortion family planning so that they will not face any problem to their uterus. We counsel what they would face, the side effects unless they adhere to our counseling. We counsel about the importance of using post abortion family planning. There could be a mother who is anemic. For such mothers we counsel them to get rest become stable until they plan about next pregnancy and they becomes willing to take our advices. They start to use family planning based on their preference.

I: What types of FP services are available at the healthcare facilities? Would you add on this points please?

R: The types of family planning services being given in our facility are ,condom ,POP,COC, emergency pills ,depo which is given for three months, implanon which is given for three years, implants given for five years(jedle),IUCD or loop which prevents pregnancy from 10 to 12years. These all are available in our health facility. There is no problem with this regards. However, we have shortages of gloves most of the time. I don’t think that this is considered during procurement. Our kebele is very vast. We have twelve kebele. This is what we face usually we don’t have gloves. If we tell a mother to bring glove when she comes, she will never come to our

health facility again. Therefore, we try our best to avail gloves to mitigate this problem. In addition to this we face laboratory equipments.

I: Are these services equally available to boys, girls, young, old, married, unmarried, people from different geographic locations, religious or cultural background, language etc.? Please tell me more

R: These all groups use the services but there are barriers not to use the services. if we openly discuss and if we let them discuss freely in a private room, when we tell them that we ensure confidentiality, they become willing to use the service. However there are barriers related to resources. Sometimes we face shortages of short acting family planning methods. During this time we give them condom but they say that their husbands are not willing to use condom. We ask mothers to bring their husbands to counsel. Some are willing to bring their husbands and others not. Sometimes mothers are not willing to use implants because they feel discomfort. During this time we counsel them to use vasectomy and tuba ligation but they are not willing to use.

I: Would you elaborate more? please tell me more.

R:Those who are not married come to us and get counseling services from YFS or OPD and get the service they need. There is no problem with this regards but married ones sometimes challenge us because their husbands are not willing to use condom.

I : Can you tell me how far the PP and PA family planning services providing facilities are located? How long does it take for women to access the facilities?

What type of transportation means do they use to reach the facilities? How easy or difficult is it to use the transportation services?

Is there any cost associated with accessing the PP and PA family planning services? Please tell me more about this

Is the PP and PA family planning service is accessible at all time at the healthcare facilities?

Are there types of FP methods of users' choice in the healthcare facilities?

Are these services equally accessible to boys, girls, young, old, married, unmarried, people from different geographic locations, religious or cultural background, language etc.? Please tell me more

How easy could they access the PP and PA family planning services? Do you think that their access to the PP and PA family planning services are affected because of their background? How?

R: There are mothers who come from far places to get these services. it is very far from the health center. In order to solve transportation problem we counsel them to use long acting family planning methods. When mothers come from such distant places and when they don't get family planning methods they prefer they may be disappointed. Therefore, to solve such problems we spend adequate time with them ahead and discuss together and we let her choose the method she prefers. We tell mothers and teach them that they have right to change the methods if they feel discomfort within six months after getting the service. In most cases we counsel them to use long acting family planning methods because of inaccessibility/transportation problem.

I:What do you mean by this ?are there situations where you force them to use ?without her preference?

R: Most of the time they are influenced by their neighbors because if mothers in their neighbor use Depo provera ,she also want to use Depo. Regarding implants they have perceptions /common belief that these method is not good, because ‘‘*the implants move here and there throughout the* body’’. The come to us with such social norms. In this case we counsel them and we tell our experience that we use these methods. We counsel them that there is no as such problem if they use implants. We counsel them in detail, and discuss about transportation related problems. There are mothers who want use Family planning services given for one month and others want to use methods given for three months. We discuss about transportation related problems ,we guide them to use long acting family planning methods. We tell them the benefits

and its side effects they get these methods. In this time, they are willing to get the services after we counsel them.

They ask us whether it is possible to remove when they feel discomfort.

R: we tell them that it is possible to remove the implants. We counsel a mother to choose what she prefers.

There are mothers come from ‘‘ Abela Ajaja’’ Anka wocha which takes 40 minute if they use Car,which is very far.Others come from ‘‘ Hobicha border ‘’ , ‘‘ Abelala gafata which is near to Sidama region’’ which is very far.

I: What type of transportation means do they use to reach the facilities? How easy or difficult is it to use the transportation services?

R: they use motor bicycle to come to the health center. Those who live in nearby kebele come on foot.We treat them in a compassionate manner and give priority for those who come from far places after we convince others who came earlier. We convince people who came from vicinity and tell all the challenges that people who came from far places face.

I: Is the PP and PA family planning service is accessible at all time at the healthcare facilities? Are there types of FP methods of users’ choice in the healthcare facilities?

R: Frankly speaking , it is God who assigned us to serve the community. There are mothers who forget their appointment and tell us they forgot due to their busy schedule. So we give the services at any time. Even if it gets dark ,we give the service. we give the service even night time ,in absence of electricity. They may come to the health center and they rarely face challenges when there is shortage of PPFP ,in this time we give them condom. We don’t force mothers who come from far places to use long acting FP. We face shortages, sometimes we don’t get gloves. However we tell them all the options counsel them to get the services voluntarily. We tell them the benefits and side effects of all methods. They can get any of the services at any time. However sometimes we are afraid to insert implants and IUCD in absence of electricity. There is electricity fluctuations here. We may get electricity during day time and we don’t get electricity the whole night. We have fear that there is a possibility to insert implants deep into the skin which can bring complications to the mother. The same is true for IUCD. We have fear that if

we give the service without having electricity during night time, this can affect the uterus. Apart from this they can get service at any time.

I: Are these PP & PA FP services equally accessible to boys, girls, young, old, married, unmarried, people from different geographic locations, religious or cultural background, language etc.? Please tell me more

R: All are willing to use the services. No matter how long we counsel to get long acting family planning methods ,there are religious groups which don't allow them to use long acting f/p methods. Especially those who have 'Hawriyat' religion are not willing to use long acting family planning methods. They prefer the short acting ones. We ask them why they are not willing to use long acting F/P methods, and they respond to us that their religion does not allow to use these(LAFPM).we discuss with our close friends why they are not willing to use the services and they told us that their church doesn't allow them to use but they use it secretly in order to care for their children in a better way and to be economical.

I: Please tell me about your facilities' resources and assets. Please tell me the resources and assets belong to these health facilities to provide PP and PA FP service.

c. How is the use of the facility/Woreda health office resources and assets decided? Who makes the decision? Do you involve in the decision making? How do you think that affects access to the healthcare services including PP and PA family planning by women?
d. Please tell me about income of in this Woreda. Please tell me about your woredas income.
e. How is the use of the woreda's income decided? Who makes the decision? Do you involve in the decision making? How do you think that affects their access to the healthcare services including PP and PA family planning?

R: I have worked in other health center. There was separate room, the room is attractive ,there is chair attractive but here we don't have such resources. There is bed shortage in the health center. There is separate room for abortion service but there is lack of bed in the room. We reported this for concerned bodies. There is also scarcity of glove in our health center. We don't have speculum. We don't have equipments for abortion service. There is also shortage of long acting family planning resources.

I: How is the use of the facility/Woreda health office resources and assets decided? Who makes the decision? Do you involve in the decision making? How do you think that affects access to the healthcare services including PP and PA family planning by women?

R: When we face scarcity related to F/P we immediately report to the head. We discuss with all health workers on resources which need urgent decisions and we report to the head of the health center. We list down all what we need currently and give priority and the head of the health center responds accordingly. Some resources may be beyond the capacity of the health center ,may be the woreda has capacity to fulfill. Regarding speculum and other resources we discussed with engender health to support us.

I: Do you involve in the decision making? How do you think that affects access to the healthcare services including PP and PA family planning by women?

R: Regarding our participation in decision making , I came to this position very recently and it is about a month. As to my knowledge, we raise questions and discuss on gaps and then these questions again are raised during health center management meetings. However, we are not taking part in decision making and we don't any role apart from and informing the head what should be done. We did not have management meeting since I came to this position. Our participation in decision making has influence on these services. We have opportunity to report to the concerned body as early as possible if we take part in decision making. We can speak to the decision makers on behalf of mothers receiving the service. We can discuss on the real gaps of the facility so that the community can get benefit. Our participation during decision making mainly helps the community because we bring their questions and speak on their behalf. The same is true if we don't participate during decision making, the service and the community can be affected.

I: Please tell me about income of in this Woreda/health center. Please tell me about your woredas income.

How is the use of the woreda's income decided?

R:Some times the we face challenges. People are not getting drugs they need. We try to convince the patients to buy from private pharmacy during the time of shortage. The income of the health center is not as such bad because majority of the community is enrolled in community based

health insurance this is one of means for income. But I …..have no detail information about income since I came to this position(MCH focal person) because we don't have regular meeting. I used to take part in different meetings three years back and everything was clear and transparent. But we don't have such regular based meetings.

I: In your woreda how is use of PP and PA family planning seen? Is it acceptable for women to use PP and PA family planning? Why/why not? Please tell me more (what about boys and girls)

R: We have mothers forum in the kebele. We raise different issues during the forum. We used to conduct women's forum in three to four kebele. Mothers are very happy with such gathering. In order to prevent un intended pregnancy and related challenges we advice them to use family planning immediately after birth. We advice them to adhere to health care workers advices, We tell the advantage of giving birth at health facilities and the drawbacks of giving birth at home. In addition to this we teach mothers to listen and adhere messages being given by health care workers related to family planning immediately after birth. Personally, I share my experience while teaching about the benefits. We used to ask if mothers are willing to use family planning after we counsel them. Honestly speaking sometimes they disclose to us what they hide from their husbands. Sometimes we discuss with their husbands after taking their phone from mothers/wives. We tell all their rights in order to insert as well as remove when they don't want to continue. During this time, mothers of recently delivered women or their mother in laws motivate these people to use family planning during post partum period immediately after birth. They even try to convince her to use. Mothers/mother in lows usually say to her ''*Please agree with what the health provider is saying ,unless you will for get to use if you go home ,so please start to use family planning here ''this is what God provided you as opportunity. we would never had challenges if we had such services in our times but you are lucky''. This is what mothers advice her recently delivered daughter.*

However this is not the same across all kebele, among different mothers.Some recently delivered mothers are not willing to use family planning during immediate post partum period. When they were counseled by health workers to use family planning They say '' *Do you want to kill me? I'm not stable, the labour was painful ,my body is not healed because I suffered a lot during labour, my bleeding is not yet stopped ,are really doing this for my benefit?? Did you do that for yourself before during your pain?? Or are you just recommending ''*.In this time, we give

adequate time for her to think about and ask her willingness during her exit time ,when she becomes stable after checking her vital signs. Lastly we tell our own as well others experience of using family planning during post partum and post abortion period. We counsel about all the benefits of using F/P and tell them their uterus becomes stable and returns to its former position with in 38 days after birth. We tell them all the detail and they become willing to accept our advices.

I: What are the social beliefs and perceptions that share women's expectations and aspiration for health care including PP and PA family planning?

R: There are social beliefs and perceptions in the society that discourage mothers from taking family planning immediately after birth. But we try to refute myths and misconceptions while teaching the community.Some say " *once implants are inserted ,you can never remove them because it will be hidden in some part of the body. You will get surgery or it may also lead to cancer, so it is not good to use F/P immediately. it is better to take after some time after mothers who delivered become stable.''*

There are recently delivered mothers who never want to listen such a services 'I don't want to hear such a thing by this time''.

I:In your opinion what should be done to improve access to the PP and PA family planning services for all women in your community?

R:In my opinion awareness creation should be given priority to improve these services. In every kebele ,for every youth groups awareness creation should be given. Once they come to our facility, everything they need should be in place starting from youth friendly services. Awareness creation should include prevention of unintended pregnancy, and telling options to get emergency contraceptives services in case pregnancy occurs within 72 hours interval. As they come to our health facility after they get pregnant, we should give them adequate counseling services, and let them decide to get any service they need.

.

I: Please describe to me the typical Woreda health office officials/experts roles in your Woreda/community?

a. How is Woreda health office officials/MCH Focal persons occupied over the course of the day? Are there seasonal differences (campaign/political assignment) in how you use time?

R:Concerning my role and responsibilities concerned, I am punctual and follow registration books, see the arrangements of equipments as per the standard, sterilize materials, monitor the sanitation of the rooms. Sometimes we have meetings at woreda level ,other than this we are occupied with our routine activities. Because I have the responsibility I am engaged and work day and night.

I: Do Woreda health office officials like you have restrictions/barriers on the accessibility? What restrictions/barriers? How do the restrictions/barriers influence the access of the PP and PA family planning services and supportive social networks?

b. In your Woreda do the restriction/barriers change by social characteristic, such as (dis)ability, economic status, health status, educational level, religion, culture, geographical location, and maritalstatus?

R:Yes these could be the barriers as I have tried to mention earlier. A mother who came to get the service may be bleeding but we don't have gloves most of the time. Sometimes we borrow gloves from private clinics, so mothers will suffer a lot because of waiting time. As a result a mother may prefer traditional practioners. We who are working in MCH have extra burden and challenges than other case teams.

I:What restrictions/barriers other than what you have mentioned? as (dis)ability, economic status, health status, educational level, religion, culture, geographical location, and maritalstatus?

R: Yes there are restrictions. For instance unmarried ones/adolescents come to us secretly, even without registration.

I:Why do they come in such manner?

R:Because of the community perception. If a girl gets pregnant without formal marriage, the community point at her and say ‘‘ *she looks pregnant and came here to abort’’ every body says*. Therefore adolescents don’t want to be seen other community members, that is why they hide themselves when they come to get the services. Therefore, we counsel them in detail and take all their history and take consent, we give them abortion services, if it is beyond our capacity we send them to hospital to get the service or other private clinics like meristopes and we counsel such adolescent girls to use family planning after they get abortion services. There are places where remote areas and people who come from such area have no information and this can also be one of the barriers.

I: Do you participate in any meetings, workshops, trainings, pregnant women conference, family, community,government, or other social networks organizations? Please tell me more.

Please tell me how your participation in these organizations affect the PP and PA family planning services provision

R: I have participated in women conference, I have also participated in trainings on post partum family planning given by ENGENDER .we were four in number who received the training. After I participated in the training I could be able to teach the community about ANC and post partum Family planning in the kebele. In addition to this I have received training on safe abortion and post abortion services.

I: In your Woreda do women assume leadership roles? What types of roles? Do these roles change by social characteristics, such as (dis)ability, economic status, health status, educational level, religion, culture, geographical location, and maritalstatus? Please tell us about your own experience with assuming any leadership roles? Please tell me how your leadership roles affect the PP and PA family planning services provision

R: Women take part in leadership roles, for example there are women who are leading different sectors in the woreda. women sectors, road and transport services, and other sectors as well. They are qualified ones.

Previously less attention was given for post partum and post abortion family planning services. Less attention was given also to safe abortion services so far. Had there been more attention given for these services, there would have increased awareness among the community. I myself gave attention to these service after I have got training. There is improvement of the service since we took the training, the performance is good. When women assume in leadership positions they can play many roles. Eg. women who are working in women office have played many roles.

I: In your Woreda/community who decides at what age people marry? What are the reasons for getting married at younger or older ages in this Woreda? Please tell me how their age at marriage affects their PP and PA family planning services use

R:Females get marry at early stage. They get marry when they are about fifteen. They get marry due to family ,peer influence ,due to lack of information. Males get marry when they are twenty and above. There are girls who even start to see their menarche or the first occurrence of menstruation after they get married. In this case their body is not mature enough to conceive so we counsel them to wait some time to get pregnant. Therefore early marriage has influence and it can affect these services.

Now we are going to discuss about the issues faced by women in accessing PP and PA family Planning services in your opinion.

I: In your Woreda what is appropriate behavior for a woman after delivery and abortion regarding the use of PP and PA family planning? How do they differ from adolescent girls and young women?

a. How do these believe influence health behavior including the PP and PA family planning use?

R: Regarding this there are two kind of perceptions found in the community. Some people are very happy to use the service and willing to accept the services. The mother or mother in law of recently delivered mothers who strongly encourage their daughter to use the services. On contrary, there are people who are resistant and not willing to accept the services. They always speak about its side effects. They say I suffered a lot even in the past my body is almost burned ,lost weight ,so I don't want to use it immediately ,let me have enough time get rest and then I will think about it." We counsel these people thoroughly. However those who have better understanding about the services are very happy and willing to accept and even they bless us for giving the service.

I:What are the social beliefs and perceptions that share women's expectations and aspiration for health care including PP and PA family planning?

R: Yes there are social beliefs. Some people are not happy. Say ''Aysi eesoyiona, aaxadfiyona?'' meaning why are you rushing to give this services immediately after birth?.Sometimes health workers let the mothers or mother in laws of recently delivered daughters go out in order to counsel the recently delivered mother about family planning after birth and their mothers are not happy and ask their recently delivered daughter what was said by the health worker. She responds them that the health workers counseled them to use family planning and that will help her to manage her life, economy. In this time mothers get disappointed. They don't want the health care workers to interfere on such matters. Some of them in salt the health care workers. Then they discourage their recently delivered daughter not to use family planning immediately after birth. On contrary the others encourage her to use by telling all the reality that she couldn't be able to lead her life and family life properly if she did not use family planning.

I: In your opinion who should make decisions regarding use of PP and PA family planning services? What types of decisions do women make regarding use of healthcare including PP and PA family planning services? What types of decisions regarding use of healthcare including PP and PA family planning services are made jointly?

R: Recently delivered mother has 100% right to use family planning immediately after birth.She should decide to use. No body can prevent her from using the services. However, mothers are not in a position to decide because of fear of their husbands. They expect their husbands' decision. If her husband is around we call him and counsel them jointly and tell all the benefit and side effects of each family planning methods. We assure her husband that it is possible to remove any time they want and they will agree after we counsel them jointly. Their husbands become willing to use any method the health workers recommend for their wives. It is good if they jointly decide. Because there are uneducated people who start to discontinue when they loose their weight. Un educated husbands can let his wife to stop or discontinue using family planning. In addition to this he can abuse or mistreat health workers because of helping his wife to get the service. That is why I say it is important to decide jointly. Starting from family planning being used daily until family planning given for 12 years ,it would be nice if it is given for couples after they discuss and decide jointly.

Now we are going to discuss about the marriage related process prevalent in this community.

I: In your community are women who delivered recently legally allowed to receive PP and PA family planning service?

- Is these same for boys and girls, people from different age groups, education background, ethnic groups, socio economic classes, excluded groups? How are they different? Why/ Why not?

R: yes they are allowed legally to receive PP and PA family planning services.

I: How do recently delivered/aborted women access PP and PA family planning information and resource from the healthcare facilities?

- Is these same for boys and girls, people from different age groups, education background, ethnic groups, socio economic classes, excluded groups? How are they different? Why/ Why not?

- Do the people in your community support recently delivered/aborted women to access PP and PA family planning information and resources? How do they provide support? Who are these people? Why do they provide support?
- Are there certain groups in your community who do not support this? Who are these people? Why don't they support?

R: the first thing is that they get information from us because we gave awareness on this issue. At the same time they access PP and PA family planning when they come here to the health facility. Before we receive training we did not have a plan. They also get information from youth friendly services from kebele.

I:Do the people in your community support recently delivered/aborted women to access PP and PA family planning information and resources? How do they provide support? Who are these people? Why do they provide support?

R:yes the community support recently delivered women to access PP and PA family planning information and resources even though there are people who don't support because of low awareness they have.

I: Do you feel they have a right to get healthcare information and resources including PP family planning services? Why/why not?

R: yes they have right to get health care information including PP family planning.

Is there legal mechanism you can follow to ensure they get quality healthcare information and resources they need? Please tell me more

R: Yes

I: With this we have come to an end of the discussion. Do you want to add anything before we end the discussion or if you have any questions you want to ask?

R:Thank you very much.

Summary

This is in-depth interview conducted with MCH focal person in Abela Faracho health center on 29/6/2022.

- ✓ There is shortage of resources in the health center. Mainly there is lack of gloves, speculum, laboratory equipments, no separate room and there is absence of electricity if mothers come during night time. This can be a challenge to insert long acting family planning methods.
- ✓ There are times where they face shortage of short acting family planning, so they give condom but husbands of recently delivered women are not willing to use condom
- ✓ Proper Counseling service helped couples to decide about immediate post partum family planning.
- ✓ The long acting family planning methods are recommended by the health workers due to inaccessibility/distance from the health facility
- ✓ There are some religious groups which do not allow their followers to use family planning, so, religion is identified as one of barrier for family planning services
- ✓ Involvement of mothers or mother-in-law of recently delivered women during counseling influences immediate family planning utilizations because recently delivered mothers accept what is said by their mothers or mother-in-law.
- ✓ There are misperceptions about implants because people perceive that the drug moves throughout their bodies, that is why some people don't prefer long acting family planning methods.
- ✓ Neighbors/peer groups can influence the type of family planning to be used. If the mother lives in neighbor has misperception towards a certain family planning, she can influence the recently delivered woman's decision to use immediate post partum family planning method.
- ✓ Adolescent girls have restrictions/barriers to use family planning after abortion because they don't want to be seen by others, so they hide themselves and don't feel confident
- ✓ Participating in meetings, receiving training on immediate post partum and post abortion has positively influenced immediate post partum and post abortion family planning services

- ✓ women do not participate in decision making because they expect their husbands to decide on every matters.

In-Depth Interview conducted with husband of recently delivered mother at Abela Farecho

.

Sociodemographic information of interviewee

Code	Keble	age	Sex	Educational-status	Job-status	Number of children
IDI_AF_H_290622	Abela Faracho	38	M	secondary	Car driver	two

Assets and Resources

Discussion

I; Health services (PP and PA family planning services) availability: Can you tell me the types of healthcare facilities that provide PP family planning services in your area?

R; Such kind of services are given by health centers, in hospitals, in the medium private clinics recognized by the government

I; what types of FP services are available at the healthcare facilities?

R; *There four types of FP services given by those institution, one of this is the FP method given for 12 years. For example my wife has taken 12 months FP method inserted in uterus two times, when she delivered first baby she had taken it after three month of delivery and stayed for eight years then health professionals removed it when she think another pregnancy. Then after this delivery, also she has taken loop within twenty-four hour of delivery. Other types of FP methods*

are drugs given for three months, three years four years and five years, this all types of family planning methods I mentioned above were also given here.

I; Are these services equally available to boys, girls, young, old, married, unmarried, people from different geographic locations, religious or cultural background, language etc.? Please tell me more

R; there all types of family planning methods that I tried to tell you above were available for all kinds of people who need it. There is condom also available for males put in the mini boxes in the different places of health center compound for those who are not married to protect themselves from different disease and unwanted pregnancy.

1. Accessibility of the PP family planning services

I; Can you tell me how far the PP family planning services providing facility is located? How long does it take you to access the facility?

R; for example we get such type of service from this health center, and it is little far to us and it takes around 30mins through motor cycle

I; what type of transportation means do you use to reach the facility? How easy or difficult is it to use the transportation services?

R; we use Ambulance to get delivery service and motorcycle for other purposes, getting ambulance is an easy for us because we have the a drivers phone number and can call him and access it, motor cycle is also accessible for us it is not difficult to get it for other purpose.

I; Is there any cost associated with accessing the PP family planning services? Please tell me more about this

R; the service is provided freely without cost as I know

I; Is the PP family planning service open at all time and is accessible with short waiting time at the healthcare facility?

R; my wife used loop two time as I told you before at that time she got the service easily without waiting much time, in case of current delivery she got it immediately after delivery

I; Are there types of FP methods of users' choice in the healthcare facility?

R; yes they have, for example after my wife delivered safely they showed and taught us together the types available at their hand with their advantages then we chose loop because she used it previously and was comfortable for her

I; Do healthcare providers receive and treat users with respect and dignity?

R; actually I'm not staying my full time here in Wolaita my work place is Hawassa, but in this short period of time I saw their caring and compassionate they show to clients when they give the services. Even before giving the service, they taught us together and created awareness, that is why we decided to receive it immediately.

I; Are these services equally accessible to boys, girls, young, old, married, unmarried, people from different geographic locations, religious or cultural background, language etc.? Please tell me more

R; yes those who needs it can access it freely and easily regardless of their background, because there is no related cost for the service people from different economic status can access it. Regarding males there condom available everywhere in the health center compound and those who are not married and have multiple sexual friends can use it without asking someone's permission only depending on their need. However, those married men do not use FP methods because of their wife uses it and they can be protected from unwanted pregnancy; they can saw either availability or accessibility when they come with their wife to receive the service.

I; How easy could you access the PP family planning service? Do you think your access to the PP family planning services is affected because of your background? How?

R; in the previous two days I was with my wife and tried to look how easy is accessing the service, first of all they created awareness among both of us and has been given loop to my wife which will serve for 12 years. Even the procedure is not that much painful and discomfurtable. Good, they treat all people equally regardless of their educational background, economical class, even sometimes people come from rural area don't know about how they can get the service and even they don't know to bring registering card at that time they show the ways and give the service in respectful manner

2. Means to access the PP family planning services

I; Please tell me about your household resources and assets. Please tell me the resources and assets belong to you.

R; for us we have household utensils like TV, bed, sofa, fridge and the kitchen utensils, we have no our own house and house building place, we are thinking to buy house or the place for house building if it is the the will of God. Nothing is my own, all things we have is for both of us.

I; How is the use of the household resources and assets decided? Who makes the decision? Do you involve in the decision-making?

R; after discussing together we save some money from monthly income and buy something which is important for us. We both decide to buy or something without my consent nothing can she do and without her consent nothing can I do, so both of us involve in decision-making on our resources and assets of our household.

I; How do you think that affects your access to the healthcare services including PP family planning?

R; our discussion in everything used us very well in including PP FP, also for example we discussed together and she received loop stayed eight years without getting another pregnancy now our first child is eight years old and she can care or her elder brother. The other benefit is our first daughter fed breast until she turns three years that is why now she is strong enough to carry and handle her brother and do some household activities. Because we used long acting family planning methods our daughter grown safely and fulfilled all requirements for her including teaching in the private school, she grown as rich people child, through food, clothes or other requirements we don't challenged even in this expensive life because she is only one for us and buying anything for one child is not difficult. We do all activities through communication and discussion that's why she has been gotten loop after this delivery by fearing what will happen in the next time because I will return back soon to my work place.

I; Please tell me about your household income. Please tell me about your own income.

R; source of our house hold income is my monthly salary with long journey, per time allowance and house allowance, my wife is house wife she has no her own income currently, our monthly income is around 9000-10,000 with allowances.

I; how is the use of the household income decided? Do you involve in the decision-making?

R; our household income determined after discussing together we set for house rent and monthly household expenditure, our daughters school payment and payments for social life those are fixed expenses and we save the remaining money for unexpected events like health care services and death in the house of relatives and for our future plan.

I; Who makes the decision?

R; we decide together for all

I; How do you think that affects your access to the healthcare services including PP and PA family planning?

R; as I told you before we set all expenses and plan for unexpected events like disease, for that matter we put some money, if she or my daughter become sick she bring them even I'm there in workplace, regarding PP FP we do it by agreement like others.

I; In your community how is use of PP family planning seen? Is it acceptable for women to use PP family planning? Why/why not? Please tell me more

R; it is fifty by fifty, that means half of people accepts and understands it and uses it, but half of them want to receive it later after two or three month of delivery, because they think that receiving that time may create discomfort for them. But few of them wants to not stay and give birth with in short period of time not accepts it. So different people have different idea regarding it.

I; In your opinion what should be done to improve access to the PP family planning services for all recently delivered mothers in your community?

R; to increase access and utilization among all child bearing age groups, all health professionals, especially who works at family planning provision class should create awareness exhaustively by going to community. By nature our community is learner and changeable so collecting them at Keble or village level continuously until they bring change. To bring more change those who benefited from the service like my wife have to share the experience and motivate others to use the service, because they are the actual persons who used the service and understands well its advantages and can be heard by the community as role model.

Practices, Roles, and Participation

I; Please describe to me the typical women’s roles in your community?

R; women’s have several roles at home and outside from home like, caring children, preparing food for family, cleaning their home and their environment, washing all family’s clothe, keeping hygiene of their children.

I; how are women and girls occupied over the course of 24 hours?

R; women more of occupied in household activities and busy in activities related with children, but most of the time girls spent most of their time at school after school they do their homework and read if they want, after doing this all they help their parents by preparing food and cleaning their home.

I; Are there seasonal differences in how women and girls use their time?

R; During winter time our daughter go to school early morning, for that matter she wake-up early at the morning and prepare breakfast and lunch to her and she take her to school, then return to prepare family breakfast. During summer time, the school become close and she can get rest from activities related with our daughter school sending.

I; what kind of work do you do?

R; my major job is working as car driver in private institution, secondly I also help my wife by doing household activities like washing our clothes, cleaning the house and even cooking food, every in our neighbor can be witness for this this. When my wife gave birth to our first baby she does not went to her family because their home is too far from ours. And at that time I got permission from the working place and treated her and my daughter well as a mother, by preparing the required food, washing the new born clothe until she become strong.

I; Do these works change when your age or status changes (young, unmarried and married, older)?

R; It will not change because when I care more for her she will give attention for me, may be our children become grow up and starts to help us I will take rest from doing such activities otherwise I will continue until I become old man. It is not shame for me rather than helping each other

I; Do you have barriers on your access? What barriers? How do the barriers/restrictions influence your access to the PP family planning services and supportive social networks?

R; there is no barriers which her from using it, she may not refuse to receive it if she faces opposition from her neighbor or from her friend, because we did all things by agreement with me

I; Do you participate in any family, community, government, or other social networks organizations? Please tell me more.

R; yes she participate in every social, spiritual or social networks, she also participate in any social networks which may need family contribution.

I; Please tell me how your participation in these organizations affect your PP family planning services use

R; it doesn't affect her from using PP or PA FP services, under the God she is mine and I'm her so we both are one so no one can influence or affect our decision. She does not accept others idea other than mine.

I; in your community do women assume leadership roles? What types of roles?

R; currently the direction that government set is very good for women, which brings them to leadership. There are so many women are take part in different leadership role, for example in my workplace woman serving the leadership role, in this health center also woman assigned in leadership positions, also in political positions women serving as leader equal with males. This kind of work is interesting and should be encouraged because women are honest than males.

I; Do these roles change by social characteristics, such as (dis)ability, economic status, health status, educational level, religion, culture, geographical location, and marital status?

R; no primarily they assigned for the position based on their leadership capacity, communication skill, educational status, based on these criteria they come to leadership position in any level, like at village, Keble, woreda, zonal, regional up to federal

I; Please tell us about your own experience with assuming any leadership roles?

R; yes before two years I used to lead and coordinate driver's association

I; Please tell me how your leadership roles affect your PP family planning services use

R; it does not affect PP or PA FP use for me

I; In your community who decides at wh-at age should people marry?

R; the person who going to marry decides at what age he/she has to marry

I; What are the reasons for a women getting married at younger or older ages?,

R; after the age of puberty they starts thinking of marrying someone and living together, most of the time they decide to marry when they think that they are economically independent and able to take family responsibility.

I; Please tell us about your own marriage experience? How old were you when you marry? What are the reasons you she get married at that age?

R; I got marriage when I was 32 years old adult and the reason initiated me to marry was my age and I were economically capable to lead family.

I; Please tell me how your age at marriage affects your PP family planning services use

R; it helped me very well because I were mentally matured to differentiate what is good and bad for me and accepts what health professional teach me regarding the use PP or PA FP.

I; what are your suggestions to improve women's roles and participation to enhance the use of PP family planning services?

R; as you told u before those women who used it have share their experience to others in their community and health professionals should teach about PP PA FP method for reproductive age woman even they come to health institution for other purposes.

Knowledge, Beliefs, and Perceptions

I; in your community what is appropriate behavior for recently delivered woman regarding use of PP and PA family planning services?

R; one out of ten may be has negative feeling regarding it but most of them for sure can receive it with pleasure. Few of them my want to receive it later after they become strong and starting of menstruation so those who work in delivery class should teach and convince them.

I; How do they differ from adolescent girls and young women?

R; Those adolescent girl and young women can receive it at any time

I; what is the appropriate behavior for a recently delivered mother? How do these believe influence health behavior including the PP family planning use?

R; in our community it is not allowed for recently delivered mother to go out of home for at least six weeks of postpartum, so even if she don't received it after delivery it is difficult for her to come to health facility to receive it.

I; what are the social beliefs and perceptions that share women's expectations and aspiration for PP family planning?

R; most of them accept it as good culture, but few of them may say as why she received this at this time since she is in pain? Ohm, are they think to do sex at this time before drying of blood? And the like.

I; In your opinion who should make decisions regarding use of PP family planning services?

R; in my opinion they both wife and husband should have to discuss on which method they have to use.

I; What types of decisions do women make regarding use of healthcare including PP family planning services?

R; some of mothers decide to receive PP PA FP without discussing with their husband and this can lead them to conflict.

I; What types of decisions regarding use of healthcare including PP family planning services are made jointly?

R; most of the time if there is discussion between them they decide to use PP FP.

Legal Rights and Status

I; in your community are women who delivered recently legally allowed to receive PP family planning service?

R; Yes it is allowed legally because if it is illegal government do not give permission to do such thing.

I; Is these same for boys and girls, people from different age groups, education background, ethnic groups, socio economic classes, excluded groups? How are they different? Why/ Why not?

R; yes it is the same for all

I; How do recently delivered women access PP family planning information and resource from the healthcare facilities?

R; she can share experience from mothers who used it previously and can get information and resources from health professionals during delivery time.

I; Is these same for boys and girls, people from different age groups, education background, ethnic groups, socio economic classes, excluded groups? How are they different? Why/ Why not?

R; yes it is similar for all

I; Do the people in your community support recently delivered women to access PP family planning infatuation and resources? How do they provide support? Who are these people? Why do they provide support?

R; yes for example my give information directly or indirectly to others come to visit her through kidding, she used to say why not you take rest before becoming pregnant like me by taking it in the health center after delivery.

I; Are there certain groups in your community who do not support this? Who are these people? Why don't they support?

R; there is few of them who opposes the idea especially taking of loop, by saying you took a 12 years drug why not five or three years, why you don't waited until you become strong?

I; Please tell me your experience with accessing healthcare information and resources including PP family planning.

R; I can get the information regarding health care is from radio, television, from the community and from health institution and regarding PP is only from this health center and from my wife.

I; Do you feel you have a right to get healthcare information and resources including PP family planning services? Why/why not?

I; yes I have right to get the information and resource regarding PP and PA FP because I am also one of the citizen of this country

I; Do you think your experience is different from other people in your community? How? Please tell me more

R; some people may get from leaflets given from health center and home to home visit of health professional, since I am not available at home all time due to my work behavior unable to meet them when they make home to homme visit teach people.

I; is there legal mechanism you can follow to ensure you get quality healthcare information and resources your need? Please tell me more

R; if I do not get the quality service which I belong to use, I will ask my right through health center head and report my complain through suggestion provision box found in the health center compounding.

Summary

This interview conducted with recently delivered mother husband at Abela faracho health center. He was learned up to secondary and now he is car driver.

Key findings were;- there are all kinds of family planning methods available for all who need it. Health professionals respect people regardless of their background. Most of people are currently using service. To improve the access mothers who received the service should share their experiences to pregnant mother. Receiving the service is very important because they stay at home for long period and may get another pregnancy.

Interview details

**Interviewee**	Husband
**Age**	30
**Interviewee code**	IDI_BA_HU_270722
**Name of interviewer**	Beimnet Desalegn
**Date**	27/07/2022
**Time started**	4:46 lt
**Time finished**	**5:19 lt**
**Venue**	Bedesa health center

Assets and Resources

Discussion

Now we are going to discuss about the Healthcare resources and assets available to recently delivered mothers in your area.

I: - Health services (PP and PA family planning services) availability: Can you tell me the types of healthcare facilities that provide PP family planning services in your area?

a. What types of FP services are available at the healthcare facilities?
b. Are these services equally available to boys, girls, young, old, married, unmarried, people from different geographic locations, religious or cultural background, language etc.? Please tell me more

R: - I know Bedesa health center… I was using this health center for any health-related issues. In addition, I go to Sodo Dr. Getu's clinic for better service. My wife has previous abortion history… eee and post-abortion care were given here at Bedasa health center. After three months she got pregnant again. We had ANC follow-up here … and thanks to God she gives birth on 16/11/2014 Ec…

I don't know any family planning methods… hahaha

I: - Accessibility of the PP family planning services

c. Can you tell me how far the PP family planning services providing facility is located? How long does it take for you to access the facility?

d. What type of transportation means do you use to reach the facility? How easy or difficult is it to use the transportation services?
e. Is there any cost associated with accessing the PP family planning services? Please tell me more about this
f. Is the PP family planning service open at all time and is accessible with short waiting time at the healthcare facility? Are there types of FP methods of users' choice in the healthcare facility? Do healthcare providers receive and treat users with respect and dignity?
g. Are these services equally accessible to boys, girls, young, old, married, unmarried, people from different geographic locations, religious or cultural background, language etc.? Please tell me more
h. How easy could you access the PP family planning service? Do you think your access to the PP family planning services is affected because of your background? How?

R: - I am from 01 kebele … it is very close to health center … we can walk to come to health center… and even if we need we come with car … I am driver… deriving government office car. It takes about 5 minutes if we walk …

When we come here for family planning or other maternal health services I did not pay for service or for drugs that we took. It is not only here but when we go to the hospital I did not pay for service. Even during her abortion time, we got free service.

Regarding the health center service, they provide for the community … I was satisfied with every visit to the health center. I don't know about another service in the health center but maternal service is very nice. Any time we can call or present to the health center for required service or care. They are working for 24 hours.

When I came to the health center they did not serve me because I am their relative… or because looking at my background or educational status they just serve every client equally. We did not pay for service …

I am one hundred percent sure that maternal health care service is very comforting and friendly for women. I did not spend a coin during delivery.

I: - Means to access the PP family planning services

i. Please tell me about your household resources and assets. Please tell me the resources and assets belong to you.

j. How is the use of the household resources and assets decided? Who makes the decision? Do you involve in the decision making? How do you think that affects your access to the healthcare services including PP family planning?

k. Please tell me about your household income. Please tell me about your own income.

l. How is the use of the household income decided? Who makes the decision? Do you involve in the decision making? How do you think that affects your access to the healthcare services including PP and PA family planning?

m. In your community how is use of PP family planning seen? Is it acceptable for women to use PP family planning? Why/why not? Please tell me more

R: - she is a housewife … she completed college and search for work. And I am a government office car driver. Haha, you know derivers income … so in this way, we live …

Yeah, we do everything discussing each other … we save money from what we get. The only income is mine … but we use it together… discussing each spend of money.

Now … I know my friends who were using contraceptives for a long period of time… standing from this I can say youth community members are positive towards the family planning method. I have one friend who used it for four years and delivered after for years recently.

I: - In your opinion what should be done to improve access to the PP family planning services for all recently delivered mothers in your community?

R: - in my opinion, it is better if the community member has meetings and discussions about contraception regularly in this way the utilization of family planning may be improved. If there is a feedback system for users and discussion and appropriate action were taken by the concerning body to improve the service quality there will be a high flow of users.

Practices, Roles, and Participation

Now we are going to discuss about your women's and your role and responsibilities.

I: - Please describe to me the typical women's roles in your community?

- How are women and girls occupied over the course of 24 hours? Are there seasonal differences in how women and girls use their time?

R: - most of the time women are occupied by household activity … they care for the baby, wash cloth, cook at home for their family, and generally, women are busy. The male partner engaged in activities out of the home.

I: - What kind of work do you do? Do these works change when your age or status changes (young, unmarried and married, older)?

R: - my responsibility is to lead my family in an exemplary way… to supply money required for living …

I: - Do you have barriers on your access? What barriers? How do the barriers/restrictions influence your access to the PP family planning services and supportive social networks?

- In your community do the restrictions/barriers change by social characteristic, such as (dis)ability, economic status, health status, educational level, religion, culture, geographical location, and marital status?

R: - my wife was using contraceptive … that is for our own benefit… we have a plan when to give another birth.

I: - Do you participate in any family, community, government, or other social networks organizations? Please tell me more.

- Please tell me how your participation in these organizations affect your PP family planning services use

R: - Yes, I have experience of participating in different training and meeting … in my participation, I understood the importance of using family planning, and … we see it as valueless because we get it freely but do you imagine how much we cost for it if we pay for service?

I: - In your community do women assume leadership roles? What types of roles? Do these roles change by social characteristics, such as (dis)ability, economic status, health status, educational level, religion, culture, geographical location, and marital status?

- Please tell us about your own experience with assuming any leadership roles?
- Please tell me how your leadership roles affect your PP family planning services use

R: - In leading position holding, we can say it is fifty by fifty with a male in our woreda. I can mention many women in positions of women and child affairs, city sector affairs, road, and transportation affairs, were female. Here there are many women in leadership positions. People are assigned positions based on their merit and educational quality, individual skills saw for.

I: - In your community who decides at what age should people marry? What are the reasons for a women getting married at younger or older ages?

- Please tell us about your own marriage experience? How old were you when you marry? What are the reasons you she get married at that age?
- Please tell me how your age at marriage affects your PP family planning services use

R; - hahaha… now day marriage process is start with and ends with mobile communication… without family involvement in the process. I don't know about the others but when I married my wife she was 20 years old. I don't have detailed information may be 20 and above…

I: - What are your suggestions to improve women's roles and participation to enhance the use of PP family planning services?

R: - as I told before to improve women's participation… you have to be aware of the people surrounding her like their husband, mother, and family member because they have an influence on her decision. Women have to be supported by other community members.

Knowledge, Beliefs, and Perceptions

Now we are going to discuss about the issues faced by adolescents and women in accessing PP and PA FP services in your opinion.

I: - In your community what is appropriate behavior for recently delivered woman regarding use of PP and PA family planning services? How do they differ from adolescent girls and young women?

a. What is the appropriate behavior for a recently delivered mother? How do these believe influence health behavior including the PP family planning use?

R: - health workers consult the mother before leaving the health center after delivery as I was seen by my wife… so at that time she received family planning. In our woreda, people volunteer to receive contraceptives and also as woreda we are models and got many awards.

I: - What are the social beliefs and perceptions that share women's expectations and aspiration for PP family planning?

R; -

I: - In your opinion who should make decisions regarding use of PP family planning services? What types of decisions do women make regarding use of healthcare including PP family planning services? What types of decisions regarding use of healthcare including PP family planning services are made jointly?

R: - even I don't know about the others if you take us or my family this is the first birth for us… after she was delivered we discussed to gather when we have to have other baby and she took contraceptive method. So, discussion among partners is important in my opinion.

Legal Rights and Status

Now we are going to discuss about the marriage related process prevalent in this community.

I: - In your community are women who delivered recently legally allowed to receive PP family planning service?

- Is these same for boys and girls, people from different age groups, education background, ethnic groups, socio economic classes, excluded groups? How are they different? Why/ Why not?

R: - If it is not legal, how do health facilities deliver this (PP/PA family planning) for mothers… it is legal.

I: - How do recently delivered women access PP family planning information and resource from the healthcare facilities?

- Is these same for boys and girls, people from different age groups, education background, ethnic groups, socio economic classes, excluded groups? How are they different? Why/ Why not?
- Do the people in your community support recently delivered women to access PP family planning infatuation and resources? How do they provide support? Who are these people? Why do they provide support?
- Are there certain groups in your community who do not support this? Who are these people? Why don't they support?

R: - since I am a government employee, I know that family planning service was provided here at the health center… but in the case of other communities as I told you before there are community health workers among the community so they disseminate information about health-related issues including family planning for community members. They disseminate information going house to house or during community forums and meetings.

Yeah, there is individuals and group who are supporting women in this service … among the community, there are selected individuals leading 1 to 5 team so the team leader helps individual under their supervision to come and use the service in the health center. There is also a youth team here at Bedesa health center that also supports young women at school when they need any reproductive health-related support.

I: - Please tell me your experience with accessing healthcare information and resources including PP family planning.

- Do you feel you have a right to get healthcare information and resources including PP family planning services? Why/why not?
- Do you think your experience is different from other people in your community? How? Please tell me more
- Is there legal mechanism you can follow to ensure you get quality healthcare information and resources your need? Please tell me more

R: - I know that have the right to ask and get information about any health-related issue here at the health center. And also, a person who is providing service for the client has also to provide the required information appropriately for his/her client. I expect health professionals give correct information to their clients…. I am a red cross ambulance driver and I have the responsibility to bring patients to the health center as that is my responsibility and have to accomplish it on time every professional has to respond accordingly.

With this we have come to an end of the discussion. Do you want to add anything before we end the discussion or if do you have any questions you want to ask?

Thank you for your time and in case you want, you can get in touch with us through the details mentioned in the consent form.

****

Summary

This DII was conducted on 27/07/22. before going into the interview, we explain the objective of the study. And he was very interested in the issue but he explained that he cannot give detailed information since he has no information about FP methods. So, we explain that there is no right and wrong answer and just we need the idea to intervene accordingly. Generally, the interview was very interesting and he tried his best to explain what he knows about the issue raised in the interview guide.

Discussion

Rapport Building

Thank you for proving consent to participate in the discussion. There are no right or wrong answers, please share your frank opinion, as it will help us in understanding the situation better.

Enquire about her recent delivery experience. An example is given below:

Please tell us about family planning service in your woreda. Where did women get family planning services? Who provided the services? How far is the health facility from women's home (in different directions? How do women travel to the health facility?

Assets and Resources

Discussion

Now we are going to discuss about the Healthcare resources and assets available for provision of PP and PA family planning services in your area.

1. Health services (PP and PA family planning services) availability: Can you tell me the types of healthcare facilities that provide PP and PA family planning services in your area?
   a. What types of FP services are available at the healthcare facilities?
   b. Are these services equally available to boys, girls, young, old, married, unmarried, people from different geographic locations, religious or cultural background, language etc.? Please tell me more

Postpartum and post abortion family planning services are being given in our woreda even though there is some gap and unsatisfactory. There are different reasons for that. The trained professionals are not well delivering as expected. The facilities providing PP and PA are 6 health centers. The seventh one is a catholic church owned health center and they are not providing the service. The other six provide the service. Recently three health centers took the training and delivering the service well. The other three are not providing a good PP and PA FP service.

Regarding the accessibility of PP and PA family planning I would say it is not fully accessible. Through our discussion with the community and health care providers we have learned that there is problem related with accessibility. The reasons include lack of knowledge and misconception regarding the service. The community is also not well aware of where the serices are being provided. Health care providers have also problems to address access gaps to the grass root level. So, there are gaps from the community and from the health care providers. Few providers took the training, and they also transfer to other facilities. There is cultural influence regarding young boys and girls in our woreda. Especially recently married couples are not advised to take family planning immediately. There are no religious and language barrier in our woreda regarding PP and Pa family planning utilization.

2. Accessibility of the PP and PA family planning services
   a. Can you tell me how far the PP and PA family planning services providing facilities are located? How long does it take for women to access the facilities?
   b. What type of transportation means do they use to reach the facilities? How easy or difficult is it to use the transportation services?
   c. Is there any cost associated with accessing the PP and PA family planning services? Please tell me more about this

d. Is the PP and PA family planning service is accessible at all time at the healthcare facilities? Are there types of FP methods of users' choice in the healthcare facilities?
e. Are these services equally accessible to boys, girls, young, old, married, unmarried, people from different geographic locations, religious or cultural background, language etc.? Please tell me more
f. How easy could they access the PP and PA family planning services? Do you think that their access to the PP and PA family planning services are affected because of their background? How?

Concerning accessibility, there is one kebele in Buge catchment area called Hade Burquto and Harto Qonxola. The trained providers went there by motorcycle last time. it is relatively far and somehow challenging to address there. there is also language barrier in that kebele. Most of the people there speak Hadiya language. The transportation they use is motorcycle for those who can afford that and others have to walk. The transportation ost would take about 60 birr that is burden for some of the community members. There is motorcycle already accessible everywhere. There is no service cost associated with PP and PA family planning. The supplies are provided by Engender health and the government. Now a days there is shortage of supplies and we requested the Engender health and gov't officials. The service is available when there is trained professional in the facility. When they are off due to shifting, there is gap in service provision. There is shortage of trained providers in each facility. There is also shortage of supplies sometimes. So, when such things happen there is gap in accessibility. Young boys and girls are being trained and using the service around Gacheno. Other areas are starting such training and there some education being given for them. Cultural and religious influence for young boys and girls exist. The church is also not accepting discussions related with family planning for young unmarried boys and girls. The family is also not happy. We are providing education for the family and it is slow and there is a big gap in that area.

3. Means to access the PP and PA family planning services
a. Please tell me about your facilities' resources and assets. Please tell me the resources and assets belong to these health facilities to provide PP and PA FP service.
b. How is the use of the facility/Woreda health office resources and assets decided? Who makes the decision? Do you involve in the decision making? How do you think that affects access to the healthcare services including PP and PA family planning by women?
c. Please tell me about income of in this Woreda. Please tell me about your woredas income.
d. How is the use of the woreda's income decided? Who makes the decision? Do you involve in the decision making? How do you think that affects their access to the healthcare services including PP and PA family planning?
e. In your woreda how is use of PP and PA family planning seen? Is it acceptable for women to use PP and PA family planning? Why/why not? Please tell me more (what about boys and girls)

Regarding resources such as rooms there is shortage of facilities in our Woreda. Een during the construction, there is some gap in addressing PP and PA family planning. So, we are using the existing rooms. There is delivery coach in every facility. There is shortage of supplies sometimes for PP and PA family planning service provision. Training is also one challenge. After they get trained they transfer to other facilities. regarding PP and PA family planning supplies are donated. Both the woreda and zonal governments are not allocating budget for that. However, health centers present their requests to the woreda health office. After compiling all the requests we present to the zonal health department. I participate in the decision making process that take place in the form of meetings and evaluations. There are quarterly, biannual, and annual evaluation regarding service provision. So, I participate in decision making in through these ways. We present what we did and also present what is missing in the entire woreda. The income of this woreda can be categorized in to three. There are highlands, midland,

and lowlands in our areas. The highland and midland areas are better compared to lowland areas. The lowlands are frequently exposed to drought and shorage of food. The midland and highland produce crops such as teff, sorghum, beans, peas, and corn. They are relatively better. The lowlands living condition is worse. Decision regarding the use of resources is decided by the woreda management and the chief administrator. Health sector involve in planning by sending a base plan, but it is only on paper and no body applies based on that. For example about 15% of the budget is supposed to be spent on health, but this is not being practiced. Health office heads are participated in the budget planning, but application is poor. In the last two years we don't have even budget to pay for salaries and other basic expenses. They cut about 8 million birr for the debt of fertilizer and it is posing a big challenge for us. The woreda is not paying the full salary as well. Shortage of budget directly and indirectly affect our monitoring and supervision activities that in turn affects the PP and PA family planning service provision. We even couldn't buy fuell for the vehicles to go supervision. We can't meet with mothers in mothers conference due to shortage of fuel.

Regarding acceptability when we teach on mothers conference they are willing to receive after delivery of baby. There is improved awareness nowadays. For example some mothers who gave about four birth underwent tubal ligation some time ago. So, there is a good acceptance from the community.

4. In your opinion what should be done to improve access to the PP and PA family planning services for all women in your community?

As woreda to improve access to PP and PA family planning we should improve awareness creation during pregnancy. If we assign strong professionals there we can achieve that. Improving access to the far kebels should be addressed by going there to provide the service. Generally improving awerness and resources would increase access to PP and PA family planning.

Practices, Roles, and Participation

Now we are going to discuss about your role and responsibilities.

1. Please describe to me the typical Woreda health office officials/experts roles in your Woreda/community?
   a. How is Woreda health office officials occupied over the course of the day? Are there seasonal differences (campaign/political assignment) in how you use time?

As a department we have many roles. We work on maternal, child, newborn, adolescent health and nutrition. We work with mothers, children and adolescents including family planning. As I have told you we plan according to the total population we serve. We supply the required materials in each health center according to the plan including PP and PA family planning. We facilitate training for those who didn't receive training. We then take report of the activities in each month. We take reports and evaluate them for future action. When there is campaign there is more burden for health extension workers. When there is campaign HEWs will not be able to gather those who need PP and PA services to the health center. We will not have much burden when there is campaign. When we have campaigns we try to compensate the work left undone. At the health center level when there is campaign women may not get health care providers supposed to provide the family planning on the appointed date.

2. As Woreda health office official, what kind of work do you do? Do these works different when facility status changes (Rural, urban and geographical locations)?

3. Do Woreda health office officials like you have restrictions/barriers on the accessibility? What restrictions/barriers? How do the restrictions/barriers influence the access of the PP and PA family planning services and supportive social networks?
    a. In your Woreda do the restriction/barriers change by social characteristic, such as (dis)ability, economic status, health status, educational level, religion, culture, geographical location, and marital status?

At the catholic health center, family planning is not provided in that specific facility. But at the health post women get the services by the level two HEWs at the health post. They provide jaddle, implanon, depo and pills as well. The service is not provided at that specific compound only. Besides, the services is given equally for all population equally including those with disabilities and anyone who want to receive the service. There is no discrimination regarding the service provision.

4. Do you participate in any meetings, workshops, trainings, pregnant women conference, family, community, government, or other social networks organizations? Please tell me more.
    a. Please tell me how your participation in these organizations affect the PP and PA family planning services provision

    Yes I do participate in meetings, workshops and training including pregnant women conference. It has helped a lot to improve access to PP and PA family planning. The training I took at Shashemene changed my knowledge regarding the type of family planning women chooses while being on coach after abortion or delivery. I learned she can take any kind of family planning after delivery either pills, IUCD, implanon. So, I understood that after participating in that training. I learned also that it would be better if we find women before they give birth. Another thing is about the use of community conversation understanding. There are women who want the service but don't know from whom to receive the service. I learned that there is also a need to improve men's knowledge and involvement regarding PP and PA family planning.

5. In your Woreda do women assume leadership roles? What types of roles? Do these roles change by social characteristics, such as (dis)ability, economic status, health status, educational level, religion, culture, geographical location, and marital status?
    a. Please tell us about your own experience with assuming any leadership roles?
    b. Please tell me how your leadership roles affect the PP and PA family planning services provision

For example in our Woreda women assume different leadership roles. For example the woreda speaker and deputy are both women. there are also women in different sectors who assumed leadership role. Buge health center head is also a female. There are women in different areas in leadership position in every sector. There are also women leading at kebele and health development army as well. The role of women leadership affect PP and PA family planning. For example women's federation, healath development army help in accessing family planning. We also work in close collaboration with women and children office to improve access to PP and PA family planning. It is good not only for family planning provision, but also for every activity we do as a sector.

6. In your Woreda/community who decides at what age people marry? What are the reasons for getting married at younger or older ages in this Woreda?

Nowadays people decide by themselves. It is only later the family become aware of their decision. Sometimes young boys and girls marry before 18 years of age. At that age they are not well aware of

marriage. They don't know the advantages and disadvantages of marriage clearly. When they marry early they have resource to manage their house and the husband migrates to others area in search of job after sending her to her family. There are such cases in our community.

   a. Please tell me how their age at marriage affects their PP and PA family planning services use

7. What are your suggestions to improve women's roles and participation to enhance the use of PP and PA family planning services?

To improve women's role and participation concerning PP and PA family planning, we have to create awareness in each kebele and encourage them to give birth at the health facilities. If we encourage them well, they will share the information with other women who need the service.

Knowledge, Beliefs, and Perceptions

Now we are going to discuss about the issues faced by women in accessing PP and PA family Planning services in your opinion.

1. In your Woreda what is appropriate behavior for a woman after delivery and abortion regarding the use of PP and PA family planning? How do they differ from adolescent girls and young women?
   a. How do these believe influence health behavior including the PP and PA family planning use?

In our community there is no awareness regarding PA family planning after a woman faces abortion. What is important for them is to become pregnant after a while. They have no awareness that there is PA family planning service. Much is also not done regarding PA FP. There is awareness regarding PP FP and some women use, but there are still some gaps in some localities. The community perception regarding family planning is improving from time to time. Nowadays young boys and girls don't want too much children and they want not more than three or four children.

2. What are the social beliefs and perceptions that share women's expectations and aspiration for health care including PP and PA family planning?
3. In your opinion who should make decisions regarding use of PP and PA family planning services? What types of decisions do women make regarding use of healthcare including PP and PA family planning services? What types of decisions regarding use of healthcare including PP and PA family planning services are made jointly?

The decision should be made jointly. We are planning to participate males on women's conference in order to increase the uptake of PP and PA family planning.

Legal Rights and Status

Now we are going to discuss about the marriage related process prevalent in this community.

1. In your community are women who delivered recently legally allowed to receive PP and PA family planning service?
   - Is these same for boys and girls, people from different age groups, education background, ethnic groups, socio economic classes, excluded groups? How are they different? Why/ Why not?

They are legally allowed to receive PP and PA family planning irrespective of any background and all is allowed to use it including young boys and girls. There is no legal barrier for PP and PA family planning.

2. How do recently delivered/aborted women access PP and PA family planning information and resource from the healthcare facilities?
   - Is these same for boys and girls, people from different age groups, education background, ethnic groups, socio economic classes, excluded groups? How are they different? Why/ Why not?
   - Do the people in your community support recently delivered/aborted women to access PP and PA family planning information and resources? How do they provide support? Who are these people? Why do they provide support?
   - Are there certain groups in your community who do not support this? Who are these people? Why don't they support?

The recently delivered woman access PP and PA family planning form the mothers conference and from the health care providers in the kebele. currently young boys and girls access the information and use the service more than the older ones even. There are no groups in the community working against family planning.

3. Please tell me women's experience with accessing healthcare information and resources including PP family planning.
   - Do you feel they have a right to get healthcare information and resources including PP family planning services? Why/why not?
   - Do you think women's experience is different from other people in your community? How? Please tell me more
   - Is there legal mechanism you can follow to ensure they get quality healthcare information and resources they need? Please tell me more

Women have the right to get information regarding PP and PA family planning. There is a weekly command post where every activity is reported to the woreda health office. We get reports and check the amount of PP and PA family planning delivered per week. Through weekly reports and communications we check whether women are getting the information and service regarding family planning. We have women development army and 1 to 5 team where women share the information. There is also pregnant women conference where they share the information.

With this we have come to an end of the discussion. Do you want to add anything before we end the discussion or if you have any questions you want to ask?

Thank you for your time and in case you want, you can get in touch with us through the details mentioned in the consent form.

****

Interview details

**Interviewee**	Recently deliverd woman
**Age**	19
**Interviewee code**	IDI_BU_WO_290722
**Name of interviewer**	Beimnet Desalegn
**Date**	29/07/22
**Time started**	6:07 lt
**Time finished**	6;42 lt
**Venue**	Buge Health center

Assets and Resources

Discussion

Now we are going to discuss about the Healthcare resources and assets available to recently delivered mothers in your area.

I: - Health services (PP and PA family planning services) availability: Can you tell me the types of healthcare facilities that provide PP family planning services in your area?

a. What types of FP services are available at the healthcare facilities?
b. Are these services equally available to boys, girls, young, old, married, unmarried, people from different geographic locations, religious or cultural background, language etc.? Please tell me more

R: *- I don't know about family planning which is given immediately during postpartum and post-abortion.… but I know about a contraceptive that health centers provide to us when we need it at any time*.

I: - ok, you know the family planning which is given in health centers generally ... how about contraceptives that health workers give to mother immediately after giving birth? Have you ever heard about it?

R: *- I haven't heard about it, it is new for me. This is my first birth for me and I have no experience in using contraceptive methods.*

Ok, … I know contraceptives which users take for three months, and the other given three year. I know this two well… but I don't have experience of using it just I have information about them. I haven't used contraceptive methods … I have plan to use it in the future.

Yeah … health workers here are serving the community in good manner, they are so disciplined… I have heard from my colleagues that they respect their client… I had come with my husband's mom and I saw they are serving respectfully.

2. Accessibility of the PP family planning services

a. Can you tell me how far the PP family planning services providing facility is located? How long does it take for you to access the facility?
b. What type of transportation means do you use to reach the facility? How easy or difficult is it to use the transportation services?
c. Is there any cost associated with accessing the PP family planning services? Please tell me more about this
d. Is the PP family planning service open at all time and is accessible with short waiting time at the healthcare facility? Are there types of FP methods of users' choice in the healthcare facility? Do healthcare providers receive and treat users with respect and dignity?
e. Are these services equally accessible to boys, girls, young, old, married, unmarried, people from different geographic locations, religious or cultural background, language etc.? Please tell me more
f. How easy could you access the PP family planning service? Do you think your access to the PP family planning services is affected because of your background? How?

***R: -** it's not far for me to come to here … it takes about five minutes. And I came here now with my feet.*

*As I know, people who has **matama** account use service without payments.*

Ok… regarding maternal service like delivery and family planning service are free of charge.

Yeah… health center here is providing 24-hour services … and we get service we need at any time when we come to health center. I can tell you about day time service … I came for vaccination as well as for other health care with patient … we get them on work place while serving clients.

Sometimes they prescribe drugs to buy from privet pharmacy if they don't have at the health center. If they have they give us from their pharmacy.

Yeah, … I was followed ANC care and now vaccinating my baby here … during my ANC follow up as well as now in vaccination time as I see they are so humble and respectful. I was given birth here at the time they treat me in good manner.

Sometimes the absence of drug is the problem here … no it is not for family planning it's in other services.

I: - Means to access the PP family planning services

g. Please tell me about your household resources and assets. Please tell me the resources and assets belong to you.
h. How is the use of the household resources and assets decided? Who makes the decision? Do you involve in the decision making? How do you think that affects your access to the healthcare services including PP family planning?
i. Please tell me about your household income. Please tell me about your own income.
j. How is the use of the household income decided? Who makes the decision? Do you involve in the decision making? How do you think that affects your access to the healthcare services including PP and PA family planning?
k. In your community how is use of PP family planning seen? Is it acceptable for women to use PP family planning? Why/why not? Please tell me more

R: - *We are living in the rental house and …. eee he is merchant, selling second hand clothes (bonda clothes) in local area market. I am student and not yet involved in any work.*

He plans together, … We will customize the monthly allowance … and put for other required things … we plan for a month. We don't put money for health-related issue alone, we put money in bank and we use from bank when we need money.

We always discus together to use money when one of us need money…

In my opinion using contraceptive methods immediately after having birth before returning to home is good for us… I haven't taken it because I have no information at that time. Ok... at that time I have no information no one has told me … I give birth here but I have no information about postpartum family planning services. If I know I took at that time…

Many women have good attitude for contraception… because for those who want to delay their birth it is very important. There are many people who cannot afford sufficient think for their family those people can use and manage their family size. Those people are very happy …

I: - In your opinion what should be done to improve access to the PP family planning services for all recently delivered mothers in your community?

R: - to improve this service … the health center should have to avail family planning method for users and have to inform the mother about the service. There are many women who were getting pregnant due to the absence of drug they chose to use like depo. Most of the time I hear that they complaining the absence of depo by women. And sometime women have no information about specific information like

immediate postpartum family planning so they have to inform them very well and have to offer service for them at the time. Those women who know about the service may aske and use … but mothers like me need to be informed. Otherwise we go without getting service. So, this is the responsibility of the health workers…

Practices, Roles, and Participation

Now we are going to discuss about your women's and your role and responsibilities.

I: - Please describe to me the typical women's roles in your community?

- How are women and girls occupied over the course of 24 hours? Are there seasonal differences in how women and girls use their time?

R: - *hahaa… I don't have work, I am student… can I consider caring my baby as my work? Hahaha. If so, I care for my child … I clean, feed, and take care of my baby … also prefers food for my family. Most of the time I care for my baby… because as this birth is the first I am living with my family. But when I turn to my home I will do all the home work … cocking, washing, caring for my baby and family this all is a woman's responsibility.*

I: - What kind of work do you do? Do these works change when your age or status changes (young, unmarried and married, older)?

R: - Yeah, role of women may change as the age of women increase … or their experience or educational level increase… if I got additional job it is obeys workload is increasing. That woman having office work has also responsibility of caring their family … they may spend working time in office and again when they return home … they may cock, clean house and care for their family.

As I told you was completed preparatory school last year before getting marry … now I have plane to continue my study at collage.

I: - Do you have barriers on your access? What barriers? How do the barriers/restrictions influence your access to the PP family planning services and supportive social networks?

- In your community do the restrictions/barriers change by social characteristic, such as (dis)ability, economic status, health status, educational level, religion, culture, geographical location, and marital status?

R: - I don't have any barrier, I told you that I have no experience of using contraceptive methods. After marriage we planned to give birth so I did not use contraceptive methods, but now I was taking immediately after birth if I had information.

I haven't seen any restrictions, most of our community member support contraceptive use by married women but for those who did not married … using contraceptive is difficult because community perception is not good for them if they use contraceptive methods. …. Eee they talk behind if they use before marriage. Using contraceptive method before marriage is not supported by many so that my strict youth unmarried contraceptive users.

I: - Do you participate in any family, community, government, or other social networks organizations? Please tell me more.

- Please tell me how your participation in these organizations affect your PP family planning services use

R: - I don't have experience of participating in meeting which is either governmental or non-governmental … but I perceive that I have to space my birth… till this child get into school … minimum of four to six years. I wouldn't want to give birth over and over again. … I thing like this.

I: - In your community do women assume leadership roles? What types of roles? Do these roles change by social characteristics, such as (dis)ability, economic status, health status, educational level, religion, culture, geographical location, and marital status?

- Please tell us about your own experience with assuming any leadership roles?
- Please tell me how your leadership roles affect your PP family planning services use

***R:** - I think women can assume different position … even they can be a doctor… if they work hard. The only thing is being hard worker to assume any role or position. I see women in different position… they could be leader in different organization.*

As I told you I am now student … and I did not assume any leadership position. So, I have plane to continue my study and plane to take leadership in the future.

I: - In your community who decides at what age should people marry? What are the reasons for a woman getting married at younger or older ages?

- Please tell us about your own marriage experience? How old were you when you marry? What are the reasons you she get married at that age?
- Please tell me how your age at marriage affects your PP family planning services use

R: - *yeah…. Haha. Now there is phone for every one so they talk woman talk to the man and …. most of the time decided by themselves to marry. Previously family decides whom should marry and when but now it is changed. Yea, most of the time 18 years and above … but they may talk to man since when they are 14 years old because they got phone access at that age.*

I was married at 19 years old… I was not ready while I marry it was accidental to me at the time … I am talking to him but I didn't have plan to marry at that time.

Most of the time I heard that using contraceptives method at youth age is leading to some conditions like infertility… this is how the community perceive. Also using contraceptive before having first birth has also leads to infertility … I hear this thing from women.

I:- What are your suggestions to improve women's roles and participation to enhance the use of PP family planning services?

R: - health workers should council the client with appropriate information about each drug. If the women understand each drug behavior … the miss conception I raised above will be corrected and contraception use in postpartum immediately my improved. Also concerned body should have to give education at community level for women.

<u>Knowledge, Beliefs, and Perceptions</u>

Now we are going to discuss about the issues faced by adolescents and women in accessing PP and PA FP services in your opinion.

I: - In your community what is appropriate behavior for recently delivered woman regarding use of PP and PA family planning services? How do they differ from adolescent girls and young women?

a. What is the appropriate behavior for a recently delivered mother? How do these believe influence health behavior including the PP family planning use?

R: - *most of the community member take it as appropriate action … no one is against it if they got information about the service. This is in my view …*

I: - What are the social beliefs and perceptions that share women's expectations and aspiration for PP family planning?

R: - *no I don't think any ….*

I: - In your opinion who should make decisions regarding use of PP family planning services? What types of decisions do women make regarding use of healthcare including PP family planning services? What types of decisions regarding use of healthcare including PP family planning services are made jointly?

R: - *in my view I have to decide for myself because I know my self more then other … also my problem so I have to decide for myself when to use and what to use by myself. And in addition, health worker should have to help us.*

Legal Rights and Status

Now we are going to discuss about the marriage related process prevalent in this community.

I: - In your community are women who delivered recently legally allowed to receive PP family planning service?

- Is these same for boys and girls, people from different age groups, education background, ethnic groups, socio economic classes, excluded groups? How are they different? Why/ Why not?

R: - *yes, it is allowed legally … I thing that is why health center providing services here. No …*

I: - How do recently delivered women access PP family planning information and resource from the healthcare facilities?

- Is these same for boys and girls, people from different age groups, education background, ethnic groups, socio economic classes, excluded groups? How are they different? Why/ Why not?
- Do the people in your community support recently delivered women to access PP family planning infatuation and resources? How do they provide support? Who are these people? Why do they provide support?
- Are there certain groups in your community who do not support this? Who are these people? Why don't they support?

R: - *most of the time from health workers … there is community health extension workers providing contraceptive menthods information for the community. Additionally, from mass medias radio and TV.*

Yeah there are group of women who are supporting and educating community with health extension workers. They disseminate information about contraceptive method and take those who need the service to the health post. No … there is no groups who is preventing the use of contraceptive methods.

I: - Please tell me your experience with accessing healthcare information and resources including PP family planning.

- Do you feel you have a right to get healthcare information and resources including PP family planning services? Why/why not?
- Do you think your experience is different from other people in your community? How? Please tell me more
- Is there legal mechanism you can follow to ensure you get quality healthcare information and resources your need? Please tell me more

R: - *yeah, that is my right to take my chose and getting full information about my chose. The health worker has also obligation to give information about the drug either it is a side effect of the drug or advantage.*

Yeah, we are getting information from different sources, to check the quality or truthiness of the information the option we have is asking the health workers. I will ask health workers at the health center.

With this we have come to an end of the discussion. Do you want to add anything before we end the discussion or if do you have any questions you want to ask?

Thank you for your time and in case you want, you can get in touch with us through the details mentioned in the consent form.

****

Summary:

The interview was conducted on 29/07/22 at Buge health center. She comes to the health center to vaccinate her 45-day baby. and she was waiting for health workers in the immunization room while we met her. The interview was interesting but she did not have exposure to the FP method before. So her response was very shallow and so, we tried our best to explore her idea.

In-Depth Interview conducted with head of Delbo health center by Degnesh Dawit

Socidemographic information of interviewee

Code	Keble	age	sex	Educational-status	Job responsibility	Marriage-status	Work experience
IDI_DHC_HCH_300622	Delbo	35	M	BSC nurse	Health center head	Married	10 years

Assets and Resources

Discussion

I; Health services (PP and PA family planning services) availability: Can you tell me the types of healthcare facilities that provide PP and PA family planning services in your area?

R; before training regarding PP and PA FP has been given, our performance regarding PP and PA FP during monthly PRT evalution was too least, the activities are also not started so far. However after training given to professionals its performance was increased and the activities started as a new program

I; What types of FP services are available at the healthcare facilities?

R; before coming to health center we have gone to community and collected all pregnant mothers with their husband at kebele lable and gave them health education regarding ANC follow up and PP and PA , after that when they come to health center for ANC visit cousel them to receive PP FP after delivery. When we come to family planning related with post abortion those who come to receive abortion service counselling them to receive family planning services to stay atleast six months before occurance of next pregnancy. When we looking the methods of family planning given after postpartum period or postabortion period, previously we focus on only IUCD, we encourage mothers to receive IUCD within fourty eight hour of delivery, but currently after training provided all types of long acting family planning is possible based on their choice. Now in our health center we have all types services available like IUCD, Jadelle,

Implanon, Dipo,pills, postpills, condom for males. For the last three or four consecutive months we lack Dipo, but currently the organization started to provide it so now we have Dipo also.

I; Are these services equally available to boys, girls, young, old, married, unmarried, people from different geographic locations, religious or cultural background, language etc.? Please tell me more

R; yes all services are equally available for people from different sociodemographic background as their interest, we have all types of services available including condom for males, IuCD, Implanon , Jadelle and Postpills, pills and Dipo. We put condom in mini boxes in different places of health cervice compound for those who needs it but there is no trained professional in our health center to provide the permanent (vasectomy) family planning method for males in our health center service

1. Accessibility of the PP and PA family planning services

I; Can you tell me how far the PP and PA family planning services providing facilities are located? How long does it take for women to access the facilities?

R*; the two catchment area the so called Xando and Damota are far to this health center, for them to come to this health center for example for people resided in Xando it takes around one hour and 25-30 mins through walking and motorcycle respectively, for Damota it takes around two hours through walking.*

I; What type of transportation means do they use to reach the facilities?

R; those who resided in Xando use motorcycle as means of transportation, or those who come from Damota come through walking because motorcycle is unable to go through there due to difficult topography or road, to get a motorcycle they should come to down concrete road from mountains region. To the services related with family planning they can get it from health extension workers, but for the seck of immidieate family planning they have to come to health center for delivery service and at that time they can receive the service. Health extension worker cannot provide the delivery service because there is no fulfilled materials in health post. But health extension workers can provide family planning services for those who don't received it immediately after delivery and want to receive it within postpartum period.

I; How easy or difficult is it to use the transportation services?

R; currenly getting motorcycle is not much difficult for Xando residents, but for people resided in Damota it is impossible to use transportation before reaching to the concrete road.

I; Is there any cost associated with accessing the PP and PA family planning services? Please tell me more about this

R; in the health center level the service is being provided freely, currently we are suffering with costs related with family planning as health center previously several organizations supports this service but now there is no support from anywhere, eventhough the government given priority position for this services still now and continued the service provision freely.

I; Is the PP and PA family planning service is accessible at all time at the healthcare facilities? Are there types of FP methods of users' choice in the healthcare facilities?

R; as a standard a family planning provision center should be open and ready to provide the service related with this, since it is the governmental organization it is mandatory to provide the service required at any time, and our health center also acting accordingly. When one client come to our health center to receive family planning service the service is based on informed choice, frist she has been informed on the available methods and counselled over it, after counselling and convicing her she choose in which to receive it. Specifically we lacked Dipo for the last four or five consecutive days the organization the so called EPSA which provides Dipo for healthcenters and it stopped providing for the last four months but currently it started providing of it so it is also available in our health center. Most of woman in this community have more interest on dipo and most of them uses it, so when we lack it it created us great opportunity to convince them to use long acting family planning methods. Otherwise we have all types as Delbo health center and people can choose and use it.

I; Are these services equally accessible to boys, girls, young, old, married, unmarried, people from different geographic locations, religious or cultural background, language etc.? Please tell me more

R; yes it is equally accessible for all regardless of their language, age, marriage status, economic status etc. Those mothers who are within reproductive age group can get services in wich they need. Those girls aged greater than eighteen come to our health center to get abortion care service can get family planning services at the same time. All people can get the

services regardless of their difference, even if males need the service we will give to them after counselling them.

I; How easy could they access the PP and PA family planning services? Do you think that their access to the PP and PA family planning services are affected because of their background? How?

R; postpartum or post abortion family planning is some what new for us and its not that much practiced so far before,its not that much easy to provide the service because since it is new it needs awareness creation among the community so far, even after counselling several times they may refuse to receive the service we can not enforce them, but some of them agrees to receive the service we give the service for those who decides to receive the service. Since we are on the way of implementing the activities awareness creation and counselling them exauslively until we get the result we expect to achieve. The service is provided equally beyond their background because it is the matter of right.

2. Means to access the PP and PA family planning services

I; Please tell me about your facilities' resources and assets.

R; facilities assets and resources are, building, professionals, tables, chairs, beds, drugs, syringes, gloves delivery sets, sterilizers, delivery cotches, family planning drugs etc

I; Please tell me the resources and assets belong to these health facilities to provide PP and PA FP service.

R;Rooms, trained professionals, tables, chairs, beds, drugs, syringes, gloves delivery sets, sterilizers, delivery cotches, family planning drugs, registration books etc

I; How is the use of the facility/Woreda health office resources and assets decided? Who makes the decision? Do you involve in the decision making??

R; The resources and assets of our health center decided based on the plan of health center as health center we have monthly planning, quarterly planning and yearly planning for example our yearly delivery service plan is 523, from that our monthly plan is 44, from that we may achieve atleast 40, regarding immediate family planning we planned around 250 for one year from that we may achive atleast 200, based on this plan the resources and assets allocated for MCH class. The decision making body regarding income assets and income were the health center health

care financing, health center head and MCH focal. Since I m one of decision maker every month as health center head I request EFSA to provide us family planning drugs as our plan others like gloves syringes we got it through bought from health center budjet.

I; How do you think that affects access to the healthcare services including PP and PA family planning by women

R; our involvement in both in planning and resource allocation helps to strength the post partum and post abortion family planning service because we can allocate the resources based on our plan.

I; Please tell me about income/economic capacity of in this health center . Please tell me about your health center income.

R; *our 2014 income I sabout 666000 Ethiopian birr. The source of income for this health center is our customer, that means indirectly we the source from the patients through community based insurance in addition to that priorily Engender health supports us but currently they stopped helping us, there is no that much support from government we get. We can get family planning drugs monthly in kind from nongovernmental organization.*

I; How is the use of the woreda's income decided? Who makes the decision? Do you involve in the decision making? How do you think that affects their access to the healthcare services including PP and PA family planning?

R; Most of this health center income decided through health care system of this health center. Almost 80% of income we got from CBHI will be for drug. There is board established to decide such kind of issue the members are Woreda admin person Woreda health office person the health center health care financing person, health center head and also we have standard which leads such kind of things. When we make meeting with Established board we orient our monthly plan in our every sector including PP and PA FP service. At that time they decide based on the existing situation, at that time we can debate if the provision is less than the required one. We also try to understand the esisting situation of our country. Then based on the decision we try to allocate based on the the requirement of the service, almost 50% of our total budjet is allocated for MCH services this is put as a manual or guidline but it is not in real world they think that other doors can provide for this service but there is nothing currently.

I; In your woreda how is use of PP and PA family planning seen? Is it acceptable for women to use PP and PA family planning? Why/why not? Please tell me more (what about boys and girls)

R; After short period of time means after the training the activities are widely started we evaluate the performance weekly from the weekly performance around sixteen mothers received postpartum family planning and four received post abortion family planning as our health center weekly report, currenly the utilization of the service among mothers are increased day to day, at the beginning it was very low and now it becomes increasing. *Regarding the acceptability of the program among women couselling given after delivery there are mothers who are receiving the service and similarily there is mothers who refuse to receive. Those who have got counselling from ANC to delivery and understood well about the service were deciding to receive it, and while those think that they cant get pregnant up to two or three years after giving birth by naturally without using pregnancy refuse to receive the service immidiaely after delivery.*

I; In your opinion what should be done to improve access to the PP and PA family planning services for all women in your community?

R; in my opinion the activities related with counselling should be done starting from community level by the high involvement of health extension workers to create awareness among pregnant mothers during pregnant mothers forum and home to home visit, the other one is counselling them during ANC visit exhaustively up to last visit in addition to that they should be counselled before latent period of labor and after delivery.

Practices, Roles, and Participation

I; Please describe to me the typical health center head roles in your community?

R; there role is allocating and availing resources as their need in specific department, like professionals instruments, monioring those instruments wether it is functions or not and evaluating the activity performed in each department weekly. Identifying the challenges and threats in the activity performance of each class and also providing the solution for those challenges as much as possible, identifying those professionals who have skill gap to do some activities and after identifying them arrange training for them based on specific gap by communicating with concerned body. Joining on the woreda or health center intended meetings.

I; How is Woreda health office officials occupied over the course of the day? Are there seasonal differences (campaign/political assignment) in how you use time?

R; day to day activities is not similar fore example in one week we may have campaign, keble visits to graduate model kebles, managing clients controlling overall activities of the health center by communicating with department heads and following what is available and not. For example when we have campaign and keble leaders and HEWs evaluation and keble graduation I become busy.

I; As health center head, what kind of work do you do? Do these works different when facility status changes (Rural, urban and geographical locations)?

R; my frist role is allocating and availing resources as their need in specific department, like professionals instruments, monioring those instruments wether it is functions or not and evaluating the activity performed in each department weekly. Identifying the challenges and threats in the activity performance of each class and also providing the solution for those challenges as much as possible, identifying those professionals who have skill gap to do some activities and after identifying them arrange training for them based on specific gap by communicating with concerned body. Joining on the woreda or health center intended meetings. All activities are done by according to standard or guide line so its similar in either urban or rural setting because in both of them the service utilizers are community, but the people acceptance of the service is not similar in both but the service provided is the same everywhere, despite the health center is located in urban area it includes both rural and urban catchment.

I; Do health center head like you have restrictions/barriers on the accessibility? What restrictions/barriers? How do the restrictions/barriers influence the access of the PP and PA family planning services and supportive social networks?

R; things can be barrier for us are resource related things especially those things we get through bought when we change it to money it needs a large money to fulfill the required money our health center currently facing scarcity of income.The other one is not all professionals trained on the PP and PA FP service provision so those who are not trained lacks skill and consent to do the activities. Other thing can be barrier as our health center is registration books previously we got it from engender health but now we completed it and we have no formal registration book. In addition to that health extension workers hasn't been taken formal training regarding

the service. This all barriers can affect the activities because if we have no goose, syringe or gloves we cant keep up the activity, and also if all professionals not trained for the service some mothers may miss the service those who got delivery service by those professionals. If health extension workers received the training they are near to tha community than us and they can orient and create awareness to them if it is not its difficult to us to provide the service by only health center level.

I; In your Woreda do the restriction/barriers change by social characteristic, such as (dis)ability, economic status, health status, educational level, religion, culture, geographical location, and marital status?

R; Those things you mentioned above does not change the barriers I mentioned you above .

I; Do you participate in any meetings, workshops, trainings, pregnant women conference, family, community, government, or other social networks organizations? Please tell me more.

R; yes I used to participate in different type of meetings, pregnant mothers conference, meetings arranged by endanger health, I were in Halaba when this research conference first launched and participated in different types of workships.

I; Please tell me how your participation in these organizations affect the PP and PA family planning services provision

R*; for one person to lead or to evaluate specific activities should have knowledge regarding it, being participated in those meetings and workships helped me to understand what should be done for mothers, what is required from professionals, what is required from me and this all things I m talking with you is the result of the participation because I have awareness regarding the service*.

I; In your health center do women assume leadership roles? What types of roles? Do these roles change by social characteristics, such as (dis)ability, economic status, health status, educational level, religion, culture, geographical location, and marital status?

R; females involvement in any organization is must this also encouraged by government from up to down, by considering this in our health center also brought women for leadership position for example in our health center, MCH focal, laboratory focal and emergency focal are women. As our health center the number of women are greater than the number of male, so without their

high involvement we cant achieve our goal. Assuming woman for leader ship role is depend up on their hardworking status because hardworker woman can be role model for others, our criteria to assign woman for leadership position is only hardworking because hardworkers can engage others and can be role model for them who are not working their work properly.

I; Please tell us about your own experience with assuming any leadership roles?

R; I came to leadership position based on my hardwork culture, educational status and work expreince.

I; Please tell me how your leadership roles affect the PP and PA family planning services provision

R; in my behavior I m happy by sharing knowledge/ information what I got from any training and workshops to professionals by arranging the training session on the postpartum and post abortion family planning.Personally I m sociable and interactive with staffs so they accept my concern so sharing information and working in coordination with them encourages them to perform their activities carefully.If we do such activities in coordination with all professionals it is easy to achieve our goal and convincing mothers are not become challenge to delivery service providers and for those who work in family planning.

I; In your community who decides at what age people marry? What are the reasons for getting married at younger or older ages in this Woreda?

R; previously such kind of issues were decided by family but, currently it is decided by those persons who are going to marry. The reasons for getting married are when someone thinks that he/ she was matured by mentally, physically, economically and able to lead family and have enough resource to live stable life. As health institution our health center teaches young boys and girls about sexual and reproductive health in the YFS center.

I; Please tell me how their age at marriage affects their PP and PA family planning services use

R; a person should get marriage at appropriate either early or late marriage is not recommended because both of them have their own health related consequences if they get marriage at earlier age it has several related complications specially for woman. To receive the postpartum family planning one person should be at reproductive age group. For example for

one fourty nine years old aged mother the service cannot be given because they are out of reproductive age like that for girls aged below eighteen receiving it is not recommended.

I; What are your suggestions to improve women's roles and participation to enhance the use of PP and PA family planning services?

R; womens participation is key enhance the use of PP and PA family planning, because females are close to each other so if one mother who previously received the service can share her experience to others. When you go to community one to thirty leaders are women health extension workers are females,most of professionals in MCH department is also females so engaging those stake holders in this activity and letting them to actively participate in counselling pregnant woman in their community and at health center can enhance the use of PP and PA FP services. For example when we go to community some of mothers suffering from children borne within short birth space without economic capability. Teanagers also started to practice sexual intercourse suffering from abortion done by traditional way, so the work should be start from community level to increase service accessability among child bearing age groups. Therefore working incollaboration with all stake holders should start in strong manner.

Knowledge, Beliefs, and Perceptions

I; In your Woreda what is appropriate behavior for a woman after delivery and abortion regarding the use of PP and PA family planning? How do they differ from adolescent girls and young women?

R; some of mothers decide to receive the service while others refuse to receive the service, specially those who give birth by stich feels that insertion of IUCD for them is difficult, because they feel pain and discomfort at that time, they may choose to receive implanon and Jadelle. For all activities to do better appropriate counselling is necessary.Those who refuse to receive the service were also due to lack of appropriate counselling. Adolecent girls can receive the service at any time based on informed choice.

I; How do these believe influence health behavior including the PP and PA family planning use?

R; Due to the pain and discomfort they feel after delivery they may refuse to receive the service.

I; What are the social beliefs and perceptions that share women's expectations and aspiration for health care including PP and PA family planning?

R; The program is new for our community it has not been widely used among the community before, so that when they get someone who received the service immediately after delivery they why don't you stayed until you become strong? What made you to receive this at this time? Are you want to do sexual intercourse within short period of time, this too fast etc. aTo avoid such kind of beleifs woman who received the service should convince them but if she don't convinced to receive the service she unable to do it.

I; In your opinion who should make decisions regarding use of PP and PA family planning services?

R; recently delivered mother herself and her husband should make decision together to receive or not to receive the service because no professional has the right to force her.

I; What types of decisions do women make regarding use of healthcare including PP and PA family planning services?

R; Most of the time the decision regarding use of healthcare including PP and PA family planning made by mothers herself or by discussion with her husband.

I; What types of decisions regarding use of healthcare including PP and PA family planning services are made jointly?

R; if they decide together or jointly they reach to decision to receive PP and PA family planning.

Legal Rights and Status

Now we are going to discuss about the marriage related process prevalent in this community.

I; In your community are women who delivered recently legally allowed to receive PP and PA family planning service?

R; if it is not legally allowed it cannot be approved and allowed to provide the service. So it is recognized by legal bodies also. Government cannot allow illegal thing for the community officially. Since it is important and legally approved several thing is expected from us as professional to excute it. The full service is based on the right of mother.

I; Is these same for boys and girls, people from different age groups, education background, ethnic groups, socio economic classes, excluded groups? How are they different? Why/ Why not?

R; It is the same for all people from different background but still now the service is not started for boys, due to lack of their interest.

I; How do recently delivered/aborted women access PP and PA family planning information and resource from the healthcare facilities?

R; she can get the information from the community as I told you every month there is pregnant mothers forum at that time midwives assigned to go to specific kebele and teach them, when she come to health center for ANC visit she can get counselling regarding the service and and also during delivery time again she can get counselling and receive family planning drugs immediately after delivery or within six weeks of delivery.

I; Is these same for boys and girls, people from different age groups, education background, ethnic groups, socio economic classes, excluded groups? How are they different? Why/ Why not?

R; It is the same for all people from different background but still now the service is not started for boys, due to lack of their interest.

I; Do the people in your community support recently delivered/aborted women to access PP and PA family planning information and resources? How do they provide support? Who are these people? Why do they provide support?

R; yes there is some women who have positive outlookings in the service and used it previously encourages others to receive it based on their experience, the other one is we planned to engage and oreinte one to thirty leaders about PP PA FP methods in the activity ike other package of HEWs and let them to involved in the activity by teaching pregnant mothers under their coordination. And also planned to engage religious leaders on the activity by creatingawareness among them because this service is somewhat new for all.

I; Are there certain groups in your community who do not support this? Who are these people? Why don't they support?

R; There may be kind of people those who opposes the activities, but personally I didn't find them, the resean behind is the idea is new for all that's why.

I; Please tell me women's experience with accessing healthcare information and resources including PP family planning.

R; they can get from massmedia, from pears, from the school, from the one to thirty leaders, from one to five leaders, from Health extension workers, from health center, from hospital, from health professional etc.

I; Do you feel they have a right to get healthcare information and resources including PP family planning services? Why/why not?

R; yes they have well deserved right to get either resource or information , no one can prevent them from getting it

I; Do you think women's experience is different from other people in your community? How? Please tell me more

R; women have special concern and access than other groups of people because most of people served by MCH classes are females because of their specific need

I; Is there legal mechanism you can follow to ensure they get quality healthcare information and resources they need? Please tell me more
R; A client has right to get the quality service and the health professional have obligation to provide the quality information and resource. If this not done accordingly and if one client informed to me or to complaint hearing committee established in our health center, we all committee members and me make meeting together and solve the problem.

Summary

This interview conducted with Health center head at Delbo health center in 30/06/22. He has 10 years work expreince and have sufficient knowledge on PP FP and PA FP.

Key findings were:- the service available are creating awareness starting from community level to latent phase of delivery and giving service after after convincing. Awareness creation should

be done at community level for both pregnant mothers and husband. Previously we focus on IUCD but currently all types of methods. The program is new for our community it has not been widely used among the community and people have misconceprion regarding it. People who received the service supports other to use it. The main challenges are, we face financial constraints to sustain the service, lack of registration book, HEWs not received formal training to be involved in program.

In-Depth Interview Guide with Recently delivered Mother Husband at Delbo health center by Deginesh Dawit

<u>Sociodemographic information of interviewee</u>

Code	Keble	age	sex	Educational-status	Job-status	Number of children
IDI_DHC_H_270722	Delbo	42	M	Diploma	Government employee	five

<u>Discussion</u>

<u>Assets and Resources</u>

<u>Discussion</u>

I; Health services (PP and PA family planning services) availability: Can you tell me the types of healthcare facilities that provide PP family planning services in your area?

R; family planning service given immediately after woman given birth is at the health center and hospitals

I; What types of FP services are available at the healthcare facilities?

R; types of family planning methods given immediately after birth are three month injection, also three years family planning methods given for voluntary mothers.

I; Are these services equally available to boys, girls, young, old, married, unmarried, people from different geographic locations, religious or cultural background, language etc.? Please tell me more

R; the family planning services should be given for young because most of the time they face unwanted pregnancy at that time they forced to abort in traditional way. The other one is

receiving family planning methods within 24 hour of delivery is very important. For females, if they come to receive the service they have all kinds of family planning methods as their need. For males, there is condom at health center but their wives are not voluntary to do sexual intercourse by condom, and also males are not voluntary to use it.

1. Accessibility of the PP family planning services

I; Can you tell me how far the PP family planning services providing facility is located? How long does it take for you to access the facility?

R; my home is not that far from this health center it takes around fifteen up to twenty minutes through walking

I; What type of transportation means do you use to reach the facility? How easy or difficult is it to use the transportation services?

R; we used to walk to come here due it is near to our home

I; Is there any cost associated with accessing the PP family planning services? Please tell me more about this

R; not only family planning services related to pregnancy has no associated cost, even sometimes they come to our home to give the information regarding family planning

I; Is the PP family planning service open at all time and is accessible with short waiting time at the healthcare facility? Are there types of FP methods of users' choice in the healthcare facility? Do healthcare providers receive and treat users with respect and dignity?

R; it is around thirteen years has been passed since this health center started to serve the community up to today the service provision classes are open twenty four hours and the health professionals are ready to serve the mothers come with requirement. They have no lunchtime, dinnertime they work by shift. When woman come to them first they tell about importance of drug and provide based on their interest without forcing them, and informs them to consult health professional if the received drug make discomfort for them. I think Health professionals especially who works at delivery and family planning service has changed mind and they treat people in respect with dignity and all of their services are given freely, all persons who works at health center starting from gate keeper gives special respect to mothers. They accepts mothers whatever time she comes either she comes at 4 o'clock, or 10 or at night or day time

I; Are these services equally accessible to boys, girls, young, old, married, unmarried, people from different geographic locations, religious or cultural background, language etc.? Please tell me more

R; all services are equally accessible for females they can use as their need but males don't voluntary to use condom it is not comfortable to them or for their wife and also they want to be productive at every age of their life so they fear to use permanent family planning method.

I; How easy could you access the PP family planning service? Do you think your access to the PP family planning services is affected because of your background? How?

R; one woman become pregnant for nine month then passes through difficult delivery after this all *challenges if they told to receive the services they accept it in happiness because it is for themselves to take rest before getting another pregnancy, their husband also asks them whether they received it or not for example I ask my wife whether she received the family planning method or not when she come to home after delivery. Because she don't know what will happen up to six weeks because she is living in her home with her husband, some of woman when they become pregnant before six weeks forced to abort in traditional way. For not to that kind of things they are happy to receive the services, if they told in detail about the service I think no one opposes it. I think Health professionals especially who works at delivery and family planning service has changed mind and they treat people in respect with dignity and all of their services are given freely, all persons who works at health center starting from gate keeper gives special respect to mothers*

2. Means to access the PP family planning services

I; Please tell me about your household resources and assets. Please tell me the resources and assets belong to you.

R; we have our own home, I have working instruments I am working as cameraman, we have cattle, and household utensils. All of resources we have are for both of us in common nothing is for one person alone.

I; how is the use of the household resources and assets decided? Who makes the decision? Do you involve in the decision-making? How do you think that affects your access to the healthcare services including PP family planning?

R*; we decide our household resources and assets after discussing together reaches to decision. Using family planning is very important to us because at this year growing children is challengful thing if they born without birth spacing it is difficult to buy clothes, teaching them, so it allows to born children, which matches with our capacity, now we stopped bearing children now. Our discussion in everything and reaching in agreement helped us to grow our children in better way by using family planning method.*

I; Please tell me about your household income. Please tell me about your own income.

R; I m government employee at the same time I'm merchant and also work as cameraman from those all I will get 5000-6000 Ethiopian birr monthly. My own income is the money I got from government but others are from both of us.

I; How is the use of the household income decided? Who makes the decision? Do you involve in the decision-making? How do you think that affects your access to the healthcare services including PP and PA family planning?

R; similar to that of resources our allocated for everything in our home after deciding together, like the income we also discuss on which type of family planning methods my wife should which is comfortable for her then decide to use one of it.

I; In your community, how is use of PP family planning seen? Is it acceptable for women to use PP family planning? Why/why not? Please tell me more

R; Currently the mind of people has been changed they easily accepts what is told by health professionals even there are some woman who uses family planning methods hidden without telling to their husband, previously they say that it causes burning sensation to me, makes me thin. Husbands are also voluntary if their wife receives the family planning methods immediately after delivery because the time so difficult to grow children.

I; In your opinion what should be done to improve access to the PP family planning services for all recently delivered mothers in your community?

R; to improve the access involving one to thirty leaders in the activity and creating opportunity to them to teach the community should be needed, as I told you the time is difficult to grow up children who knows the coming year will be worse than the current, some of husbands don't considers even their pain and starts to do sexual intercourse after fifteen days of delivery an they

may get unplanned pregnancy again. So the mothers should be aware about using family planning methods immediately after delivery n by the help of one to thirty leaders. The other one is health extension worker should follow recently delivered mothers in their community they should ask mothers about whether they used family planning method or not if not they should provide them in their home before six weeks of delivery, they should also assure from health center who has received or not and if not they should give them a vaccination.

Practices, Roles, and Participation

I; Please describe to me the typical women's roles in your community?

R; previously mothers engaged in activities related with household, like cooking food, cleaning house and bearing children and growing them. But currently they can do every kind of activities equal with males, they work in farm with their husband, go to school, can do business activity

I; how are women and girls occupied over the course of 24 hours? Are there seasonal differences in how women and girls use their time?

R; the woman is backbone for one house, she washes the clothe of her children, cooking food for them and feeding them is not less than farming the land. She can work load when her children goes to the school because at that time all activities were over her.

I; What kind of work do you do? Do these works change when your age or status changes (young, unmarried and married, older)?

R; I'm working as government employee, merchant, and also doing my private business as cameraman. The work of my wife can be changed when our children get grow and can give her rest by doing some activities

I; Do you have barriers on your access? What barriers? How do the barriers/restrictions influence your access to the PP family planning services and supportive social networks?

R; the first barriers can be drug side effects most of the time five year family planning method is not comfortable for woman who has no good living condition and not get sufficient amount of food in their home, at that time they forced to change to three month drug.

I; Do you participate in any family, community, government, or other social networks organizations? Please tell me more.

R; yes I used to participate in social or governmental networks and my wife too.

I; Please tell me how your participation in these organizations affect your PP family planning services use

R*; when we participate in different social networks people talk about side effects of family planning methods they say that it can increase bleeding, causes body burning sensation, causes dizziness, making them thin and the like, people also complain that this year for us is the most challenging year and we faced scarcity of food so using this for us now is the difficult thing for us .*

I; In your community do women assume leadership roles? What types of roles? Do these roles change by social characteristics, such as (dis)ability, economic status, health status, educational level, religion, culture, geographical location, and marital status?

R; yes I know that females assigned as leaders at Keble label, for example women affairs is female and also selected as leaders in different places. They are assigned based on their educational status, ability to communication, ability to convince others, being influencal among others, considered as important person in the village. This kind of woman assigned irrespective of educational status, and social background

I; Please tell us about your own experience with assuming any leadership roles?

R; I used to selected and served as leader of developmental association at Delbo Keble, served as leader of Keble youth at 2006 EC, served as sodo zurya youth federation member for two years, then served as youth league at Delbo, then served as delbo court office registrar for four years, currently I m serving as delbo youth league leader.

I; Please tell me how your leadership roles affect your PP family planning services use

I m serving as positive deviant in dissemination of health information, for that I worked with different NGOs who aware community about prevention of HIV AIDS, using of family planning methods ane keeping our environment clean. By getting such opportunity, I tried to share health information to youths and community, at that time they complains about side effect of drugs an now they accepted to receive it at any time even immediately after delivery.

I; In your community who decides at what age should people marry? What are the reasons for a women getting married at younger or older ages?

R; those going to marry decides their age of marriage based on their feeling and love of each other, most of they depend on their feeling they don't considers whether they are capable to lead family or not educationally or economically.

I; Please tell us about your own marriage experience? How old were you when you marry? What are the reasons you she get married at that age?

R; when I marry my wife we both had equal feeling to be engaged in marriage, when I marry her I were 25 years old young.

I; Please tell me how your age at marriage affects your PP family planning services use

R; At that time we don't think about using of family planning methods all things we understood through time. After we born our first daughter we decided to receive three-month injection from health center.

I; What are your suggestions to improve women's roles and participation to enhance the use of PP family planning services?

R; participating previously received mothers as role models, participating one to thirty leaders and health extension workers on sharing information

Knowledge, Beliefs, and Perceptions

I; In your community what is appropriate behavior for recently delivered woman regarding use of PP and PA family planning services? How do they differ from adolescent girls and young women?

R; most of people are voluntary to receive service, when I hear some women they I have already received the drug when they discharge from health center after delivery, but there is few women who refuses to use it immediately they want to stay and receive it later, and also some of them think it as negative thing because their mind has not been changed. And also at that time people fears to use long acting family planning method rather they prefer to use three month injection

I; What is the appropriate behavior for a recently delivered mother? How do these believe influence health behavior including the PP family planning use?

R; previously it is not familiarized that using family planning methods immediately after delivery they practiced that using It after two or three months this can expose them to unwanted pregnancy

I; What are the social beliefs and perceptions that share women's expectations and aspiration for PP family planning?

R; currently people don't rise it as big deal because the mind of people has been changed and their thinking status has been increased. May be few of them think as negative thing and have misconception regarding it

I; In your opinion who should make decisions regarding use of PP family planning services? What types of decisions do women make regarding use of healthcare including PP family planning services? What types of decisions regarding use of healthcare including PP family planning services are made jointly?

R; first of all I should decide to my wife to use family planning methods and should encourage her to use it because unwanted pregnancy can happen and she may suffering from it without growing her first baby. I should encourage her to receive it immediately after delivery and we should reach decision for this during pregnancy time. Sometimes woman can say I am suffering from burning effect of it but not you at that time further convincing is required from her husband he has fulfill her weakness. If they both decide they considers about side effect and the husband can give special care for her.

Legal Rights and Status

I; In your community are women who delivered recently legally allowed to receive PP family planning service?

R; first of all the service has not been started by her and also it is being given for long period of time but this is only modified by timing of giving time. She should know that her husband may ask her to do sex even before six weeks of delivery and finally all problems comes to her, so health extension workers should give her adequate knowledge regarding the issue. So the woman should receive it happily and this issue cannot be brought to think it as legally or not legally.

I; How do recently delivered women access PP family planning information and resource from the healthcare facilities?

R*; they get the information and resources from nearby health post through health extension workers, from one to thirty leaders at the community level before this they teach community on eighteen health extension packages and making this one as additional, because they are even closer to mothers from health professionals and also they are capable of disseminating the information regarding health.*

I; Is these same for boys and girls, people from different age groups, education background, ethnic groups, socio economic classes, excluded groups? How are they different? Why/ Why not?

R; Young males can get information from the school, university, mass media

I; Do the people in your community support recently delivered women to access PP family planning infatuation and resources? How do they provide support? Who are these people? Why do they provide support?

R; There are some people who received especial training from health extension workers like one to thirty leaders, one to five leaders supports recently delivered woman to receive it. Others mothers also who have changed mind status also asks recently delivered mothers whether they get the service or not and encourages mothers to go and receive the service even before six weeks.

I; Are there certain groups in your community who do not support this? Who are these people? Why don't they support?

R; may be few of them can oppose those who have no changed mind, but they have no right to enter in someone's personal life.

I; Please tell me your experience with accessing healthcare information and resources including PP family planning.

R; I used to get health information from health professionals during their home to home visit, they teach us during meeting time, give us leaflets when we go health center

I; Do you feel you have a right to get healthcare information and resources including PP family planning services? Why/why not?

R; yes I feel I have right to get health information and resources because I myself benefited from the service.

I; Do you think your experience is different from other people in your community? How? Please tell me more

R; others may get from school, mass media or from their pears

I; Is there legal mechanism you can follow to ensure you get quality healthcare information and resources your need? Please tell me more

R; yes I have legal mechanism to follow if they dot give me the quality service or information I will inform to health center head but such kind of problem has not been happened nowadays

****

Summary

This interview conducted with recently delivered woman husband at Delbo health center in 27/07/22. He has diploma and both government and self-employed.

Key findings were- receiving family planning methods within 24 hour of delivery is very important. male are not voluntary to receive family planning method, the decision should be made between both husband and wife, people accepted and understood its importance currently. to increase the service the health extension workers should follow recently delivered mothers weather they received or not if not they should give them by going to their home. The first

challenge too receive pp family planning method is its side effect and peoples poor living condition

**Code**	**IDI_ DE _WO_ 30_6_22**
**sex**	**Female**
**Age**	**25**
**Educational status**	**10th grade**
**Occupation**	**Farmer**
**No of children**	**3**
**Date of delivery**	**(oydu giya) at the time of interview (1month)**
**kebele**	**Delbo**

I: Health services (PP and PA family planning services) availability: Can you tell me the types of healthcare facilities that provide PP family planning services in your area?

a. What types of FP services are available at the healthcare facilities?
b. Are these services equally available to boys, girls, young, old, married, unmarried, people from different geographic locations, religious or cultural background, language etc.? Please tell me more

R: Currently I am using ‘‘mittiyooga hah’I ekaas’’ oral contraceptives. I used to use (a method given for three years) implants In the first delivery and I used Depo in the second delivery and now this is third time when I use family planning. Health care workers gave me oral contraceptives immediately after birth. In my opinion this service is available and given for boys ,girls married and unmarried ones.

I: Can you tell me how far the PP family planning services providing facility is located? How long does it take for you to access the facility?

a. What type of transportation means do you use to reach the facility? How easy or difficult is it to use the transportation services?
b. Is there any cost associated with accessing the PP family planning services? Please tell me more about this
c. Is the PP family planning service open at all time and is accessible with short waiting time at the healthcare facility? Are there types of FP methods of users' choice in the healthcare facility? Do healthcare providers receive and treat users with respect and dignity?
d. Are these services equally accessible to boys, girls, young, old, married, unmarried, people from different geographic locations, religious or cultural background, language etc.? Please tell me more
e. How easy could you access the PP family planning service? Do you think your access to the PP family planning services is affected because of your background? How?

R: It takes one hour from the health center. It found in zala shasha kebele. I use motor bicycle for transportation. It is far from here.

I: Is there any cost associated with accessing the PP family planning services? Please tell me more about this

R:There is no cost associated with getting the service except transportation cost.

I: Is the PP family planning service open at all time and is accessible with short waiting time at the healthcare facility? Are there types of FP methods of users' choice in the healthcare facility? Do healthcare providers receive and treat users with respect and dignity?

R: I can get the service at any time. I can use the service based on my preference. The health care workers are good. They taught me during antenatal follow up. They told me all the options and they told me that there are different family planning methods given for 3 years, five years, and they also told me that there is family planning method given for 12 years and I told them that the method given for three years is not comfortable for me because I am busy with home matters, I get tired when I use it from my previous experience. I told them that the methods given for long time are not comfortable for me ,I told them that I better use oral contraceptives ones and they

agreed with my idea and gave me ‘mittiyooga’ oral contraceptives. The health workers were good because they treated me well.

R:Yes in my opinion all the services are given for all people in the same manner. For me there is no barrier for using the service.

I: Please tell me about your household resources and assets. Please tell me the resources and assets belong to you.

a. How is the use of the household resources and assets decided? Who makes the decision? Do you involve in the decision making? How do you think that affects your access to the healthcare services including PP family planning?
b. Please tell me about your household income. Please tell me about your own income.
c. How is the use of the household income decided? Who makes the decision? Do you involve in the decision making? How do you think that affects your access to the healthcare services including PP and PA family planning?
d. In your community how is use of PP family planning seen? Is it acceptable for women to use PP family planning? Why/why not? Please tell me more
e. In your opinion what should be done to improve access to the PP family planning services for all recently delivered mothers in your community?

R: I have domestic animals ,cow, ox, donkey. We get income from this animals. We don’t have our own land.

I: How is the use of the household income decided? Who makes the decision? Do you involve in the decision making? How do you think that affects your access to the healthcare services including PP and PA family planning?

R:it my husband who decides on these matters. But he is not the only decision maker we discuss on different matters and decide jointly. There is no barrier to get family planning service because I have already planned to get the services. Because I use the service voluntarily without the influence of some one. I come on foot or using motor bicycle It is must for me to get the service, nothing hinders me from using the service.

I: In your community how is use of PP family planning seen? Is it acceptable for women to use PP family planning? Why/why not? Please tell me more

R: There is negative attitude within the community. Rural people raise a question 'why are you rushing to use family planning?it is too early to use, *''hanikeenan aybe oli eqide'',it is better if women get the service when they become stable and healed.*

After delivery there were many people relatives with me. I communicated with the health worker using body language ''qimo'taas'' because there were many people in the room. The health worker understood my condition ,what I wanted to convey through my body language or '' qimootaa''

I: why did you communicate in this manner?

R:Because the health worker is about to give the service ,I did not want to use the service before them.It is obvious that we will not have sexual intercourse immediately until I get healed but the community perception is not good. They perceive as if we are going to have sexual intercourse immediately if I start family planning immediately after birth. People are backbiting us if we start family planning immediately after birth. Personally I wanted to use the service immediately after birth because I don't have anybody who supports me ,because I am busy with home matters I will be in a difficulty if don't use family planning immediately after birth. I want to stay for five six years so that is why I wanted to use family planning.

I: In your opinion what should be done to improve access to the PP family planning services for all recently delivered mothers in your community?

R: Family planning should be taken in secret, only my husband and wife should know. If I don't give birth with in one year interval the community say ''why is she not getting pregnant? Eager see what could be the reason for not conceiving '',in addition to this if I get pregnant and give birth without spacing they still say '' xossi I yeluwape asho'' discourage not to give birth. People should not know whether I use family planning or not. One of the problem I observed among health care workers is that they don't follow mothers after they gave the service. There is a mother in my neighbor who is using family planning method which is given for five years. She is daily laborer. She using the service but continuously bleeding but nobody asked her why that is so. The health care workers could have changed the method she is using, they are not treating

well, not serving us in a compassionate manner. This not only for this service but also when people come to health center for different reasons, they do the same thing. In our culture when you are entering into someone's house as a guest, the first thing that the guest sees his face ,the way how the person welcomes the guest. We as clients are not happy to visit the health center because they don't respect us. when someone comes to get service they simply sit and don't welcome us and they say I will come stay there. During this time the clients will be disappointed. That is why they are not happy to come. Sometimes we don't get them and we wait until they come. In previous delivery I went to private clinics for delivery because there was no health care worker by the time and I used to get depo from private clinics because of their absence in the health center. I Paid for the services in the private clinic.

I: So what should be done to improve such issues?

R:As far as I know the health center is supposed to be open and give 24 hour service and the health workers should work 24 hour. However, mothers don't get health workers at any time they want and they suffer a lot. Some may go to traditional practionars to terminate their pregnancy due to absence of health care providers. They pay extra many in private clinics and suffer a lot. The health care workers should avail themselves at any time. Some husbands are drunkards and will not listen to their wives when they are intoxicated they forcefully engaged in sexual intercourse and she may conceive at this time. During this time a mother may come to get services and get support or services from the health center. The other problem I observed is that there is only one bed in delivery room, there could be blood on bed after birth but the cleaners don't respect us if they see something unclean. A recently delivered mother is not stable and she is not in a position to keep the cleanness of the room but the cleaners disrespect us and shout on us if the room is unclean. ''*How can a recently delivered women be asked such a question ?''*

I: Please describe to me the typical women's roles in your community?

- How are women and girls occupied over the course of 24 hours?Are there seasonal differences in how women and girls use their time?

What kind of work do you do? Do these works change when your age or status changes (young, unmarried and married, older)?

R: ‘‘oshshaa kesays’’ , taking away the dungs of domestic animals ,‘‘buuccayas’’ cut and collect grass for animals, ‘‘mittaa qerays’’, collect woods for cooking, prepare breakfast, lunch and dinner, take care of domestic animals, I am engaged in all these activities.

I: Do you havebarriers on your access? What barriers? How do the barriers/restrictions influence your access to the PP family planning services and supportive social networks?

- In your community do the restrictions/barriers change by social characteristic, such as (dis)ability, economic status, health status, educational level, religion, culture, geographical location, and maritalstatus?

R: I came to know about the service in this year because the health workers counseled me during follow up time during pregnancy. They told me all the options and available methods in the health center. They told us to start family planning method in order to prevent unintended pregnancy after immediately after delivery. Then I agreed with their idea and said ok to use. It is very good if I start to use a method given for 12 years but I am busy person because It is me who is responsible collect grass for cattle, clean their dungs, so I get tired if I use this method. Other than this there is no other barrier hinder me from using the services.

I: Do you participate in any family, community, government, or other social networks organizations? Please tell me more.

- Please tell me how your participation in these organizations affect your PP family planning services use

I: Yes I have participated. we have one to thirty leader but they don’t teach us. The kebele people also discuss about their issues and conclude their session without discussing. If there is training one to thirty leaders don’t allow any body to attend rather they attend training for themselves. But I have participated in different gatherings. This helped me a lot because they used to counsel us, they encourage us to use different services and I accepted their advise.

I:In your community do women assume leadership roles? What types of roles? Do these roles change by social characteristics, such as (dis)ability, economic status, health status, educational level, religion, culture, geographical location, and maritalstatus?

- Please tell us about your own experience with assuming any leadership roles?
- Please tell me how your leadership roles affect your PP family planning services use

R:They participate and play different leadership roles.

I: In your community who decides at what age should people marry? What are the reasons for a women getting married at younger or older ages?

- Please tell us about your own marriage experience? How old were you when you marry? What are the reasons you she get married at that age?
- Please tell me how your age at marriage affects your PP family planning services use

R: Nobody decides because this is up to the adolescents, they decide by themselves, no body interferes up on their decision. Currently adolescents get marry without their family's decision ,they may get to know each other in school and get children. This can affect family planning service utilization because of their age they may not know what to decide.

I: What are your suggestions to improve women's roles and participation to enhance the use of PP family planning services?

R: their role is ..mmmm..?

I: what should women's participation and role to improve the use of family planning service given immediately after birth and after post abortion? how?

R: Women should participate to enhance the service but they have their own challenges.

I:what are those challenges?

I:women usually have a problem of forgetting, they forget their date of appointment because they are busy and engaged in different tasks and forget to take the family planning methods. Sometimes there is problem from health care providers side because they write in English language on the follow up card which is not clear for all of us because most of the people are un educated. It would be clear if they write in amaharic language. I may understand English where as others may not understand it means. Mothers can ask anybody, their children if it written in amaharic language. They should not write the date of appointment in English because mothers may forget their appointment date.

I: In your community what is appropriate behavior for recently delivered woman regarding use of PP and PA family planning services? How do they differ from adolescent girls and young women?

1. What is the appropriate behavior for a recently delivered mother? How do these believe influence health behavior including the PP family planning use?
   What are the social beliefs and perceptions that share women's expectations and aspiration for PP family planning?

R: Mothers who aborted want to have children as soon, as early as possible without delay because they lost or missed the opportunity of having child, so they don't want to use family planning. The community also don't encourage a mother who aborted to use family planning. Both the community as well as mothers who aborted don't encourage using family planning after abortion. Recently aborted mothers want to have child because they think that God favored them if they get pregnant as soon as possible.

I: What do you think so? what is your perception on this?

R: As to me it is good to take some time once a mother aborted and wait some time until next pregnancy because if she get pregnant immediately , this can affect her. Therefore, it is better to use family planning as soon as possible. I should plan for myself and God provides what I need.

I: In your opinion who should make decisions regarding use of PP family planning services? What types of decisions do women make regarding use of healthcare including PP family planning services? What types of decisions regarding use of healthcare including PP family planning services are made jointly?

R: We should decide jointly regarding use of these services. Because we know our economic status, we know all the challenges we face. In our culture , more than twenty or thirty people at a time visit a mother who gave birth. In both cases after delivery or abortion it is common for the community members to visit those mothers. They cost a lot to welcome the guests. They loose all what they have in order to serve the guests. Therefore, it is good to decide jointly on this matter.

I: In your community are women who delivered recently legally allowed to receive PP family planning service?

- Is these same for boys and girls, people from different age groups, education background, ethnic groups, socio economic classes, excluded groups? How are they different? Why/ Why not?

R: Yes they are legally allowed to receive the services. Nothing prevents them from using. boys and girls, people from different age groups can use the service.

I: How do recently delivered women access PP family planning information and resource from the healthcare facilities?

- Is these same for boys and girls, people from different age groups, education background, ethnic groups, socio economic classes, excluded groups? How are they different? Why/ Why not?
- Do the people in your community support recently delivered women to access PP family planning infatuation and resources? How do they provide support? Who are these people? Why do they provide support?
- Are there certain groups in your community who do not support this? Who are these people? Why don't they support?

R: I heard about immediate family planning service during the current pregnancy during follow up, the health care workers told me during counseling. I did not know before. In the previous delivery I used to get service after long time it was not immediately after birth like that of the current one. During the follow up period the health care workers told us the methods available in the health center and asked our preferences.

I: Please tell me your experience with accessing healthcare information and resources includingPP family planning.

- Do you feel you have a right to get healthcare information and resources including PP family planning services? Why/why not?
- Do you think your experience is different from other people in your community? How? Please tell me more

- Is there legal mechanism you can follow to ensure you get quality healthcare information and resources your need? Please tell me more

R: The community members access health care information but they are not willing to accept. Even I used to discourage mothers who are using the service. There was a friend of me who delivered here and I came here to seek care and ask her. My friend who recently delivered had inserted a method given for five years on her arm immediately after birth. I asked my friend what it is. She told me that the health workers inserted the family planning method given for five years and I asked why? Because I did not know that I would start to use the service after birth…loughing. My friend's husband lives in moyale and she lives here and I asked her that your husband is somewhere in moyale, why are you using the family planning method and the nurse heard what I am saying and she said go out because I discouraged my friend not to use the service. I did not know that I would start to use the service immediately after birth. Then I started to use the method I said before immediately after birth ‘‘mittiyoga”. They community don't like to use the service immediately after birth because here in rural setting we don't have conducive atmosphere or condition to use ,we don't have enough food, no body give care for us. So if we start to use the service we may loose weight.

I feel that I have right to get the service but someone has to tell us about the service unless we don't have any source. From where do we get information unless someone tell us. I can get information if it was disseminated through announcements in the kebele.

I: With this we have come to an end of the discussion. Do you want to add anything before we end the discussion or if do you have any questions you want to ask?

R: In order to attract the clients there was coffee ceremony after delivery in the health center. They used to give care for recently delivered women but now there is nothing done for mothers after delivery. As a result most of people are not happy to come here to get services. Some of the community members even tell their previous experience and say we used to give birth at home, and they don't want to come here. When compared female health workers and male ones ,males are somehow good. Females are mistreating and they don't respect us although they are like us. Females are not serving in a good manner. Sometimes we don't know what to do because of our

pain but health workers don’t understand this.So ,we used to say “awu boossha?” where should I go? Because they mistreat us. Therefore, they should get education so as respect the community. If they respect and give care the community will be satisfied and willing to come to get the service.

I:Thank you

R:Thank you “xoosi immo”

Summary

This is in-depth interview conducted with recently delivered woman in Delbo health center on 30/6/2022.

- ✓ Have knowledge on the availability and accessibility of the services but the service is not easy to access due to the distance.
- ✓ However, distance is not mentioned as a barrier to use the service b/c the woman mentioned that this couldn’t hinder from using the service
- ✓ The community perceive that it is too early to use family planning ,it is better to stay and use family planning latter after a woman becomes stable and healed.
- ✓ Recently delivered woman is fear of using family planning immediately after birth because of fear of backbiting of family members, people who accompany her during delivery
- ✓ Recently aborted women don’t like to use family planning immediately after abortion because they want to replace or they want to have children as soon as possible.
- ✓ The use of long acting family planning is seen as something which can affect their health and they prefer pills over long acting family planning method.
- ✓ Health workers don’t follow mother once they inserted the family planning
- ✓ Initially the woman said that the health providers treated her well however, she turned her idea while probing and told us that health care workers, cleaners mistreating people, don’t respect mothers who came for the service, therefore, they prefer to go to private clinics. Ambivalent kind attitude is observed.

- ✓ There is communication gap between health care workers and mothers because the health workers write in English language on their appointment dates so that they may not understand, may forget their appointment dates.

Discussion

Rapport Building

Thank you for proving consent to participate in the discussion. There are no right or wrong answers, please share your frank opinion, as it will help us in understanding the situation better.

Enquire about her recent delivery experience. An example is given below:

Please tell us about your recent childbirth experience. Where did you deliver? Who attended you during delivery? If you deliver at health facility how far is it from your home? How do you travel to the health facility?

Assets and Resources

Discussion

Now we are going to discuss about the Healthcare resources and assets available to recently delivered mothers in your area.

1. Health services (PP and PA family planning services) availability: Can you tell me the types of healthcare facilities that provide PP family planning services in your area?
   a. What types of FP services are available at the healthcare facilities?
   b. Are these services equally available to boys, girls, young, old, married, unmarried, people from different geographic locations, religious or cultural background, language etc.? Please tell me more

Such services “PP and PA family planning services” are provided in this health center. There are also other facilities that provide such services. It is also provided in private clinics. If we don’t get in health center, we ask private clinics. For my case we go jointly to get the service. We receive the service based on mutual agreement. I don’t force her to use, rather we agree and go there to sue the service. People around the church and school teach people to have a balanced family. They don’t provide the service directly, but they teach about having a balanced family with our income. The types of family planning available are the one used for five years, for three years, and depo. I don’t think condom is applicable for those already married.

I know the services are available for my wife since most of the staffs are her friends and they serve her positively. Since I am a busy man as you see, I don’t know whether the services are available for other or not. I don’t how they serve others. I don’t have a much information about others, but when I go with my wife, I saw they “health care providers” are serving well those who come to get the services. Sometimes they have shortage of some types of family planning. In addition they health care providers are also reluctant when they were not paid such as duty payments. So, sometimes they are not available. When extra time work is not paid, they will not be available around the facilities. When I saw them during my visit to the health health center, I saw them giving services for those coming from rural areas as well. In rural areas I know health extension works are also providing education and services to mothers.

2. Accessibility of the PP family planning services
   a. Can you tell me how far the PP family planning services providing facility is located? How long does it take for you to access the facility?

b. What type of transportation means do you use to reach the facility? How easy or difficult is it to use the transportation services?
c. Is there any cost associated with accessing the PP family planning services? Please tell me more about this
d. Is the PP family planning service open at all time and is accessible with short waiting time at the healthcare facility? Are there types of FP methods of users' choice in the healthcare facility? Do healthcare providers receive and treat users with respect and dignity?
e. Are these services equally accessible to boys, girls, young, old, married, unmarried, people from different geographic locations, religious or cultural background, language etc.? Please tell me more
f. How easy could you access the PP family planning service? Do you think your access to the PP family planning services is affected because of your background? How?

The health center is not far from the place where I live. It is less than 10 minutes. We can walk slowly be foot or we can use motorcycle since I have one. I don't know the services are accessible for unmarried people. It is up to the health care providers and they know it whether it is allowed or not. If it is allowed for boys and girls, health care providers know it and do what they know. I don't have any knowhow about that.

The health care providers treat with a big care for pregnant mothers and those who seek family planning as well. They give a maximum care and attention. For example my eldest son is about 8 years, the next one is a girl and she was born in 2011, and now I got the third one this week. I have about four years gap in between.

I think they treat well those who come from rural areas as well. they "rural community" may not have enough information regarding family planning, but when they come to the facility I belive they treat them well.

3. Means to access the PP family planning services
a. Please tell me about your household resources and assets. Please tell me the resources and assets belong to you.
b. How is the use of the household resources and assets decided? Who makes the decision? Do you involve in the decision making? How do you think that affects your access to the healthcare services including PP family planning?
c. Please tell me about your household income. Please tell me about your own income.
d. How is the use of the household income decided? Who makes the decision? Do you involve in the decision making? How do you think that affects your access to the healthcare services including PP and PA family planning?
e. In your community how is use of PP family planning seen? Is it acceptable for women to use PP family planning? Why/why not? Please tell me more

About household asset, thank God, it is good. I have everything I need for living. I have a good house, sofa, TV, control shelf, freezer, chairs and tables. I have what I need, thank God. Regarding decision making it is me who pass the decision. Family planning decision is made by me because most of the burden falls on me. It is my responsibility to teach, provide, and fulfill what they need. It is my responsibility to fulfill more than 70% of their requirements. So, I am the one who make the decision. When I make the decision, she also accepts. I was the one who told her to use the family planning serving for three years. Sometimes she is afraid to use it due to fear that it may cause further delay of birth.

The income is from this shop I am working. After covering other expenses, we put around 4000 birr weekly “equb”.

Some people are too religious to use it “PP family planning”. They say Abraham was told in the bible to be multiplied. So we don’t have to use family planning. They think it as a sin. Some people think it as something bad and have a negative view towards it. This is due to lack of knowledge. Some people support because it has advantages. Those who accept say Luke who is in the bible is also a doctor. So, God gave us doctors to listen to them and use what they give. They accept using family planning. Besides, having a gap between children helps them to have a quality child and health mother. It is useful for the health of the mother and a child as well.

4. In your opinion what should be done to improve access to the PP family planning services for all recently delivered mothers in your community?

First of all, it is health care providers who know about this question. But I believe health care providers should go to their home and provide the service before they come to health facilities. Health care providers should go to their house “recently delivered mothers” and show the quality of shortly spaced and those who have long gaps between births. They should teach mothers very well.

Practices, Roles, and Participation

Now we are going to discuss about your women’s and your role and responsibilities.

1. Please describe to me the typical women’s roles in your community?
    - How are women and girls occupied over the course of 24 hours? Are there seasonal differences in how women and girls use their time?

The work of women is more than men in the community. They are the ones who prepare a breakfast, they clean household, and they prepare lunch, then after they go for social services. If they don’t go there they also help in business such as working in retailer shops and other activities. For example my wife helps me in this shop business. They work from breakfast to lunch and from lunch to dinner. Even after that they work such as cleaning the house and utensils as well. The work burden is more in summer times since the summer time is dirty and they have to clean the house again and again.

2. What kind of work do you do? Do these works change when your age or status changes (young, unmarried and married, older)?

I have completed first degree in Economics from Wolaita Sodo University in addition to working in this shop.

3. Do you have barriers on your access? What barriers? How do the barriers/restrictions influence your access to the PP family planning services and supportive social networks?
    - In your community do the restrictions/barriers change by social characteristic, such as (dis)ability, economic status, health status, educational level, religion, culture, geographical location, and marital status?

The barrier can be lack of knowledge due to shortage of education from health care providers. They should teach about the benefit of it. The main reason I believe is shortage of information. There is also belief that before having a first child using it ”FP” will prevent a chance of having a child. I don’t think so, but there are people who think so. I would like also to ask you a detail about that. But I don’t know about whether not married and young boys and girls can use it. Health care provider may know about that.

4. Do you participate in any family, community, government, or other social networks organizations? Please tell me more.
    - Please tell me how your participation in these organizations affect your PP family planning services use

I didn't participate in any such meeting. No one called me for such meeting.

5. In your community do women assume leadership roles? What types of roles? Do these roles change by social characteristics, such as (dis)ability, economic status, health status, educational level, religion, culture, geographical location, and marital status?
    - Please tell us about your own experience with assuming any leadership roles?
    - Please tell me how your leadership roles affect your PP family planning services use

I know some of women were appointed in different position is gov't offices as house speaker of the woreda and other positions. Some of my friends' wives were appointed in different administrative positions. I think those who are in position will teach others to use more since they will have more responsibility to do so. I believe coming to some positions will make them more responsible and knowledgeable regarding family planning.

6. In your community who decides at what age should people marry? What are the reasons for a women getting married at younger or older ages?
    - Please tell us about your own marriage experience? How old were you when you marry? What are the reasons you she get married at that age?
    - Please tell me how your age at marriage affects your PP family planning services use

The marriage age is depends on the ones who get married. It is them "boys and girls" who decide about marriage. Most of the time boys marry about the age of 20 and girls around 15 years. Since it is a hot climate area girls marry in younger ages. Having a hot climate exposes them to other things "increased sexual desire".

I was about 25 years and she was around 18 years when we get married. We get married in 2004 E.C and she gave birth in 2006. We used depo in the middle for some time. when we get married, we were not economically good. We used family planning due to economic reasons.

7. What are your suggestions to improve women's roles and participation to enhance the use of PP family planning services?

Women should be taught and supported by health care providers than even husbands. They "HCP" should increase their participation to use family planning.

Knowledge, Beliefs, and Perceptions

Now we are going to discuss about the issues faced by adolescents and women in accessing PP and PA FP services in your opinion.

1. In your community what is appropriate behavior for recently delivered woman regarding use of PP and PA family planning services? How do they differ from adolescent girls and young women?
    a. What is the appropriate behavior for a recently delivered mother? How do these believe influence health behavior including the PP family planning use?

First, I myself encourage my wife to use family planning after three months of delivery. She wants to use it and I also think so. I don't know much about the behavior from the community may be health care

worker know it. Community doesn't know whether a recently delivered mother is using family planning or not. So, I didn't hear much about that.

2. What are the social beliefs and perceptions that share women's expectations and aspiration for PP family planning?
3. In your opinion who should make decisions regarding use of PP family planning services? What types of decisions do women make regarding use of healthcare including PP family planning services? What types of decisions regarding use of healthcare including PP family planning services are made jointly?

She should make the decision since she is the one who whose health is affected more. Joint decision is necessary I believe, but economic conditions are also considered. So, I should be the one who should make the decision. The first idea should come from me to whether to use family planning or not. Then after we should discuss it and agree to use the type such as the five years or the three years one.

Legal Rights and Status

Now we are going to discuss about the marriage related process prevalent in this community.

1. In your community are women who delivered recently legally allowed to receive PP family planning service?
    - Is these same for boys and girls, people from different age groups, education background, ethnic groups, socio economic classes, excluded groups? How are they different? Why/ Why not?

The law is from the government and health "facility" is also from government. The source is one, so the use is legally allowed. The law supports use of family planning for everyone.

2. How do recently delivered women access PP family planning information and resource from the healthcare facilities?
    - Is these same for boys and girls, people from different age groups, education background, ethnic groups, socio economic classes, excluded groups? How are they different? Why/ Why not?
    - Do the people in your community support recently delivered women to access PP family planning infatuation and resources? How do they provide support? Who are these people? Why do they provide support?
    - Are there certain groups in your community who do not support this? Who are these people? Why don't they support?

In rural areas, health extension workers are responsible for visiting and teaching mothers about that "PP". It is their responsibility to go and teach them. In urban areas, health care providers should teach mothers. I don't know any support group and I don't know also who don't support.

3. Please tell me your experience with accessing healthcare information and resources including PP family planning.
    - Do you feel you have a right to get healthcare information and resources including PP family planning services? Why/why not?

- Do you think your experience is different from other people in your community? How? Please tell me more
- Is there legal mechanism you can follow to ensure you get quality healthcare information and resources your need? Please tell me more

Most of the time, my wife goes to the health center and get the information and I get from her. I believe I have a right to get information and resources regarding family planning.

With this we have come to an end of the discussion. Do you want to add anything before we end the discussion or if do you have any questions you want to ask?

Thank you for your time and in case you want, you can get in touch with us through the details mentioned in the consent form.

****

Summary:

I conducted this interview at the shop of the respondent where he sell different motorcycle spare parts. His wife gave birth on the week when the interview was conducted. Before we reach him, we searched for the other two husbands and we couldn't find them in their house and wok place. This respondent was available and willing participates in the interview. In the middle of the interview some customers come to buy different things. I ensured him to fee free at any time to do his business and we agreed to pause when the customers come. At least three customers visited and we were comfortable to continue our interview from the point where we stopped. Finally he asked me whether it is possible to use condom among married couples and whether it possible for unmarried ones to use family planning of three years and five years method. I responded to both questions from scientific points of view all types family planning methods available to use for anyone who wants to use them.

Discussion

Rapport Building

Thank you for proving consent to participate in the discussion. There are no right or wrong answers, please share your frank opinion, as it will help us in understanding the situation better.

Enquire about her recent delivery experience. An example is given below:

Please tell us about your recent childbirth experience. Where did you deliver? Who attended you during delivery? If you deliver at health facility how far is it from your home? How do you travel to the health facility?

Assets and Resources

Discussion

Now we are going to discuss about the Healthcare resources and assets available to recently delivered mothers in your area.

1. Health services (PP and PA family planning services) availability: Can you tell me the types of healthcare facilities that provide PP family planning services in your area?
   a. What types of FP services are available at the healthcare facilities?
   b. Are these services equally available to boys, girls, young, old, married, unmarried, people from different geographic locations, religious or cultural background, language etc.? Please tell me more

Eventhough i don't know their names their names there are professionals who provide the service in the health facilities. in the facilities they give like the one working for three months, the other which is buried under the arms, and the other that is inserted in the uterus. The services are provided in all health facilities like the hospitals, the health centers, and health posts. They provide the service according to the choice of the people. It can be for three months, for three years, or the one that is given monthly to be taken orally. I think there are four kinds of birth protecting medicines. In all areas they provide according to the choice of the people. For example I attended my antenatal care at Bitana hospital. The service is also given there. They first ask for the choice of the people and give them accordingly. I also followed my antenatal care there and the service for that is also good.

Whether it is the injection contraceptive or any other type, they don't ask if they are married or not married. They don't have any right to ask them for their marital status. There is no such a thing. If someone wants to protect pregnancy no one can ask them if they are married, not married, young, or old. Since those who went there know themselves and their right, no one can ask them so. Those who are married can also take and those who are not can also take from the facilities. It is given for anyone who wants to use the service. It is given for anyone who wants to use the service. I don't think there is discrimination based on where people come from. It is the government's service availed for all people. It is not something that you eat or drink. It is the drug that helps to protect pregnancies and made available by the government. Even yesterday after I gave birth to a baby, they said the device that is inserted in to the uterus is better than others and why don't we give you this service? They asked to put in to my uterus, but I denied until I will get better. Even before that when I went last time for malaria treatment at the health center and I saw when they ask a mother to put the device in the uterus to protect pregnancy. But, the woman refused and went back to her house. They asked the women to use the method, but she denied. She came from far rural area, but they asked her to give the service. They

don’t discriminate in this regards. They also asked me the same thing. That means the treatment is the same for both urban and rural people. I believe they give the same service for all people.

2. Accessibility of the PP family planning services
   a. Can you tell me how far the PP family planning services providing facility is located? How long does it take for you to access the facility?
   b. What type of transportation means do you use to reach the facility? How easy or difficult is it to use the transportation services?
   c. Is there any cost associated with accessing the PP family planning services? Please tell me more about this
   d. Is the PP family planning service open at all time and is accessible with short waiting time at the healthcare facility? Are there types of FP methods of users’ choice in the healthcare facility? Do healthcare providers receive and treat users with respect and dignity?
   e. Are these services equally accessible to boys, girls, young, old, married, unmarried, people from different geographic locations, religious or cultural background, language etc.? Please tell me more
   f. How easy could you access the PP family planning service? Do you think your access to the PP family planning services is affected because of your background? How?

The health center is next to my house. But people from Charicho or Monopol area are more disadvantageous with regard to the distance. They have to use a motorcycle. Not only this service, but it is difficult for them to get the treatment for malaria as well. it is far from here. They have to pay some 50 birr for one round and 100 birr for coming and getting back. When they get sick, it is difficult for them to come and get treatment. It is far for them. For us we can use the service from the health center here and we can go to Bitana by paying only 40 birr for round trip. It was even 30 birr only previously. People from Charicho and Timbaho Monopoly suffer a lot due to distance. If they are not the employee of the organization, other can’t afford the transportation cost. For us it is close to use and we can use simply the treatment.

There is no payment to use family planning. It is totally free. Even people who were sick with malaria get free treatment. It is free to take coartem even. Any family planning service is free. If they want to get a new card, they have to pay only 10 birr for that. Besides, other things are free in government facilties. In private facilities we have to pay. For example to buy coartem from the private facilities we have to pay 100 or 120 birr. In government facilities everything is free related with it or family planning.

Family planning is open in most of the times, except if there are some occasions where drug stocks out. Except that people can easily get the family planning they choose. If for example there is no depo, they tell you that there is no depo and give you other options to choose. Besides, they usually give the drug people chose easily. Most of the time, people want depo, but they want to give mothers the one that is inserted in to the uterus and people don’t want to use that. They say the one that is inserted in to the uterus is better than other methods and encourage us to use that one. They say like that, but I myself was scared to use this method. I am not comfortable with it. I use most of the time biological method. I don’t see the one inserted in to the uterus. I checked the one that is inserted in to my arms, depo and pills. But, none of them was comfortable for me. I checked them and I felt sick. The one that is inserted in to the uterus is scary and no one wants to use it. Everyone is scared to use it and no one is using it here in this kebele. May be people from the urban areas use this method. No one is using it here. I am too scared of it.

The health extension workers go all around the rural areas teaching people to use family planning. In the urban areas people easily go to the health facilities and get the services. they treat with respect. No one

is discriminated and no one is pushed back from using the service. The work ethics of providers doesn't allow them to do such a thing. No one is asked about his background. So, no is pushed back.

3. Means to access the PP family planning services
   a. Please tell me about your household resources and assets. Please tell me the resources and assets belong to you.
   b. How is the use of the household resources and assets decided? Who makes the decision? Do you involve in the decision making? How do you think that affects your access to the healthcare services including PP family planning?
   c. Please tell me about your household income. Please tell me about your own income.
   d. How is the use of the household income decided? Who makes the decision? Do you involve in the decision making? How do you think that affects your access to the healthcare services including PP and PA family planning?

Now we are living in a rental house. But both of us are government employees and we discuss on every matter that is important for us. household responsibilities are shared and we both engage making decisions. We discuss on every matter that belongs to us and make a joint decision. I don't know about others, but we discuss on all issues before making decisions. Thank God we both have income. For know we are living in rental house. But we make a joint decision and there is no superiority between us. he don't show superiority, and I also don't show superiority on him. By the way regarding family planning I want to give birth as quickly as possible, but it is him who refuses to do so. He says we are still living in rental house and we have to use family planning. Our first child is five years old and now we have here the second one. We discussed together to give birth to this second child.

The total net income we have is about 11,000 birr per month and we always discuss on how to use our resources. There is no superiority and inferiority among us.

   e. In your community how is use of PP family planning seen? Is it acceptable for women to use PP family planning? Why/why not? Please tell me more

People have their own view towards PP family planning. I believe people should take the depo injection after 45 days of giving birth. In rural areas people don't come around 45th day to the facilities. in the urban areas people come and use either loop or injection after 45 days. I would say 75% see taking PP family planning as something good and the rest 25% see it as bad. The ones don't using it say how can we use family planning before our body heals completely. Event there are other women who use before 45 days after birth and they say no one know what is going to happen. I know a lady who took the drug that is taken the 48 hours drug after birth and who got sick. She told me her story. But most of the time people see using PP family planning as good. About 75% people accept that way.

4. In your opinion what should be done to improve access to the PP family planning services for all recently delivered mothers in your community?

I was surprised when providers ask the woman to put a device in to the uterus immediately after giving birth. I was also shocked when they ask me to do the same. I don't know what you may think of it, but when they asked me that I said do you want to put something on this wound? People can have their own way. But I was shocked when he asked me to put a device in to my uterus after birth. Some people think to use family after they saw a mensus. But others use before seeing their mensus. But no one wants to insert a device in to the uterus immediately after giving a birth. It is impossible to imagine. It may look easy for providers to put it, but I was shocked and scared.

To improve access, training should be given for mothers and they should be well aware of family planning why are going to use.

Practices, Roles, and Participation

Now we are going to discuss about your women's and your role and responsibilities.

1. Please describe to me the typical women's roles in your community?
   - How are women and girls occupied over the course of 24 hours? Are there seasonal differences in how women and girls use their time?

In a day women do a lot of roles. Most of the time giving care for children is the role of women. Women also go to market places and there are many merchants in this town. They go from place to place to sell and buy different things. They do a lot of works. In this town all women are active. If you go any house, you will find them selling breads, Injera, "qoqer" and other things. No one is simply sleeping now a days. They are merchants. There are few or about 25% of the population in this town are government employees, the rest are all merchants I can say. So, women do household activities like cooking breakfast, feeding children, taking care of husbands, and then they go for marketing activities.

Those activities don't vary from seasons to season. They do the same activities.

2. What kind of work do you do? Do these works change when your age or status changes (young, unmarried and married, older)?

As I have told you we are teachers and we teach students. During summer times we have a break and we don't have any work.

3. Do you have barriers on your access? What barriers? How do the barriers/restrictions influence your access to the PP family planning services and supportive social networks?
   - In your community do the restrictions/barriers change by social characteristic, such as (dis)ability, economic status, health status, educational level, religion, culture, geographical location, and marital status?

For example some women say they fill discomfort and sick when they use PP family planning. Others say my husband doesn't allow me to use it. Some people say I don't have a milk so that to drink after taking family planning. Others in rural area have lots of activities and they forget to take family planning. Some rural women say in urban area people have food to eat so that they can use family planning. On the other hand some urban people say we don't have a milk to drink like the rural people, so we will not take family planning. Different people think differently. But most of the time in rural areas husbands doesn't want women to use family planning. They say let children be born and grow.

There is no church that says take or don't take family planning. There is also no such a think that taking family planning is related to disability. I never heard anything like that. People can use whoever they are. There is no church that thought not to take family planning. Disability also doesn't prevent from taking family planning. Being married or unmarried doesn't prevent form taking family planning.

4. Do you participate in any family, community, government, or other social networks organizations? Please tell me more.
   - Please tell me how your participation in these organizations affect your PP family planning services use

I participated when I was in Badesa. There is a project called link and they teach pregnant mothers, girls and sometimes fathers to the conference and they teach a goof thing with family planning and family

issues like respect within the family. There are people who took the training and changed the way they treat women. There was a man who went to Sodo and took family planning for men. That happened after taking that training. Training can change anything. There is no training here in this kebele. Training is given in Badesa.

5. In your community do women assume leadership roles? What types of roles? Do these roles change by social characteristics, such as (dis)ability, economic status, health status, educational level, religion, culture, geographical location, and marital status?
   - Please tell us about your own experience with assuming any leadership roles?
   - Please tell me how your leadership roles affect your PP family planning services use

Yes, there are women who lead in "idir", in market places who were assigned to do a policing job. Those women help the market place to be peaceful and they help people to live and work in a peaceful ways. There are women in other places like schools and health facilities. For example my elder sister and my fried work as school leaders here. I didn't assume any leadership roles to that level, but I worked a team leader to facilitate the link between students and teachers.

There is no association between being a leader somewhere and PP family planning. For example the idir leader is concerned with idir related issues, not with family planning. The one leading in market place are concerned with law and order there, not related with family planning.

6. In your community who decides at what age should people marry? What are the reasons for a women getting married at younger or older ages?
   - Please tell us about your own marriage experience? How old were you when you marry? What are the reasons you she get married at that age?
   - Please tell me how your age at marriage affects your PP family planning services use

Nowadays girls are not waiting too much. For example my niece married at the age of 15. There is also another one married at the same age. There are also other two girls who marry at that age. The reason for that could be lack of vision. Nowadays, there are few students who have future plan. Most of the students don't know why they are in schools even. Girls are not scared to talk to boys like our times and engage in other things then they get married. Boys marry relatively later than girls. Most of the time boys marry after 20 years and they don't have a capacity to manage. Motorcycle is also creating a problem. Girls look a boy with it and just marry without thinking anything.

I married at the age of around 27 years. Marrying at that age has no relation with using family planning.

7. What are your suggestions to improve women's roles and participation to enhance the use of PP family planning services?

Training should be given about the advantages of using family planning. In this locality there is no training being given to mothers of fathers. But, I believe training is the most important thing.

<u>Knowledge, Beliefs, and Perceptions</u>

Now we are going to discuss about the issues faced by adolescents and women in accessing PP and PA FP services in your opinion.

1. In your community what is appropriate behavior for recently delivered woman regarding use of PP and PA family planning services? How do they differ from adolescent girls and young women?

a. What is the appropriate behavior for a recently delivered mother? How do these believe influence health behavior including the PP family planning use?

Now people are getting civilized in urban areas. They think they have to use family planning after 45 days. They are not against using family planning. But the problem is with people from rural areas. They have problems with accessing and using family planning. Those who marry at younger age usually give birth immediately and then after they start using family planning.

2. What are the social beliefs and perceptions that share women's expectations and aspiration for PP family planning?

Husbands in rural areas want more and more children and they view them as resources and gifts. Husbands believe they are the only ones who can make decisions regarding this matter.

3. In your opinion who should make decisions regarding use of PP family planning services? What types of decisions do women make regarding use of healthcare including PP family planning services? What types of decisions regarding use of healthcare including PP family planning services are made jointly?

I believe husbands should be able to make decision. Unless husbands will create problems in the house. There are people I know who fall in conflict regarding FP. For example if a woman wants to use FP, the husband will say I am the one who get the money and feed my children, why are you using FP? There are such husbands. Husbands should make the decision first and encourage their wives to use FP. Due to that reason a lot of women hide using family planning. Husbands show superiority.

I believe the best thing would be making a joint decision. They should discuss and make a decision together.

Legal Rights and Status

Now we are going to discuss about the marriage related process prevalent in this community.

1. In your community are women who delivered recently legally allowed to receive PP family planning service?
   - Is these same for boys and girls, people from different age groups, education background, ethnic groups, socio economic classes, excluded groups? How are they different? Why/ Why not?

They are legally allowed to receive PP family planning. There are no law that prevent for anyone who want to use FP.

2. How do recently delivered women access PP family planning information and resource from the healthcare facilities?
   - Is these same for boys and girls, people from different age groups, education background, ethnic groups, socio economic classes, excluded groups? How are they different? Why/ Why not?
   - Do the people in your community support recently delivered women to access PP family planning infatuation and resources? How do they provide support? Who are these people? Why do they provide support?
   - Are there certain groups in your community who do not support this? Who are these people? Why don't they support?

In rural areas, health extension worker provide information and also give injection by going from house to house. In urban areas people come to the health facilities to get information and services. I never saw

here providers gather people to give training and information. If there is training regarding family planning it would be been best.

There is no group in the community who work against using PP family planning. But occasionally there are some people who say why do you use family planning, Let it be born and grow, It is not necessary to use family planning. Children grow by their lack. There are such people here and there. it is difficult to specify. They think in such ways because they need more and more children. They don't have any concern for women and the health issues.

3. Please tell me your experience with accessing healthcare information and resources including PP family planning.
   - Do you feel you have a right to get healthcare information and resources including PP family planning services? Why/why not?
   - Do you think your experience is different from other people in your community? How? Please tell me more
   - Is there legal mechanism you can follow to ensure you get quality healthcare information and resources your need? Please tell me more

Regarding my experience, after I gave birth I tried both loop and depo. It created discomfort. Again I changed to pills, but it still created discomfort. Then after, we changed our plan to natural method since we live in different places it wasn't difficult. Every time I consulted health care providers and the change the method. They finally advised me to use the one that will be inserted in to uterus, but I denied it. It is scary if they even mention this one. Even yesterday they advised me use it, but I was shocked to hear that to insert inside already wounded uterus. May be after this I have two children and after the uterus get healed, I may want to try this method.

With this we have come to an end of the discussion. Do you want to add anything before we end the discussion or if do you have any questions you want to ask?

Thank you for your time and in case you want, you can get in touch with us through the details mentioned in the consent form.

****

Summary:

The interview was conducted at the house of the recently delivered woman. She gave birth th day before I visited her house. Some people were there when I entered her house, but after I inform the purpose of my visit the left the house. Few people still come to visit her in the middle of the interview and I paused the interview until they left the house. Understanding that I am conducting an interview they didn't stay long giving me a chance discuss. Generally I believe the informant was honest and described well the topics under discussion.

Interview details

**Interviewee**	Recently deliverd woman
**Age**	28
**Interviewee code**	IDI_GA_WO_290622
**Name of interviewer**	Beimnet Desalegn
**Date**	29/06/22
**Time started**	9;00 lt
**Time finished**	9;35 lt
**Venue**	Gacheno Health center

Assets and Resources

Discussion

Now we are going to discuss about the Healthcare resources and assets available to recently delivered mothers in your area.

I: - Health services (PP and PA family planning services) availability: Can you tell me the types of healthcare facilities that provide PP family planning services in your area?

a. What types of FP services are available at the healthcare facilities?
b. Are these services equally available to boys, girls, young, old, married, unmarried, people from different geographic locations, religious or cultural background, language etc.? Please tell me more

***R: -** okay, thank you for giving this chance for me to… eee here in our community many mothers while they have menstruation … to space birth … for instance there are mothers who give birth every year if they don't use family planning methods because of this they did not care for their baby properly. Since this health center stablished and the government started to provide contraceptive method … women who were using this method is delaying their birth for three or four years. And also, community health extension workers supporting the community by providing service and information related to this service. I am also observing them educating other health related education for community specifically for mother and children.*

Yeas this is the first birth for me …. Yeah, I know a method like … which inserted in to arm, eee also drug which taken through mouth, … I know these drugs.

Thank to God here in health center … health workers serve us in good manner … only focusing in our need without any discrimination considering clients background. They didn't look at their religion, educational status, culture and …. They help anyone in need while they come to health center. I have seen this by my eye … and wetness for it they helped me a lot.

I: - Accessibility of the PP family planning services

c. Can you tell me how far the PP family planning services providing facility is located? How long does it take for you to access the facility?
d. What type of transportation means do you use to reach the facility? How easy or difficult is it to use the transportation services?
e. Is there any cost associated with accessing the PP family planning services? Please tell me more about this
f. Is the PP family planning service open at all time and is accessible with short waiting time at the healthcare facility? Are there types of FP methods of users' choice in the healthcare facility? Do healthcare providers receive and treat users with respect and dignity?
g. Are these services equally accessible to boys, girls, young, old, married, unmarried, people from different geographic locations, religious or cultural background, language etc.? Please tell me more
h. How easy could you access the PP family planning service? Do you think your access to the PP family planning services is affected because of your background? How?

R: - for me, it takes about 30 minutes to reach here health center when I come with my feet, those who didn't have money to pay for transportation come with their feet. It is difficult for those who have no money or difficulty of getting money. Those who have many can use motor cycle service … now I paid 20 birrs to come here and he is waiting me at outside now to brink me back so I pay about 50 birr including waiting time in my return. We have many problem … rather than paying for motor cycle but we pay if we have … other ways we come with our feet.

Eee the service is free of charge as I heard from others … I did not used contraceptive method before this is my first birth after now I will consider. I was living in Boditi and I have checkup at Boditi health center during my pregnancy period during that time their service were good for client. Also, I give birth here in Gacheno health center … during my stay here in Gacheno for

delivery they were also serve in good manner. I come here from Boditi because this is my first birth so in our culture a woman gives their first birth coming to here family home. I didn't pay for any service at that time and also, I have not seen anyone paying for family planning service.

Sometimes in case if the drug of users chose or absence of contraceptive at health center we did not get service here so we go to other near by health center or privet health facility. I now mothers who used contraceptive from private health clinic. Yea, health center serving community 24 hours, and health post only during government working time which is day time only.

There are health workers who were blessed by God and serving in good manner with respect and providing compassionate service for clients. They see us as their child, support and play with patient make us to feel strong… there are health workers like this. On other hand there is health workers who put pressure on client and make them feel bad and discomforted. So not all are the same…

Yea, I want to use contraceptive method in near… I am volunteer to use and I think its easy to get service at any time when I get ready.

Its my right to use contraceptive method of my chose, and the service is also equally available for all to get service and service provider should have to serve us equally… here its good they serving in good manner.

I: - Means to access the PP family planning services

i. Please tell me about your household resources and assets. Please tell me the resources and assets belong to you.
j. How is the use of the household resources and assets decided? Who makes the decision? Do you involve in the decision making? How do you think that affects your access to the healthcare services including PP family planning?
k. Please tell me about your household income. Please tell me about your own income.
l. How is the use of the household income decided? Who makes the decision? Do you involve in the decision making? How do you think that affects your access to the healthcare services including PP and PA family planning?

m. In your community how is use of PP family planning seen? Is it acceptable for women to use PP family planning? Why/why not? Please tell me more

R: - yeah, we both are working in governmental institution/office, so we have plan to space our birth also … we are living in rental house now.

Regarding decision, we put money for emergency case/use together … also we set plan together how to use our money we have. Yea I can use anytime when I need money weather he is around or not…

Using contraceptive method immediately after giving birth is good but in some rural area women were not using well… those for instance mother for whom her male partner not allowed to use… most of the time here in rural area husband's want to have many children… God cares for them why we worry about them they said. But know because of expansion of education and having educated family member at each house this kind of perception were changing. Because of this now many mothers were using contraceptive method form health facility.

I:- In your opinion what should be done to improve access to the PP family planning services for all recently delivered mothers in your community?

R: - my opinion to improve this service … health workers in health facility side … they should have to serve the community at community level going into the community. There are many women who has no information/knowledge about contraception or have no formal education because of this they are suffering with many problems. Yeah … health workers were educating the community but they have to strength and have to educate them very well about contraceptive methods which used immediately after giving birth.

Practices, Roles, and Participation

Now we are going to discuss about your women's and your role and responsibilities.

I:- Please describe to me the typical women's roles in your community?

- How are women and girls occupied over the course of 24 hours? Are there seasonal differences in how women and girls use their time?

R: - regarding women's role, in urban area their role is few while in rural area they have heavy burden … they work through a day … even because of heavy workload there are mothers whore were get seek while they use contraceptive method. I know may women who were seek using contraceptive.

I: - What kind of work do you do? Do these works change when your age or status changes (young, unmarried and married, older)?

R: - They stand up early in the morning prefer breakfast … again at lunch prefer food for their family and care their baby … if they have cattle they bring grass and food for their caw. They also pitch water …

I: - Do you have barriers on your access? What barriers? How do the barriers/restrictions influence your access to the PP family planning services and supportive social networks?

- In your community do the restrictions/barriers change by social characteristic, such as (dis)ability, economic status, health status, educational level, religion, culture, geographical location, and marital status?

R: *- most of the time women in rural area were not educated … so they did not influence their partner since most of the partner did not allow them to use contraceptive. Also, they were dependent on husband economically … she spends most of her time at home so she did not chance to get enough information about the dangerous of having frequent deliver and how to control or regulate birth.*

I: - Do you participate in any family, community, government, or other social networks organizations? Please tell me more.

- Please tell me how your participation in these organizations affect your PP family planning services use

R: - yeah, I participate in different meeting … at woreda level. I was also taken part in meeting with health professionals and health center administrating body. I got important information about health-related issue …

This meeting helped me in many way … it created good awareness about reproductive health and reproductive health problems. I also understood how to keep my family healthy and how to be healthy myself. Learned about birth controlling methods.

I: - In your community do women assume leadership roles? What types of roles? Do these roles change by social characteristics, such as (dis)ability, economic status, health status, educational level, religion, culture, geographical location, and marital status?

- Please tell us about your own experience with assuming any leadership roles?
- Please tell me how your leadership roles affect your PP family planning services use

R: - most of the time to take or assume leadership position educational status and personal ability to manage things were considered most by assigning body.

There are many women in the community leady different groups and teams like 1 to 5 team ….

I don't have any leadership role now … if I got in the future I will consider this issue specifically family planning. I will educate mothers … there are mother who understand things well even they don't have formal education.

I: - In your community who decides at what age should people marry? What are the reasons for a women getting married at younger or older ages?

- Please tell us about your own marriage experience? How old were you when you marry? What are the reasons you she get married at that age?
- Please tell me how your age at marriage affects your PP family planning services use

R: - here in our community most of the time specially now both male or female were getting marry at early age. Most of the time in 15 or 16 years. They engaged in to marriage by themselves even teachers at school or family telling them or educating them about the consequence but they did not hear. Most of the time they decide by themselves.

Now I am 25 years old, I was married 2 years back when I was 23 … I was gotten pregnant after one year of marriage. I was not used any contraceptive method. That is why if it is used before having first birth delay pregnancy or made women infertile say community member or older people. Because of this I did not use contraceptive at that time. So, I want to use contraceptive after giving my first birth … so now I have plan to use contraceptive now…

I: - What are your suggestions to improve women's roles and participation to enhance the use of PP family planning services?

R: - eee… to improve women's role health workers should support them by education and empowering to make decision by themselves… to set free or ride of social influence which is rumor about the contraceptive method. Most of the time our community is ready to accepted new thing if they got appropriate information from right person.

Knowledge, Beliefs, and Perceptions

Now we are going to discuss about the issues faced by adolescents and women in accessing PP and PA FP services in your opinion.

I: - In your community what is appropriate behavior for recently delivered woman regarding use of PP and PA family planning services? How do they differ from adolescent girls and young women?

a. What is the appropriate behavior for a recently delivered mother? How do these believe influence health behavior including the PP family planning use?

R: - yeah, many women did not want to take or use contraceptive immediately after giving birth… because of this health workers should causal them and convince them.

In our community this is seen as inappropriate by many women… because they have fear of bad consequence since this is new for us.

2. What are the social beliefs and perceptions that share women's expectations and aspiration for PP family planning?

R: -

I: - In your opinion who should make decisions regarding use of PP family planning services? What types of decisions do women make regarding use of healthcare including PP family planning services? What types of decisions regarding use of healthcare including PP family planning services are made jointly?

R: - its better if they decide together with their partner to use contraceptive … if she decide alone it may cause distrust among them … even I know family who were divorced because of this kind of issue.

Legal Rights and Status

Now we are going to discuss about the marriage related process prevalent in this community.

I: - In your community are women who delivered recently legally allowed to receive PP family planning service?

- Is these same for boys and girls, people from different age groups, education background, ethnic groups, socio economic classes, excluded groups? How are they different? Why/ Why not?

R: - there is no rule which is preventing women from using contraceptive method … if she want to use at any time.

I heard one thing recently that is Dr. Abiy has told to give more birth and not to enforce mother to use contraceptive method but I don't now detail weather women were applying it or not …

I: - How do recently delivered women access PP family planning information and resource from the healthcare facilities?

- Is these same for boys and girls, people from different age groups, education background, ethnic groups, socio economic classes, excluded groups? How are they different? Why/ Why not?

- Do the people in your community support recently delivered women to access PP family planning infatuation and resources? How do they provide support? Who are these people? Why do they provide support?
- Are there certain groups in your community who do not support this? Who are these people? Why don't they support?

R: - most of the time women get information from community health workers… they educate mother … we have community meeting monthly with health extension worker… at that time they teach mother about contraceptive methods. Many women got all information from extension workers and use contraceptive from health post.

Yeah, there are other people supporting mother to come to health post and use contraceptive like 1 to 5 team leaders encourage and assure for community health workers who is using contraceptive in their team and not.

Previously there are some people who were against use of contraceptive but now there is no anyone openly against the use of contraceptive method.

I see some woman who got pregnant while using contraceptive … we don't know what the problem has with method. Even now I know mothers who is pregnant while they are using contraceptive method.

I: - Please tell me your experience with accessing healthcare information and resources including PP family planning.

- Do you feel you have a right to get healthcare information and resources including PP family planning services? Why/why not?
- Do you think your experience is different from other people in your community? How? Please tell me more
- Is there legal mechanism you can follow to ensure you get quality healthcare information and resources your need? Please tell me more

R: - yeah, I know that I have full right to get information about the drug I have going to take because I have learned it from school. I know that I have to ask them if I have unclarity with things … but most of the time health workers did not provide sufficient information if the client

did not ask them as you know in our community there are many illiterate women. So, they did not give enough information for those mother… for instance I know my right cause I have some formal education where I got information about my right and duty. For example, when I come here now to vaccinate my child they told me to go back because the person who is working at vaccination room is not there at health center… but I know that today is Thursday and it is vaccination day … then I go to heath center head office and talked to him then he orders health worker there to give vaccination to my baby. What I want to tell here is I do this because I know my right and what to do. But may mothers do not know this is there right … specifically illiterate mothers.

Yeah, I have probability of getting information from different source and to be sure with information most of the time I see the source if the source is credible which is health professional I trust it other ways I aske or consult health workers or others who I considerbetter them me.

Okay, thank you additionally what I want to tell you is there are many think to be improved by health workers side… sometimes when we come from long distance … after then they told us there is no drug… because of this many woman were getting unplanned pregnancy. Even we are not getting drug here for other health problem after diagnosis … so this thing should be improved for the future…

With this we have come to an end of the discussion. Do you want to add anything before we end the discussion or if do you have any questions you want to ask?

Thank you for your time and in case you want, you can get in touch with us through the details mentioned in the consent form.

****

Summary:

The interview was conducted on 29/06/22 at Gachno health center. She come to the health center to vaccinate her 45-day baby and waited for health workers in the immunization room. The interview was interesting but she was disappointed with the long time she spent waiting for health workers who give immunization services when I get her. Understanding that I am conducting an interview they didn't stay long giving me a chance to discuss. As she told us she is working in a government office and explained things in a good manner and here the explanation is nice during the interview.

**Interview no**	**02**
**Interviewee code**	**IDI _KO_HU_27_7_2022**
**Sex**	**M**
**Age**	**35**
**Educational status**	**10**
**Number of children**	**6**
**Marital status**	**Married**
**Name of interviewer**	**Befekadu Bekele**
**Date**	**27/7/2022**
**Time started**	**9:30**
**Time finished**	**10:11**
**Venue**	**Sura Koyo**

Now we are going to discuss about the Healthcare resources and assets available to recently delivered mothers in your area.

I: Health services (PP and PA family planning services) availability: Can you tell me the types of healthcare facilities that provide PP family planning services in your area?

a. What types of FP services are available at the healthcare facilities?

R: The community suffered a lot in the past years. We lost the life many of mothers on the way to health facilities. That was what happened before the construction of health center here. Improvement has been made since the establishment of the health center. Availability of health workers and health extension is also one of the change. There are different activities done so far for example ,home delivery free activities have been implementing. The health extension workers are reaching the community and if it is beyond their capacity they refer to hospitals., connect with ambulance services.

I: you told us about delivery services in the health facility. Thank you for that.What type of family planning services are available? would you elaborate more?

R: Injection is given for children when they are sick. vaccination is given for children ,there are vaccinations given for children orally. In addition to this, after mothers give delivery they give Depo.There are implants "hashiyan tokettiya xaliya'' and there are oral contraceptives "mittiyo xaliya'' and the like. If mothers give birth without spacing" boli boli yelikko'',it affects mothers. Therefore these methods are given for mothers after delivery to have adequate spacing. Mothers use the services on their preference. some prefer oral contraceptives. others prefer implants for four five years. we know about these services very well because we have been using the services we know about the services given. My wife is still using the service.

I:Which method is your wife using?

R:"hashiyan tokkisiyoga'' that is ''implants'' she is still using it.

I: Are these services equally available to boys, girls, young, old, married, unmarried, people from different geographic locations, religious or cultural background, language etc.? Please tell me more

R:yes it is available for all groups .Everybody is getting and using the service. It become community practice.

Accessibility of the PP family planning services

I: Can you tell me how far the PP family planning services providing facility is located? How long does it take for you to access the facility?

-What type of transportation means do you use to reach the facility? How easy or difficult is it to use the transportation services?

-Are these services equally accessible to boys, girls, young, old, married, unmarried, people from different geographic locations, religious or cultural background, language etc.? Please tell me more

-How easy could you access the PP family planning service? Do you think your access to the PP family planning services is affected because of your background? How?

R:it is not far. It is about five minutes from the health center. We come here on foot or we can use motor bicycle some times. There is no problem with accessibility.

I: Is there any cost associated with accessing the PP family planning services? Please tell me more about this

R:There is no cost associated with family planning. It is given free of charge.

I: Is the PP family planning service open at all time and is accessible with short waiting time at the healthcare facility? Are there types of FP methods of users' choice in the healthcare facility? Do healthcare providers receive and treat users with respect and dignity? Sometimes mothers may visit the health center in the weekends, during night times.

R:It is open all the time.The health care workers are working all the time. There is no problem with health care workers. There is no rumor I heard regarding health providers related problems. Mothers get the services /F/P methods they need based on their own choice. If they want to use long acting ones they can use and the same is true for short acting ones. No one forces them to use the methods against their will. Therefore, all types are available in the health center.

I:Do healthcare providers receive and treat users with respect and dignity? Mothers from different background may visit the health center,un educated or educated may come. How do they receive and treat the mothers?

R: They serve people in a better way and they are disciplined. They treat mothers according to their behavior.

I:Are there any problems related to this? Would you tell what is really on the ground ,what did you observe?

R:sometimes there are some problems because they are human beings they are not perfect. For example sometimes there is long waiting time. They are supposed to treat people timely but they let people wait for long time until they get the services. It is good to be honest. There are members of community based health insurance. These people pay on yearly basis. If they pay for it, they should get the service they need. However, they are not getting services. We don't know whether they do it intentionally or due to shortage of drugs. They give us prescription paper to buy drugs from private pharmacies. The government has designed "mayxeema'' which means Community based health insurance in order to support the community but we question in our

mind. I’m one of the beneficiary community based health insurance. I pay for the CBHI membership , however the services is given for other people who live in other kebele who did not pay for the services.This is the problem we have observed.

I:it is good that you mentioned about community based health insurance related problems. And we are discussing about post paritum family planning methods.Are there any problems related with this?

R:Franckly speaking every body is getting on his her preferences. They are using the services ,there is no problem with this regards.

2. Means to access the PP family planning services

I: Please tell me about your household resources and assets.Please tell me the resources and assets belong to you.

-Please tell me about your household income. Please tell me about your own income.

-How is the use of the household income decided? Who makes the decision? Do you involve in the decision making? How do you think that affects your access to the healthcare services including PP and PA family planning?

R: I have cows,oxen,goats.In addition to this I have also other resources and money I have saved.

I:How is the use of the household resources and assets decided? Who makes the decision? Do you involve in the decision making? How do you think that affects your access to the healthcare services including PP family planning?

R: Because we live in the same family ,we discuss together and decide jointly. I am not the only person who make the decision. Discussion and joint decision is very important. For example if have many children because of lack of birth spacing ‘‘boli boli yelliyogan ‘’ ,this can affect our family in different ways. That is why joint decision is important. There is nothing I hide from my wife the same is true for my wife. We openly discuss and decide together.We discuss about for how long should my wife use the method.

I: what about the source of income?

R: I can get 30 thousand birr from agricultural products per year.I have different crops.

I: In your community how is use of PP family planning seen? Is it acceptable for women to use PP family planning? Why/why not? Please tell me more

R:personally ,I think it is very important to use family planning immediately after birth. I hope the community will also accept the service. we are happy if this service is given. we will support the service so as to promote because it is very important. Here we live in rural place. we have educated and uneducated people. There are different family planning methods given so far. For example there is condom as family planning method but there are some people who have no information about it. if this service is given for most of mothers will not suffer. As we now the current living condition, it is difficult to fulfill what children need because of economic problems. Therefore, I support personally if the service is given immediately after birth. I like to share what I know for others.

The community will accept the service. The main thing is the way you disseminate information. If they don't want to accept we should tell them until they accept. The main thing is the way we convince or the way we persuade. It is obvious that there are different types of people. There are people who can easily accept and who don't want to accept. Their educational status is not the same. Those who used the service can share for others who did not use the service.

I: In your opinion what should be done to improve access to the PP family planning services for all recently delivered mothers in your community?

R: to improve the service, the health workers are expected to go to the grass root level teach the community ,arrange meeting places, women's meeting, because this is more of something related with mothers.

<u>Practices, Roles, andParticipation</u>

Now we are going to discuss about your women's and your role and responsibilities.

I: Please describe to me the typical women's roles in your community?

I: How are women and girls occupied over the course of 24 hours?Are there seasonal differences in how women and girls use their time? What kind of work do you do? Do these works change when your age or status changes (young, unmarried and married, older)?

R: we are engaged with harvesting crops ,intercropping and other agricultural activities.

I: Do you have barriers on your access? What barriers? How do the barriers/restrictions influence your access to the PP family planning services and supportive social networks?

- In your community do the restrictions/barriers change by social characteristic, such as (dis)ability, economic status, health status, educational level, religion, culture, geographical location, and marital status?

R: Mothers should get enough food while they are using the service "family planning''. They should get milk. Unless they get milk and milk products the family planning they use can affect their health. The community also think that way. Therefore, absence of milk products can be a barrier for family planning.

I: you mean poor nutrition can be a barrier for family planning?

R: Yes,people have such a perceptions. People have such questions when they think about family planning.They associate with nutrition.

I: How do you see such thoughts? is something acceptable?

R: This is not good idea. They should use the service regardless of different barriers because of unintended pregnancies and delivery, "boli boli yeluwa'' is common. Mothers give birth while their preceding baby is not yet matured. This affects the economy and other things as well. People should positively think for their family. Starting from myself I should think in this way. Therefore immediate use of family planning should be strengthened.

I:what other barriers do you know? regarding post partum family planning and post abortion family planning? Culture,religion?

R: There is no other barrier I know.

I: Do you participate in any family, community, government, or other social networks organizations? Please tell me more.

R: we haven't participated in any of meetings, I did not heard about immediate post paritum family planning.

I: Please tell me how your participation in these organizations affect your PP family planning services use

R:this is very important because when people get information ,they will practice it or change into action.

I: In your community do women assume leadership roles? What types of roles? Do these roles change by social characteristics, such as (dis)ability, economic status, health status, educational level, religion, culture, geographical location, and marital status?

- Please tell us about your own experience with assuming any leadership roles?

R: Some people are negligent due to lack of awareness and others are very serious. There are educated and uneducated ones. those who are using and not using. There are also people who have no idea, awareness about the issues (PPFP).

I: Please tell me how your leadership roles affect your PP family planning services use.

R:women participate in different social organizations, they participate in awareness creation sessions,they play their role in every place eg, during coffee ceremony the discuss about different issues and give awareness for the community. So they have many roles.their participation helps to prevent

" boli boli yelwa'' or unplanned pregnancy and birth without spacing because this can affect both the baby and mother as well.

I: how can this "boli boli yelwa'' can affect mother and her baby?

R: If the mother is from poor economy class, and if she delivers without spacing ,this can affect her health.When a mother delivers many of her organs are affected. therefore people having less economy are more likely to be affected than others having good economy.

I: In your community who decides at what age should people marry? What are the reasons for a women getting married at younger or older ages?

- Please tell us about your own marriage experience? How old were you when you marry? What are the reasons you she get married at that age?

R: in my perception it is good to get married when they are at the age of 20 or more.If they get married before twenty ,most of the time they face different problems.For example they can be exposed to fistula.Early marriage can lead to fistula and other problems as well.Therefore we teach our community about this thing. There is non-governmental organization called “Abren Integibir” which is working on this area.

I: what does this organization do?

R:It works on how to prevent fistula and other related problems.This is their focus area and we are working to prevent early marriage, child trafficking with with “Abren INtegibir”.

I: I: In your community who decides at what age should people marry? Would you elaborate it please?

R: We can decide because they are our children ,we can decide on this matter and they should also og with our decision.

I: Is there different decision made by adolescents fromwhat you told us?

R: somtimes they decide by themselves.

I:what pushes them to get married at their earlier age?

R:The reason could be due to peer pressure, ‘dafaa’ or rape is being practiced…laughing.

I:is it ‘’rape’’practiced currently?

R:there is such a practice but it not disclosed, it is done in secret. A raped girl will agree with her husband and continue her life. when she was asked why she got married she will say ‘‘I got married by my will and I did it voluntarily’’ .Therefore nobody can ask why this is happened once she agreed to live with her husband.

I: what makes you laugh?is any thing you want to share?

R: of course we observed such things in the community. For example a father may accuse and report the rape case of his daughter to policemen but his daughter deny that it is not rape and she would say “I got married voluntarily’’ and tell her father to stop accusing her husband.

I: Please tell me how your age at marriage affects your PP family planning services use.

R:If the one who is getting is educated one she can use PP family planning. Those who have better knowledge will use and those who don’t have knowledge will not practice. Therefore, information should be disseminated in different places. Schools should play their role. There should also be information dissemination in community gatherings, meetings. If this the case there is no reason for her to oppose or not to use the service.

I: What are your suggestions to improve women’s roles and participation to enhance the use of PPfamily planning services?

R: women should visit health centers and ask available options to use family planning. it could be oral or implants they have to come and decide which method to use. They should use it timely. In addition to this I have mentioned earlier they should disseminate information during community gatherings. specially there should be separate women’s meeting because this is one of their concern.

I: why is it important to conduct meeting with women only?

R:There reason is that most of the problem happens among women,this is their concern.

I: In your community what is appropriate behavior for recently delivered woman regarding use of PP and PA family planning services? How do they differ from adolescent girls and young women?

a. What is the appropriate behavior for a recently delivered mother? How do these believe influence health behavior including the PP family planning use?

R: using family planning immediately after birth is good thing and mothers also accept the idea. Uneducated husbands can make sexual intercourse with in this times, and this can affect her health and use of PPFP is helpful to prevent unintended pregnancy.

I: What are the social beliefs and perceptions that share women's expectations and aspiration for PP family planning?

In your opinion who should make decisions regarding use of PP family planning services? What types of decisions do women make regarding use of healthcare including PP family planning services? What types of decisions regarding use of healthcare including PP family planning services are made jointly?

R: there could be people who support and also oppose the use of PPPFP. All people are not the same. Their thinking, behavior is different. some people say "No one can interfere, I am the one to care or narture my children '' .However, due to the current living condition or poor economy it is better "Ararko mewuled'' is good. There should be wide/adequate spacing. Information should be given not only for mothers because this should be the concern of both husbands and wife. Husbands should get information. We don't have to ignore husbands while delivering message. There needs to be discussion. There shouldn't be independent decision making regarding the use of family planning.if a wife decides independently this may create conflict between couples.a husband may want to have children but if his wife doesnot want and use family planning this may create 'Tseb' or conflict. There should be consensus between the couples.

Legal Rights and Status

Now we are going to discuss about the marriage related process prevalent in this community.

I: In your community are women who delivered recently legally allowed to receive PP family planning service?

- Is these same for boys and girls, people from different age groups, education background, ethnic groups, socio economic classes, excluded groups? How are they different? Why/ Why not?

R:Yes it is legally allowed. because it would not be recommended and promoted if the services is not allowed locally.It is already being promoted because it is legally allowed.

I: How do recently delivered women access PP family planning information and resource from the healthcare facilities?

- Is these same for boys and girls, people from different age groups, education background, ethnic groups, socio economic classes, excluded groups? How are they different? Why/ Why not?
- Do the people in your community support recently delivered women to access PP family planning infatuation and resources? How do they provide support? Who are these people? Why do they provide support?
- Are there certain groups in your community who do not support this? Who are these people? Why don't they support?

R: They get information from health post, health center, private clinics. Specially those who cannot easily access the health center can get easily from their nearby private clinics. All group of people get information however, utilization can be different .Most people support the use of PPFP.Mostly educated people are using the service. As I have said earlier most of the time it is a mother who will face the challenges unless she use the services.

Please tell me your experience with accessing healthcare information and resources includingPP family planning.

- Do you feel you have a right to get healthcare information and resources including PP family planning services? Why/why not?
- Do you think your experience is different from other people in your community? How? Please tell me more

R: Yes they have right to get information.

I: Is there legal mechanism you can follow to ensure you get quality healthcare information and resources your need? Please tell me more

R:Yes there is legal mechanism where we can follow. We can ask head of health centers.

I: With this we have come to an end of the discussion. Do you want to add anything before we end the discussion or if do you have any questions you want to ask?

R: In addition to what I have said people should know the benefit of using the services, and there should be information dissemination at kebele level, one to five networks, women development armies.

I: Thank you for your time and in case you want, you can get in touch with us through the details mentioned in the consent form.

R:Thank you.

****

Summary

This is in-depth interview conducted husband of recently delivered woman in sura koyo

- ✓ Have awareness about majority of family planning methods available in the health facility
- ✓ The health care workers respect and treat women as they come to the health center to get the service but still there is long waiting time.
- ✓ There is lack of drugs and the patients are sent outside to private clinincs.
- ✓ Open discussion and joint decision about PPFP and PAFP is needed
- ✓ Proper health information dissemination helps people/mothers to convince /persuade mothers
- ✓ Women associate nutrition with the use family planning. If there is no enough food in their home they prefer use family planning methods.

- ✓ Decision to use family planning should be made joint decision in order to avoid conflict
- ✓ Rape is still practiced in secret
- ✓ Better to consider husbands while educating mothers about PPFP andPAFP.
- ✓ There are different audiences in terms of utilization PPFP.There are those who easily use and oppose or resist.
- ✓ Schools, existing networks, like one to five, and one to thirty was mentioned as good platforms for intervention.

Interviewee code	IDI_KO_ MF_ 30-6-2022
Sex	Female
Age	27
Marital status	Married
Service year	Five years
Job title	MCH focal person
Name of interviewer	Befekadu Bekele
Date	30/06/22
Time started	5:00
Time finished	5:57
Venue	Koyo health center

Now we are going to discuss about the Healthcare resources and assets available for provision of PP and PA family planning services in your area.

I: Health services (PP and PA family planning services) availability: Can you tell me the types of healthcare facilities that provide PP and PA family planning services in your area?

a. What types of FP services are available at the healthcare facilities?
b. Are these services equally available to boys, girls, young, old, married, unmarried, people from different geographic locations, religious or cultural background, language etc.? Please tell me more

R: Both long acting and short acting family planning services are given in our health facility. Recently we started to give immediate post partum and post abortion family planning services. we give these services for all groups you have mentioned.

I: Accessibility of the PP and PA family planning services

-Can you tell me how far the PP and PA family planning services providing facilities are located? How long does it take for women to access the facilities?

-What type of transportation means do they use to reach the facilities? How easy or difficult is it to use the transportation services?

-Is there any cost associated with accessing the PP and PA family planning services? Please tell me more about this

-Is the PP and PA family planning service is accessible at all time at the healthcare facilities? Are there types of FP methods of users' choice in the healthcare facilities?

-Are these services equally accessible to boys, girls, young, old, married, unmarried, people from different geographic locations, religious or cultural background, language etc.? Please tell me more

-How easy could they access the PP and PA family planning services? Do you think that their access to the PP and PA family planning services are affected because of their background? How?

R: we started to give the services recently after we received training. The performance is good as compared to the previous years because of the training. More than half of health workers have received training on family planning. So we are giving the services and working to ensure accessibility. The service is given for mothers, girls who came to the health center as well as in the community level. Concerning the distance, most mothers come from far places. For example, there are mothers from 'Adedawe, kuxo' and Tora wulisho which are far from the health center. Sometimes they use motor bicycle to come here, some people come on foot, ambulance service is also given for mothers.

I: Is there any cost associated with accessing the PP and PA family planning services? Please tell me more about this

R: As per the cost associated with accessing PP and PA family planning services, there is no any cost related to these services. These services are given for free of charge.

I: Are these services equally accessible to boys, girls, young, old, married, unmarried, people from different geographic locations, religious or cultural background, language etc.? Please tell me more

R:All of the services are equally accessible to all groups. But there are some religious groups which don't allow their members to use family planning. If I am not mistaken Adventist religion don't allow their members to use family planning because it seen as sin. This can prevent people from accessing the service. There could also be people who don't use the service due to culture.

I: Is the PP and PA family planning service is accessible at all time at the healthcare facilities? Are there types of FP methods of users' choice in the healthcare facilities?

R: We give the services based on user's choice. First of all we counsel them and tell both advantage and also the side effects. Then we give the service based on her preference. There is no room where we force mothers to use any of the methods. Implanon is mostly the preferred by mothers currently as compared to the other methods. previously they prefer the short acting ones.

Means to access the PP and PA family planning services

I: Please tell me about your facilities' resources and assets. Please tell me the resources and assets belong to these health facilities to provide PP and PA FP service.

-How is the use of the facility/Woreda health office resources and assets decided? Who makes the decision? Do you involve in the decision making? How do you think that affects access to the healthcare services including PP and PA family planning by women?
-Please tell me about income of in this Woreda. Please tell me about your woredas income.
-How is the use of the woreda's income decided? Who makes the decision? Do you involve in the decision making? How do you think that affects their access to the healthcare services including PP and PA family planning?

I: In your woreda how is use of PP and PA family planning seen? Is it acceptable for women to use PP and PA family planning? Why/why not? Please tell me more (what about boys and girls)

R: We have resources. we have adequate family planning methods like Depo, IUCD, Implanon, jaddle, COC, post pills. However, we have lack of rooms to give the services. we don't have adequate rooms, As you see we give ANC and family planning in this narrow room. So we have scarcity of rooms. In addition to this we could be able to see our scarcities/gaps after we took trainings. In addition to this we have shortage of cannula   for safe abortion services.

When we face shortage of the necessary resources, we request all the necessary resources, I have the role and I participate in this matter since I am the coordinator of MCH services. Then the

issues will be raised discussed within management bodies, we decide together in order to avail all the necessary resources.

I :How do you think that affects their access to the healthcare services including PP and PA family planning?

R: Our participation in decision making is crucial because we are the one who provide the services and responsible for that. We know the gaps in detail than other bodies. It has effect in improving the services.

I : Please tell me about income of in this Woreda. Please tell me about your woredas income

R: From my observation as well as experience I don't think that the health center has enough resources. As a result many of activities are lagging behind or less performing. There is no problem with how the income and budget management because everything is done transparently because there are management bodies.

I: In your woreda how is use of PP and PA family planning seen? Is it acceptable for women to use PP and PA family planning? Why/why not? Please tell me more (what about boys and girls)

R: As I have said earlier we started to give this services recently. We started to give immediate PP and PA family planning very recently since we received training from Engender health ,it is about four months ago. We used to give the services even before the training but it was not supported with training.

I : How do women in your society see PP and PA family planning services?it could before you receive or after you training?

R: This can differ from person to person. There are Some mothers who are ready to accept immediately after birth and there are also mothers who resist, who are asking us usually what is the significance of using PP and PA family planning immediately after birth. I can say not all mothers accept and willing to accept because there are some mothers who are not willing to accept. The one who resist say :‘‘yet ihedalewu ,what benefit can I get from it ,yidersal mechem

biwesdim chigir yelwum'', which is to mean there is no problem if I start to use any time.This could be due to attitude problem.

I:what kind of attitude problem? is there in the community regarding these services?

R: Since the service is given immediately after birth primi mothers usually say ‘‘ I will not stay with my husband so there is no sexual intercourse because I will stay with my family'' so there is no problem if I start to use the services when I come back to my husband. This is what I have faced most of the time.

I: what about others ?other than primi mothers?

R: They perceive it as something rude because it is given privately.

Now we are going to discuss about your role and responsibilities.

I: Please describe to me the typical Woreda health office officials/experts roles in your Woreda/community? How is Woreda health office officials occupied over the course of the day? Are there seasonal differences (campaign/political assignment) in how you use time?

R: There are routine activities that I am engaged with. Family planning services, delivery service, abortion services, immunization services. We give all MCH related services. I'm also engaged with data, follow registration books, report to the concerned bodies on monthly basis. Since it is mandatory for me to do these activities whether there is work load or campaign, I play my role in doing all these activities because this is my responsibility.

I: As Woreda health office official, what kind of work do you do? Do these works different when facility status changes (Rural, urban and geographical locations)?

I: Do Woreda health office officials like you have restrictions/barriers on the accessibility? What restrictions/barriers? How do the restrictions/barriers influence the access of the PP and PA family planning services and supportive social networks?

a. In your Woreda do the restriction/barriers change by social characteristic, such as (dis)ability, economic status, health status, educational level, religion, culture, geographical location, and maritalstatus?

R: There are some barriers for mothers and adolescents not to use the services One of this could be religion as I have tried to mention. Using family planning can be seen as sin in some religions. Culturally *‘‘having children is seen as blessing from God so we don’t need to limit ourselves, it is not good to use family planning* ’’ they say. Others say : “*I want to have children since it is gift from God’’ , egziabiher yisexal egizabiher yasadigal*’’. ‘‘*It is God who gives children and he provides what we need”* they say. So such attitude is common barrier for post partum family planning. I did not face restrictions by disability. I have seen that there disabled people who voluntarily use the services. There are people who come from far places but they can access the services from health post.

I: Do you participate in any meetings, workshops, trainings, pregnant women conference, family, community, government, or other social networks organizations? Please tell me more. Please tell me how your participation in these organizations affect the PP and PA family planning services provision

R: I have participated in trainings related to these services. Participating in training helped me a lot to work my activities ,it added skills and theoretical knowledge. The training also helped me to fill my skill gaps,so it helped me to have skills, good experience. Especially it helped us to have good performance on pp and PA family planning services, give attention to these services. on the other way It helped mothers to use family planning, therefore ,the number of mothers using post partum family planning and adherence of the service has increased.

I: In your Woreda do women assume leadership roles? What types of roles? Do these roles change by social characteristics, such as (dis)ability, economic status, health status, educational level, religion, culture, geographical location, and marital status? Please tell us about your own experience with assuming any leadership roles? Please tell me how your leadership roles affect the PP and PA family planning services provision

R: Women can play great role when they are assigned in a leadership position. particularly women are more concerned about family planning services than males. They are effective in terms of organizing, putting plans into actions. The services should be efficiently provided. Women in any leadership position can play role because they are part of the problem and very close to the service and they know the problems. Therefore, engaging women in such position will improve the service. Coming to personal life starting from family I have several roles, I am coordinator of MCH case team and I have other responsibilities too. If women are assigned in PP and PA family planning related services they know more about the services and activities than other bodies, it is easy for us. As I have said earlier it will help to ensure accessibility of the services.

I: In your Woreda/community who decides at what age people marry? What are the reasons for getting married at younger or older ages in this Woreda? Please tell me how their age at marriage affects their PP and PA family planning services use

R: Mostly females marry at the age of 18 years and males also started to marry at this age. The main reason to get married at this early stage is unemployment because this expose them to get married. The other possible reason could be their age, adolescent age by itself pushes them to get married. The other thing is that the society itself because the society pushes females to get married at their early age. ''*Lij belijinet newu selizih agbiy* '' you should get marry and give birth at younger age they say. These factors can push them to get married at their earlier age.

I: Please tell me how their age at marriage affects their PP and PA family planning services use

R:Their age affects their marriage because they are not matured. They are not in a position to understand and accept things that we recommend. They could be able to bear children but they are not in a position to decide, their decision making capacity is very low at this stage. This can affect PP and PA family planning services.

I: What are your suggestions to improve women's roles and participation to enhance the use of PP and PA family planning services?

R: There are many things to be done from health center side. One of this is awareness creation. Health care workers have to reach the community and teach the community about this service.

There is women forum which is done in each kebele, so each health care workers should go to the community and play their role and create awareness so that this can help the community utilize the service. In addition to this there are barriers related with religion so, health care workers should go and teach religious leaders because we can change the community behavior by educating them and the community should be willing to be changed and use the service. Engaging women can play great role because we can use them as role models to teach others.

Now we are going to discuss about the issues faced by women in accessing PP and PA family Planning services in your opinion.

I: In your Woreda what is appropriate behavior for a woman after delivery and abortion regarding the use of PP and PA family planning? How do they differ from adolescent girls and young women? How do these believe influence health behavior including the PP and PA family planning use?

R: They don't want to use the service. They come to us secretly whenever they want to receive the service. Married ones are somehow better than the unmarried ones. Unmarried ones are afraid of abortion service because it seen as a SIN by the community. It is like killing people. Regarding post abortion family planning services there are attitude related problems within the society. Such negative attitude can influence PP and PA services because they don't use the services voluntarily. When their husbands are not willing to use the service this things complicate all things. Her capacity to make decision immediately after birth is very low. They usually want to use the service after they discuss with their husbands.

I: In your opinion who should make decisions regarding use of PP and PA family planning services? What types of decisions do women make regarding use of healthcare including PP and PA family planning services? What types of decisions regarding use of healthcare including PP and PA family planning services are made jointly?

R: both of the couples should take part in decision making. Even though the service us given for a woman, her husband is also benefited. Because it is for common goal both husband and wife should decide together. Husbands can play great role and should have positive attitude towards the service. This will help mothers to come to decide willing fully. Women should also be

engaged in decision making process.When deciding jointly they can discuss about the benefit of the family planning methods, they can discuss for how long they should use the service, they can also discuss about the number of children they should have, and also they can discuss and decide the type of family planning to use.

I: In your community are women who delivered recently legally allowed to receive PP and PA family planning service?

- Is these same for boys and girls, people from different age groups, education background, ethnic groups, socio economic classes, excluded groups? How are they different? Why/ Why not?

R: Yes they are legally allowed to use the PP and PA family planning services. There is no restriction with this regards. There is no excluded groups. It is equally given for all groups.

I: How do recently delivered/aborted women access PP and PA family planning information and resource from the healthcare facilities?

- Is these same for boys and girls, people from different age groups, education background, ethnic groups, socio economic classes, excluded groups? How are they different? Why/ Why not?
- Do the people in your community support recently delivered/aborted women to access PP and PA family planning information and resources? How do they provide support? Who are these people? Why do they provide support?
- Are there certain groups in your community who do not support this? Who are these people? Why don't they support?

R: Because the service is given at the health center they can access information from health care providers during counseling. Therefore, mothers will get information that will help them to make decision. In addition to this we have community engagement. We do all the activities at community level. There is women forum done every month, they will get information from monthly forum. During this meeting women choose they type of family planning they want to use, and make agreement and we let them bring their written decision when they come for

delivery service. Based on this we give them the service on their preference. Because they get information from different places that will not be new when they come to us to get the service.

Mainly the health extension workers support the community to get the services, Health development armies also support them to use. There is also one to five net work. Those model mothers also support and encourage the other community members to use the services. There are some religious groups which don't support their members to use family planning as I have mentioned earlier because it is seen as SIN. So the members of that religion may not use family planning.

I:Do you feel they have a right to get healthcare information and resources including PP family planning services? Why/why not?

- Do you think women's experience is different from other people in your community? How? Please tell me more
- Is there legal mechanism you can follow to ensure they get quality healthcare information and resources they need? Please tell me more

R: Yes, they have the right to get health care information and resources. All of them have the right to use the service. They have right to use service based on their preference.

I: Thank you very much. If you have anything to say or add lastly.

R: It is good to conduct research because it will help to identify gaps will help to improve service. There should be many things to be done at community level. The support from concerned bodies should be maintained in order to ensure accessibility of the service.

With this we have come to an end of the discussion. Do you want to add anything before we end the discussion or if you have any questions you want to ask?

I: Thank you for your time and in case you want, you can get in touch with us through the details mentioned in the consent form.

R:Thank you.

Summary

This is interview conducted with MCH focal person in koyo health center on 30/6/2022

- ✓ For some mothers religion was found to be one of the barrier for family planning. Use of family planning is seen as sin. Faith based education/intervention is recommended to enhance the service
- ✓ There is lack of rooms to give the PPFP and PAFP services, lack of resources
- ✓ There are extremes in the use se of immediate PPFP and PAF, those who are very happy and those resist.
- ✓ Prmi mothers want to use family planning when they come back home, because primi mother is supposed to stay with her family for few months.
- ✓ Culture is also one barrier for mothers not to use the service because having many children are seen as gifts from God they don’t want to limit the number of children
- ✓ The role of women perceived to be greater than males.
- ✓ Receiving training improved the performance of health care workers on immediate post partum and post abortion services.
- ✓ Unemployment , family or community influence were factors which push adolescents to get married at their early age.
- ✓ Existing networks like one to five, one to 30 believed to enhance PPFP and PAFP

IDI-RHB-RE-17082022

Background information

I: Please tell me about your age, level of education, role and experience?

R: I am 35 years old and I have attained master or postgraduate degree. I am family planning expert at regional level and worked for 14 years in different level of health system.

Assets and Resources

I: Health services (PP and PA family planning services) availability: Can you tell me the types of healthcare facilities that provide PP and PA family planning services in your area?

a. What types of FP services are available at the healthcare facilities?
b. Are these services equally available to boys, girls, young, old, married, unmarried, people from different geographic locations, religious or cultural background, language etc.? Please tell me more

R: Normally to say whether there is service or not, there should be data for number of facilities that give the service and number of facilities that doesn't give service. For instance, if we look in to Wolaita Zone data, what I can remember is that, with in last one year from existing health facilities, about 33 health facilities didn't started to provide service. I can say about half from existing facilities. Therefore, about only half facilities were providing the service. Regarding service utilization, what I can remember is, we can see Woliata data, from the women who delivered at health facility, nearly 10%, even not approached to 10%, it around that, which indicates it is still at beginning level, these show that the program persons as well as health professionals didn't recognized separately the impact of the program and it could also be due to lack of capacity to recognize, nearly 33 facilities are not providing service and the remaining are providing the service. It is about half of the facility. The service uptake is also as I said before. It is about 10% from the women who delivered in the health facility. The service access seems like these. Normally what we promote is, in all facilities there are FP options, there is POP, oral contraceptive, implants, IPIUD, permanent especially tubal ligation, the IPIUD and permanent methods require especial training and the above that I mentioned also require especial training and at this point, with the evidence we have currently, in all facilities there are service providers who can gave implants at this time. In all health facilities, there are providers who can counsel and Provide POP or oral contraceptives. These methods can be given by all facilities. Mostly implants are provided as evidenced by data. Most of postpartum family planning users are using implants. Which is followed by IPIUD and OC.

I: Accessibility of the PP and PA family planning services

c. Can you tell me how far the PP and PA family planning services providing facilities are located? How long does it take for women to access the facilities?
d. What type of transportation means do they use to reach the facilities? How easy or difficult is it to use the transportation services?
e. Is there any cost associated with accessing the PP and PA family planning services? Please tell me more about this
f. Is the PP and PA family planning service is accessible at all time at the healthcare facilities? Are there types of FP methods of users' choice in the healthcare facilities?
g. Are these services equally accessible to boys, girls, young, old, married, unmarried, people from different geographic locations, religious or cultural background, language etc.? Please tell me more
h. How easy could they access the PP and PA family planning services? Do you think that their access to the PP and PA family planning services are affected because of their background? How?

R: Most of the time when we observe, those who use post abortion or safe abortion service, one of the component for post abortion service is family planning. It should be done with high emphasis. It has been recommended for professionals and provided by that extent. Normally it is accessed to them. But related to awareness, especially adolescent girls are not accessing service. It could be due to providers level of awareness as well as girls. It could also their (adolescents) health literacy level. The service uptake is also not comparable with other age groups especially regarding FP service. For some extent the level of utilization is low as per my observation. Especially the adolescent group health literary level is not equal with others, the age is lower from other groups to get service from service outlets, they need privacy and confidentiality, the service set up and area should be comfortable and suitable for them, these are one of hindering factors, another factor is the health provider level of awareness, if one young girl come and ask family planning or abortion service, the providers attitude is not similar with the attitude for other age group. By all means the main thing is not having suitable and comfortable service set up for adolescents. The activities that has be done at community level are not focusing them. It could be due to related with that. The services are available but it is not tailored to them. I think religion couldn't be a factor for FP uptake. But geography could be a factor. The culture rather than religion. The myths or misconception and things like that may hinder. There are occasions that we meet with religious leaders, we let them talk all things but there is not that much things related with religion. We discuss with protestant or Muslim and it is not related that much with religion but there are issues related with geographical location and population size. There are thoughts which are dominantly seen not at centrally areas but around remote zones and periphery woredas. Those areas where there are pastoralists and agrarians live.

I: Means to access the PP and PA family planning services

i. Please tell me about your facilities' resources and assets. Please tell me the resources and assets belong to these health facilities to provide PP and PA FP service.
j. How is the use of the facility/Region health office resources and assets decided? Who makes the decision? Do you involve in the decision making? How do you think that affects access to the healthcare services including PP and PA family planning by women?
k. Please tell me about income of in this Region. Please tell me about your woredas income.
l. How is the use of the region income decided? Who makes the decision? Do you involve in the decision making? How do you think that affects their access to the healthcare services including PP and PA family planning?
m. In your region how is use of PP and PA family planning seen? Is it acceptable for women to use PP and PA family planning? Why/why not? Please tell me more (what about boys and girls)

R: When we look in to overall FP service, regarding PPFP there is big variation among urban and rural areas. There is disparity between urban-rural, Educated or uneducated, when we look in to overall family planning, the difference is not between urban and rural, it is among pastoralists or living condition, educated and uneducated, when we look in to urban-rural, there are service at health post level. It doesn't require cost even for transportation, the service is available in each health post. The health professionals who work there provide implants or OC, May they expected to come to Health center if they need IUCD, we didn't seen that much difference between urban and rural area. The service uptake is nearly similar and even it is better in rural area. There is variation in method mix. If we take rural areas, they use mostly injectable, almost all. If you see urban data, it is predominantly implants. There is some level of variation in method mix. But there is no that much difference in overall service uptake. The observed difference in method mix seems to be due to awareness. It could be due to the providers level of understanding and client awareness. To say it is due to availability, the implants are available at health post level. If you go to every corner, the implants are available. Though

there is stock out of supplies, it is based on their request. Their request is pulling factor, It helps to get supplies. It is mainly due to poor counselling and lack of client awareness or attitude. Regarding the cost, we know that the service is free of charge but the government buy from abroad and provide free of charge. But sometimes, there could be shortage of consumable materials and they may buy them. When we are saying free service, it doesn't mean that the supplies come free of charge. But the government arranged it to be provided free of charge. It is provided in all places free of charge. Except private facilities where they have been franchised with minimum cost via DKT, Meristops,. They charge for service and some amount for commodity. Except them the public facilities gave free of charge. My concern on this is that if they face shortage of consumable materials, I doubt at that time, otherwise the service is free of charge. They can get the service at any time. For instance, regarding postpartum FP, they will get the service at any time after getting the delivery service. Because delivery service is available at any time. Immediately after getting delivery services with in 48 hours, especially it is recommended to be given within 10 minutes. There is no effect of time barrier. The good thing about Integration is, it will decrease resource needed. For instance, when delivery service given, one can give postpartum FP in the same delivery couch. It doesn't require additional resource, the set up prepared for delivery is enough to provide PPFP service. Live alone other thing, the register book even have one column leveled PPFP to register the service. If start CAC or post abortion service, you can give PAFP on the same set up that you gave PA/CAC service. When you integrate FP with ART, it requires nothing additionally. You will give the service with existing resources. May it require additional human power. Those things will be arranged by leaders at facility level. Another thing is, they may need training and it also demands resource. The peoples at regional or Zonal level or woreda level will arrange that. The things related to resources within facilities and building are, one thing is design issue. This will be done at national or regional level. The current or short term problems will be solved by facility head and woreda personnel there. Things like room arrangement and availing suitable rooms. The design issue is at national level. For instance, CAC room arrangement is facility heads role by selecting appropriate room which is suitable for abortion service from existing rooms. It is by facility head, woreda personnel and by nearby people in that structure. Currently, the region can't take initiatives related to existing context. At regional level there are about 700 or 800 facilities. What should region take is that, at time of construction of buildings, what should be taken in to consideration is that does the rooms enough for the services, Are they suitable to provide the services. Because, if the buildings are constructed these year, it should take into consideration of services for the coming 10 and 20 years. But most of the building didn't take in to account these things. It should take in to consideration the coming new initiatives in the future. When buildings are constructed, the design will be presented to us for comment and then you will put your program perspective. We will not intervene on individual facilities. There is budget line for FP, though it is not adequate, the budget planned at regional level for advocacy for higher officials and awareness creation for program managers. But the budget is not balanced with the service required. We allocate budget every year. The PAFP goes generally with post abortion care. When we check service availability, only about 46% facilities providing the service. We could able to expand the service. It is related to budget. Because we need to capacitate the providers and avail materials required. For this thing, adequate budget needed. You can't talk about PAFP with out ensuring availability of CAC service. The same is true for PPFP. There is budget constraints to capacitate staff and fulfill materials required. As region we couldn't moved both programs as we planned because of budget shortage. Regarding demand creation, activities like social mobilization, working with partners and media, health extension workers didn't done that much. We addressing only those who visit facility for ANC and delivery service. If they know availability of service before they get pregnant , there might be no challenge. The demand creation part , especially activities that need to be done at community level was not done very well. One reason is budget constraint. Activities that need to reach HEW and medias, not done, we focused only those who come to facilities and our denominator is also

those who come to facility and they are our target group. As you mentioned in future we need to create awareness and activities that increase demand should be done. The budget is a constraint that prevent you from looking broader scope. The women as well as young women accept the service. You can look in to EDHS data that the unmet need is high and even higher for mothers during postpartum period which is about 74%. If you ask them randomly they don't want to give birth in the coming two to three years. But currently that women not getting the service. Overall they didn't accessed services very well. If they counselled appropriately and service availed to them, they will utilize it. Our counselling approach and not availing service affected the uptake, otherwise they use it very well. How you know that is when you observe last year performance it was 6% and this year it is 10%. This indicate that the service uptake was increasing, the uptake will increase as we avail services close to them. The use and there is big demand. It is similar to PAFP, sometimes we measure service integration just looking in to data. How many clients received post abortion service and how many used FP services. It around 50 to 60 % in some facilities and levels. If there is good counselling, it will reach up to 80%. Those women who received abortion service they want to delay pregnancy for while but they are currently not using it. If we availed the services and counselled them appropriately, there is huge demand and the uptake will increase.

I: In your opinion what should be done to improve access to the PP and PA family planning services for all women in your community? You mentioned appropriate counselling and demand creation previously, what you went to add?

R: The first thing is that when women come for ANC service, they should be counselled. She should be counselled early and not during delivery at couch. She should decide and ready which method she will take later. She will say " I will use this method immediately after giving birth". Her decision should be stick to her medical records. If she say "I will take IUCD after delivery", her choice to take IUCD should be indicated in her medical record. It would be new to her immediately after delivery which method she going to take. The provider look in to her medical record and say " you have decided to take this method and shall we give for you?" therefore, counselling should be this much strong. She should decide early. There should be awareness creation for program managers. They should track the service uptake. If the program manager at zone or woreda level aware of the program and track it, he/she will Identify facility level gaps and intervene to that extent. If program mangers track, support and review continuously, the service will stand and they will do local interventions, the program managers should have adequate level of awareness. When I am saying program managers, I mean facility heads, woreda and zone level program coordinators, starting from hospital and health center heads, the program will exist if they follow it. To that extent they should incorporate in to PMT, Supportive supervision. Another thing is supply issue. The supplies should be availed. It is meaningless if the mother demand it and the provider is ready to give, and if the supply is not available. The supply issue should be strengthened. The final one is demand creation. There are mothers who not come for ANC service and the community mobilizations at community level through HEWs should be done. There are also women, whose husbands not allow then to use the FP service. The activities that need to be done at community level and via media should be strengthened. If these things done, we can see better progress. To prevent stock out of supplies, they should request appropriately on time. RRF should be strengthened very well. There is also national stock issue. Sometimes, there will be stock out at national level. It is purchased by money and there could be shortage of money or dollars, or currency exchange issues as you know our countries current conditions. Sometime the dollar shoots and it will be so expensive than we planned to buy and the agreement may be cancelled. At that time there will be national stock out. To prevent this, there should be advocacy works and allocating adequate budget. The third thing is distribution related issue. Distribution will be done by EPSA, there are regional hubs for EPSA. They should discharge their responsibility regarding discharge. These three bodies are responsible for stock out. The facilities should submit RRF report

timely and with good quality. EPSA should strictly follow distribution and the national team should also alert that there is no national stock out. Focusing on this things will decrease the supply and commodity problem to some extent.

Practices, Roles, and Participation

I: Please describe to me the typical regional health office officials/experts roles in your Region/community?

a. How is Regional health office officials occupied over the course of the day? Are there seasonal differences (campaign/political assignment) in how you use time?

As regional health office official, what kind of work do you do? Do these works different when facility status changes (Rural, urban and geographical locations)?

R: As regional team we focus more on strategic issues, for instance at community level there is PWC, and we guide how it should be done. Another thing is, we create opportunity to increase level of awareness how program managers can monitor PPFP and PAFP by preparing documents and sharing with them. Another issue is following commodity issues. We follow national through Liaison EPSA, we follow especially community issues, we also work on demand creation via local media. Conducting program specific supportive supervision, continuously conducting reviews, if we have budget or partner, we identify focus areas and communicate accordingly. The activities will vary by location, setting and geography. The activities are not similar in areas with high demand gap and those areas with no problem with demand, areas with and with out supply problem in central areas where EPSA hub is closer and periphery areas, areas with adequate staff and those areas with high staff turnover, we give due emphasis and especially technical support to areas where there is high staff turnover, periphery and difficult to reach the supplies. The interventions vary with the location. For instance, areas like Konso, Amaro, Burji areas, South Omo, is not similar with activities done in Wolaita and Hadiya area. Those at center can own themselves, but those at periphery need close follow up and partner support. If you go to those areas the political leaders don't have good attitude towards FP. We must engage them and let them to advocate. There is also staff turnover. You will check availability of staff and let them get trained, they don't want to enter in to the community but you let them go to community.

I: Do regional health office officials like you have restrictions/barriers on the accessibility? What restrictions/barriers? How do the restrictions/barriers influence the access of the PP and PA family planning services and supportive social networks?

b. In your Woreda do the restriction/barriers change by social characteristic, such as (dis)ability, economic status, health status, educational level, religion, culture, geographical location, and marital status?

R: One of the barrier is political instability. Related to that the professionals lack commitment and lack of motivational means, and as I said before, turnover of trained staffs, budget constraints which hider from expanding the service as you want, supply and commodity interruption, the data tool it self. The DHS2 didn't access PPFP and PAFP. It is available in registration book, tally sheet at facility level but DHIS2 didn't have access which hinder tracking at woreda, zone and regional levels. For instance the level of awareness is one of the barrier, turnover of professionals, and the areas where this things common has been identified and resource or budget related problems are common in all areas. The instability related areas though it is whole over the region but most sever areas also identified. There areas where the barriers are that much and also areas where the barriers exist predominantly. There are shared as well as area specific barriers and they all are identified.

I: Do you participate in any meetings, workshops, trainings, pregnant women conference, family, community, government, or other social networks organizations? Please tell me more.

c. Please tell me how your participation in these organizations affect the PP and PA family planning services provision

R: There are national and regional review meetings that we participate most of the time. It can be prepared by RHB or partners, sometimes research conferences and dissemination workshops, and when we go to community there were occasions that we gather community and other stakeholders, we gather ideas from them, we also participate in training and capacity building activities. When our team organize the occasions, our focus is on PPFP and PAFP.

I: In your region do women assume leadership roles? What types of roles? Do these roles change by social characteristics, such as (dis)ability, economic status, health status, educational level, religion, culture, geographical location, and marital status?

d. Please tell us about your own experience with assuming any leadership roles?
e. Please tell me how your leadership roles affect the PP and PA family planning services provision

R: Related to leadership, there is concern now a days. Currently PPFP and PAFP is a flagship program. If you ask all officials and leaders, they have concern. Currently the leadership concern is good. It is good initiation. However, though the concern is good, still there is continuity problem and getting engaged in urging duties. It is most of the time focus on current issues. Women will accept if the approach is good and they can become agent for others, the service is currently at start up level and it didn't expanded, I can't say anything at this level. If you provide the service, they can be agent especially for PPFP and PAFP. They can lead and coordinate at community level.

I: In your Woreda/community who decides at what age people marry? What are the reasons for getting married at younger or older ages in this Woreda?

a. Please tell me how their age at marriage affects their PP and PA family planning services use

R: The community is just as you know. If I tell you about this (Wolaita) community, it is not similar with other areas, it is usually get married after 20 years. Unless it is rural part, there is trend of getting married after 20 years. But there is trend of giving birth immediately after marriage and completing the desire by giving birth continuously. Even educated one want to give birth to the level of children they need very fast and complete it soon. There is big problem in spacing. There is nothing especially that I observed in this community. If you go to other areas, there are bridal expenses and money needed. For instance if you go to South Omo area, she will get ready to marry in her 10th year.

I: What are your suggestions to improve women's roles and participation to enhance the use of PP and PA family planning services?

R: My suggestion is increasing male involvement but how it should be done, we need to look in to existing approach. Should we discuss issue with men and women together or men separately, but by any means we need to engage them, if you look in to every places the influence of men is high. The current intervention give focus mainly to PAFP and PPFP, but there are other integration points, like for example ART service, OTP/SAM; by the way the impact will be not less than current integration point if we integrate with SAM and ART. It is good to look in to this points, but it may be out of your objective since when you work you focus on your objectives. Besides, the engagement of health facility leaders is also important and looking in to PMT whether it has been done or not and which components are addressed in the PMT. Another thing is checking whether it has been included in the checklist of supportive supervision, and related recommendations are very important for higher officials.

Knowledge, Beliefs, and Perceptions

I: In your Woreda what is appropriate behavior for a woman after delivery and abortion regarding the use of PP and PA family planning? How do they differ from adolescent girls and young women?

a. How do these believe influence health behavior including the PP and PA family planning use?

What are the social beliefs and perceptions that share women's expectations and aspiration for health care including PP and PA family planning?

R: In community if the abortion happen spontaneously to the women, then that mother should get pregnant and give birth immediately. They didn't consider staying for 6 months. Except that the delivered women expected to delay next pregnancy. Almost all community because there is awareness. But there is problem of service availability and uptake. It could be delaying for it could be one year or more. Those who married they want to delivered frequently three or four children and rest for their rest of life. They didn't focus spacing. PPFP has high impact on spacing. This way of taught may affect PPFP and even if they use it they withdraw when it is one year. There is discontinuation.

I: In your opinion who should make decisions regarding use of PP and PA family planning services? What types of decisions do women make regarding use of healthcare including PP and PA family planning services? What types of decisions regarding use of healthcare including PP and PA family planning services are made jointly?

R: Normally according to our view, the mother should decide after having adequate information. Most of the time full free and informed choice. Providing options, adequate information and she will decide based on the information she got. I say the women who decides which method to use. Unless they decide together with her husband, it is less likely that they receive the service with their husbands. It less likely in our area.

Legal Rights and Status

I: In your community are women who delivered recently legally allowed to receive PP and PA family planning service?

- Is these same for boys and girls, people from different age groups, education background, ethnic groups, socio economic classes, excluded groups? How are they different? Why/ Why not?

R: The women have right to get the service. Unless it is related to availability of service as I mentioned earlier, 33 facilities are not providing the service and about 40 and above provide the service. It is right in facilities where there is service. It is right though there are awareness related issues among program managers and service providers. Use of FP is right. It the young girls right to receive the service.

1. How do recently delivered/aborted women access PP and PA family planning information and resource from the healthcare facilities?
   - Is these same for boys and girls, people from different age groups, education background, ethnic groups, socio economic classes, excluded groups? How are they different? Why/ Why not?
   - Do the people in your community support recently delivered/aborted women to access PP and PA family planning information and resources? How do they provide support? Who are these people? Why do they provide support?
   - Are there certain groups in your community who do not support this? Who are these people? Why don't they support?

Please tell me women's experience with accessing healthcare information and resources including PP family planning.

- Do you feel they have a right to get healthcare information and resources including PP family planning services? Why/why not?
- Do you think women's experience is different from other people in your community? How? Please tell me more
- Is there legal mechanism you can follow to ensure they get quality healthcare information and resources they need? Please tell me more

R: It is their right to access information's as well. Normally according to health sector, there are no service granted by law prevented from getting. Unless it is the issue of availability, for instance, a women have the right to get CAC based on legal grounds. If abortion service is not available, she should have another cost and get service from other places. There are other costs due to unavailability of the services. Except this things there are no service prevented from using it. The service are not availed and there should be activities that needs to be done to expand PPFP and PAFP services and activities related demand creation. Another things is program monitoring and evaluation and data use related activities needs to be strengthened. I hope good support will come from your side on this issues.

With this we have come to an end of the discussion. Do you want to add anything before we end the discussion or if you have any questions you want to ask?

Thank you for your time and in case you want, you can get in touch with us through the details mentioned in the consent form.

Summary

This interview has been conducted with RHB FP exert at Wolaita Sodo, Gutera café. The interviewee was stable and calm. He pointed out to all section of interview questions. The interview was conducted successfully after a number of schedule changes.

Key findings: PPFP and PAFP service are not available and accessible in 33 health facilities in Wolaita Zone. The coverage is about 10% currently. It varies with location and geographic areas as well as age group. Young women have less access and uptake of the service due to providers attitude and low level of adolescent health literacy. The services are free of charge but the consumable will some time not available and clients may be forced to buy from outside. There is also stock out of service due to poor monitoring by RRF, misdistribution and national stock out related to budget. There is high demand for services evidenced by high unmet need.

There are barriers such as staff turnover, the engagement of leaders is not continuous, budget shortage, stock out of commodities, activities that create demand at community level were not done very well, and less male involvement, spacing problem or withdraw of method after use of one year.

Efforts should be made to strengthen demand creation at community level, strong monitoring and evaluation system and supportive supervision needed. Further integration of FP with SAM and ART needs to be considered.

In-Depth interview conducted with recently delivered Mothers husband at Shella Borkoshe health center

Sociodemographic information of interviewee

Code	Keble	age	Sex	Educational-status	Job-status	Number of children
IDI_SHB_H_270722	Shella Borkoshe	30	M	primary	farmer	two

Assets and Resources

.

I; Health services (PP and PA family planning services) availability: Can you tell me the types of healthcare facilities that provide PP family planning services in your area?

R/ services provided in shela Health centre ; health post provide as this to delivery child giving space . Delivery distancing and planning. They provide injection for three month, for 3 years. Thank you came with us good services; we accept Family planning that benefits us, and our wives. I do not know other methods mentioned before, three-month injection and three years implants.

I; What types of FP services are available at the healthcare facilities?

R; *Females take three months and three years, I do not know other method*

I; Are these services equally available to boys, girls, young, old, married, unmarried, people from different geographic locations, religious or cultural background, language etc.? Please tell me more

R; which fit all,? Females take three months and three years, I don't know other method used for males.

1. Accessibility of the PP family planning services

I; Can you tell me how far the PP family planning services providing facility is located? How long does it take for you to access the facility?

R; It doesn't not take more than three minutes, I live near the facility

I; What type of transportation means do you use to reach the facility? How easy or difficult is it to use the transportation services?

R; Community can walk to the facilities, some can use motor bycle

I; Is there any cost associated with accessing the PP family planning services? Please tell me more about this

R, Services provided freely without payment for services, they did not ask to pay for services. She receive the services freely

I; Is the PP family planning service open at all time and is accessible with short waiting time at the healthcare facility? Are there types of FP methods of users' choice in the healthcare facility? Do healthcare providers receive and treat users with respect and dignity?

R; when women came to receive the services, it is always open..on Sunday they can receive the services. Eee they can get services at Sunday, they enter to the facilty. When I came here to

receive the fp services, I got as I wanted , if I want three months I get three months, if I want to get three years I can receive by my choices. They do not force me to take, it is my choice. Health workers do not choice for mothers to take this … and not that. They give as requested. Health workers don't prefer women from women. They give equally for all. They don't treat differently.

I; Are these services equally accessible to boys, girls, young, old, married, unmarried, people from different geographic locations, religious or cultural background, language etc.? Please tell me more

R; I think boys not use it for themselves they let their wife to use the service

I; How easy could you access the PP family planning service? Do you think your access to the PP family planning services is affected because of your background? How?

R; aking family planning immediately after delivery is difficult and in my perception giving immediately to mothers as delivered is difficult because she will be tried by laboring. It is difficult for mothers.

2. Means to access the PP family planning services

I; Please tell me about your household resources and assets. Please tell me the resources and assets belong to you.

R; we serve…. We have less ability to fulfil what we need. I have land for farming, house, kitchen, we produce maize, bean. I have one cow; I have no single owned things. Assets belongs to my wife and me.

I; How is the use of the household resources and assets decided? Who makes the decision? Do you involve in the decision-making? How do you think that affects your access to the healthcare services including PP family planning?

I: *we discuss and agree together with my wife, if we need to sell the cow. Why we sell it, we discuss together. I didn't only myself to decide, we need to agree each other. We discuss to take family planning because if child come soon by one another. We discuss and agree with my wife she has to receive three years family planning method.*

I; Please tell me about your household income. Please tell me about your own income.

R, from farming we get to kuntal , we have small farming land . Yearly we get one kuntal maize, 1 kuntal beans and 25 kg other.

I; How is the use of the household income decided? Who makes the decision? Do you involve in the decision-making? How do you think that affects your access to the healthcare services including PP and PA family planning?

R; I do not say it belongs to me, we use together for our household consumption.

I; In your community how is use of PP family planning seen? Is it acceptable for women to use PP family planning? Why/why not? Please tell me more

R; from the birth spacing community have benefited. Now a day's community changed the practice of birth spacing. Previsiouly community delivery child without spacing. Now making birth spacing. Due to birth spacing community benefited , children became health and it also benefited mothers and households community perceive varies according their own, some say why she hurry to take birth spacing as soon as immediately giving birth. Say what is hurrying here. Community live according to their home and perception differs. I myself perceive doing sexual intercourse as soon as wife gave birth consider as her husband is died. Kkkkk people live according to their home and some my do sexual intercourse even she delivery birth. My wife did not received birth spacing immediately as she delivered, she will came and take the birth

spacing. Kkkkkkk, Still we didn't make sexual until this time. I agree my wife to take birth spacing. Community perceive it is good taking immediately after delivery. If delivered mother receive birth spacing community might perceive it is good practice and do not perceive it is bad.

The delivered mother might not happy to receive birth spacing immediately after birth because she is tried during delivery labor. I am also not happy if she receive immediately after delivery. Community and family and neighbor also do not happy, they may why she stay for three to four moth and why she hurry to receive it. Other people might point her.

I; In your opinion what should be done to improve access to the PP family planning services for all recently delivered mothers in your community?

R; I know my home and myself, there is no hindrance to receive the birth spacing. She might get the services. Health workers should teach mother about birth spacing explaining if you didn't receive birth spacing you will be pregnant and teach to women to receive it immediately after birth.

Mother can decide to receive which birth spacing she could get. Husband and wife should discuss and agree to use birth spacing which is preferable for them. They discuss together if they give birth without birth spacing affecting delivered child as well as mother and other child next delivered. They have discuss and use birth spacing.

Practices, Roles, and Participation

I; Please describe to me the typical women's roles in your community?

R; in home women prepare food, fetch water, nurture their children, she involve social activities like Idir, burial ceremony, they interact with their society, keep her house and participate in social activities.

I; How are women and girls occupied over the course of 24 hours? Are there seasonal differences in how women and girls use their time?

R; sometimes when child go to school and there is burial ceremony, she could have burden of her work. They perform and carry out their responsivities. When she became old she doesn't carry out her responsibility because she became weak.

I; What kind of work do you do? Do these works change when your age or status changes (young, unmarried and married, older)?

R; since I m farmer most of the time I stay in farm, collect grass for cattle, bringing drinking water for cattle, yes when my children become grow they give rest or share with me

I; Do you have barriers on your access? What barriers? How do the barriers/restrictions influence your access to the PP family planning services and supportive social networks?

R*; there is pain and she became tried in labour and she might not happy to receive the birth spacing immediately. It is difficult for her to receive birth spacing.*

I; In your community do the restrictions/barriers change by social characteristic, such as (dis)ability, economic status, health status, educational level, religion, culture, geographical location, and marital status?

R, my wife gather in kebele, HAD meeting and involve in social meeting. Previously she received birth spacing with discussion and agreement with me.she has willing to receive. She attended training on birth spacing ant tell me some points.

I; Do you participate in any family, community, government, or other social networks organizations? Please tell me more.

R; ye I used to participate in all social networks

I; Please tell me how your participation in these organizations affect your PP family planning services use

R; nothing affected me from using it

I; In your community do women assume leadership roles? What types of roles? Do these roles change by social characteristics, such as (dis)ability, economic status, health status, educational level, religion, culture, geographical location, and marital status?

R*: yes , she involve in church leadership, not because of education because they attend church services. Because they are known by community and assigned by them..*

I; Please tell us about your own experience with assuming any leadership roles?

R; I have not practiced leadership, I didn't involve in leadership.

- Please tell me how your leadership roles affect your PP family planning services use

I; In your community who decides at what age should people marry? What are the reasons for a women getting married at younger or older ages?

R; A boy can ask her, perceiving she is matured for marriage. She also decide she want to marry. Kkkkkkk when they physical mature to marry. If they love each other and they became marry, they themselves decide to marry. No one decide for them.

I; Please tell us about your own marriage experience? How old were you when you marry? What are the reasons you she get married at that age?

R; from my experience I did not requested for marriage, some asked her for me, then when I approached there I asked her marry me. I gave for her what she want like clothing, we married together, and she delivered child for me. Kkkk when I marry I age might no more than 15 years. We stayed marriage for ten years. Kkkkkk it is very interesting to marry, that influenced to marry my wife.

I; Please tell me how your age at marriage affects your PP family planning services use

R: When she marry me, her age might not more than 15 years. Using birth spacing helped as more, after using birth spacing, we built our home.

I; what are your suggestions to improve women's roles and participation to enhance the use of PP family planning services?

R: I would suggest gathering community together and other health workers came and teach us, there is a local saying " Local ladies don't respect a local gay" you know when those near to community teach community might not give full attention for the training and accept fp. It would be better those from other area came teach community could accept the family planning. Teaching both women and men would be suggested, if you teach only women it could not work well. It needs teaching both men and women they could accept the training and it also helps.

Knowledge, Beliefs, and Perceptions

I; In your community what is appropriate behavior for recently delivered woman regarding use of PP and PA family planning services? How do they differ from adolescent girls and young women?

R; as I said previously, it needs education, as I mentioned before there is mentally poor people that didn't want to hear about the family planning, those educated people accept the

family planning and bring change and others could be trained can be changed for the future. If there is training woman can accept the family planning, I would suggest providing training

I; What are the social beliefs and perceptions that share women's expectations and aspiration for PP family planning?

In your opinion, who should make decisions regarding use of PP family planning services? What types of decisions do women make regarding use of healthcare including PP family planning services? What types of decisions regarding use of healthcare including PP family planning services are made jointly?

R; Both woman and man should make a decision to make the use of family planning, in a family woman might want to give birth and at same time man not want to give child, in the other hand man want to have child and but woman cannot want. Therefore both should agree appropriate time for them to have family planning. They should both agree and decide.

Legal Rights and Status

1. In your community are women who delivered recently legally allowed to receive PP family planning service?

   R; I didn't think it is legal for giving birth spacing immediately after delivery because she is tired and pain due to ,labour.

   - Is these same for boys and girls, people from different age groups, education background, ethnic groups, socio economic classes, excluded groups? How are they different? Why/ Why not?

2. How do recently delivered women access PP family planning information and resource from the healthcare facilities?

   R: they get information health workers; health workers teach mother home-to-home visit and call, them to health facility to give training. There are community members encourage mothers to receive birth spacing. Some community members say it is good receive birth spacing. Receiving immediately after delivery is not good, even they need should have receive after 15 day or 20 days. Because it might burden for mothers. I did not hear that community members support receiving birth spacing immediately after delivery.

   - Is these same for boys and girls, people from different age groups, education background, ethnic groups, socio economic classes, excluded groups? How are they different? Why/ Why not?

   I; Do the people in your community support recently delivered women to access PP family planning infatuation and resources? How do they provide support? Who are these people? Why do they provide support?

   R; Health professionals and educated mothers support woman to receive the service

I; Are there certain groups in your community who do not support this? Who are these people? Why don't they support?

   R; I don't think so

3. Please tell me your experience with accessing healthcare information and resources including PP family planning.

   R; we get access for birth spacing information from health post, they visit home to home visit.

I; Do you feel you have a right to get healthcare information and resources including PP family planning services? Why/why not?

R, I do not have right. What is the relation between heath worker and me? I don't feel I have right.

I; Do you think your experience is different from other people in your community? How? Please tell me more

R; I think it is the same for all

I; Is there legal mechanism you can follow to ensure you get quality healthcare information and resources your need? Please tell me more

R; yes, if health workers do not provide the information and service, I have right to ask their leader and complain they are not giving the services.

With this we have come to an end of the discussion. Do you want to add anything before we end the discussion or if do you have any questions you want to ask?

Thank you for your time and in case you want, you can get in touch with us through the details mentioned in the consent form.

R, I have no question, thank you

Summary

This interview conducted with recently delivered mother husband at Shella Borkoshe health center in 27/0722. He had no adequate awareness regarding pp fp and PA fp service.

Key finding were-He knows only three months and three years family planning method, It is better for females to use family planning methods after 15 0r 20 days, both wife and husband should discuss and decide together, he doesn't feel he have right to get the quality service. When those near to community teach community might not give full attention for the training and accept fp. It would be better those from other area came teach community could accept the family planning, Local ladies don't respect a local gay"

**Interviewee code**	**IDI_SB_MF_27_7_2022**
**Sex**	**Female**
**Age**	**26**
**Service year**	**2**
**Job title**	**MCH focal person**
**Name of interviewer**	**Befekadu Bekele**
**Date**	**27/7/2022**
**Time started**	**3:00**
**Time finished**	**3:59**
**Venue**	**Shela Borkoshe Health center**

I: Health services (PP and PA family planning services) availability: Can you tell me the types of healthcare facilities that provide PP and PA family planning services in your area?

a. What types of FP services are available at the healthcare facilities?
b. Are these services equally available to boys, girls, young, old, married, unmarried, people from different geographic locations, religious or cultural background, language etc.? Please tell me more

R: Thank you. We give implants, jedile ,COC,IUCD. However there is no one trained on immediate post partum family planning and post abortion family planning services. We have all types of family planning methods and we give the services according to their choice. if they prefer implants we give them implants and the acceptance is also good. If we face shortage we request and we get all F/P methods we need.

I:Are these services equally available to boys, girls, young, old, married, unmarried, people from different geographic locations, religious or cultural background, language etc.? Please tell me more

R:it is not equally available. For example Tuba ligation which is given for males it is not available here.

I: Accessibility of the PP and PA family planning services

-Can you tell me how far the PP and PA family planning services providing facilities are located? How long does it take for women to access the facilities?

-What type of transportation means do they use to reach the facilities? How easy or difficult is it to use the transportation services?

-Is the PP and PA family planning service is accessible at all time at the healthcare facilities? Are there types of FP methods of users' choice in the healthcare facilities?

-Are these services equally accessible to boys, girls, young, old, married, unmarried, people from different geographic locations, religious or cultural background, language etc.? Please tell me more

-How easy could they access the PP and PA family planning services? Do you think that their access to the PP and PA family planning services are affected because of their background? How?

R: It is far for the community to get the service. But there are family planning services given at health post level. for example depo is given at health post level. Implants, jaddle and IUCD are given at health center because this methods are given by trained health care workers. Transportation is one of the challenges for the community to get the service. It may take an hour or more than that for them to get the service. most of the time they come here on foot. Because there are places which are not good motor or car.They use motor after move a long distance on their foot. most of the time they come here on foot because of lack of money. Therefore, it not easy for them to access the service.

I: Is there any cost associated with accessing the PP and PA family planning services? Please tell me more about this?

R:There is no cost associated with this. the service for maternal health service is free of charge.

I: Is the PP and PA family planning service is accessible at all time at the healthcare facilities? Are there types of FP methods of users' choice in the healthcare facilities?

R: Honestly speaking the health care workers are ready to give these services at any time. When we are assigned in the health center we have shifts and the skilled personnel will be assigned at any time so that mothers can get the service at any time. Usually there is no trend of coming in weekends but we tell them that the service is given at any time. We tell all family planning options and counsel them to choose from the available methods. There are occasions where we can influence and help them decide the appropriate method for them. They get the F/P they need but we give them options to choose. They discuss with us before they make decision. Most of the time we respect their preferences but there are some conditions where we help them change their decision based on some reasons.

Means to access the PP and PA family planning services

I: Please tell me about your facilities' resources and assets.Please tell me the resources and assets belong to these health facilities to provide PP and PA FP service.

-Please tell me about income of in this Woreda. Please tell me about your woredas income.

R: The instruments we have are old ones. so we face challenges. instruments are not easy to use. The family planning drugs are available except POP.

I: what about room setup for this service?

R: I can say there is no good room setup. We don't have room for abortion services. Almost there is no adequate room for these services. ANC, family planning is in the a single room, the place for counseling and procedure is the same.

I: How is the use of the facility/Woreda health office resources and assets decided? Who makes the decision? Do you involve in the decision making? How do you think that affects access to the healthcare services including PP and PA family planning by women?

R: I can report for the head of the health center if face scarcity. The only thing I do is reporting when there is scarcity of drugs and other necessary materials we need. Other than this I am not

participating in decision making. It is good if we participate in decision making because we can easily get what we need. we can get responses as soon as possible.

I: How is the use of the woreda's income decided? Who makes the decision? Do you involve in the decision making? How do you think that affects their access to the healthcare services including PP and PA family planning?

R: most of health center income is from community based health insurance. Each member of Community based health insurance pay 300 birr and use the service. I cannot exactly say this amount of money we get. Apart from this the income we get from others is very few. The head of health center and finance bodies,human resources, pharmacists decide on budget issues. My role as MCH focal person is just reporting all necessary equipments and drugs when we face scarcity.

I: In your woreda how is use of PP and PA family planning seen? Is it acceptable for women to use PP and PA family planning? Why/why not? Please tell me more (what about boys and girls)

R: it is new for the community it is not practiced in previous time. As we know the community had negative attitude towards implants in the past, the same is true. it is difficult to convince mothers to use immediate post partum and post abortion family planning services. We counsel them about immediate post partum family planning. Those who are prmi mothers go to their family after they give birth,so they want to use the service after forty five days when they come to vaccinate their child. Because they stay with their family and depart from her husband for few months they want to stay until they become stable. This is one of the challenge. In addition to this those who have multi parity also say that we will not have sexual intercourse until we bring the child to church for blessing. We may have intercourse after forty five days even though they live in the same house. There are also mothers who immediately use the service. There are mothers who don't see bleeding or menstrual cycle for a year. They are happy to start family planning as early as possible. Because they have fear that If they wait for menstrual cycle ,they can conceive accidentally so that they want to use family planning immediately after birth.

I: how is the use of PP F/P and PAFP is seen in the community? What do mothers who accompany a mother who came for delivery services say ?Would you add more?

R: As we have observed during counseling some mothers recently delivered women say" *let me take rest ,I don't want to be burn ,I don't want to rush to use family planning immediately after birth. Why are you adding additional pain ,because this is double burden.'' Let me take rest at least for a month and or I will think about it after forty five days.''* They don't oppose the services but their question is related to timing. Using the service immediately after birth doesn't give sense for them. They are willing to use service after forty five days, or when they come vaccinating their children.

I: what about post abortion family planning is seen?

R:They are willing to use family planning during post abortion. Adolescents before 18 years of age are not willing to use the services. Married ones don't challenge us to use post abortion family planning.

I: In your opinion what should be done to improve access to the PP and PA family planning services for all women in your community?

R: The community has no problem regarding uptake of the services. What matters a lot is the way we convince or persuade to use the services. Health extension workers are very close to mothers because they give home to home services. Therefore, attention should be given for them. There should be training ,counseling services for HEWs. Mothers in the community can easily access health extension workers. The health extension workers should create awareness for the community.

I:what else?how and what should be done to improve access to the PP and PA family planning services in addition to what you have said?

R: It is better if all health workers have equal understanding about the service. Actually this idea is introduced very recently. The health care workers ,the community ,the health extension workers have little information about immediate post partum and post abortion family planning service.

Practices, Roles, and Participation

Now we are going to discuss about your role and responsibilities.

I: Please describe to me the typical Woreda health office officials/experts roles in your Woreda/community?

R:I am engaged in delivery service, give family planning service.

I: How is Woreda health office officials occupied over the course of the day? Are there seasonal differences (campaign/political assignment) in how you use time?

As Woreda health office official, what kind of work do you do? Do these works different when facility status changes (Rural, urban and geographical locations)?

R: These are my routine activities. we arrange women' conference in the community level. As I have said earlier there are women who live remote areas who are in need of family planning service. As the health extension workers mobilize the community we give long acting family planning methods. This is what we do at community level.

I: Do Woreda health office officials like you have restrictions/barriers on the accessibility? What restrictions/barriers? How do the restrictions/barriers influence the access of the PP and PA family planning services and supportive social networks?

a. In your Woreda do the restriction/barriers change by social characteristic, such as (dis)ability, economic status, health status, educational level, religion, culture, geographical location, and marital status?

R: One of the barriers could be husbands. Sometimes mothers tell us that their husbands are not willing and don't allow them to use family planning. They come here secretly. Some mothers put their appointment card in their neighbor's house due to fear of their husband. There are mothers who put appointment card under their armpit when they come to health center because they don't want to be seen by their husbands. We ask them why they are hiding their appointment card and they tell us that their husbands don't allow them to use because they want to have children. Therefore husbands can have influence on immediate family planning utilization.

I:what about others? Cultural , religious related barriers?

R: So far I did not encounter mothers who are not willing to use family planning due to their religion. What I encountered is related to husbands. Honestly speaking I didn't encountered mothers who don't want to use immediate family planning due to culture.

I: Do you participate in any meetings, workshops, trainings, pregnant women conference, family, community,government, or other social networks organizations? Please tell me more.

R: yes I have participated in trainings. I have received training on abortion, immediate post abortion family planning.

I: Please tell me how your participation in these organizations affect the PP and PA family planning services provision

R: It helped us a lot because we have got new knowledge, participation in the training helped us to develop our skill and confidence. Those who received training can give better service for the community. We have got additional information on the existing knowledge., helps you to have confidence.

I: In your Woreda do women assume leadership roles? What types of roles? Do these roles change by social characteristics, such as (dis)ability, economic status, health status, educational level, religion, culture, geographical location, and marital status?

- Please tell us about your own experience with assuming any leadership roles?
- Please tell me how your leadership roles affect the PP and PA family planning services provision

R: They have different roles and they are trying their best to accomplish their responsibilities. There are focal persons in the health center, they play their roles. It is good to engage women in leadership because they are successful in different leadership places. This can play role in enhancing post partum family planning services, because it is possible to share skill, knowledge for others.

I: In your Woreda/community who decides at what age people marry? What are the reasons for getting married at younger or older ages in this Woreda?

R: I don’t think that family has a role to decide on adolescents issues. Most of the time males decide for marriage. Most of the time it is male’s role to decide on marriage issues. Most of the time they marry when they are at the age of 25.There also adolescents who get marry at their early stage. For example there are adolescents who marry at the age of 15 and 16.We have encountered this during antenatal follow ups.

I:What are the reasons for getting married at younger or older ages in this Woreda?

R: we don’t see girls who report as rape cases as previous times. They marry voluntarily, no body forces them to get married. We rarely hear that few girls report rape cases.

I: Please tell me how their age at marriage affects their PP and PA family planning services use

R: It can influence pp family planning service. There are adolescents who get marry because the girl conceived before formal marriage. they may not want to abort once the girl conceives and they get marry soon. Therefore, one of the reason pushes them to get marry is pregnancy before formal marriage. These people start to use long acting family planning immediately after birth.

I: What are your suggestions to improve women’s roles and participation to enhance the use of PP and PA family planning services?

R: women share their experience for others ,they tell others especially to the young age groups to limit the number of children. They share their own experiences, challenges they experienced due to having many children without adequate spacing. They tell them that they wouldn’t face different challenges if there were family planning methods were available in their times. Therefore, elder age groups encourage the younger age groups to use immediate family planning services. They share their past experiences and teach others to use the services.

I: In your Woreda what is appropriate behavior for a woman after delivery and abortion regarding the use of PP and PA family planning? How do they differ from adolescent girls and young women?

-How do these believe influence health behavior including the PP and PA family planning use?

R: They accept family planning method. But reason they are challenged to use is because the approach is changed ,which is immediately after birth. Previously there was a trend to use family planning after forty five days. They know about it(using family planning after forty five days).what is new for the mothers is starting to use F/P immediately after birth. They say to the midwive ‘‘ *Axadefshign ke 45 ken behual exeqemalewu’’*. “*I will start to use the service after forty five days ,but you let me use the services soon after birth that seems a rush decision.’’ Let me start after I enter church because we don’t do sexual intercourse with in forty five days after birth’’.* This is what majority of the community say about 60% of recently delivered mothers have such a thought. Those who delivered with CS are willing to use immediate post partum family planning immediately after birth because they know that they should wait at least two years after they gave birth. A woman delivered in CS will be happy to use immediate post partum family planning, she will not oppose.

I: What are the social beliefs and perceptions that share women’s expectations and aspiration for health care including PP and PA family planning?

R: As to my observation the community has no negative attitude towards immediate post partum family planning services. They are willing to accept the service immediately after birth except the timing. “*Texadefe …Koch lay mehonu* texadefe’’ because this is new approach they perceive that the timing after birth is hasty or fast because this is not practiced before. I think this challenge will exist until they become familiar. Because since the service is given immediately after birth they think about their pain not about family planning use. So far I did not encounter mothers who are not willing to accept family planning.

I: In your opinion who should make decisions regarding use of PP and PA family planning services? What types of decisions do women make regarding use of healthcare including PP and PA family planning services? What types of decisions regarding use of healthcare including PP and PA family planning services are made jointly?

R: Husbands should decide.

I: why ?

R: we live in male dominant culture as Ethiopia in general. Most decisions are made by husbands. We tend to use and follow what our husbands say. In rural setting, It husband who decides in every matters. Therefore, women will accept everything said by their husbands. Therefore intervention should be done on husbands. If they get awareness, it is very easy to convince their wives because they accept everything said by their husbands. Therefore, acceptability of immediate post partum family planning will be easy if there is involvement of husbands because this is what we have seen during counseling, mothers use to say “let me discuss with my husband before I use the service’’ It is very difficult in this community for a mother to decide on this matters. She can decide but the decision is done after discussion with husband. It is better if the decision is made jointly. Mothers wouldn’t hide themselves if they had discussion with their husbands. They can plan and decide for how long they should stay after delivery, and they can decide on family planning method to be used.

.

I: In your community are women who delivered recently legally allowed to receive PP and PA family planning service?

- Is these same for boys and girls, people from different age groups, education background, ethnic groups, socio economic classes, excluded groups? How are they different? Why/ Why not?

R: I think it is legally allowed. But I cannot say that it is hundred percent legally allowed for different age groups because I have no reference.Laughing….

I: How do recently delivered/aborted women access PP and PA family planning information and resource from the healthcare facilities?

- Is these same for boys and girls, people from different age groups, education background, ethnic groups, socio economic classes, excluded groups? How are they different? Why/ Why not?
- Do the people in your community support recently delivered/aborted women to access PP and PA family planning information and resources? How do they provide support? Who are these people? Why do they provide support?

- Are there certain groups in your community who do not support this? Who are these people? Why don't they support?

R: They get information from the health extension workers, health care workers, from radio. However, it is not the same for boys and girls. They don't access information and resources equally.

Please tell me women's experience with accessing healthcare information and resources including PP family planning.

I: Do you feel they have a right to get healthcare information and resources including PP family planning services? Why/why not?

R: Yes they have right to use the service. If they want use the service they have right to get information

I: Is there legal mechanism you can follow to ensure they get quality healthcare information and resources they need? Please tell me more

R: There is no legal mechanism to follow.

I: How do you ensure whether they get healthcare information and resources?

R: we have ways to ensure, they will call to us if they don't get any information or resources they need. But there is no legal mechanism where we can follow or ensure whether they get healthcare information and resources.

I: With this we have come to an end of the discussion. Do you want to add anything before we end the discussion or if you have any questions you want to ask?

R: I have got a lessons about immediate post partum and post abortion services and I want this services to be scaled up in the community. There is women conference in kebele. The same thing should be done on husbands. There should be husbands conferences that could be done every month. Husbands should get counseling services and get awareness and if there is such a thing ,it will be successful.

Thank you for your time and in case you want, you can get in touch with us through the details mentioned in the consent form.

I:Thank you so much.

R:Thank you.

Summary

- ✓ There is lack of trained personnel on immediate post partum and post abortion family planning
- ✓ Males family planning method like tubal ligation is not available
- ✓ For some people the place they live is far from the health center, so it is not easy to access
- ✓ There is lack of POP, outdated instrument, no separate room to give these services
- ✓ Not participated in decision making
- ✓ Fear of challenge to convince /persuade mothers about immediate post partum and post abortion services
- ✓ There are mothers who don't see bleeding or menstrual for several month,this kinds of women are willing to use PPFP and PAFP
- ✓ The way health care workers information can influence PPFP and PAFP
- ✓ The decision making power of women is under question/ low/b/c all decisions are made by their husbands
- ✓ The timing of immediate post partum and post abortion service is not conducive for them ,they are not happy with this.

Interview details

Interviewee	Recently deliverd woman
Age	29
Interviewee code	IDI_SE_WO_260722
Name of interviewer	Beimnet Desalegn
Date	26/07/22
Time started	6:24 lt
Time finished	6:59 lt
Venue	Seke, recentely deliverd mothers' home

Assets and Resources

Discussion

Now we are going to discuss about the Healthcare resources and assets available to recently delivered mothers in your area.

I: - Health services (PP and PA family planning services) availability: Can you tell me the types of healthcare facilities that provide PP family planning services in your area?

a. What types of FP services are available at the healthcare facilities?
b. Are these services equally available to boys, girls, young, old, married, unmarried, people from different geographic locations, religious or cultural background, language etc.? Please tell me more

R: - I know health post and health center … in our kebele providing family planning service for the community…

There is a 1 to 5 team who teaches women about contraceptive methods with community health extension workers.

Previously we deliver at home, but now we are aware of the consequence, which we got from the community health working team and now we use the health centers for delivery services with compassionate care and respect by health workers. They (health workers) thought us about our health during pregnancy follow-up... they counsel us on the different health issues.

I did not use the contraceptive method … this is my second birth and after the first birth I was in other places (in college) for a long time because of this at that time I was not using

contraceptives… he (husband) is also in another place due to his work… we were not together because of this I was not using contraceptive methods. but on my return, I got pregnant. But now since we are living together I plan to use contraception…

I know drugs which are used for three months, depo, again there are methods which taken for five years… even I am not using it I heard from my colleague and friends.

Yeah, the service provider was serving in a good manner… they (health workers) are compassionate and respect full … they serve equally for all clients who need the service.

I: - Accessibility of the PP family planning services

c. Can you tell me how far the PP family planning services providing facility is located? How long does it take for you to access the facility?
d. What type of transportation means do you use to reach the facility? How easy or difficult is it to use the transportation services?
e. Is there any cost associated with accessing the PP family planning services? Please tell me more about this
f. Is the PP family planning service open at all time and is accessible with short waiting time at the healthcare facility? Are there types of FP methods of users' choice in the healthcare facility? Do healthcare providers receive and treat users with respect and dignity?
g. Are these services equally accessible to boys, girls, young, old, married, unmarried, people from different geographic locations, religious or cultural background, language etc.? Please tell me more
h. How easy could you access the PP family planning service? Do you think your access to the PP family planning services is affected because of your background? How?

R: - the health center is not far for me … I can walk it does not need any transportation… in case of delivery for those who live far away from the health center, there is an ambulance service the health center provides to bring them to the health center. We did not worry about transportation access in case of delivery. For other services people come to the health centers by different means, some may use motorcycles, walk or use any available transportation means. To reach the health center it takes 5 to 10 minutes for me… to walk, many people prefer to walk …

They (health workers) do not ask for money for service … the service is free. Even though I did not use contraceptives, I heard from my colleague and friends the service were free of charge. I also observed in the health centers and health posts when women used contraceptives free of charge.

Yeah, sometimes I heard that women were not getting the drug they choose to use … but the health center here services 24 hours…. At health post sometimes when they leave for a meeting or training the health post was closed during a working hour at that time women's come and use service at the health center as the health center stands for four or five kebele together… when health post is closed because of the different reason they(women) come to the health center. So, at the health post, there are times when the health post was closed for a week or more when extension workers are out of kebele for training or meeting.

Regarding contraceptive option as I heard… all option of drugs was available at the health facility (health post or health center).

Okay, now there are individuals who were using contraceptive methods while they were living with their family (before getting married) as I know they were using free and also health workers serve them appropriate manner. They (health workers) see all clients equally.

I: - Means to access the PP family planning services

i. Please tell me about your household resources and assets. Please tell me the resources and assets belong to you.
j. How is the use of the household resources and assets decided? Who makes the decision? Do you involve in the decision making? How do you think that affects your access to the healthcare services including PP family planning?
k. Please tell me about your household income. Please tell me about your own income.
l. How is the use of the household income decided? Who makes the decision? Do you involve in the decision making? How do you think that affects your access to the healthcare services including PP and PA family planning?
m. In your community how is use of PP family planning seen? Is it acceptable for women to use PP family planning? Why/why not? Please tell me more

R: - we both are government workers. We use our money together … we save our monthly income for emergencies and other purpose. If we have something which requesting at one of our family or in our home we use money from our saving money. We have an agreement in our home on how to spend and use our money. As we have money we save together … I have the freedom to use it at any time for a required purpose… like in case of a child ill and other.

In our community, (PP/PA) is seen as a new thing and many women were hesitant to use immediate post-partum family planning. I think those who have formal education were good at using this service because they have aware of it.

I: - In your opinion what should be done to improve access to the PP family planning services for all recently delivered mothers in your community?

R: - In my opinion, the main thing health worker has to do is educate women about post-partum or post-abortion family planning. Also organizing community forum to discuss together, sharing experience by users … if these things were done appropriately it could be improved. educating about birth spacing and the importance of contraceptives for spacing …. Putting it on agenda in 1 to 5 team discussion and involving health workers in this discussion… consulting health workers.

<u>Practices, Roles, and Participation</u>

Now we are going to discuss about your women's and your role and responsibilities.

I: - Please describe to me the typical women's roles in your community?

- How are women and girls occupied over the course of 24 hours? Are there seasonal differences in how women and girls use their time?

R: - yeah, a woman has many roles in the community or in their household … at her house, she has to cook for her family, care for cows or animals at their house, care for the baby if she had a baby and generally all household activities were expected to be done by women in our community.

Yeah, the workload varies sometimes as conditionally… during Autumn and winter season at that time may be women has more work burden than the other season of the year.

I: - What kind of work do you do? Do these works change when your age or status changes (young, unmarried and married, older)?

R: - yeah, I have the responsibility of caring for my family… home care, cooking, …. Yes as women age and birth increase the role of women also increase… she is going to engage in social issue more… as well as family responsibility increase.

I: - Do you have barriers on your access? What barriers? How do the barriers/restrictions influence your access to the PP family planning services and supportive social networks?

- In your community do the restrictions/barriers change by social characteristic, such as (dis)ability, economic status, health status, educational level, religion, culture, geographical location, and marital status?

R: - I am new to this village, I was in another village before… during my stay I see the community is very nice and cooperative ... I haven't seen any barriers for me in accessing any service here ... from the community members' side. Sometimes there is a rumor that religious preachers may not allow using contraceptives… but many women are using contraceptives. Now people consider their living conditions and do what is good for their health and family. Generally, I don't have any restrictions in accessing health care services. ... all religions did not allow the use of contraceptives but many women were using them.

I: - Do you participate in any family, community, government, or other social networks organizations? Please tell me more.

- Please tell me how your participation in these organizations affect your PP family planning services use

R: - Even I am not participated as needed in all meeting ... I was participating in very important meetings and those meeting helped me in many ways. I have participated in the seasonal meeting

which held with health extension workers and what I heard from health workers during our meeting and seen other woman experience … there are thinks which made me happy and help me a lot to know about contraceptives.

I: - In your community do women assume leadership roles? What types of roles? Do these roles change by social characteristics, such as (dis)ability, economic status, health status, educational level, religion, culture, geographical location, and marital status?

- Please tell us about your own experience with assuming any leadership roles?
- Please tell me how your leadership roles affect your PP family planning services use

R: - yeah, now day women are assuming leadership position in better way than from the previous time. At government office or religious institution they are participating very well … for instance I do not remember her name, eee since I am new for this area… 1 to 5 team leaders who advised me to go to health center for delivery were female. What I want to say is she is putting their part or contributing her part … informing me.

To bring in to leader position, ... is there are quality in sharing what they had to other, eee again who are able to share what she learned to the other… those who is knowledgeable… people consider this thing to assign a person to leading position.

I did not assume any leadership position before … I am just government worker, I have plan for the future.

I: - In your community who decides at what age should people marry? What are the reasons for a woman getting married at younger or older ages?

- Please tell us about your own marriage experience? How old were you when you marry? What are the reasons you she get married at that age?
- Please tell me how your age at marriage affects your PP family planning services use

R: - In our community, women marry starting from 15 years and above… there are also women's who stay single till 25 years and above. I don't think women marry under 15 years of age…

Okay, am around 18 years old while marring. I was completed my collage study before I was married and worked for two years in government office before getting marriage.

The main reason to get marry might be family economically unable to teach their child, other women may look at their friends living standard and wish to live like them, there are also women's who got marriage due to some personal problems.

Those who married at early age may have face health problem … during pregnancy and delivery. They are not volunteer to use contraceptive in early stage of their marriage because they have fair of side effect.

I: - What are your suggestions to improve women's roles and participation to enhance the use of PP family planning services?

R: - To improve women's participation health workers should work intensely in awareness creation among the women about contraceptive method.

Knowledge, Beliefs, and Perceptions

Now we are going to discuss about the issues faced by adolescents and women in accessing PP and PA FP services in your opinion.

I: - In your community what is appropriate behavior for recently delivered woman regarding use of PP and PA family planning services? How do they differ from adolescent girls and young women?

a. What is the appropriate behavior for a recently delivered mother? How do these believe influence health behavior including the PP family planning use?

R: - To improve women's participation health workers should work intensely on awareness creation among women about contraceptive methods.

I: - What are the social beliefs and perceptions that share women's expectations and aspiration for PP family planning?

R: - I do not know any belief related against PP or PA family planning … among our community…

I: - In your opinion who should make decisions regarding use of PP family planning services? What types of decisions do women make regarding use of healthcare including PP family planning services? What types of decisions regarding use of healthcare including PP family planning services are made jointly?

R: - it's better if she uses it after discussing it with her husband. It should be decided by both partners… that is why if she decides and takes without the partner's say and if he wants to have another baby then that may break their relationship. so they should have discussed to be the same. So, they have to decide together.

Legal Rights and Status

Now we are going to discuss about the marriage related process prevalent in this community.

I: - In your community are women who delivered recently legally allowed to receive PP family planning service?

- Is these same for boys and girls, people from different age groups, education background, ethnic groups, socio economic classes, excluded groups? How are they different? Why/ Why not?

R: - Yeah, it is legally allowed … to use contraceptives during the post-partum or post-abortion time. But there should be a woman's agreement to take contraceptive methods. There should also be male partner involvement.

I: - How do recently delivered women access PP family planning information and resource from the healthcare facilities?

- Is these same for boys and girls, people from different age groups, education background, ethnic groups, socio economic classes, excluded groups? How are they different? Why/ Why not?
- Do the people in your community support recently delivered women to access PP family planning infatuation and resources? How do they provide support? Who are these people? Why do they provide support?
- Are there certain groups in your community who do not support this? Who are these people? Why don't they support?

R: - Mainly community health workers teach women about contraceptives ... so they (women) got information from them (health workers). Also, 1 to 5 team leaders also disseminate health-related information among their team. In this way, information reaches the woman.

Yeah, there are supporting groups… as I mentioned above 1 to 5 team leaders and health development army leaders support women in the community to come and use the services in the health centers or health posts.

No there is no officially opposing body here…

I: - Please tell me your experience with accessing healthcare information and resources including PP family planning.

- Do you feel you have a right to get healthcare information and resources including PP family planning services? Why/why not?
- Do you think your experience is different from other people in your community? How? Please tell me more
- Is there legal mechanism you can follow to ensure you get quality healthcare information and resources your need? Please tell me more

R: - yeah, women know that it is their right to get information ... yes people know it.

Yeah, many women were using contraceptives here, so they can confirm from the user as well as from their experience. So, women can compare what they see or observe with what they hear about…

With this we have come to an end of the discussion. Do you want to add anything before we end the discussion or if do you have any questions you want to ask?

Thank you for your time and in case you want, you can get in touch with us through the details mentioned in the consent form.

****

Summary:

The interview was conducted on 26/07/22 with a recently deliver mother's home. The interview was interesting but she did not explain things associated with the FP experience very well because she does not have a history of using a contraceptive method. She is working in a government office as she told us explained things in a good manner and her explanation is nice during the interview.

Discussion

Rapport Building

Thank you for proving consent to participate in the discussion. There are no right or wrong answers, please share your frank opinion, as it will help us in understanding the situation better.

Enquire about her recent delivery experience. An example is given below:

Please tell us about your recent childbirth experience. Where did you deliver? Who attended you during delivery? If you deliver at health facility how far is it from your home? How do you travel to the health facility?

Assets and Resources

Discussion

Now we are going to discuss about the Healthcare resources and assets available to recently delivered mothers in your area.

1. Health services (PP and PA family planning services) availability: Can you tell me the types of healthcare facilities that provide PP family planning services in your area?
   a. What types of FP services are available at the healthcare facilities?
   b. Are these services equally available to boys, girls, young, old, married, unmarried, people from different geographic locations, religious or cultural background, language etc.? Please tell me more

Family planning is available at the health center. Another higher level is the hospital. First we go to the health post, and if above health center we go to the hospital. The service is available for those who want to use the service, but not available for those who don't want to use it, I say it is not available. It is free will and it is already available for those who want to use it.

2. Accessibility of the PP family planning services
   a. Can you tell me how far the PP family planning services providing facility is located? How long does it take for you to access the facility?
   b. What type of transportation means do you use to reach the facility? How easy or difficult is it to use the transportation services?
   c. Is there any cost associated with accessing the PP family planning services? Please tell me more about this
   d. Is the PP family planning service open at all time and is accessible with short waiting time at the healthcare facility? Are there types of FP methods of users' choice in the healthcare facility? Do healthcare providers receive and treat users with respect and dignity?
   e. Are these services equally accessible to boys, girls, young, old, married, unmarried, people from different geographic locations, religious or cultural background, language etc.? Please tell me more
   f. How easy could you access the PP family planning service? Do you think your access to the PP family planning services is affected because of your background? How?

My house is so close and near to the health center. It will not last more than 7 minutes. It is very close to the health center. Most of the time I use motorcycle because I work on it. I have also friends and I can use their motorcycle in case I don't have mine. But it is easy to come here for us.

The health center is open and gives the service whoever wants to get it. When we come here we easily get services whenever we come. When my wife comes here she get a respectful care.

I don’t know whether they give for everyone who asks the service or not. But I think they will give for those who come and ask them the service. I think the door “health center” is open for anyone who uses it.

3. Means to access the PP family planning services
   a. Please tell me about your household resources and assets. Please tell me the resources and assets belong to you.
   b. How is the use of the household resources and assets decided? Who makes the decision? Do you involve in the decision making? How do you think that affects your access to the healthcare services including PP family planning?
   c. Please tell me about your household income. Please tell me about your own income.
   d. How is the use of the household income decided? Who makes the decision? Do you involve in the decision making? How do you think that affects your access to the healthcare services including PP and PA family planning?
   e. In your community how is use of PP family planning seen? Is it acceptable for women to use PP family planning? Why/why not? Please tell me more

Thank God life is not so bad for me. I work as daily laborer and motorcyclist. I work anything that is available. The income condition is not known. More than income the expenses is high. I have an ox and it is shared with other guy. I don’t have any other animal. I have some plot of land , but there is no use since the crop is already died by the recent weather condition. The income is small.

Regarding the decision making we make a joint decision making. It is impossible to live without discussion. Concerning family planning she took the decision by herself and she became very sick then after. I have to go different places to treat her and finally she is well now. But I stopped her from using it because she became sick after using FP. She told me later after I took her a lot of clinics and traditional healers to treat her. Now she is not using it.

Family planning hurts a lot because it needs economic capacity. You have to get a good food and drink to use family planning. Unless it is dangerous and hurts a lot. So, I believe it harms a lot. If there is no milk in the house to drink, it hurts a lot. I think there are three types of family planning. they say there is for 3 months, three years and six years. In some cases after taking FP, birth can disappear. I know a guy who brought his wife to remove it “FP” because he wanted to have a child. The woman was sick after giving the previous birth and she developed mental health problem then at the health center they gave her FP. After a while her husband wanted a child and he brought her to remove it “implanon”. So, there should be an agreement b/n husband and wife.   Husbands doesn’t allow their wives most of the time because after using FP, wives complain of health problems. They complain dizziness and husbands don’t want to take them to the facilities and spend money by the problem caused by using FP because by itself it drinks blood. Due to that reason husbands prevent wives from using FP.

4. In your opinion what should be done to improve access to the PP family planning services for all recently delivered mothers in your community?

If there is agreement and consensus between husband and wife it is possible to increase the use. They have to agree first before she decides to use FP by herself.

Practices, Roles, and Participation

Now we are going to discuss about your women's and your role and responsibilities.

1. Please describe to me the typical women's roles in your community?
    - How are women and girls occupied over the course of 24 hours? Are there seasonal differences in how women and girls use their time?

They are responsible for all household activites. They take care of their children, cook , clean the house and work on other activities in the household. Their main roles is helping the house by doing those activities.

2. What kind of work do you do? Do these works change when your age or status changes (young, unmarried and married, older)?

I work as motorcyclist and as daily laborer as well. I already told you this.

3. Do you have barriers on your access? What barriers? How do the barriers/restrictions influence your access to the PP family planning services and supportive social networks?
    - In your community do the restrictions/barriers change by social characteristic, such as (dis)ability, economic status, health status, educational level, religion, culture, geographical location, and marital status?

If husbands decide and manage themselves "use natural family planning FP" it can restrict from using FP "PP/PA". They can still use FP if they agree and discuss to use it though. If there is no agreement, it can cause a problem and conflict between themselves. Due to that reason people may not use FP. My wife is not using it because she got sick by using FP.

4. Do you participate in any family, community, government, or other social networks organizations? Please tell me more.
    - Please tell me how your participation in these organizations affect your PP family planning services use

I usually spend most of my time outside. I heard some women were attending a conference where they discuss about women's health and about family planning. I don't have much information about this.

5. In your community do women assume leadership roles? What types of roles? Do these roles change by social characteristics, such as (dis)ability, economic status, health status, educational level, religion, culture, geographical location, and marital status?
    - Please tell us about your own experience with assuming any leadership roles?
    - Please tell me how your leadership roles affect your PP family planning services use

I don't know anyone in this locality who came to the leadership role. In nearby area I know a woman who leads other women and others in who lead other women. I also know other women who do policing in the market places. These women are not teaching anything related with family planning.

6. In your community who decides at what age should people marry? What are the reasons for a women getting married at younger or older ages?
    - Please tell us about your own marriage experience? How old were you when you marry? What are the reasons you she get married at that age?
    - Please tell me how your age at marriage affects your PP family planning services use

Nowadays people use mobile phones and girls decide by themselves after discussion with their partners. They put love as the main reason for them to marry early. Most of them marry early. Some girls migrate urban areas to work and others opt to marry than suffering. Some girls marry after they fail in national examinations.

7. What are your suggestions to improve women's roles and participation to enhance the use of PP family planning services?

Knowledge, Beliefs, and Perceptions

Now we are going to discuss about the issues faced by adolescents and women in accessing PP and PA FP services in your opinion.

1. In your community what is appropriate behavior for recently delivered woman regarding use of PP and PA family planning services? How do they differ from adolescent girls and young women?
   a. What is the appropriate behavior for a recently delivered mother? How do these believe influence health behavior including the PP family planning use?

There are different thoughts in the community. It is up to the individual household to decide what is best for them. For example it is one week since my wife gave birth. The community doesn't want such women to use FP. They say it drinks/sucks blood of women. If someone has no economic capacity to avail a good food, and milk and other nutritious food, it is good. If not, it is not a good thing to use family planning. if there is no availability of good economic condition in the household, using family planning is not good. For example my sister was severely sick for a long time after using FP. Thanks to her brothers who took her different places to treat her. Now she is alive after all this treatments. If someone has a good economic power, it is good to use FP. This is actually what the community believes. Those with a good economic condition should use FP, others shouldn't.

2. What are the social beliefs and perceptions that share women's expectations and aspiration for PP family planning?
3. In your opinion who should make decisions regarding use of PP family planning services? What types of decisions do women make regarding use of healthcare including PP family planning services? What types of decisions regarding use of healthcare including PP family planning services are made jointly?

First wives can arise the issue " about using FP", but the husband should make the decision whether she should use it or not. If not, there will be a conflict between themselves and it will create a lot of problems in the household. He may agree or not. So, she should ask him and he should make the decision to use FP.

Legal Rights and Status

Now we are going to discuss about the marriage related process prevalent in this community.

1. In your community are women who delivered recently legally allowed to receive PP family planning service?
   - Is these same for boys and girls, people from different age groups, education background, ethnic groups, socio economic classes, excluded groups? How are they different? Why/ Why not?

The law allows them to use FP. There is no law that prevents from using FP. But it is up to the individuals to decide whether to use it or not.

2. How do recently delivered women access PP family planning information and resource from the healthcare facilities?
   - Is these same for boys and girls, people from different age groups, education background, ethnic groups, socio economic classes, excluded groups? How are they different? Why/ Why not?
   - Do the people in your community support recently delivered women to access PP family planning infatuation and resources? How do they provide support? Who are these people? Why do they provide support?
   - Are there certain groups in your community who do not support this? Who are these people? Why don't they support?

The information is from the educated ones. They are health care providers who were trained about it. I don't know anyone who support or don't support it. What I usually here is some women removing it "implanon/jaddele" after suffering some health problems. This is a common rumor in the community.

3. Please tell me your experience with accessing healthcare information and resources including PP family planning.
   - Do you feel you have a right to get healthcare information and resources including PP family planning services? Why/why not?
   - Do you think your experience is different from other people in your community? How? Please tell me more
   - Is there legal mechanism you can follow to ensure you get quality healthcare information and resources your need? Please tell me more

Yes, I believe I have the right. I can consult the health staffs and get information. In the future I want to manage myself "use natural FP" method than using it. I think it is better to give women something to eat /economic support when they are providing FP.

With this we have come to an end of the discussion. Do you want to add anything before we end the discussion or if do you have any questions you want to ask?

Thank you for your time and in case you want, you can get in touch with us through the details mentioned in the consent form.

****

Summary

The discussion with this informant was conducted late afternoon on 27/07/22. We have tried to find from the recently delivered women husbands and we finally found him after third trial. When we explain the purpose of the study, the informant looks like he expects something in return like money. we have to explain him repeatedly and friendly that it will not have direct monetary or other return. He looks like less interested as we go through long questions and we cautiously managed until the end of the interview. Generally, we the interview has captured some important responses and misconceptions

from the informant. As part of the ethical dimension we have made clear the informant about myths and misconceptions on using FP. We also counseled on the availability of options incase on method is causing side effects.

In-Depth Interview conducted with MCH Focal at Sod Health Center by Degnesh Dawit

.

Sociodemographic information of interviewee

Code	Keble	age	sex	Educational-status	Job responsibility	Marriage-status	Work experience
IDI_SHC_MCHF_230622	Merkato	35	F	BSC midwife	MCH focal	Married	8 years

Assets and Resources

Discussion

I; Health services (PP and PA family planning services) availability: Can you tell me the types of healthcare facilities that provide PP and PA family planning services in your area?

R; this healthcare facility is one of providing center of postpartum and post abortion family planning services. After delivery, we check for the presence of second baby then we remove the placenta and we can give them long term family planning services based on their choice, and their agreement. However, to do this first we counsel them starting from their ANC follow up to time and she has been counseled to receive family planning service after she come to this health center to give birth before latent phase of labor. After delivery, she has awareness regarding the types, their advantages and side effects, and then we can provide them based on their need. At that time, we encourage them to receive IUCD because the time is more suitable to insert IUCD than other time, because their uterus cannot returns back to its normal position that is why it does not create discomfort for them.

I; what types of FP services are available at the healthcare facilities?

R; *there are all kinds of family planning services we have in our health facilities, like, Jadelle, implanon, IUCD pills and post pills. However, especially we counsel them to receive long acting*

family planning methods because they should stay and fed breast their child at least for two years before getting again. Among them, also as I told u above we encourage them to receive IUCD.

I; Are these services equally available to boys, girls, young, old, married, unmarried, people from different geographic locations, religious or cultural background, language etc.? Please tell me more

R; to say equal services available to all it is difficult to me because currently in case of males only condom is available but for women there is so many options like long acting or short acting, there are permanent methods for males like vasectomy but it doesn't started in our setup. Even the boys' need condom we put it in mini boxes in different places of health center compounding. Despite this difference, all other services are equally available for all age groups, and people from different sociodemographic background.

Accessibility of the PP and PA family planning services

I; Can you tell me how far the PP and PA family planning services providing facilities are located? How long does it take women to access the facilities?

R; two Keble population of this catchment area located in Bossa kacha (the name of Keble) and kokate (the name of Keble) located in far from the health center relative to others. For them to come this health center it takes around 40 minutes through walk but it takes around 20 minutes through Bajaj transport.

I; What type of transportation means do they use to reach the facilities? How easy or difficult is it to use the transportation services?

R; *for the case of delivery they use Ambulance of health center, we have almost two ambulances which can give service for population resided in catchment area. We give them the phone number of ambulance's drivers during their last ANC period. Most of the time this services given during delivery time they do not need any other transportation. If they do not get Ambulances when they need it due to technical issues related to Ambulance we orient term mothers ahead through health extension workers about they should not wait for ambulances for specific days and come quickly when they feel signs and symptoms of labor by any means of transportation. By considering such kind of problems, we counsel them during last ANC visit to prepare some*

money for transportation if she do not get ambulance, to buy soft drinks, and tell her to prepare towel to wrap newborn. Because of she has been told to prepare herself in any aspects as much as possible she do not face that much challenge even if she don't get Ambulance. Due the ambulance, related problem if they lack ambulance they come with Bajaj transportation and it is not difficult to them to get it. Some of mothers refuse to receive family planning immediately after delivery even after counselling and they choose to take it after 45 days of delivery, such kind of mothers uses either Bajaj or motorcycle transportation to receive the service.

I; Is there any cost associated with accessing the PP and PA family planning services? Please tell me more about this

R; We don't let her to pay for the services all such like services are provided to women freely in country level in governmental health facilities. Even currently we are suffering from lacking of gloves, but from that we get few we prioritize it for maternal case. They may pay for their own costs like to buy soft drinks etc.

I; Is the PP and PA family planning service is accessible at all time at the healthcare facilities?

R; in our health center the service is provided for 24 hours. We are working in three shifts, two professionals who entered at one o'clock exits at eight o'clock and other two enter at eight o'clock exit at two o'clock at night, and another two enter at two o'clock at night and exit at one o'clock at morning time. So the door is open at all three shifts, and ready to provide for anyone who needs the service at any time.

I; Are there types of FP methods of users' choice in the healthcare facilities?

R; first we counsel them based on the type of method existed in our hand we tell them about which is exist currently in our hand or not. Then we can provide them all kinds of methods in our hand and they make choice based on their interest and advantages and side effect of drugs. Even some of them prefer to use condom and it is their right to use any kind of service they want to use. One of short acting family planning methods used for 3 months (Dipo) is not always available for women who needs it, beyond this all are available according to their need.

I; Are these services equally accessible to boys, girls, young, old, married, unmarried, people from different geographic locations, religious or cultural background, language etc.? Please tell me more

R; yes services equally accessible to all persons from different background, for example if one woman come from Addis Ababa or Arbaminch or this nearby catchment area they all can get similar service regardless of their language, religious or cultural difference.

I; how easy could they access the PP and PA family planning services?

R; *It is so easy to get this service because there is no related costs, the only thing required from them is coming to health facility and being ready to receive it.*

I; Do you think that their access to the PP and PA family planning services are affected because of their background? How?

R; *not at all, because when we graduated from medical school made oath not to do such bad acts. So we treat them equally regardless of their background, they may come from elsewhere. Their utilization is only based on their interest, no any other criteria is needed.*

1. Means to access the PP and PA family planning services

I; Please tell me about your facilities' resources and assets.

R; Resources of this health center are, service provision rooms, tables, chairs, delivery coch, professionals, customers, drugs, syringe, gloves, FP method drugs, pillow, blanket. delivery sets, strilizer customers etc.

I; Please tell me the resources and assets belong to these health facilities to provide PP and PA FP service.

R; resources found in our facilities that used to provide PP and PA FP service. *are glove, syringes long acting and short acting methods, like IUCD, Jadelle, Implanon, pills, Dipo and condom. Immediately after delivery we counsel them to use IUCD and Jadelle implant, because it is better. If they do not receive short acting at that time and we encourage them to receive especially IUCD at that time because it is suitable to insert it at that specific time because their uterus not returns and IUCD does not contain hormones, it is simply device.*

I; How is the use of the facility resources and assets decided? Who makes the decision? Do you involve in the decision making?

R; The facility resources and assets decided based on the requirement and the customer flow and need, I'm one of body who involve in decision making regarding resource allocation, after

decision made at every two weeks case team coordinator gives me a form to get out the drugs then by using that form I get the drugs from the stock, as our need. We get out the drugs before our preexisting drugs become zero level.

I; How do you think that affects access to the healthcare services including PP and PA family planning by women?

R; *I t is my pleasure to serve mothers regarding this issues because, if a mother get it immediately she can take rest before getting another pregnancy and also her child can get also sufficient breast milk. so im grateful for doing this kind of work, especially after taking the PCG training. I am motivated to serve mothers who are in need of this service and to serve them more than we serve them before. Another thing we got from PCG training is making our service attractive and creating interests and love among mothers regarding PP and PA FP services Training more of focuses on counselling mothers until she understands our concern and being assure of she understood us either well or not. My presence there plays a specific role in making availing of those resources needed during PP and PA, because I know the women preference and which drug is more important at that time*

I; Please tell me about income of in this health facility. Please tell me about your health facility's income.

R; Actually I don't know specifically about income of this health center it is primarily the activities of professions' who work in finance, but mostly the source of income in this health center come from customer they for drugs and laboratory, then that money serves back to replace drugs and laboratory reagents

I; How is the use of the health facility income decided? Who makes the decision? Do you involve in the decision making?

R; *in the beginning of the year the budget of this health center is estimated based on the income (from inside and outside) and the customer flow and their need, The decision made regarding the income is made jointly with head of this health center and workers of finance of this health center.*

I; How do you think that affects their access to the healthcare services including PP and PA family planning?

R*; I don't think so because we can get all of family planning drugs freely through international aid, but we can get only gloves and syringes by buying. We request them what we want as MCH then they can provide us.*

I; In your health facility how is use of PP and PA family planning seen?

R; The utilization of PP and PA FP is very low currently because most of mothers not understood about PP and PA FP well, but few of them who get counselling during ANC and labor until delivery are voluntary to receive it. Those who have follow up in another facility and come to here to give birth most of the time refuses to receive the service immediately after delivery they say I will come back after 45 weeks of delivery. We do not enforce those mothers to receive the service because it should be based on their interest and choice. The only thing needed from us is creating awareness regarding the service.

I; Is it acceptable for women to use PP and PA family planning? Why/why not? Please tell me more

R; *most of the time it is not acceptable among mothers because it is new for them to receive FP method immediately after delivery, it is common to receive FP methods after 45 days of delivery when they come to receive vaccination to their baby. It got acceptance only around few of them because its new beginning for us also in addition to them. Even giving counselling regarding this issue during ANC follow up is not started well, they think, as it is painful situation for them. When we recommend them, they may say " ohh I'm in pain, I have abdominal cramp, or lower back pain due to delivery, so how can u tell such kind of things at this time/ situation is it possible? Why not I take it after delivery? Ohh sister my God forgive u"*

I; In your opinion what should be done to improve access to the PP and PA family planning services for all women in your community?

R*; It is possible to increase the utilization of PP and PA FP by creating awareness on them in collaboration with Health extension workers, HDA and positive deviants, like other packages of HEWs. Currently around 7-8 professionals trained on it and started to serve the communities, this is good thing and encouraged to access additional mothers. This all concerned body should engage in counselling starting from ANC first visit to delivery to think for which family planning method she wants to receive after delivery and they should convince her to take PP FP*

immediately after delivery by telling her the related consequences like who knows unwanted/ unplanned pregnancy may occur to u before 45 days of delivery.

The professionals should show respect, care compassionate when they counsel the mother during her ANC visit. They should show the options that which would you choose after safe delivery if it is the will of God?

Practices, Roles, and Participation

I; Please describe to me the typical MCH focal roles in your health center?

R; As MCH focal my role is providing the needed instruments (materials for all working classes under my controls, preparing any materials needed for either delivery or family planning services and controlling either the staffs working and serving the customers according to their schedule or not

I; How is health center MCH focal occupied over the course of the day?

R; Over all controlling of activities undergoing according to scheduled time or not is my day to day activities in addition to that reporting to head of health center if I face any challenge and trying together to solve the problem, this one part of controlling, as a professional I m serving the clients regarding their need according to my schedule.

I; Are there seasonal differences (campaign/political assignment) in how you use time?

R; yes most of the time I have busy schedule, for example if I have meeting at day time and work shift at night time, at the same day, this may make me busy, because I have house, marriage, children and this all things need my existence. This kind of busy schedule my happen may four or five times per week.

I; As a health center MCH focal, what kind of work do you do?

As MCH focal my role is providing the needed instruments (materials for all working classes under my controls, preparing any materials needed for either delivery or family planning services and controlling either the staffs working and serving the customers according to their

schedule or not. As one professional, I am serving the community according to predetermined schedule.

I; Do these works different when facility status changes (Rural, urban and geographical locations)?

R; no since its health institution the activities should be done is similar elsewhere.

I; do health center MCH focal like you have restrictions/barriers on the accessibility? What restrictions/barriers?

R; As you know that this service should be given immediately after delivery or abortion as much as possible but most of the time they wants one extra person (their mother, husband relatives, friends) with them during labor up to delivery to assist them, to give them drinking water or soft drinks, to support them when they go to toilet. We health professionals allow her to be with someone which she prefers to be to share her pain psychologically and assist her. However this mother counselled from first ANC visit up to delivery, but the person who are assisting her at that time have no idea about the issue.

Another thing, which can be barrier to access the service, is their awareness status I can say they have no awareness at all regarding PP and PA FP service.

I; How do the restrictions/barriers influence the access of the PP and PA family planning services and supportive social networks?

R; If a woman come with her is her husband it is good opportunity for us to counsel together and to convince them together, but most of the time they prefer to be with their mother, sister or cousin. Such condition can *Taking someone with her during delivery time most of the time let her to refuse the actions even though she agreed to receive it before. If a husband unable to come to health center he sends his wife with her/his mother or other relatives, although those persons with her makes conflict with health professionals like by saying , is her husband agreed, is he put his signature of agreement? What an evil act how can you do such kind of thing at this time? Who allowed you to do this? Is this the right time to do such things? Don't you have another time to do such things? How can you do a kind of things in a mother with a pain?. They think that the service we are giving to mother are given by the enforcing mothers not based on agreement because they do not know about we counselled and convinced her during her ANC*

visit. They make their sound become loud and collects other people came with her, at this time she also can change her idea even she agreed to receive it before. This kind of things can be barrier to access the service for some mothers.

Another one is because of they have lack of awareness regarding the PP and PA FP service they think that taking long acting family planning especially loop before their uterus retuned back can create a pain and discomfort for them, so they have fear to take it before 45 days of delivery. They may say that let me get relief from this pain, let me get rest, let me think over it. So creating awareness among community should be prior thing.

I; In your Health center do the restriction/barriers change by social characteristic, such as (dis)ability, economic status, health status, educational level, religion, culture, geographical location, and marital status?

R; For the mothers their educational background is health it is easy to convince them, because they priorly the mechanism of action of family planning methods, but for others it is difficult to bring behavior change within short period of time, so educational status and educational background matters

I; Do you participate in any meetings, workshops, trainings, pregnant women conference, family, community, government, or other social networks organizations? Please tell me more.

I; yes, actually not only me all midwives participate in pregnant women conference/forum every month, at that time we teach them to follow ANC attentively, about danger signs of pregnancy, birth preparedness. Tell to them should come to health center by calling to ambulances early when they see labor signs and symptoms and promise to help them during labor. Another one is there is the project the called Engender health which supports us and gave training to around six mid wives with me regarding PP and PA FP. The other one is on job training, which was conducted in in this health center and gave capacity building training for three mid wives from FP, three from ANC and Three from delivery service provision center.

I; Please tell me how your participation in these organizations affect the PP and PA family planning services provision

R; *those training created awareness to me to perform those activities by knowledge and to convince mothers accordingly*

I; In your health center do women assume leadership roles? What types of roles?

R; In our health center there are several women assumed in leadership position, like MCH, Case team leader, MCH coordinator etc.

I; Do these roles change by social characteristics, such as (dis)ability, economic status, health status, educational level, religion, culture, geographical location, and marital status?

R; leadership assumption is not based on or affected by sociodemographic characteristics of some body, but it is based on the women's hard work, can she substitute other professional when he/ she missed from the work environment, does she respect mothers when she treating them? Does she show compassionate; does she have patience when she treat mothers during delivery? A person who comes to position should get witness for her hard working from others.

I; Please tell us about your own experience with assuming any leadership roles?

R; personally I count mothers pain and situation as mine, treating mothers in respectful and compassionate way. If one of my colleague missed from workplace due to disease or other urgent case, I may substitute them. Working for the satisfaction of my clients rather than mine, if I assumed to do in delivery class, I do not restrict myself in delivery class also mothers in ANC or FP class who needs my help. When mothers come from their home they plan to return back immediately, if they stay too much time to get the service they are not voluntary to come again at that health center at that time, at that I scarify y time even my lunch time or by not going to home early without serving them.

I; Please tell me how your leadership roles affect the PP and PA family planning services provision

R; my primary interest is their satisfaction if she is happy to receive the PP service after delivery I m voluntary to scarify my extra time by doing so.

I; In your community who decides at what age people marry? What are the reasons for getting married at younger or older ages in this community?

R; *most of the time their family decides their age of marriage or the persons who are going to marry also decides their age of marriage.*

I; Please tell me how their age at marriage affects their PP and PA family planning services use

R; *for me their marriage during marriage do not affect their utilization of PP or PA FP methods, since they married to give birth there is nothing affects them from using it. Those whose age greater than fifteen allowed to use family planning methods based on their choice. Sometimes those married after the age of 35 are not volunteer to use PP because they are in need to bear additional baby.*

I; what are your suggestions to improve women's roles and participation to enhance the use of PP and PA family planning services?

R; *The health professionals, especially who works MCH department should be responsible and take part in all activities should be done regarding PP and PA FP, like creating awareness among reproductive age group mothers, creating awareness for pregnant mothers during their forum, and counselling and convincing them during their ANC visit, through one to thirty leaders, with HEWs.*

Knowledge, Beliefs, and Perceptions

Now we are going to discuss about the issues faced by women in accessing PP and PA family Planning services in your opinion.

1. In your Woreda what is appropriate behavior for a woman after delivery and abortion regarding the use of PP and PA family planning?

R; in our community most of the time recently delivered mother should stay atleast six weeks at home and come to health center after 45 days of delivery to vaccinate their baby, even some mothers who have mother or relatives with them not come to vaccinate their they just send their baby over them. Regarding immediate taking of family planning, they complain to receive it they say as they are in pain, they will come back after 45 day of delivery to receive it, they think that receiving IUCD at that time may create severe pain for them.

I; How do they differ from adolescent girls and young women?

R; regarding young or adolescent woman, they can use at any time when they needed it. When they come to health center they decided to receive it, the only thing required from health professional is showing them options and telling about its advantages and side effects

I; How do these believe influence health behavior including the PP and PA family planning use?

R; *It may affect their utilization of PP PA FP methods*

I; What are the social beliefs and perceptions that share women's expectations and aspiration for health care including PP and PA family planning?

R; the social beliefs and perception regarding this service is difficult, they may may why you fast to take such think at this time? Why don't you take rest? What motivated you to take this since you are in pain? Do you have interest to do sexual intercourse with your husband before stopping of bleeding? Why you did this?. They count it as shameful thing before drying of her blood

I; In your opinion who should make decisions regarding use of PP and PA family planning services?

R; In my opinion the decision made between her husband and herself no one should enter between them regarding this issue. Because it the responsibility of both of them to grow up their child.After counselling the mother we call her husband to confirm.

I;What types of decisions do women make regarding use of healthcare including PP and PA family planning services?

R; most of the time she doesn't discuss with her husband and decides to receive by herself, at that time when her husband hears about it he lets her to remove it immediately, in some scenario we heard such kind of thing.

I; What types of decisions regarding use of healthcare including PP and PA family planning services are made jointly?

R; during delivery is the ideal time to get most of husbands at that time we have to teach and convince them together and give which they prefer. When we want to counsel them together, we tell other social to stay outside and wants time with her husband only.

Legal Rights and Status

I; In your community are women who delivered recently legally allowed to receive PP and PA family planning service?

R; *it is legally allowed because we do not enforce her to take it, it is 100% based on her interest, we can give the service after counselling and convincing her. If she is not voluntary, to receive it do not enforce her it is her right to take or not after understanding it. If it is legally not allowed, how the government trains around six up to seven professionals to do those activities*

I; Is these same for boys and girls, people from different age groups, education background, ethnic groups, socio economic classes, excluded groups? How are they different? Why/ Why not?

R;The activities done is similar for people from different sociodemographic background, but it is not common in our setting to give them family planning service after delivery of their wife

I; how do recently delivered/aborted women access PP and PA family planning information and resource from the healthcare facilities?

R; when they come to health facility to deliver they can get counselling and service regarding PP PA Methods,

I; Is these same for boys and girls, people from different age groups, education background, ethnic groups, socio economic classes, excluded groups? How are they different? Why/ Why not?

R; yes it is the same for all

I; Do the people in your community support recently delivered/aborted women to access PP and PA family planning information and resources? How do they provide support? Who are these people? Why do they provide support?

R;Not all people support recently delivered/aborted women to access PP and PA family planning information and resources, because most of people for themselves have no clear understanding on utilization of FP, the information is not reached that much for all community members even most of health extension workers and one to thirty leaders are not awared to this much. However, all health professionals including those who works MCH classes or other support the idea, like by creating awareness among them and counselling them. Because the health professional understood well about the service

I; Are there certain groups in your community who do not support this? Who are these people? Why don't they support?

R*; yes most of people from our community opposes the activities because they have no understanding on it and it is not practiced among the community so far.*

I; Please tell me women's experience with accessing healthcare information and resources including PP family planning.

R; *their experience of getting health care information is they get information regarding PP is from health facility starting from first ANC visit up to delivery*.

I; Do you feel they have a right to get healthcare information and resources including PP family planning services? Why/why not?

R; *they have the right to get the service and information which they prefer, because they are also citizen of this country.*

I; Do you think women's experience is different from other people in your community? How? Please tell me more

R; *for me their all experience of getting the service and information is similar, may be a woman who doesn't have history of ANC visit can get the information during delivery when she come to health center.*

I; Is there legal mechanism you can follow to ensure they get quality healthcare information and resources they need? Please tell me more

R*; since I am MCH focal I try to look over whether the activities were going according to done is mothers get the quality service or not, directly or indirectly, when I say directly I ask mother whether she counselled properly and get the service she wants or not, or indirectly means by just watching what they are doing. If a mother complains about not getting of required service, I do meeting with that specific professional and try to solve the problem. If they get the required information and service from health center due to different reasons, they may get it from HEWs because they follow them during their postpartum period. For example, those who come from the rural Keble can get family planning from HEWs because they are trained on it especially implant*

****

Summary

This interview conducted with MCH focal at health Sodo health center in 23/06/22. She was responsive and happy at that time. They finding were the service was not equally accessible for males and females. The utilization of PP fp were low. The awareness creation, counselling and convincing should be done during ANC visit. To increase the utilization involving one to thirty leaders, health extension workers should be involved in activities. People have misconception on the service utilization. The decision should be made between both husband and wife
