# Supplementary material for: Barriers and enablers to the implementation of immediate postpartum and post-abortion family planning service integration in primary health care units of Wolaita Zone, Southern Ethiopia: A baseline study for implementation research
Source: PLoS One. 2024 Jul 25;19(7):e0303809. doi: 10.1371/journal.pone.0303809 (PMC11271869; doi:10.1371/journal.pone.0303809)
Supplement: S3 File — (PDF) [file pone.0303809.s003.pdf]

ADDIS ABABA UNIVERSITY, COLLEGE OF HEALTH SCIENCES (IRB)
አዲስ አበባ ዩኒቨርስቲ፡ ጤናሳይንስ ኮሌጅ

Institutional Review Board

ANNEX 3
Form AAUMF 03-008

IRB's Decision

Meeting No: 04/2022 Meeting Date: April 23,2022
Protocol number: 031/22/SPH

**Protocol Title:** National Family Planning Integration Assessment & Implementation Research in Ethiopia		
Principal Investigator:	Meselech Assegid	
Institute:	College of Health Sciences, AAU	
Elements Reviewed (AAUMF 01-008)	☑ Attached	☐ Not attached
Review of Revised Application ☐ Yes ☐ No	Date of Previous review:	
Decision of the meeting:	☑ Approved ☐ Approved with Recommendation ☐ Resubmission ☐ Disapproved	

I. Elements approved-
1. Protocol Version No: 2
2. Protocol Version Date:
3. Informed consent Version No. 2
4. Informed Consent Version Date:

II. Obligations of the PI-
1. Should comply with the standard international & national scientific and ethical guidelines
2. All amendments and changes made in protocol and consent form needs IRB approval
3. The PI should report SAE within 10 days of the event
4. End of the study, including manuscripts and thesis works should be reported to the IRB
5. The PI should report non-compliance and unanticipated events

III. TO NERC ☐

Institution Review Board (IRB) Approval: Period from: June 07, 2022 to June 06,2023
Follow up report expected in 3 Months ___ 6 months ___ 9 months ✔ ___ one year___

Chairperson, IRB
Dr. Adamu Addissie
Signature
Date: June 07, 2022

Institutional Review Board Office
Faculty of Medicine
ADDIS ABABA UNIVERSITY
