# Supplementary material for: Barriers and enablers to the implementation of immediate postpartum and post-abortion family planning service integration in primary health care units of Wolaita Zone, Southern Ethiopia: A baseline study for implementation research
Source: PLoS One. 2024 Jul 25;19(7):e0303809. doi: 10.1371/journal.pone.0303809 (PMC11271869; doi:10.1371/journal.pone.0303809)
Supplement: S4 File — (DOCX) [file pone.0303809.s004.docx]

Table: 2 Formative Assessment analysis based on CFIR domains and GYSI components

| **CFIR Domains** | **GYSI Components** | | | |
| --- | --- | --- | --- | --- |
|  | Asset and Resources | Practice, roles, and participation | Knowledge, belief, and perception | Legal rights and status |
| 1. **Characteristics and Attitudes of Individuals** | | | | |
| Client's acceptance and use of IPPFP and PAFP varied (-/+) | √ | x | √ | x |
| Good or improving level of awareness and positive perception (+) | X | x | √ | x |
| Joint decision-making, skilled/trained and good mannered health care providers, the existence of community structure and trained Health extension workers (HEWs), multiple means to access information, and no restriction by background (+) | √ | x | X | x |
| Misconceptions (Implanon causes abortion, the belief of high income and balanced diet to use FP, use of FP harm health, Implanon migrates to other body parts, rumors, use of PAFP is sin) and stigmatizing abortion (-) | X | x | √ | x |
| Sole decision-making (mainly by husbands, distance, and lack of access to information for disadvantaged groups (-) | √ | x | x | x |
| 1. **Inner setting and context** |  |  |  |  |
| Supply and overall resource status varied across facilities (-/+) | √ | x | x | x |
| Married women provide information to unmarried, conducting Pregnant Women Conference (+) | √ | √ | x | x |
| Culture doesn't allow sex before marriage (+) | X | x | √ | √ |
| HCWs treat all clients equally (+) | X | √ | x | x |
| Staff and supply shortage, delay in supply refill/poor requesting mechanism of supplies, trained staff turnover, lack of electricity and water in facilities, the problem with health care financing system, Poor staff satisfaction (Gov't employees are not getting their whole salary at right time but half/quarter of their salary) (-) | √ | x | x | x |
| Religion barrier to abortion service, there is resistance to PPFP use (young unmarried girls don't use FP, and women from rural areas), Some care providers prevent youth from using FP and mistreat clients (-) | X | x | √ | x |
| Household food shortage is more important than FP (FP is not a priority) (-) | √ | x | x | x |
| Women marry at an early age (high sexual desire, lack of awareness and plan, parent's influence, peer pressure, environment, and teacher influence, family problem, desire to have birth to many children (-) | X | x | √ | x |
| 1. **Intervention characteristics** |  |  |  |  |
| People don’t see IPPFP/PAFP as other FP methods and understand its importance, contraceptive and other MCH services are free of charge, use Health Center income by board decision (+) | √ | x | x | x |
| The body is not prepared after birth to take IPPFP/PAFP (timing is not good) (-) | X | x | √ | x |
| High workload in urban health centers, Health centers are not refunded for CBHI (-) | √ | x | x | x |
| Private clinics charge for FP service (-) | √ | x | x | √ |
| Perceived side effects and discontinuation/ removal (-) | √ | x | √ | x |
| poor accountability among service providers and inappropriate system to achieve goals (-) | X | x | x | √ |
| Health development army’s (HDA), HEWs, husbands or husbands and wives together didn’t get trained, irregular support supervision and review meetings (-) | √ | x | x | x |
| 1. **Outer-setting** |  |  |  |  |
| Clients use of method of their choice was varied across facilities (-/+) | X | x | x | √ |
| FP is legally allowed in IPP period and Clients (including young women) have the right to choose and use FP methods and have the right to get full information, legally allowed to use safe abortion services, and HCWs responsibilities posted to clients (+) | X | x | x | √ |
| Perceive that abortion and PAFP is not legally allowed (-) | X | x | x | √ |
| The health center is far and Clients from far kebeles suffer to get transport (deliver at home), FP is not given at the household level and HEWs didn’t conduct house-to-house visits (-) | √ | x | x | x |
| Girls go to private clinics or another facility for abortion services (-) | √ | x | x | x |
| Health posts are closed at night and campaign time, post-abortion service is not always available, and there are no means to serve people in far kebeles (-) | √ | x | x | √ |
| Male partners want to have many children, Most girls give birth immediately after marriage and not use PAFP (-), | √ | x | √ | x |
| 1. **Process** |  |  |  |  |
| Preparing a plan for IPPFP and PAFP, follow up of health center performance/service quality, coaching Health Center staff and evaluating them varied across facilities (-/+). | X | √ | x | x |
| Model women provide information to the community. Besides educated women’s and kebele leaders, encourage others to use FP (+) | √ | √ | x | x |
| Women's participation in leadership increasing and it increases service uptake, Female teachers at school counsel and help girls on Reproductive health matters (+) | X | √ | x | x |
| Check reports and conduct review meetings and get feedback about the service provided, Provide FP service fairly for everyone including IPPFP and PAFP, PAC (+) | X | √ | x | x |
| HCWs do not work closely with the community and have no follow-up, Trained on abortion but not actively working (-) | √ | x | x | x |
| Use of IPPFP/PAFP has no association with women leadership and not all women involved in leadership | X | √ | x | x |
| Elders and religious leaders are not participating, and Weak implementation plan and monitoring (-) | X | √ | x | x |

**Footnote:** In table 2 below the “√” and “ X” marks indicate the reflection of our study finding that it is based on the GYSI components and matches its component. Moreover, the (-) marks indicate barriers, and (+) indicate facilitators.

**Abbreviations:** HEWs: Health Extension workers, HCWs: Health care workers, PAC: Post-abortion care, PAFP: Post-abortion family planning, IPPFP: Immediate postpartum family planning, FP: Family planning, MCH: Maternal and child health, IPP: Immediate postpartum, CBHI: Community-based health insurance, HDA: Health Development Army
