# Supplementary material for: Barriers and enablers to the implementation of immediate postpartum and post-abortion family planning service integration in primary health care units of Wolaita Zone, Southern Ethiopia: A baseline study for implementation research
Source: PLoS One. 2024 Jul 25;19(7):e0303809. doi: 10.1371/journal.pone.0303809 (PMC11271869; doi:10.1371/journal.pone.0303809)
Supplement: Attachment — Submitted filename: Response to Editor and Reviwers 11 Oct 2023.docx [file pone.0303809.s005.docx]

**PLOS ONE: Response for the editor and Reviewers**

**Manuscript ID: PONE-D-23-12106**

Title: **Barriers and enablers to the implementation of immediate postpartum and post abortion family planning service integration in Primary Health Care Units of Wolaita Zone, Southern Ethiopia: A Consolidated Framework for Implementation Research**

PLOS ONE

Correspondent author: **Mengistu Meskele**

Journal Requirements:

When submitting your revision, we need you to address these additional requirements.

1. Please ensure that your manuscript meets PLOS ONE's style requirements, including those for file naming.

We thank the editors of PLOS ONE for this critical query. We have now followed and incorporated the journal styles.

2. We note that the grant information you provided in the ‘Funding Information’ and ‘Financial Disclosure’ sections do not match.

When you resubmit, please provide the correct grant numbers for the awards you received for your study in the ‘‘Funding Information’ section

We thank the reviewer, now we have corrected this comment.

3. Thank you for stating the following financial disclosure:

"The funders had no role in study design, data collection and analysis, decision to publish, or preparation of the manuscript."

At this time, please address the following queries:

a) Please clarify the sources of funding (financial or material support) for your study. List the grants or organizations that supported your study, including funding received from your institution.

b) State what role the funders took in the study. If the funders had no role in your study, please state: “The funders had no role in study design, data collection and analysis, decision to publish, or preparation of the manuscript.”

c) If any authors received a salary from any of your funders, please state which authors and which funders.

d) If you did not receive any funding for this study, please state: “The authors received no specific funding for this work.”

Please include your amended statements within your cover letter; we will change the online submission form on your behalf.

We thank the editor. Now we have included the funding information in the cover letter and kindly ask the editor to include it at online submission on my behalf.

4. Please include captions for your Supporting Information files at the end of your manuscript, and update any in-text citations to match accordingly. Please see our Supporting Information guidelines for more information: http://journals.plos.org/plosone/s/supporting-information.

Thank you the reviewer, we noted and included the supporting information files as per your comment.

5. We are unable to open your Supporting Information file Data_Transcripts.rar. Please kindly revise as necessary and re-upload.

We noted the comment, thank you editor. I will merge the files and send it as PDF_merged file

Reviewers' comments:

Reviewer's Responses to Questions

Comments to the Author

1. Is the manuscript technically sound, and do the data support the conclusions?

The manuscript must describe a technically sound piece of scientific research with data that supports the conclusions. Experiments must have been conducted rigorously, with appropriate controls, replication, and sample sizes. The conclusions must be drawn appropriately based on the data presented.

Reviewer #1: Partly

Reviewer #2: Partly

We thank the reviewers for these important queries. No we made the conclusion in line with the finding.

2. Has the statistical analysis been performed appropriately and rigorously?

Reviewer #1: N/A

Reviewer #2: Yes

3. Have the authors made all data underlying the findings in their manuscript fully available?

The PLOS Data policy requires authors to make all data underlying the findings described in their manuscript fully available without restriction, with rare exception (please refer to the Data Availability Statement in the manuscript PDF file). The data should be provided as part of the manuscript or its supporting information, or deposited to a public repository. For example, in addition to summary statistics, the data points behind means, medians and variance measures should be available. If there are restrictions on publicly sharing data—e.g. participant privacy or use of data from a third party—those must be specified.

Reviewer #1: No

Reviewer #2: Yes.

We thank the reviewer/editors for this important query. We have now uploaded our data as well, and we have included all the participants' thick descriptions in our manuscript.

4. Is the manuscript presented in an intelligible fashion and written in standard English?

PLOS ONE does not copyedit accepted manuscripts, so the language in submitted articles must be clear, correct, and unambiguous. Any typographical or grammatical errors should be corrected at revision, so please note any specific errors here.

Reviewer #1: Yes

Reviewer #2: No.

We thank the reviewers and editor for this critical comment. We now have edited the English as indicated by the reviewers and editors.

5. Review Comments to the Author:

Please use the space provided to explain your answers to the questions above. You may also include additional comments for the author, including concerns about dual publication, research ethics, or publication ethics. (Please upload your review as an attachment if it exceeds 20,000 characters)

Reviewer #1: This is an interesting piece of work on improving FP services among women most in need.

General comment:

Though the author attempted to apply CFIR for FP services following births or abortion in the contexts of gender, youth or social inclusion, they did not manage to address the required points.

I am more inclined to reject the paper but would rather advise them to be specific and focused on rewriting the manuscript to convey a clear message.

Thank you for your comment. We accepted the comment, and we managed to be specific and focused on the revised manuscript.

The following key comments:

1. The post-partum and abortion FP services are already available services.what specific intervention or strategy was on your mind regarding FP for this group. Eg. Was Gender and social inclusion the strategy in your mind? For what strategy you tried to mention relative advantage, compatibility or complexity...? Was that for gender, youth, and social inclusion in the program or service? This is not well said.

We thank the reviewers for this concern. As a specific intervention or strategy, we have tested the model for integration of immediate PPFP and PAFP service with existing ANC, Delivery and abortion services. We have used the theory of change models for each intervention strategy. We have used three intervention strategies, namely engaging relevant stakeholders, filling equipment and supply gaps and building the capacity of community and health workers. However, it is not the objective of this paper to report the detailed intervention strategies and models tested. Therefore, as a baseline study, we studied the relative advantage, compatibility or complexity for the suggested interventions with existing interventions on which our study analysis is based. We have used GYSI dimensions mainly to develop the tools and based our analysis on them, as mentioned in the method and result section of the manuscript.

2. Individual characteristics ignored the women/adolescents' characteristics and expectations while it emphasized on health workers

We thank the reviewer for this critical concern. The inner setting and context domain of CFIR can only discuss institutional and health workers' characteristics. That means the domain only focuses on organizations' issues, not clients. On the other hand, characteristics and attitudes of individual’s domain address only clients, and we have already included them in Table 2.

3. Though the attempt to address 39 constructs of CFIR is generally fine, the effort to address all these aspects brought shallow findings for each of the five domains. Hence, important questions pertaining implementation strategies of the services remained unanswered. E.g. to what extent the FP service is accessible for post abortion or partum when they are done at home or traditionally? Contexts of the youths? Affordability when done in private facilities due to free of stigma…...

We thank the reviewer for this important concern. Sure, but we didn’t include all 39 constructs of the CFIR domains. We have used only 13 CFIR constructs to analyze and report our data and showed them in the method section of our main manuscript. We have used the RE-AIM model to measure the overall progress of the implementation. However, it is not the objective of this paper to report the detailed implementation outcome.

4. The study drew conclusions about the need to mobilize the community without assessing anything about it. Implementation of intervention strategies need to be assessed before concluded. Pls remove such conclusions. You should have explored what implementation problems of different interventions associated to Fp service (e.g: gender involvement, social inclusion, friendliness, community engagement and mobilization.....) this comment was partly indicated by comment #1.. so you should have picked several challenges and facilitators about these. If possible be advised to narrow the scope of the manuscript. This section alone is one paper. It is nice to have many focused and clear papers than one extensively but narrowly reported papers.

We thank the reviewers for this concern. As the reviewer #1 pointed it in comment #4, now we have removed such conclusions that need to mobilize the community in our manuscript as a specific intervention or strategy. We have tested the model for integration of immediate PPFP and PAFP service but it is not part of the objective of this paper to report the detailed intervention strategies and models tested.

5. All other domains are challenged from lack of focus

Please present this manuscript by separating key aspects as distinct papers so that you can be informative and clear. At the moment the main question of this manuscript is not clearly answered, the context of gender, youth and social inclusion not addressed and the conclusion looks like common sense.

We thank the reviewer for this very important query. We now have tried to get our domains focused. See Table 2 in the result section. The main framework used in this paper is CFIR and is supported by GYSI.

Reviewer #2: Review Report

Title: Barriers and enablers to the implementation of immediate postpartum and post abortion family planning service integration in Primary Health Care Units of Wolaita Zone, Southern Ethiopia: A Consolidated Framework for Implementation Research.

Version I:

Manuscript Number; PONE-D-23-12106

Review Comments

i. On the title and abstract

 Is that evaluation of the already implemented service or exploring the possibility of integration of both services?

We thank the reviewer for this critical query. This paper is part of the bigger project of implementation of immediate postpartum and post-abortion family planning service integration in Primary Health Care Units. Therefore, this is a baseline study to identify potential barriers and enablers for the implementation of immediate postpartum and post-abortion family planning service integration in Primary Health Care Units. It is to explore the possibilities of integration of both services rather than evaluating already implemented interventions. We corrected the title accordingly and included the statement that explained more about these issues in the introduction section.

 What was the problem with the non-integrated service delivery? Where is the panel analysis before this study?

ii. The background is not strong and the problem statement is mostly missed.

We thank the reviewer for this important concern. We revised the background section in the revised manuscript.

iii. On the methods section

• Inconsistent stud participants. On one hand you have interviewed those who gave birth and on the other hand you have interviewed youth who didn’t have a history of abortion. Even, I wisely guess the questions will slightly push the youth to have abortion in the future and seek post abortion family planning care?

We thank the reviewer for the concern. We recruited study participants from different groups to assess the barriers and enablers for both postpartum and post abortion services. Thus, women who gave birth were included to assess the barriers and enablers for postpartum family planning service, and the adolescents were included to assess the service on the post abortion side. The interview guides used were different for these two groups. Regarding the reviewer’s fear about pushing youth to have abortion services, we also had a similar fear when we were developing the proposal. However, the main reason for selecting the youth is that it is difficult to identify women who have had abortion services in the past because of privacy issues, and youth are the ones that use abortion services based on the quantitative baseline data we have received.

• Have you assessed the friendliness of the service by time, place and conditions?

We thank the reviewer for this concern. We have assessed friendliness of the service through using the construct of the CFIR. We have noted the findings under the sub-heading of patient needs and resources in the line number 277-280 and 376-419 as access and utilization of youth friendly service.

• The attitude and opinion of local community leaders and religious leaders were not captured

We thank the reviewer for this important query. Actually we didn’t interview religious and community leaders for their attitude and opinions. But the study participants expressed the religious and community leader’s attitude and opinions were included in the sub-heading of this paper line 455-464.

• Additionally, the saying of the women, youth and child affairs and the local administration was not captured.

We thank the reviewer for the concern. We did not include women, youth and child affairs and local administration in the study because, based on our objective, no such issue has a link with the administrative bodies on the integration of family planning with postpartum and post abortion services. In the current abortion law, a woman must not provide evidence to get abortion services if she claims any of the eligibility criteria.

• Cite the reference of the study area and the number of population and the number of hospitals and PHCU/standard

We thank the reviewer for pointing this out. We cited the reference in the revised manuscript.

• The methods section fails to respond to the qualification of the data collector and trustworthiness which is highly essential.

We thank the reviewer for pointing this out. We have included the qualification of the data collectors and trustworthiness in the revised manuscript.

iv. On the result, discussion and conclusion section

♣ The beginning of the result section is absorbing but fails to shorten, clarify, simplify and to maintain logical flow. In addition, it didn’t address the standard way of presenting qualitative research.

We thank the reviewer for this critical comment. We thoroughly revised the result section based on your comment in the revised manuscript.

♣ The discussion section should have theoretical and practical considerations and ground level explanations without missing the reality?

We thank the reviewers for these important issues. We now have included it in the discussion.

Kindly regards,
