# Supplementary material for: Barriers and enablers to the implementation of immediate postpartum and post-abortion family planning service integration in primary health care units of Wolaita Zone, Southern Ethiopia: A baseline study for implementation research
Source: PLoS One. 2024 Jul 25;19(7):e0303809. doi: 10.1371/journal.pone.0303809 (PMC11271869; doi:10.1371/journal.pone.0303809)
Supplement: Attachment — Submitted filename: Rviewer comment for authors.docx [file pone.0303809.s006.docx]

Comments

- Line 91 to 92: it says that “Studies thus far revealed that integrating family planning with other health services was still weak and indicated the need for well-designed evaluation research.”, *But, it lack references.*
- Line 102 to 104: Most previously conducted studies in Ethiopia on postpartum family planning utilization were observational studies and lacked interventional study or implementation study designs to provide evidence-based interventions to improve postpartum family planning uptake (17). How many studies you reviewed for this paragraph?
- Line 137 to 138: You said that “Moreover, three FGDs with boys and three with girls were conducted.” Since your study is about postpartum and post-abortion family planning service, how adolescents were your target population? I need your justification how you select your target population?
- Under your sample size section, would you elaborate how you determine the sample size?
- Line 142 to 143: you sad that “We adopted a semi-structured interview guide from GYSI tools and used the CIFR domains/construct “you directly adopted interview guide. Do you mean you did not modify something, please?, why you do a consultative workshop? How you contextualize the tool?
- **Line 162 to 164:** Credibility: The primary investigator spent time in the field and gathered data from the healthcare providers to make sure the study accurately represents the opinions of the participants. Please check it, is it healthcare providers?
- Under Trustworthiness section, lacks member checking? Why?, If you do it add it? If not why?
- Result section lacks who says what?
- Under Discussion section, why you discuss the finding based on domain?
- Line 500, our study has reported a household food shortage and low income in the community? What do you mean food shortage?
- Line 587: An earlier study confirms our finding and suggests the importance of conducting PWC every month. What does this means, would you make it clear, please?
- Please add the limitation and strength of the study?
