# Supplementary material for: Barriers and enablers to the implementation of immediate postpartum and post-abortion family planning service integration in primary health care units of Wolaita Zone, Southern Ethiopia: A baseline study for implementation research
Source: PLoS One. 2024 Jul 25;19(7):e0303809. doi: 10.1371/journal.pone.0303809 (PMC11271869; doi:10.1371/journal.pone.0303809)
Supplement: Attachment — Submitted filename: Response to Reviwers and Editors 23 March 2024.docx [file pone.0303809.s007.docx]

Response to Reviewers and Editors

| Reviewer/Editor quarries | Response of the author |
| --- | --- |
| Consult again for the requirement of the journal E.g. Is that only the conclusion or Conclusion and recommendation in the abstract section. Again see the Introduction for the same concern.  Try to revisit the whole manuscript for clarification, since it is very crucial because it is lacking in the manuscript E.g. if you read this sentence “less attention being given to adolescents and husbands that 38 hinder uptake of immediate postpartum and postabortion family planning”. Who gave less attention? Attention towards what?  BE consistent throughout the document and maintain its cope.  Language, grammar and editorial issue and study period • Clarity E.g., in the abstract section “…a higher rate when offered at the same time and location.” can be re-written as “…a higher rate when offered timely at appropriate site”. Secondly, in the same section “aimed to explore” can be re-written as “explored”. Again, in the result section of the result section you can avoid ‘significant’ from barriers. Also, again “service free of charge” can be re-written as well “waivered services” …etc. • Plural e.g., Conclusion • Full stops are lacking • The end or the introduction section have no study period. • The formatting is incorrect • Hence try to revisit or consult for editorial service before the next submission. | We thank the reviewer and editor for this concern. Now we have corrected the heading as conclusion and recommendation as both are in the abstract section together. Similarly, the introduction is now also corrected.  We thank the reviewer for this concern. Now we have corrected the ambiguity as “healthcare providers paying less attention to adolescents and husbands, which prevented them from using immediate postpartum and postabortion family planning services”. Moreover, we have corrected the language and grammer as indicated by the reviewers.  We accepted the reviewer's suggestions for this concern and we have now addressed the queries raised in the introduction and result section of the abstract.  We thank the reviewer and now we have included the study period. we have corrected the language and grammer as indicated by the reviewers. |
| II. Specific Comments a. On the Introduction Section It is not concise, occupied wide space and the adverse consequences and benefits the whether use of PP and PA-family planning should be also clearly depicted.    Reference 19 is not correct and try t use Guba (1972). Don’t tell us the science of trustworthiness, but report what you have already done to ensure quality.  You can avoid coding and put it under data analysis.  Incorporate report or plan for dissemination of the finding to the study are local admin and the community.    These methods still need enrichment E.g. How did you handle emerging issues and the quality of the data collectors? How did you sample? What type of Qualitative research is it?    b. Result and the Consequent Sections • Lacks clarity, concise and needs major refinement. • If I were, I will depict the summary of the findings in one domain and then describe it in detail in the consequent sentences. • Stick to the main findings and avoid unnecessary findings. • Try to slightly address the agreement and the difference between the two times of rendering service and framework findings. • The conclusion is not the conclusion.  • Put the abbreviation and acronyms separately at its due place.  • Avoid the use of ‘we” from the discussion section. • The space of the result and the discussion section should revisit/ • Compare with the comparable one • Have no recommendation • Revisit the consequent section. Regards,  Reviewer #3: Comments  Line 91 to 92: it says that “Studies thus far revealed that integrating family planning with other health services was still weak and indicated the need for well-designed evaluation research.”, But, it lacks references.  Line 102 to 104: Most previously conducted studies in Ethiopia on postpartum family planning utilization were observational studies and lacked interventional study or implementation study designs to provide evidence-based interventions to improve postpartum family planning uptake (17). How many studies did you review for this paragraph?  Line 137 to 138: You said that “Moreover, three FGDs with boys and three with girls were conducted.” Since your study is about postpartum and post-abortion family planning service, how adolescents were your target population? I need your justification for how you select your target population.  Under your sample size section, would you elaborate how you determine the sample size?  \ Line 142 to 143: you said that “We adopted a semi-structured interview guide from GYSI tools and used the CIFR domains/construct “you directly adopted the interview guide. Do you mean you did not modify something, please?Why do you do a consultativeworkshop? How do you contextualize the tool?  Line 162 to 164: Credibility: The primary investigator spent time in the field and gathered data from the healthcare providers to make sure the study accurately represents the opinions of the participants. Please check it, is it healthcare providers?  Under the Trustworthiness section, lacks member checking? Why?, If you do add it? If not, why?  Result section lacks who says what?  Under the Discussion section, why do you discuss the finding based on domain?  Line 500, our study has reported a household food shortage and low income in the community? What do you mean food shortage?  Line 587: An earlier study confirms our findings and suggests the importance of conducting PWC every month. What does this mean, would you make it clear, please?  Please add the limitation and strength of the study?  Reviewer #4: (No Response) | We thank the reviewer and now we made the introduction concise and clear.  We thank the reviewer and now we replaced reference number 19 with the correct one.Guba,  Trustworthiness reference was included as indicated by the reviewer. We included only what we have done and removed the science about trustworthiness.  We thank the reviewer for this concern. Now we deleted the sub-heading “coding” and put it under data analysis as per your suggestion.  We thank the reviewer. Now we have included the dissemination plan under the section of Ethical consideration as “We finally disseminated the findings to health extension workers, local health leaders, and the community. We also presented the paper at the 35th conference of the Ethiopian Public Health Association (EPHA) and we will publish the paper in an internationally reputable journal”  We thank the reviewers. We have corrected as indicated by the reviewers “We recruited study participants from project implementation health facilities and respective catchment communities. A purposive sampling procedure was used to identify recently delivered women and husbands, adolescents of both sexes, healthcare providers and leaders such as health center heads, maternal and child health (MCH) focal, and district and regional experts. The sample size was determined using the principle of “saturation”— women, husbands healthcare providers, and leaders were asked to participate in interviews until additional interviews did not provide additional evidence about the main themes of interest. We used a case study design using various data-gathering techniques’’.  We thank the reviewer for this important query. We deduced the redundant parts and made them concise as indicated by the reviewers. Moreover,  we have removed a very lengthy CFIR and GYSI table and included it as supplementary files.  We thank the reviewer and now we made the conclusion concise as indicated.  We thank the reviewer for this suggestion and we now have moved the abbreviations and acronyms at their due place in the manuscript.  We thank the reviewers and no we replaced “we” by ‘’this study’’. Also. We included recommendations and amended the result and discussion sections.  We thank the reviewer for these important points. We now removed Line number 91 and 92.  We thank the reviewer, now we have corrected the sentences of 102 to 104 as we reviewed one article for that particular paragraph. We corrected it as “ A previous study conducted…….”  We thank the reviewer for this concern; we just to know the abortion-related experience of adolescents. However, we encountered difficulty in identifying adolescents who had experienced abortion and we considered it as a limitation of our study. We elaborated on the sample size and included it in the method sections.  We adopted and contextualized the tool in our co-design workshop.  We thank the reviewer for this concern. The healthcare providers were also part of this study and now we corrected this section as “The primary investigator spent time in the field and gathered data from the study participants”  We thank the reviewer regarding the trustworthiness and now we corrected it as “ Furthermore, we shared the data with respondents to ensure it accurately reflected their experiences.” We provided the initial data that we transcribed to the respondent to cross-check whether it is consistent with their original (e.g. we checked it during our review meeting event that includes the participants like health care workers, women, husbands partners)  We thank the reviewer for this important concern. Now we make it clear “Our study has reported that a food shortage at household level and being from a low-income group affects family planning utilization”. participants believe that they need to have an adequate and balanced diet to use FP and those from low-income households have less chance to use it.  We thank the reviewer for this query; based on the reviewer's suggestion we have corrected the clarity issue as “ Our finding echoed an earlier study conducted by Save the Children that suggests the importance of conducting regular monthly PWCs. It can promote peer support amongst women to motivate each other to seek appropriate ANC, delivery at a health facility, PNC, and family planning” .  We thank the reviewer for this important queries. We have now included the strength and limitations of the study as “This study was based on identifying information from various study participants, data and method triangulation, and also the study used the standard CIFIR and GYSI tools as a strength. The involvement of various professionals from three universities, the involvement of Engender Health, conducting various review meetings, and making necessary corrections after the discussions with all research teams were strengths. However, the study findings are difficult to generalize to other contexts. Moreover, the research may be influenced by the researcher's preferences and personal beliefs, though we tried to bracket our ideas aside. Persuading readers who are used to precise statistical solutions might be challenging.” Excluding recently delivered adolescents and those who have had abortions from the study may limit the overall understanding of IPPFP and PAFP needs among all sexually active adolescents, as these groups may have unique perspectives and experiences that could inform future interventions. Additionally, finding ways to include these groups in research while respecting their privacy concerns should be a priority to ensure a comprehensive understanding of adolescent IPPFP and PAFP needs. |
